# Friedel–Crafts Addition of 3‑Alkylated Indoles to Aldehydes: 2‑Hydroxyalkylation Promoted by Trimethylsilyl Trifluoromethanesulfonate

**DOI:** 10.1021/acs.joc.5c03195

**Published:** 2026-04-29

**Authors:** Eric Zhou, Helen L. Xia, C. Wade Downey

**Affiliations:** Department of Chemistry, 6888 University of Richmond, 138 UR Drive, Richmond, Virginia 23173, United States

## Abstract

In the presence of trimethylsilyl trifluoromethanesulfonate (TMSOTf) and 2,6-lutidine, 3-alkylated indoles undergo Friedel–Crafts silyloxyalkylation when treated with nonenolizable aldehydes. The reaction is general across a wide range of aromatic aldehydes and tolerates a variety of *N*-alkylated indoles. Deprotection of the silylated product occurs under standard conditions, and in certain cases the initial TMS-protected product can be isolated in high yield.

## Graphical abstract

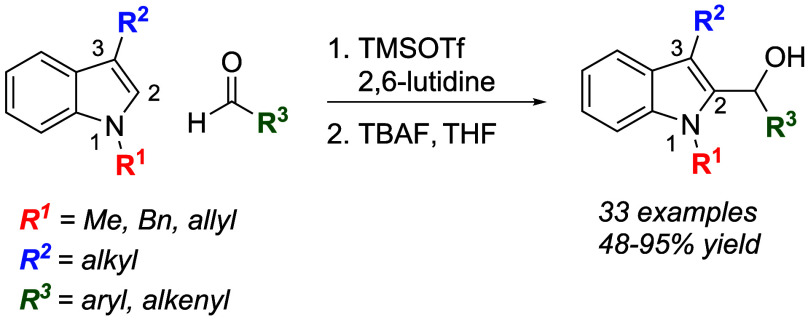

## Introduction

The indole substructure is the ninth-most common heterocyclic motif among FDA-approved drugs,^1^ and the construction of elaborated indoles^2^ and the evaluation of their biological activity^3^ remain areas of intense scrutiny. Many pharmaceutically active indole-containing compounds feature substitution at the more reactive C3 position of the indole structure, but C2-substituted indoles also appear with considerable prevalence (Figure 1). These biologically active compounds often exhibit 2,3-disubstitution as part of a fused bicycle (e.g., Alosetron,^4^ Reserpine,^5^ Yohimbine,^6^ Vinblastine,^7^ Tadalafil^8^) or 2-monsubstitution in the form of a simple methyl or bromo group (e.g., Indomethacin,^9^ Bromocriptine^10^). While other substitutions at C2 include thiomethyl (e.g, Arbidol^11^), amidoyl (e.g., Delaviridine^12^), aryl (e.g., Bazedoxifene,^13^ Rucaparib^14^), and alkenyl (e.g, Fluvastatin^15^) functional groups, these side chains seldom exhibit α-hydroxylation. Rare examples include Fluvastatin, which bears a γ-hydroxylated substituent, and Tezacaftor, which is β-hydroxylated; α-hydroxylated analogues are notably absent.

**Figure 1:**
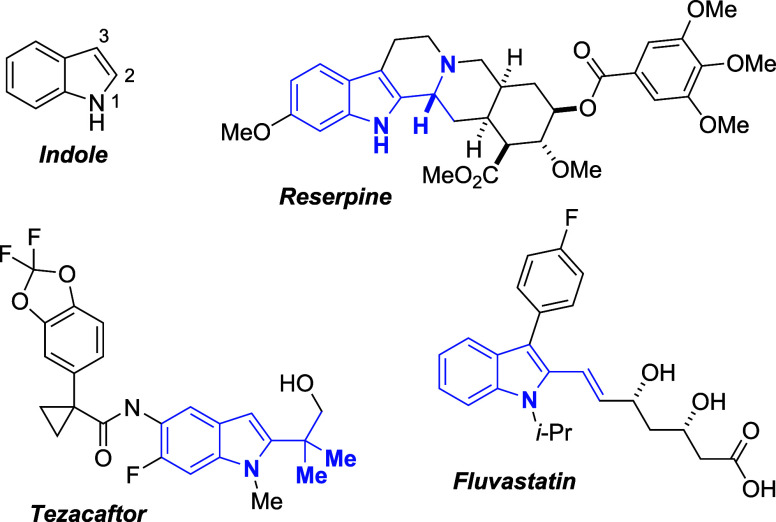
Indole numbering and representative examples of C2-substituted indoles.

Examples of 2-(α-hydroxyalklated)­indoles are scarce in the chemical literature as well. For example, a literature search for simple hydroxybenzylated indole **1a** reveals but a single synthesis, reported by Jiang and Sun,^16^ which begins from prohibitively expensive starting materials (Scheme 1a). Retrosynthetic analysis of the product, however, suggests a Friedel–Crafts hydroxyalkylation reaction between 3-methylskatole and benzaldehyde, starting materials available at a fraction of the cost of those employed in the published synthesis; indeed, skatole itself is readily available, inexpensive, and can be easily methylated with methyl iodide.^17^ Nonetheless, no examples of this potentially powerful hydroxyalkylation reaction have appeared in the literature. On the contrary, it is well established that Brønsted acid- or Lewis acid-catalyzed Friedel–Crafts reactions of these substrates produce a more thermodynamically stable product, triarylmethane **2** (Scheme 1b),^18^ a compound presumably generated when initial hydroxyalkylated indole **1a** ionizes under the reaction conditions and undergoes attack by a second equivalent of the indole.^19^

**Scheme 1:**
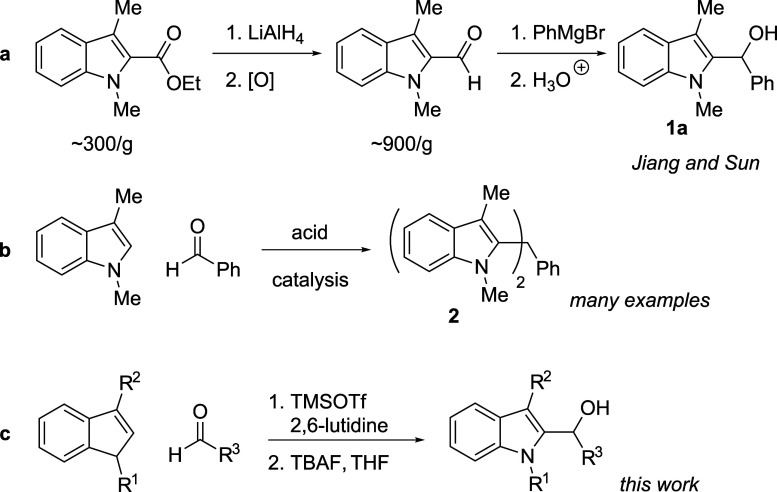
Past and Future Work

Previous results from our laboratory promised a potential convergent Friedel–Crafts route to the 2-hydroxyalkylation product. Previously, we have shown that under certain conditions, Friedel–Crafts addition of simple indoles to aldehydes in the presence of trimethylsilyl trifluoromethanesulfonate (TMSOTf) provides, after fluoride-promoted deprotection, the hydroxyalkylation product (eq 1).^20^ Similar outcomes have been observed for the reaction of indoles with nitrone electrophiles^21^ and alkylating agents,^22^ but in each of these examples the more nucleophilic C3 position of the indole dominates the reactivity. To our knowledge, no examples of 2-hydroxyalkylation reactions of indoles with aldehyde electrophiles have been reported in the literature, despite the potential to employ these products as alkylating agents^16c,18a^ for further elaboration of the indole nucleus. Here, we disclose a TMSOTf-promoted route to 2-(α-hydroxyalklated)­indoles (Scheme 1c).
1

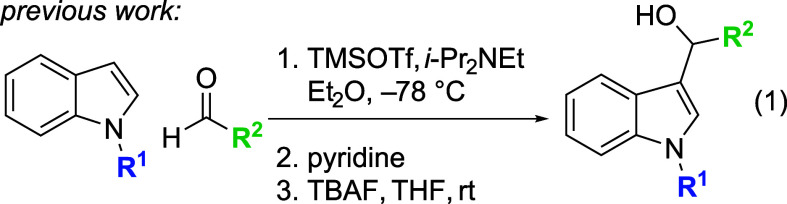




## Results and Discussion

Electrophilic aromatic substitution reactions of other heteroarenes (e.g., benzofurans, furans, thiophenes) often proceed with selectivity for the C2 position, and we speculated that employment of 3-alkylated indoles would force reactivity to C2.^23^ Initial studies targeted the generation of 2-hydroxyalkylation adduct **1a** from 3-methylskatole and benzaldehyde in the presence of TMSOTf and a variety of amine bases (eq 2). Although Et_3_N completely suppressed all product formation, both *i*-Pr_2_NEt and 2,6-lutidine provided >95% conv and >30:1 selectivity for the desired product over the undesired triarylmethane; 2,6-lutidine was chosen for further study because of the known ability of *i*-Pr_2_NEt to act as a hydride donor in TMSOTf-promoted reactions, including the deoxygenation of electron-rich alcohols similar to the desired product.^24^ A variety of solvents (CH_2_Cl_2_, THF, toluene, Et_2_O, cyclohexane) acted as effective media for the reaction, with ethereal solvents showing the highest selectivity.^25^

2

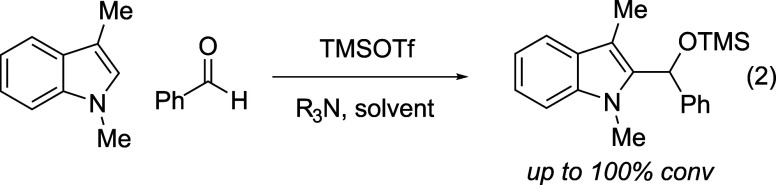




In contrast, evaluation of several other Lewis acids (LiClO_4_, BF_3_•OEt_2_, Zn­(OTf)_2_, TMSCl, and MgBr_2_·OEt_2_), both with and without added 2,6-lutidine, showed that none provided measurable conversion to either the desired product or triarylmethane **2**. Only Fe­(OTf)_3_ showed potent reactivity, achieving detectable conversion (∼5%) to triarylmethane **2** in the presence of 2,6-lutidine and 90% conversion to the triarylmethane in the absence of base, a result in accord with literature precedent.^26^ After the evaluation of these results, the TMSOTf-lutidine system was chosen for further study.

The results of a survey of various aldehyde reaction partners are summarized in Table 1. The reactivity of each aldehyde was tested with the model substrate 3-methylskatole in both THF and Et_2_O, after which the optimized reaction was performed on a 1.0 mmol scale. Aromatic aldehydes proved to be excellent substrates, with a wide range of benzaldehydes furnishing good yields (entries 1–11). Benzaldehydes bearing electron-donating substituents at the ortho position displayed exceptional reactivity, but para-substituted electron-rich aldehydes sometimes performed more poorly, suffering from a combination of lower reactivity and increased side reactions. In particular, the formation of triarylmethanes analogous to compound **2** was more difficult to suppress for these easily ionized products, and decomposition during column chromatography proved problematic for dimethylamino-substituted product **1k**. Steric bulk on the aldehyde, however, was easily tolerated, with even the highly encumbered 2,6-dimethylbenzaldehyde reacting in a respectable yield (entry 8). Larger arenes such as naphthaldehydes, as well as electron-rich heteroaryl aldehydes, provided excellent yields (entries 12–15). In contrast, ionization of the highly conjugated cinnamaldehyde adduct was difficult to suppress, and a number of unidentified side products formed in addition to a moderate yield of the expected alcohol (entry 16). Attempts to extend reactivity to aliphatic aldehydes provided no desired product, even at elevated temperatures, and although isobutyraldehyde did undergo conversion to its enol silane, the indole nucleophile was recovered unreacted (entries 17–18).

**Table 1:**
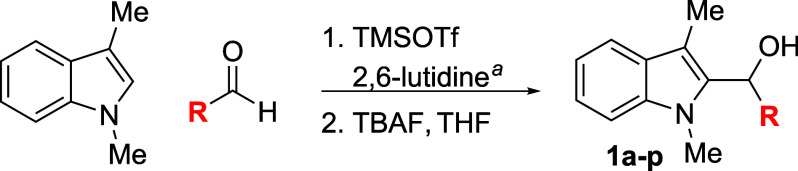
Reaction of 3-Methylskatole with Various Aldehydes

entry	R	solvent	temp (°C)	product	yield (%)^b^
1	Ph	THF	0	**1a**	75
2	4-(NO_2_)Ph	THF	23	**1b**	74
3	4-(F_3_C)Ph	THF	23	**1c**	67
4	4-FPh	Et_2_O	23	**1d**	74
5	4-BrPh	THF	23	**1e**	74
6	2-MePh	Et_2_O	23	**1f**	73
7	4-MePh	Et_2_O	23	**1g**	60
8	2,6-Me_2_Ph	THF	23	**1h**	50
9	2-MeOPh	THF	–20	**1i**	81
10	4-MeOPh	Et_2_O	0	**1j**	70
11	4-(Me_2_N)Ph	THF	23	**1k**	0 (24)^c^
12	1-naphthyl	THF	0	**1l**	79
13	2-naphthyl	THF	0	**1m**	79
14	2-furyl	THF	0	**1n**	78
15	2-thienyl	THF	23	**1o**	89
16	cinnamyl	Et_2_O	0	**1p**	52
17	*i*-Pr	THF	70	**1q**	0
18	*t*-Bu	THF	70	**1r**	0

a Typical reaction conditions: (1) 1.0 mmol indole, 1.2 mmol aldehyde, 1.5 mmol 2,6-lutidine, 1.4 mmol TMSOTf, solvent (2.5 mL), and temperature as indicated in Table 1, 0.5–16 h; (2) THF (10 mL), 1.1 mmol TBAF, rt, 5 min. See Experimental Section for substrate-specific details.

b Isolated yield after chromatography.

c Product decomposed during purification. Number in parentheses is conversion as determined by ^1^H NMR spectroscopy of the unpurified reaction mixture.

In some cases, isolation of the initial silyl ether products permitted outstanding isolated yields, but some silylated products proved sensitive to purification by column chromatography (Table 2). Benzaldehyde, as well as some electron-poor aromatic aldehydes, provided excellent yields but required purification on a basic or neutral alumina. Unfortunately, no single stationary phase or solvent system appeared to be generally optimal for chromatographic purification of these compounds, and the effectiveness of the alumina showed supplier- and batch-dependence. For some substrates, replacement of the TMS protecting group with a more stable triethylsilyl (TES) group, easily accomplished through the replacement of TMSOTf with TESOTf in the reaction mixture, allowed the isolation of more sensitive compounds, including adducts derived from both the electron-poor 4-nitrobenzaldehdye and the electron-rich 2-furaldehyde (entries 2 and 6). For other seemingly routine substrates, including 4-fluorobenzaldehyde and cinnamaldehyde, no satisfactory combination of conditions could be found for isolation of the silyl ether, despite high conversion to product. Nonetheless, those compounds that proved to be stable to chromatography were recovered with yields in significant excess of those of the corresponding deprotected products.

**Table 2:**
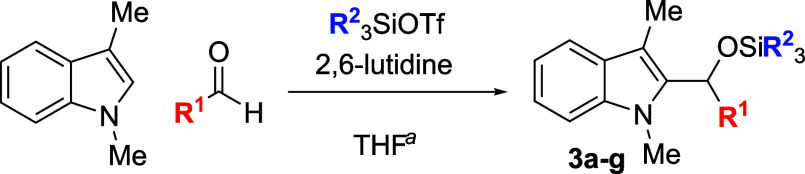
Synthesis and Isolation of 2-Silyoxyalkylation Products

entry	R^1^	R^2^_3_SiOTf	temp (°C)	product	yield (%)^b^
1	Ph	TMS	0	**3a**	89
2	4-(NO_2_)Ph	TES	23	**3b**	94
3	4-FPh	TMS	23	**3c**	0
4	4-ClPh	TMS	23	**3d**	94
5	4-BrPh	TMS	23	**3e**	95
6	2-furyl	TES	0	**3f**	54
7	cinnamyl	TMS	0	**3g**	0

a Typical reaction conditions: (1) 1.0 mmol indole, 1.2 mmol aldehyde, 1.5 mmol 2,6-lutidine, 1.4 mmol R_3_SiOTf; THF (2.5 mL) rt, 0.5–16 h. See Experimental Section for substrate-specific details.

b Isolated yield after chromatography.

The reactivity of various 3-alkylated indoles^27^ toward benzaldehyde was assessed next (Table 3). Indoles bearing primary substituents at C3 reacted with consistently good yields across a range of substitution patterns, including aliphatic (entry 1) and differently substituted benzyl groups (entries 2–8). The benzyl residue provided a convenient handle for evaluating steric and electronic effects of the side chain, with electron-rich, electron-poor, and sterically demanding substituents, all providing satisfactory results. Some limitations were evident with α-branched substrates, however. The cyclohexyl-substituted analogue of 3-methylskatole reacted sluggishly, and multiple unidentified side products were evident in the unpurified reaction mixture, while the more hindered benzhydryl substrate failed to provide products at all (entries 9–10). The reaction also proved sensitive to electronic effects on the indole nucleophile; when the alkyl substituent at C3 was replaced with a strong electron-withdrawing group (e.g., CN or CO_2_Me), no reaction was observed even when the reaction mixture was heated to reflux in high-boiling solvents.

**Table 3:**
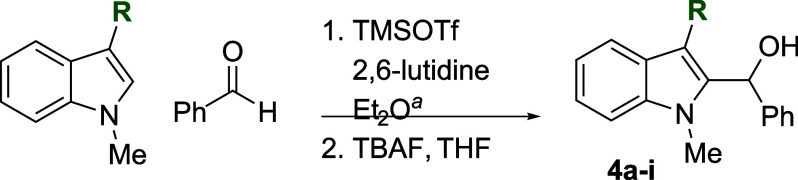
Reaction of Various 3-Alkylated Indoles with Benzaldehyde

entry	R	solvent	temp (°C)	product	yield (%)^b^
1	Et	Et_2_O	23	**4a**	76
2	CH_2_Ph	Et_2_O	23	**4b**	71
3	CH_2_(4-CF_3_)Ph	Et_2_O	23	**4c**	63
4	CH_2_(4-F)Ph	Et_2_O	23	**4d**	67
5	CH_2_(4-Cl)Ph	Et_2_O	23	**4e**	71
6	CH_2_(4-Br)Ph	Et_2_O	23	**4f**	70
7	CH_2_(4-MeO)Ph	Et_2_O	23	**4g**	77
8	CH_2_(2-naphthyl)	Et_2_O	0	**4h**	77
9	Cy	Et_2_O	23	**4i**	48^c^
10	CHPh_2_	Et_2_O	23	**4j**	0
11	CN	Et_2_O	23	**4k**	0
12	CO_2_Me	Et_2_O	23	**4l**	0

a Typical reaction conditions: (1) 1.0 mmol indole, 1.2 mmol aldehyde, 1.5 mmol 2,6-lutidine, 1.4 mmol TMSOTf, Et_2_O (2.5 mL), temperature as indicated in Table 1, 0.5–16 h; (2) THF (10 mL), 1.1 mmol TBAF, rt, 5 min. See Experimental Section for substrate-specific details.

b Isolated yield after chromatography.

c Product contaminated with approximately 4 mol % unidentified side product.

Examination of more labile N-protecting groups on skatole proved rewarding. Both allyl- and benzyl-protected skatoles performed admirably when treated with benzaldehyde, providing conveniently protected adducts in yields that were significantly higher than those observed with the standard *N*-methylskatole substrate (89% and 92% for *N*-allylskatole^28^ and *N*-benzylskatole,^29^ respectively, vs 75% for *N*-methylskatole) (Table 4). The reaction of *N*-benzylskatole with 4-nitrobenzaldehyde provided similarly improved results, but with 4-methoxybenzaldehyde the yield did not significantly differ from that of the *N*-methylskatole case. Unprotected skatole failed to react in high conversion, possibly suffering from the steric effects of observed in situ N-silylation, and BOC-protected indoles proved incompatible with the reaction conditions, readily undergoing deprotection in the presence of TMSOTf. This brief survey suggests that *N*-benzylskatole may be the ideal substrate for the synthesis of 2-hydroxyalkylated indoles in terms of both yield and synthetic versatility.

**Table 4:**
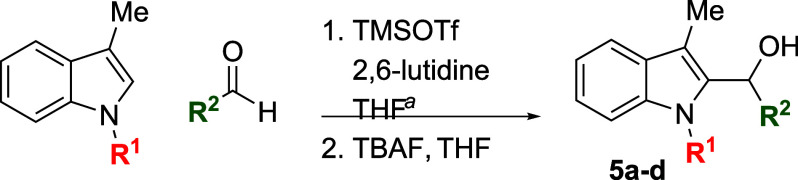
Silyloxyalkylation in the Presence of Various N-Protecting Groups

entry	R^1^	R^2^	temp (°C)	product	yield (%)^b^
1	allyl	Ph	23	**5a**	89
2	Bn	Ph	0	**5b**	92
3	Bn	4-(NO_2_)Ph	23	**5c**	84
4	Bn	4-MeOPh	0	**5d**	69

a Typical reaction conditions: (1) 1.0 mmol indole, 1.2 mmol aldehyde, 1.5 mmol 2,6-lutidine, 1.4 mmol TMSOTf, THF (2.5 mL), temperature as indicated in Table 1, 0.5–16 h; (2) THF (10 mL), 1.1 mmol TBAF, rt, 5 min. See Experimental Section for substrate-specific details.

b Isolated yield after chromatography.

The ability of silyl triflates to suppress triarylmethane formation may depend on multiple factors. First, TMSOTf activation of the aldehyde leads, after attack by the indole, to a stable, covalent silicon–oxygen bond (intermediate **6**, Figure 2). The thermodynamic favorability of Si–O bond formation^30^ may help mitigate the temporary loss of aromaticity in the intermediate, supporting a longer half-life by slowing rearomatization via retroaddition *and* providing an opportunity for deprotonation by the hindered amine base. Perhaps more importantly, the size of the TMS group may protect silyloxyalkylated product **7** from undesired ionization by slowing protonation of the silyl ether oxygen by a Brønsted acid, such as the protonated amine. Lewis acid-catalyzed activation by residual TMSOTf would be similarly deterred, and action by any stronger acids is precluded by the presence of the amine base. The sterically hindered nature of 2,6-lutidine, the base of choice for these reactions, may also play a role by slowing any potential proton transfer from its conjugate acid to the bulky silyl ether.

**Figure 2:**
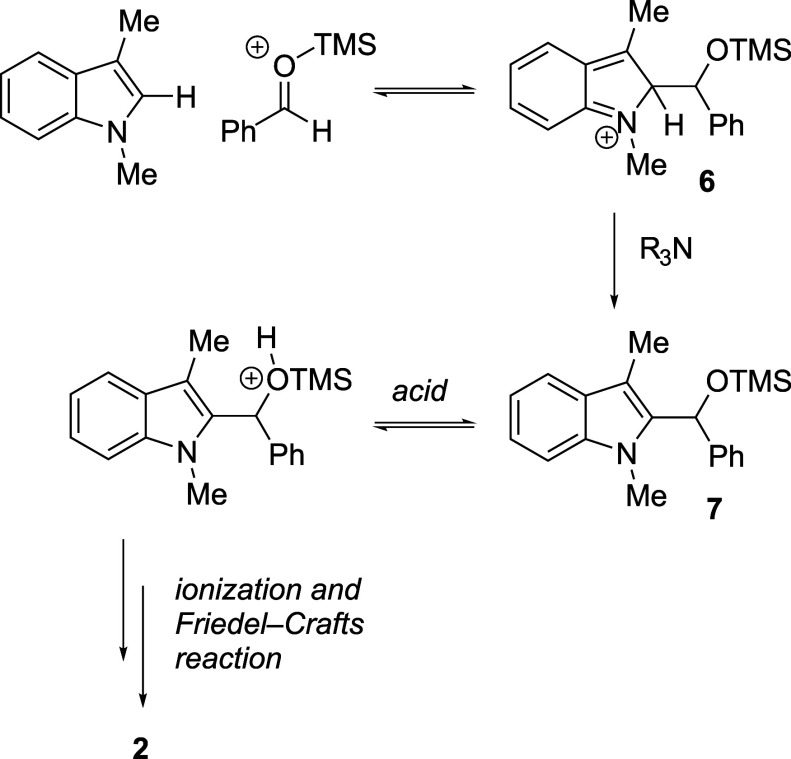
Proposed mechanism and role of the silyl group.

## Conclusion

In summary, the TMSOTf-promoted Friedel–Crafts addition of 3-alkylated indoles to aldehydes provides a reliable synthesis of 2-silyloxyalkylated and 2-hydroxyalkylated indoles. The bulk and thermodynamic stability of the initial silyl ether product may help suppress the generation of triarylmethanes known to form under similar reaction conditions. The reaction is general to a wide range of nonenolizable aldehydes and tolerates multiple substitution patterns at the 3-position of the indole. Benzyl-protected indoles appear to be especially convenient and effective substrates.

## Experimental Section

### General

Reactions were carried out under an atmosphere of nitrogen with a septum cap in oven-dried glassware with magnetic stirring. Tetrahydrofuran (THF), methylene chloride (CH_2_Cl_2_), and diethyl ether (Et_2_O) were purified by passage through a bed of activated alumina.^31^ Trimethylsilyl trifluoromethanesulfonate (TMSOTf) was stored in a Schlenk flask under an inert atmosphere. Certain aldehydes were distilled prior to use and stored in a refrigerator (*o*-anisaldehyde, *p*-anisaldehyde, *o*-tolualdehyde, *p*-tolualdehyde, 2-thiophenecarboxaldehyde, 2-furanaldehyde, *p*-(trifluoromethyl)­benzaldehyde, *p*-fluorobenzaldehyde). All 3-methylskatole was used as received from Ambeed. All other chemicals were used as received or synthesized by literature procedures as noted.. Purification of reaction products was carried out by flash chromatography using silica gel (230–400 mesh). Analytical thin layer chromatography was performed on silica gel plates. Visualization was accomplished with UV light and/or phosphomolybdic acid staining, followed by heating. Infrared spectra were recorded on an FT-IR spectrometer. ^1^H NMR spectra were recorded on a 500 MHz spectrometer, 400 MHz spectrometer, or 300 MHz spectrometer and are reported in ppm using solvent as an internal standard (CDCl_3_ at 7.28 ppm). Data are reported as (ap = apparent, s = singlet, d = doublet, t = triplet, q = quartet, sx = sextet, sp = septet, m = multiplet, b = broad; coupling constant(s) in Hz; integration). Proton-decoupled ^13^C NMR spectra were recorded on a 125 MHz spectrometer or 100 MHz spectrometer, and are reported in ppm using solvent as an internal standard (CDCl_3_ at 77.0 ppm). High-resolution mass spectra were obtained by electrospray ionization (TOF, ion trap), unless otherwise indicated. Melting points were determined by using a capillary melting point apparatus.

### General Procedure

To an oven-dried round-bottomed 10 mL flask under a N_2_ atmosphere were added the indole (1.0 mmol), solvent (2.5 mL), aldehyde (1.0–1.2 mmol), and 2,6-lutidine (175 μL, 161 mg, 1.5 mmol). To this solution TMSOTf (255 μL, 262 mg, 1.4 mmol) was added dropwise by syringe. The reaction was stirred at the indicated temperature for the indicated time, then quenched with pyridine (210 μL, 206 mg, 2.6 mmol), and passed through a column of silica (3 cm × 1 cm) with Et_2_O (20 mL) or a 1:1 mixture of EtOAc:hexane (20 mL). The solvent was removed in vacuo, and the residue was redissolved in THF (10 mL). Tetrabutylammonium fluoride (TBAF) (1.1 mL, 1.0 M in THF, 1.1 mmol) was added dropwise via syringe, and the mixture was stirred for 5 min and then partitioned between ethyl acetate (50 mL) and saturated NaHCO_3_ (50 mL). The layers were separated and the combined aqueous layers were back-extracted with ethyl acetate (50 mL). The combined organic layers were diluted with 100 mL of hexane, then dried over Na_2_SO_4_. The Na_2_SO_4_ was removed by gravity filtration, and the solvents were removed in vacuo. The residue was purified by column chromatography as indicated.

#### (1-Methyl-3-methyl-2-indolyl)­phenylmethanol (**1a**)

The title compound^32^ was prepared according to a variation of the General Procedure with 1,3-dimethylindole (138 μL, 145 mg, 1.0 mmol) and benzaldehyde (120 μL, 127 mg, 1.2 mmol) in THF. The mixture was cooled to 0 °C prior to addition of TMSOTf, the TMSOTf was added dropwise by syringe, and the mixture was stirred for 40 min at 0 °C prior to workup and TBAF deprotection. The product was isolated as an amorphous yellow solid (189 mg, 75% yield) after purification on silica (2–20% ethyl acetate/hexane): IR (solid) 3376, 3058, 3028, 2918, 2884, 2859, 1471, 1449, 1365, 1328, 1183, 999, 878, 737, 711, 697 cm^–1^; ^1^H NMR (500 MHz, CDCl_3_) δ 7.66 (dt, *J* = 7.8, 1.0 Hz, 1H), 7.40–7.35 (m, 4H), 7.34–7.24 (m, 3H), 7.20 (ddd, *J* = 7.9, 6.6, 1.4 Hz, 1H), 6.32 (s, 1H), 3.50 (s, 3H), 2.54 (s, 1H), 2.42 (s, 3H); ^13^C­{^1^H} NMR (126 MHz, CDCl_3_) δ 141.5, 137.6, 135.7, 128.5, 127.9, 127.2, 125.8, 122.2, 119.1, 119.0, 109.6, 109.0, 67.4, 30.9, 8.9; HRMS (ESI, TOF): Exact mass calcd for C_17_H_18_NO [M + H]^+^, 252.1383; found, 252.1395.
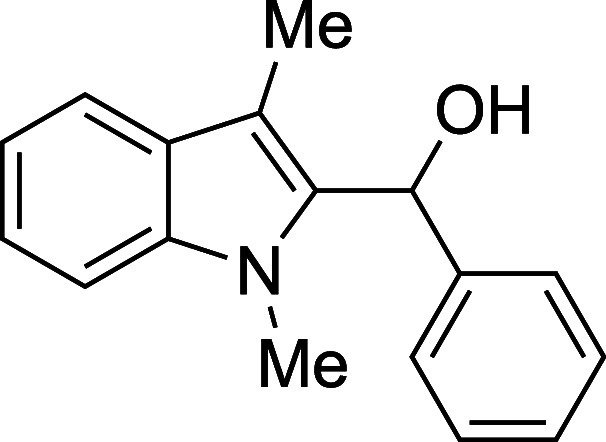


#### (1-Methyl-3-methyl-2-indolyl)­(*p*-nitrophenyl)­methanol (**1b**)

The title compound^33^ was prepared according to a variation of the General Procedure with 1,3-dimethylindole (138 μL, 145 mg, 1.0 mmol) and *p*-nitrobenzaldehyde (151 mg, 1.0 mmol) in THF. After the TMSOTf was added dropwise by syringe, the mixture was stirred for 90 min at ambient temperature prior to workup and TBAF deprotection. The product was isolated as an orange foam (219 mg, 74% yield, corrected for residual solvent) after purification on silica (2–10% ethyl acetate/hexane): IR (solid) 3435, 2921, 1735, 1598, 1517, 1471, 1343, 1239, 1182, 1041, 1013, 851, 823, 789, 739, 719 cm^–1^; ^1^H NMR (500 MHz, CDCl_3_) δ 8.24–8.11 (m, 2H), 7.65 (dt, *J* = 7.9, 1.0 Hz, 1H), 7.59–7.52 (m, 2H), 7.35–7.24 (m, 2H), 7.19 (ddd, *J* = 8.0, 6.7, 1.3 Hz, 1H), 6.40 (s, 1H), 3.48 (s, 3H), 2.92 (s, 1H), 2.45 (s, 3H); ^13^C­{^1^H} NMR (126 MHz, CDCl_3_) δ 149.0, 147.0, 137.6, 134.4, 127.6, 126.7, 123.6, 122.7, 119.3, 119.2, 110.2, 109.0, 66.7, 30.9, 8.9; HRMS (ESI, TOF): Exact mass calcd for C_17_H_15_N_2_O_2_ [M – OH]^+^, 279.1128; found, 279.1124.
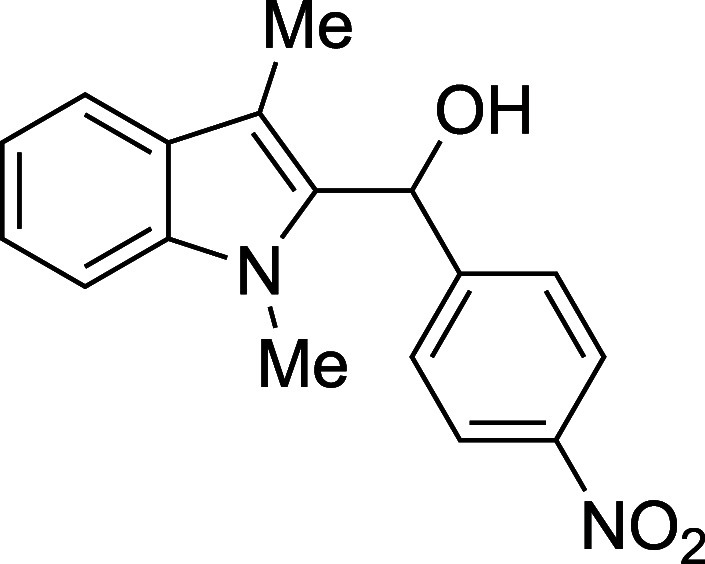


#### (1-Methyl-3-methyl-2-indolyl)­[*p*-(trifluoromethyl)­phenyl]­methanol (**1c**)

The title compound^33^ was prepared according to the General Procedure with 1,3-dimethylindole (138 μL, 145 mg, 1.0 mmol) and *p*-(trifluoromethyl)­benzaldehyde (164 μL, 209 mg, 1.2 mmol) in THF. After the TMSOTf was added dropwise by syringe, and the mixture was stirred for 40 min at ambient temperature prior to workup and TBAF deprotection. The product was isolated as a yellow foam (237 mg, 67% yield) after purification on silica (2–20% ethyl acetate/hexane): IR (solid) 3372, 2922, 1618, 1471, 1412, 1385, 1321, 1162, 1116, 1065, 1015, 868, 821, 739 cm^–1^; ^1^H NMR (500 MHz, CDCl_3_) δ 7.63–7.54 (m, 3H), 7.49–7.43 (m, 2H), 7.24–7.21 (m, 2H), 7.13 (ddd, *J* = 8.0, 5.8, 2.3 Hz, 1H), 6.36 (s, 1H), 3.45 (s, 3H), 2.39 (s, 3H), 2.32 (s, 1H); ^13^C­{^1^H} NMR (126 MHz, CDCl_3_) δ 145.6, 137.6, 134.9, 129.4 (q, *J* = 23.3 Hz), 127.7, 126.2, 125.4 (q, *J* = 3.8 Hz), 124.4 (q, *J* = 271.9 Hz), 122.6, 119.24, 119.17, 109.9, 109.1, 66.8, 30.9, 8.9; HRMS (ESI, TOF): Exact mass calcd for C_18_H_17_NOF_3_ [M + H]^+^, 320.1257; found, 320.1252.
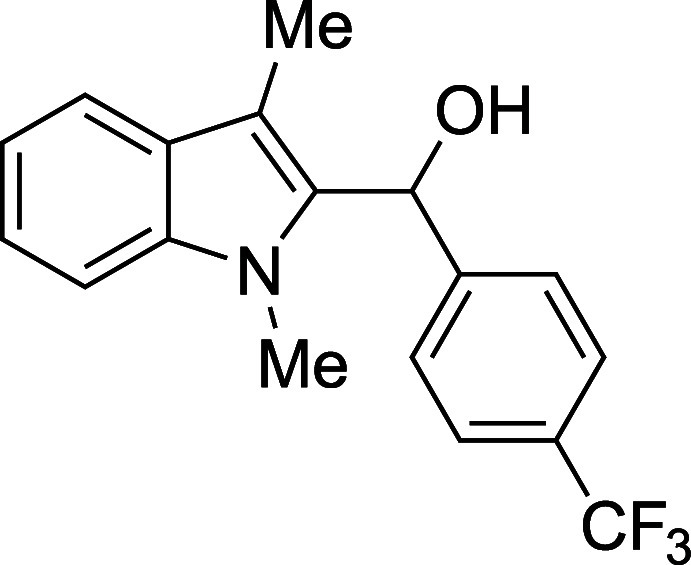


#### (*p*-Fluorophenyl)­(1-methyl-3-methyl-2-indolyl)­methanol (**1d**)

The title compound^33^ was prepared according to the General Procedure with 1,3-dimethylindole (138 μL, 145 mg, 1.0 mmol) and *p*-fluorobenzaldehyde (129 μL, 149 mg, 1.2 mmol) in Et_2_O. After the TMSOTf was added dropwise by syringe, and the mixture was stirred for 90 min at ambient temperature prior to workup and TBAF deprotection. The product was isolated as a yellow foam (199 mg, 74% yield) after purification on silica (2–10% ethyl acetate/hexane): IR (solid) 3382, 3055, 2917, 1602, 1406, 1495, 1324, 1221, 1154, 1117, 1107, 1015, 838, 734, 720, 691 cm^–1^; ^1^H NMR (500 MHz, CDCl_3_) δ 7.63 (dt, *J* = 7.9, 1.0 Hz, 1H), 7.37–7.29 (m, 2H), 7.31–7.24 (m, 2H), 7.16 (ddd, *J* = 7.9, 4.7, 3.3 Hz, 1H), 7.08–6.97 (m, 2H), 6.34 (dq, *J* = 2.7, 1.2 Hz, 1H), 3.51 (s, 3H), 2.41 (s, 3H), 2.33 (d, *J* = 3.1 Hz, 1H); ^13^C­{^1^H} NMR (126 MHz, CDCl_3_) δ 162.0 (d, *J* = 245.4 Hz), 137.5, 137.1 (d, *J* = 3.0 Hz), 135.3, 127.7, 127.5 (d, *J* = 8.0 Hz), 122.3, 119.1, 119.0, 115.2 (d, *J* = 21.3 Hz), 109.7, 108.9, 66.8, 30.9, 8.9; HRMS (ESI, TOF): Exact mass calcd for C_17_H_16_NOF [M]^+^, 269.1210; found, 269.1198.
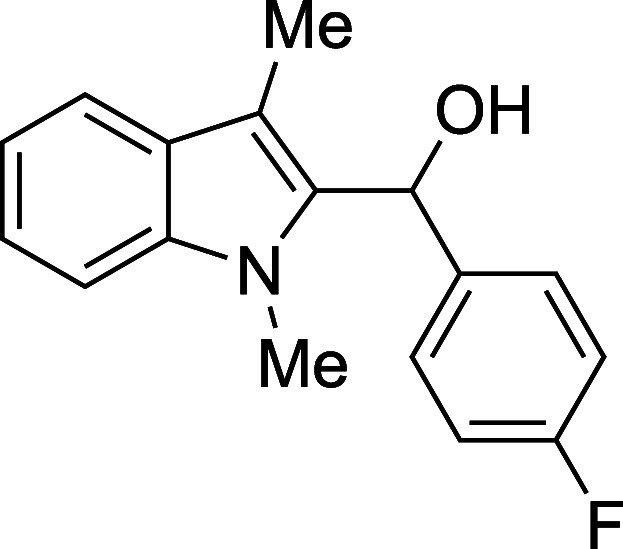


#### (*p*-Bromophenyl)­(1-methyl-3-methyl-2-indolyl)­methanol (**1e**)

The title compound^33^ was prepared according to the General Procedure with 1,3-dimethylindole (138 μL, 145 mg, 1.0 mmol) and *p*-bromobenzaldehyde (185 mg, 1.0 mmol) in THF. After the TMSOTf was added dropwise by syringe, the mixture was stirred for 90 min at ambient temperature prior to workup and TBAF deprotection. The product was isolated as a yellow foam (244 mg, 74% yield) after purification on silica (2–10% ethyl acetate/hexane): IR (solid) 3357, 3053, 2915, 1484, 1470, 1365, 1328, 1240, 1182, 1070, 1008, 858, 807, 734 cm^–1^; ^1^H NMR (500 MHz, CDCl_3_) δ 7.59 (dt, *J* = 7.9, 1.0 Hz, 1H), 7.47–7.39 (m, 2H), 7.25–7.23 (m, 2H), 7.22–7.18 (m, 2H), 7.12 (ddd, *J* = 7.9, 5.3, 2.7 Hz, 1H), 6.29–6.24 (m, 1H), 3.46 (s, 3H), 2.37 (s, 3H), 2.26 (d, *J* = 3.1 Hz, 1H); ^13^C­{^1^H} NMR (126 MHz, CDCl_3_) δ 140.4, 137.6, 135.0, 131.4, 127.7, 127.6, 122.4, 121.1, 119.1, 119.0, 109.9, 109.0, 66.8, 30.9, 8.9; HRMS (ESI, TOF): Exact mass calcd for C_17_H_15_NBr [M – OH]^+^, 312.0382; found, 312.0376.
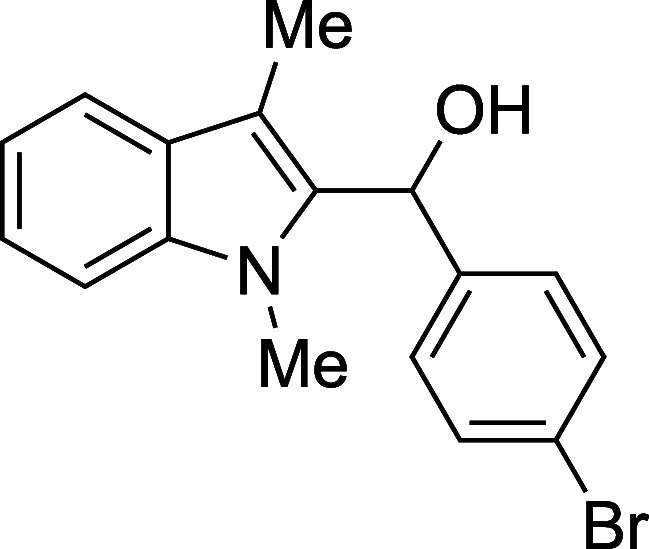


#### (1-Methyl-3-methyl-2-indolyl)­(*o*-tolyl)­methanol (**1f**)

The title compound^33^ was prepared according to the General Procedure with 1,3-dimethylindole (138 μL, 145 mg, 1.0 mmol) and *o*-tolualdehyde (139 μL, 144 mg, 1.2 mmol) in Et_2_O. After the TMSOTf was added dropwise by syringe, and the mixture was stirred overnight at ambient temperature prior to workup and TBAF deprotection. The product was isolated as a pale blue solid (194 mg, 73% yield) after purification on silica (2–10% ethyl acetate/hexane): mp: 107–111 °C; IR (solid) 3157, 2917, 1471, 1365, 1315, 1278, 1180, 1013, 905, 727 cm^–1^; ^1^H NMR (500 MHz, CDCl_3_) δ 7.64 (dt, *J* = 7.3, 1.3 Hz, 1H), 7.55 (dt, *J* = 7.8, 1.0 Hz, 1H), 7.27–7.20 (m, 4H), 7.16–7.12 (m, 1H), 7.10 (ddd, *J* = 7.9, 6.4, 1.5 Hz, 1H), 6.27 (s, 1H), 3.60 (s, 3H), 2.18 (s, 3H), 2.13 (s, 3H), 2.12 (d, *J* = 3.4 Hz, 1H); ^13^C­{^1^H} NMR (126 MHz, CDCl_3_) δ 139.0, 137.2, 135.6, 134.3, 130.7, 127.9, 127.6, 126.2, 125.8, 122.2, 119.0, 118.8, 109.6, 108.9, 66.6, 30.7, 19.4, 8.9; HRMS (ESI, TOF): Exact mass calcd for C_18_H_19_NO [M]^+^, 265.1461; found, 265.1448.
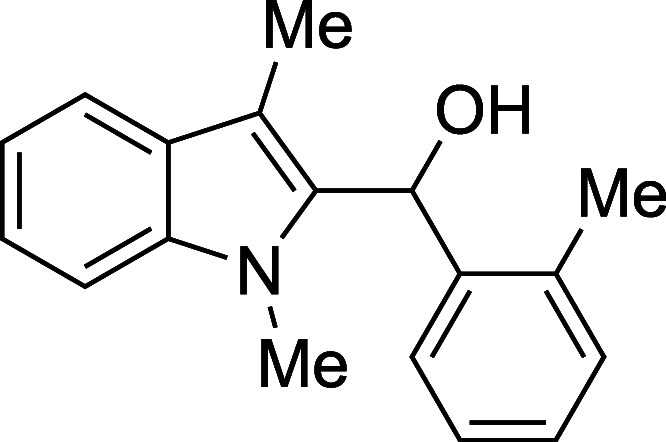


#### (1-Methyl-3-methyl-2-indolyl)­(*p*-tolyl)­methanol (**1g**)

The title compound^33^ was prepared according to the General Procedure with 1,3-dimethylindole (138 μL, 145 mg, 1.0 mmol) and *p*-tolualdehyde (142 μL, 144 mg, 1.2 mmol) in Et_2_O. After the TMSOTf was added dropwise by syringe, the mixture was stirred for 60 min at ambient temperature prior to workup and TBAF deprotection. The product was isolated as a gray solid (159 mg, 60% yield) after purification on silica (2–10% ethyl acetate/hexane): mp: 118–120 °C; IR (solid) 3600, 2916, 1699, 1472, 1364, 1322, 1020, 809, 741, 731 cm^–1^; ^1^H NMR (500 MHz, CDCl_3_) δ 7.63 (dt, *J* = 7.9, 1.0 Hz, 1H), 7.28–7.24 (m, 3H), 7.24–7.22 (m, 1H), 7.19–7.09 (m, 3H), 6.33 (d, *J* = 3.1 Hz, 1H), 3.52 (s, 3H), 2.41 (s, 3H), 2.37 (s, 3H), 2.33 (d, *J* = 3.1 Hz, 1H); ^13^C­{^1^H} NMR (126 MHz, CDCl_3_) δ 138.4, 137.5, 136.8, 135.7, 129.1, 127.8, 125.7, 122.1, 119.0, 118.8, 109.4, 108.9, 67.3, 31.0, 21.1, 8.9; HRMS (ESI, TOF): Exact mass calcd for C_18_H_19_NO [M]^+^, 265.1461; found, 265.1449.
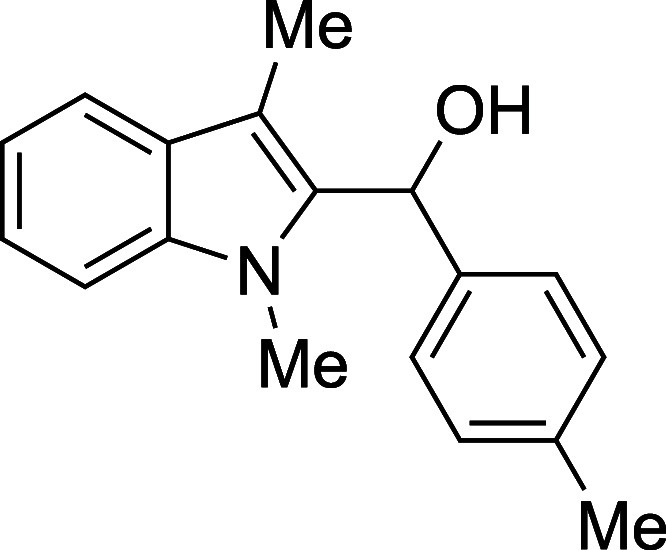


#### (1-Methyl-3-methyl-2-indolyl)­(2,6-xylyl)­methanol (**1h**)

The title compound^33^ was prepared according to a variation of the General Procedure with 1,3-dimethylindole (138 μL, 145 mg, 1.0 mmol) and 2,6-dimethylbenzaldehyde (161 mg, 1.2 mmol) in THF. Amounts of 2,6-lutidine (233 μL, 214 mg, 2.0 mmol) and TMSOTf (344 μL, 422 mg, 1.9 mmol) differed from the General Procedure. After the TMSOTf was added dropwise by syringe, the mixture was stirred for 60 min at ambient temperature prior to workup and TBAF deprotection. The product was isolated as a yellow foam (140 mg, 50% yield) after purification on silica (2–10% ethyl acetate/hexane). *This product begins to decompose upon standing; for reference, three NMR spectra are provided in the* Supporting Information *: one* ^*1*^ *H NMR spectrum taken immediately following purification by chromatography and containing residual solvent, and both a* ^*1*^ *H NMR spectrum and a* ^*13*^ *C NMR spectrum taken after more rigorous removal of residual solvent and showing evidence of some decomposition*: IR (solid) 3388, 2921, 1469, 1356, 1315, 1237, 1181, 1041, 1012, 876, 769, 735 cm^–1^; ^1^H NMR (500 MHz, CDCl_3_) δ 7.54 (dt, *J* = 7.9, 1.0 Hz, 1H), 7.32 (dt, *J* = 8.2, 0.9 Hz, 1H), 7.26 (ddd, *J* = 8.2, 6.9, 1.2 Hz, 1H), 7.20 (dd, *J* = 8.1, 7.0 Hz, 1H), 7.14–7.07 (m, 3H), 6.50 (s, 1H), 3.81 (s, 3H), 2.40 (s, 6H), 2.21–2.17 (bs, 1H), 1.82 (s, 3H); ^13^C­{^1^H} NMR (126 MHz, CDCl_3_) δ 137.4, 137.3, 137.1, 133.2, 129.5, 128.7, 127.9, 122.0, 118.7, 118.7, 109.0, 108.7, 68.4, 30.6, 20.8, 8.3; HRMS (ESI, TOF): Exact mass calcd for C_19_H_20_NO [M – H]^+^, 278.1539; found, 278.1536.
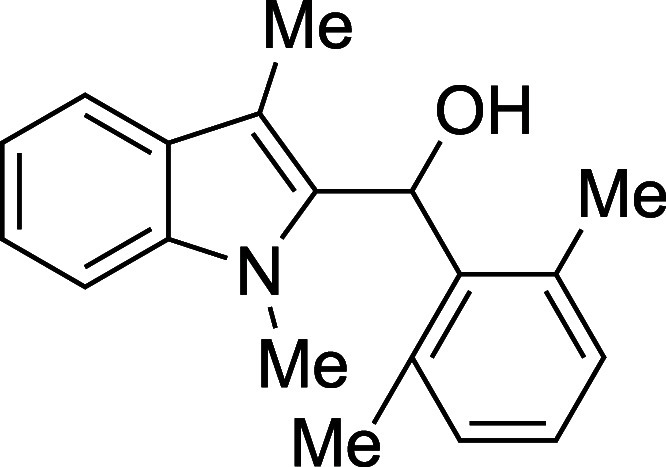


#### (*o*-Methoxyphenyl)­(1-methyl-3-methyl-2-indolyl)­methanol (**1i**)

The title compound^33^ was prepared according to the General Procedure with 1,3-dimethylindole (138 μL, 145 mg, 1.0 mmol) and *o*-anisaldehyde (145 μL, 163 mg, 1.2 mmol) in THF. After cooling the reaction mixture to −20 °C, the TMSOTf was added dropwise by syringe and the mixture was stirred at −20 °C for 9 h prior to workup and TBAF deprotection. The product was isolated as an off-white foam (228 mg, 81% yield) after purification on silica (2–10% ethyl acetate/hexane): IR (solid) 3409, 2918, 1599, 1489, 1463, 1240, 1027, 877, 753, 736 cm^–1^; ^1^H NMR (400 MHz, CDCl_3_) δ 7.56 (dt, *J* = 7.9, 1.0 Hz, 1H), 7.31–7.17 (m, 3H), 7.09 (ddd, *J* = 7.9, 6.9, 1.1 Hz, 1H), 6.94–6.88 (m, 2H), 6.84 (td, *J* = 7.5, 1.1 Hz, 1H), 6.47 (d, *J* = 2.5 Hz, 1H), 3.89 (s, 3H), 3.70 (s, 3H), 3.51 (d, *J* = 2.7 Hz, 1H), 2.28 (s, 3H); ^13^C­{^1^H} NMR (100 MHz, CDCl_3_) δ 157.1, 137.5, 133.9, 129.9, 129.0, 128.2, 127.8, 121.7, 120.7, 118.9, 118.7, 110.5, 109.3, 108.9, 65.8, 55.5, 31.3, 8.9; HRMS (ESI, TOF): Exact mass calcd for C_18_H_20_NO_2_ [M + H]^+^, 282.1489; found, 282.1499.
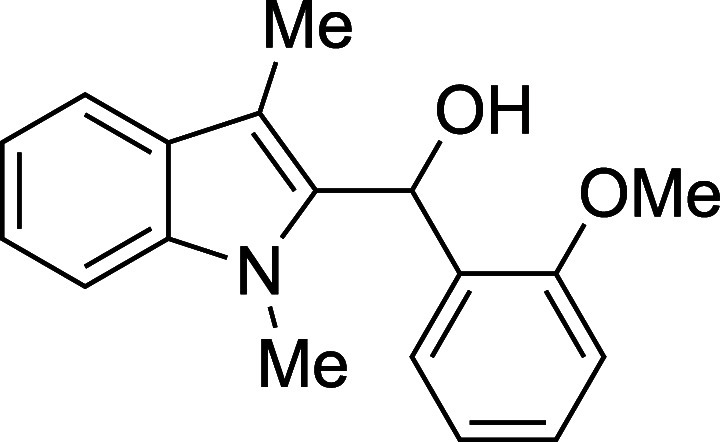


#### (*p*-Methoxyphenyl)­(1-methyl-3-methyl-2-indolyl)­methanol (**1j**)

The title compound^34^ was prepared according to the General Procedure with 1,3-dimethylindole (138 μL, 145 mg, 1.0 mmol) and *p*-anisaldehyde (145 μL, 163 mg, 1.2 mmol) in Et_2_O. The reaction was cooled to 0 °C and TMSOTf was added dropwise by syringe. The mixture was stirred for 30 min at 0 °C prior to workup and TBAF deprotection. The product was isolated as a yellow oil (217 mg, 70% yield) after purification on silica (2–10% ethyl acetate/hexane): IR (solid): 3401, 2931, 2835, 1610, 1508, 1471, 1240, 1168, 1029, 817, 736 cm^–1^; ^1^H NMR (500 MHz, CDCl_3_) δ 7.60 (dt, *J* = 7.8, 1.0 Hz, 1H), 7.27–7.22 (m, 4H), 7.14 (ddd, *J* = 8.0, 5.6, 2.4 Hz, 1H), 6.90–6.82 (m, 2H), 6.33 (d, *J* = 2.9 Hz, 1H), 3.81 (s, 3H), 3.52 (s, 3H), 2.40 (s, 3H), 2.28 (d, *J* = 3.2 Hz, 1H); ^13^C­{^1^H} NMR (125 MHz, CDCl_3_) δ 158.7, 137.6, 135.8, 133.7, 127.9, 127.1, 122.1, 119.0, 118.9, 113.8, 109.3, 108.9, 67.1, 55.3, 30.9, 8.9; HRMS (ESI, TOF): Exact mass calcd for C_18_H_20_NO_2_ [M + H]^+^, 282.1489; found, 282.1484.
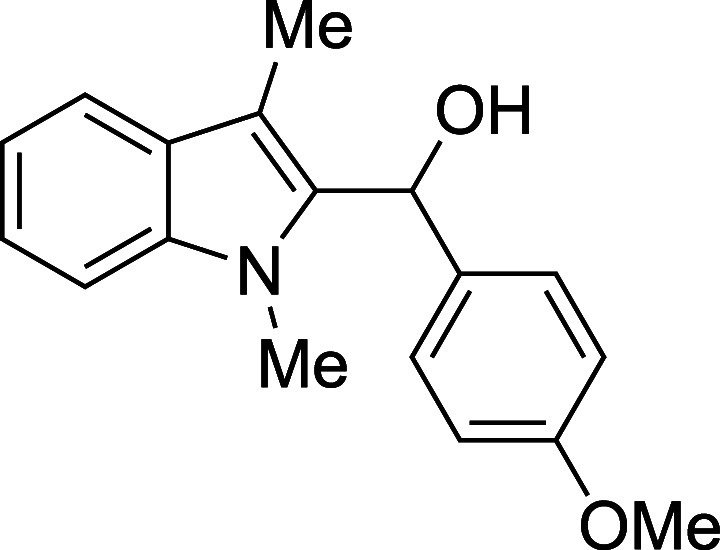


#### (1-Methyl-3-methyl-2-indolyl)­(1-naphthyl)­methanol (**1l**)

The title compound^33^ was prepared according to the General Procedure with 1,3-dimethylindole (138 μL, 145 mg, 1.0 mmol) and 1-naphthaldehyde (163 μL, 187 mg, 1.2 mmol) in THF. After cooling the reaction mixture to 0 °C, TMSOTf was added dropwise by syringe. The mixture was stirred at 0 °C for 90 min prior to workup and TBAF deprotection. The product was isolated as an off-white solid (237 mg, 79% yield) after purification on silica (2–20% ethyl acetate/hexane): mp: 137–145 °C; IR (solid) 3202, 3052, 2908, 2863, 1470, 1354, 1305, 1237, 1186, 1052, 982, 841, 798, 778, 735 cm^–1^; ^1^H NMR (400 MHz, CDCl_3_) δ 8.08–8.00 (m, 1H), 7.93–7.80 (m, 2H), 7.59 (ddt, *J* = 13.9, 7.8, 1.1 Hz, 2H), 7.53–7.40 (m, 3H), 7.31–7.19 (m, 2H), 7.12 (ddd, *J* = 8.0, 6.5, 1.6 Hz, 1H), 6.86 (d, *J* = 3.5 Hz, 1H), 3.65 (s, 3H), 2.31 (bs, 1H), 2.22 (s, 3H); ^13^C­{^1^H} NMR (100 MHz, CDCl_3_) δ 137.4, 136.5, 135.0, 134.0, 130.9, 128.8, 128.7, 128.3, 126.4, 125.8, 125.3, 124.6, 123.7, 122.2, 119.1, 119.0, 109.5, 109.0, 66.7, 30.8, 9.0; HRMS (ESI, TOF): Exact mass calcd for C_21_H_20_NO [M + H]^+^, 302.1539; found, 302.1552.
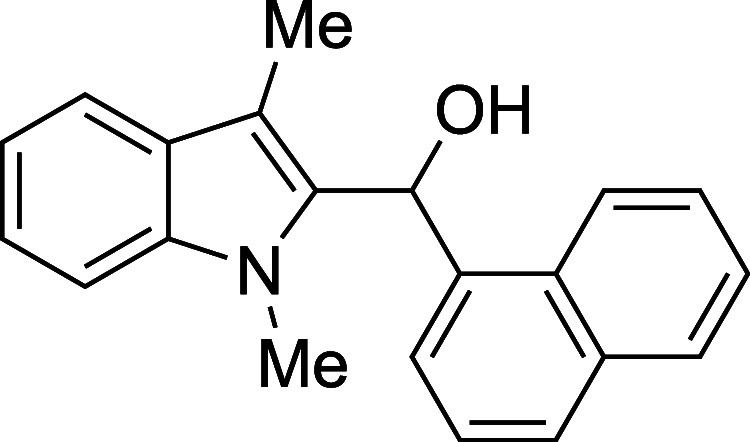


#### (1-Methyl-3-methyl-2-indolyl)­(2-naphthyl)­methanol (**1m**)

The title compound^33^ was prepared according to the General Procedure with 1,3-dimethylindole (138 μL, 145 mg, 1.0 mmol) and 2-naphthaldehyde (187 mg, 1.2 mmol) in THF. The mixture was cooled to 0 °C and TMSOTf was added dropwise by syringe. The mixture was stirred for 90 min at 0 °C prior to workup and TBAF deprotection. The product was isolated as a white foam (238 mg, 79% yield) after purification on silica (2–10% ethyl acetate/hexane): IR (solid) 3385, 3053, 2916, 1471, 1364, 1240, 1190, 1151, 1118, 1014, 853, 807, 736 cm^–1^; ^1^H NMR (500 MHz, CDCl_3_) δ 7.90 (s, 1H), 7.86–7.78 (m, 2H), 7.77 (d, *J* = 8.6 Hz, 1H), 7.63 (dt, *J* = 7.9, 1.0 Hz, 1H), 7.54–7.42 (m, 2H), 7.36 (dd, *J* = 8.7, 2.0 Hz, 1H), 7.31–7.19 (m, 2H), 7.15 (ddd, *J* = 8.0, 5.6, 2.4 Hz, 1H), 6.50 (d, *J* = 3.4, 1H), 3.49 (s, 3H), 2.44 (s, 3H), 2.40 (d, *J* = 3.2 Hz, 1H); ^13^C­{^1^H} NMR (125 MHz, CDCl_3_) δ 138.9, 137.6, 135.4, 133.3, 132.6, 128.15, 128.14, 127.8, 127.7, 126.2, 125.9, 124.2, 124.1, 122.3, 119.1, 118.9, 109.8, 109.0, 67.4, 31.0, 9.0; HRMS (ESI, TOF): Exact mass calcd for C_21_H_20_NO [M + H]^+^, 302.1539; found, 302.1554.
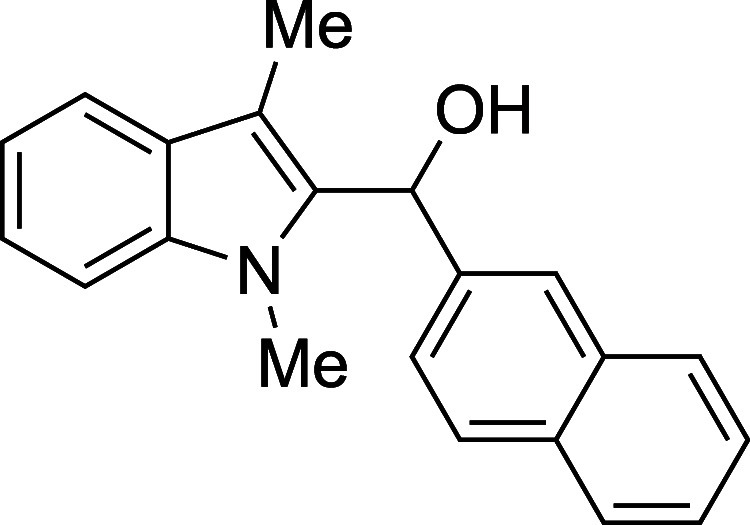


#### (2-Furyl)­(1-methyl-3-methyl-2-indolyl)­methanol (**1n**)

The title compound^33^ was prepared according to the General Procedure with 1,3-dimethylindole (138 μL, 145 mg, 1.0 mmol) and 2-furaldehyde (99 μL, 115 mg, 1.2 mmol) in THF. After the mixture was cooled to 0 °C, TMSOTf was added dropwise by syringe. The mixture was stirred for 60 min at ambient temperature prior to workup and TBAF deprotection. The product was isolated as a brown oil (188 mg, 78% yield) after purification on silica (2–20% ethyl acetate/hexane): IR (solid) 3400, 2918, 1709, 1471, 1363, 1240, 1188, 1142, 999, 733 cm^–1^; ^1^H NMR (500 MHz, CDCl_3_) δ 7.58 (dt, *J* = 8.0, 1.0 Hz, 1H), 7.44–7.39 (m, 1H), 7.33–7.23 (m, 2H), 7.13 (ddd, *J* = 8.0, 6.7, 1.3 Hz, 1H), 6.36 (dd, *J* = 3.2, 1.8 Hz, 1H), 6.28 (s, 1H), 6.15 (dt, *J* = 3.3, 1.1 Hz, 1H), 3.75 (s, 3H), 2.45 (s, 1H), 2.33 (s, 3H); ^13^C­{^1^H} NMR (125 MHz, CDCl_3_) δ 153.8, 142.4, 137.6, 132.8, 127.9, 122.3, 119.1, 118.9, 110.5, 109.8, 109.0, 107.2, 63.3, 31.0, 8.6; HRMS (ESI, TOF): Exact mass calcd for C_15_H_16_NO_2_ [M + H]^+^, 242.1176; found, 242.1175.
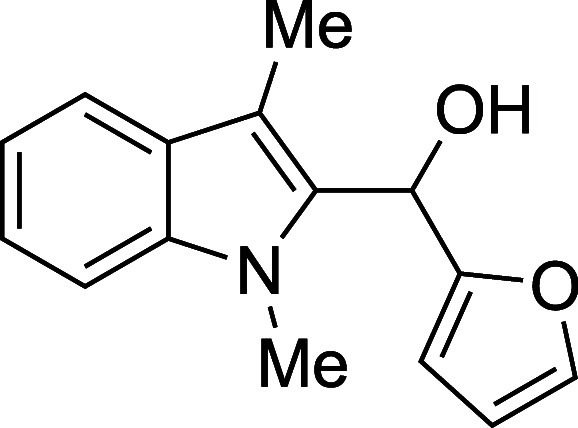


#### (1-Methyl-3-methyl-2-indolyl)­(2-thienyl)­methanol (**1o**)

The title compound^33^ was prepared according to the General Procedure with 1,3-dimethylindole (138 μL, 145 mg, 1.0 mmol) and 2-thiophenecarboxaldehyde (112 μL, 134 mg, 1.2 mmol) in THF. After the TMSOTf was added dropwise by syringe, the mixture was stirred for 90 min at ambient temperature prior to workup and TBAF deprotection. The product was isolated as a green foam (229 mg, 89% yield) after purification on silica (2–10% ethyl acetate/hexane): IR (solid) 3392, 2106, 3083, 2916, 1471, 1382, 1329, 1227, 1193, 1139, 978, 838, 743, 699 cm^–1^; ^1^H NMR (500 MHz, CDCl_3_) δ 7.61 (dt, *J* = 7.9, 1.1 Hz, 1H), 7.33–7.23 (m, 3H), 7.15 (ddd, *J* = 7.9, 5.8, 2.2 Hz, 1H), 6.95 (dd, *J* = 5.1, 3.6 Hz, 1H), 6.73 (dt, *J* = 3.6, 1.3 Hz, 1H), 6.50 (s, 1H), 3.64 (s, 3H), 2.51 (d, *J* = 2.9 Hz, 1H), 2.39 (s, 3H); ^13^C­{^1^H} NMR (125 MHz, CDCl_3_) δ 145.9, 137.7, 134.7, 127.8, 126.9, 125.2, 124.2, 122.4, 119.2, 119.0, 109.4, 109.0, 65.4, 31.1, 8.8; HRMS (ESI, TOF): Exact mass calcd for C_15_H_16_NOS [M + H]^+^, 258.0947; found, 258.0946.
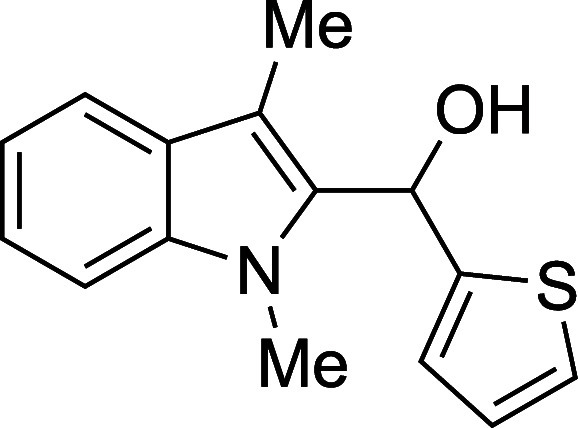


#### (*E*)-1-(1-Methyl-3-methyl-2-indolyl)-3-phenyl-2-propen-1-ol (**1p**)

The title compound^33^ was prepared according to the General Procedure with 1,3-dimethylindole (138 μL, 145 mg, 1.0 mmol) and *trans*-cinnamaldehyde (151 μL, 159 mg, 1.2 mmol) in Et_2_O. The mixture was cooled to 0 °C and the TMSOTf was added dropwise by syringe. The mixture was stirred for 90 min at 0 °C prior to workup and TBAF deprotection. The product was isolated as a yellow foam (158 mg, 52% yield) after purification on silica (2–20% ethyl acetate/hexane): IR (solid) 3355, 3026, 2918, 2861, 1471, 1364, 1328, 1188, 1067, 966, 908, 883, 737, 692 cm^–1^; ^1^H NMR (500 MHz, CDCl_3_) δ 7.57 (dt, *J* = 7.9, 1.0 Hz, 1H), 7.40–7.34 (m, 2H), 7.35–7.20 (m, 5H), 7.12 (ddd, *J* = 7.9, 6.6, 1.4 Hz, 1H), 6.59 (dd, *J* = 16.0, 1.7 Hz, 1H), 6.50 (dd, *J* = 16.0, 4.3 Hz, 1H), 5.86 (dd, *J* = 4.3, 1.8 Hz, 1H), 3.78 (s, 3H), 2.37 (s, 3H), 2.25 (bs, 1H); ^13^C­{^1^H} NMR (125 MHz, CDCl_3_) δ 137.5, 136.5, 134.7, 130.3, 129.4, 128.7, 127.9, 127.8, 126.6, 122.2k, 119.0, 118.9, 109.0, 108.9, 67.0, 31.1, 8.9; HRMS (ESI, TOF): Exact mass calcd for C_19_H_20_NO [M + H]^+^, 278.1539; found, 278.1532.
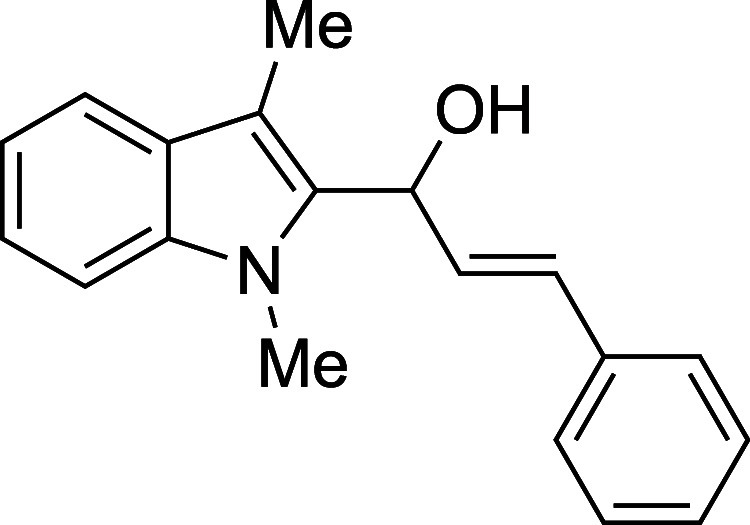


#### Trimethyl­[(1-methyl-3-methyl-2-indolyl)­phenylmethoxy]­silane (**3a**)

To an oven-dried round-bottomed 10 mL flask under a N_2_ atmosphere were added 1,3-dimethylindole (138 μL, 145 mg, 1.0 mmol), THF (2.5 mL), benzaldehyde (112 μL, 117 mg, 1.1 mmol), and 2,6-lutidine (175 mL, 161 mg, 1.5 mmol). After cooling the mixture of 0 °C, TMSOTf (253 mL, 311 mg, 1.4 mmol) was added dropwise by syringe. The reaction mixture was stirred at 0 °C for 60 min, then quenched with pyridine (210 mL, 206 mg, 2.6 mmol), and passed through a column of silica (3 cm × 1 cm) with Et_2_O (20 mL). The solvent was removed in vacuo and the title compound^33^ was isolated as a white powder (286 mg, 89% yield) after purification on neutral alumina (100% hexane): mp: 105–110 °C; IR (solid) 3052, 3027, 2963, 1472, 1385, 1367, 1327, 1290, 1243, 1185, 1082, 1062, 1048, 872, 860, 840, 752, 737, 713, 697 cm^–1^; ^1^H NMR (500 MHz, CDCl_3_) δ 7.58 (dd, *J* = 7.8, 1.1 Hz, 1H), 7.32–7.22 (m, 4H), 7.22–7.14 (m, 3H), 7.09 (ddd, *J* = 7.9, 4.8, 3.1 Hz, 1H), 6.26 (s, 1H), 3.41 (s, 3H), 2.44 (s, 3H), 0.08 (s, 9H); ^13^C­{^1^H} NMR (125 MHz, CDCl_3_) δ 143.0, 137.6, 136.2, 128.3, 128.0, 127.0, 125.8, 121.9, 119.1, 118.8, 108.9, 108.5, 67.7, 31.0, 9.2, 0.0; HRMS (ESI, TOF): Exact mass calcd for C_17_H_16_N [M–OSiMe_3_]^+^, 234.1277; found, 234.1286.
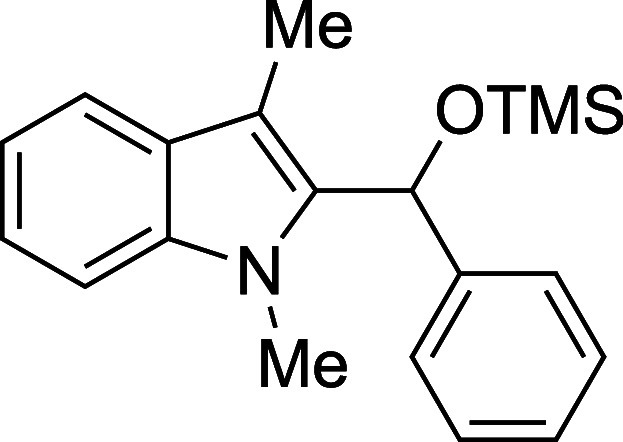


#### Triethyl­[(1-methyl-3-methyl-2-indolyl)­(*p*-nitrophenyl)­methoxy]­silane (**3b**)

To an oven-dried round-bottomed 10 mL flask under a N_2_ atmosphere were added 1,3-dimethylindole (138 μL, 145 mg, 1.0 mmol), THF (2.5 mL), *p-*nitrobenzaldehyde (181 mg, 1.2 mmol), and 2,6-lutidine (175 mL, 161 mg, 1.5 mmol). After the TESOTf (317 mL, 370 mg, 1.4 mmol) was added dropwise by syringe, the reaction mixture was stirred at ambient temperature for 90 min. The reaction was quenched with pyridine (210 mL, 206 mg, 2.6 mmol) and passed through a column of silica (3 x 1 cm) with Et_2_O (20 mL). The solvent was removed in vacuo and the title compound^33^ was isolated as yellow solid (370 mg, 94% yield) after purification on neutral alumina (0–1% ethyl acetate/hexane): mp: 117–119 °C; IR (solid) 3052, 2958, 1875, 1512, 1472, 1343, 1185, 1080, 1007, 853, 836, 735, 719, 692 cm^1^; ^1^H NMR (500 MHz, CDCl_3_) δ 8.21–8.11 (m, 2H), 7.63 (dt, *J* = 7.8, 1.1 Hz, 1H), 7.56–7.47 (m, 2H), 7.28–7.19 (m, 2H), 7.15 (ddd, *J* = 7.9, 6.5, 1.4 Hz, 1H), 6.30 (s, 1H), 3.43 (s, 3H), 2.49 (s, 3H), 0.91 (t, *J* = 7.9 Hz, 9H), 0.64 (qd, *J* = 7.9, 5.1 Hz, 6H); ^13^C­{^1^H} NMR (125 MHz, CDCl_3_) δ 150.6, 147.0, 137.5, 135.0, 127.7, 126.6, 123.5, 122.3, 119.2, 118.9, 108.90, 108.87, 67.3, 30.8, 9.1, 6.7, 4.7; HRMS (ESI, TOF): Exact mass calcd for C_17_H_15_N_2_O_2_ [M–OSiEt_3_]^+^, 279.1128; found, 279.1141.
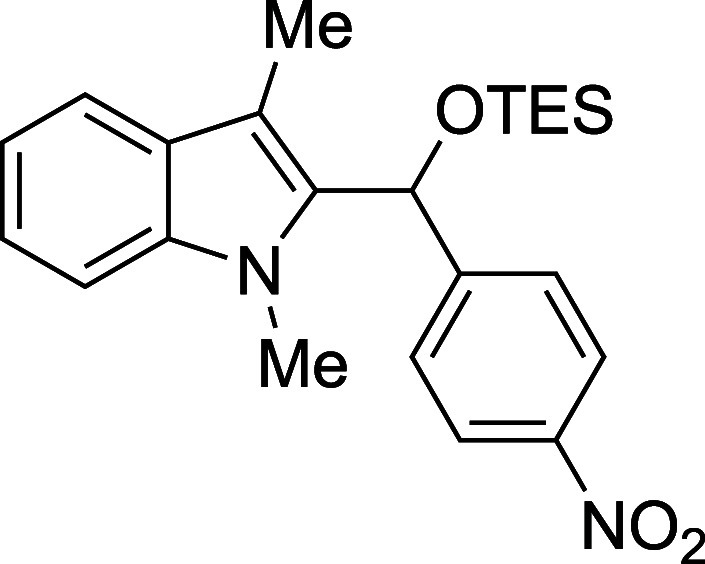


#### [(*p*-Chlorophenyl)­(1-methyl-3-methyl-2-indolyl)­methoxy]­tris­(methyl)­silane (**3d**)

To an oven-dried round-bottomed 10 mL flask under a N_2_ atmosphere were added 1,3-dimethylindole (138 μL, 145 mg, 1.0 mmol), *p*-chlorobenzaldehyde (169 mg, 1.2 mmol), THF (2.5 mL), and 2,6-lutidine (175 mL, 161 mg, 1.5 mmol). After the TMSOTf (253 mL, 311 mg, 1.4 mmol) was added dropwise by syringe, the reaction mixture was stirred at ambient temperature for 90 min, then quenched with pyridine (210 mL, 206 mg, 2.6 mmol) and passed through a column of silica (3 cm × 1 cm) with Et_2_O (20 mL). The solvent was removed in vacuo and the title compound^33^ was isolated as a green oil (335 mg, 94% yield) after purification on basic alumina (2–20% ethyl acetate/hexane): IR (neat) 3056, 2955, 2835, 1669, 1594, 1487, 1471, 1405, 1366, 1250, 1184, 1109, 1048, 1013, 887, 869, 840, 811, 738 cm^–1^; ^1^H NMR (500 MHz, CDCl_3_) δ 7.63 (dt, *J* = 7.9, 1.0 Hz, 1H), 7.30–7.20 (m, 6H), 7.18–7.10 (m, 1H), 6.24 (s, 1H), 3.44 (s, 3H), 2.47 (s, 3H), 0.11 (s, 9H); ^13^C­{^1^H} NMR (125 MHz, CDCl_3_) δ 141.5, 137.5, 135.5, 132.6, 128.3, 127.8, 127.2, 122.0, 119.1, 118.8, 108.8, 108.6, 67.2, 30.9, 9.0, −0.1; HRMS (ESI, TOF): Exact mass calcd for C_17_H_15_NCl [M–OSiMe_3_]^+^, 268.0888; found, 268.0882.
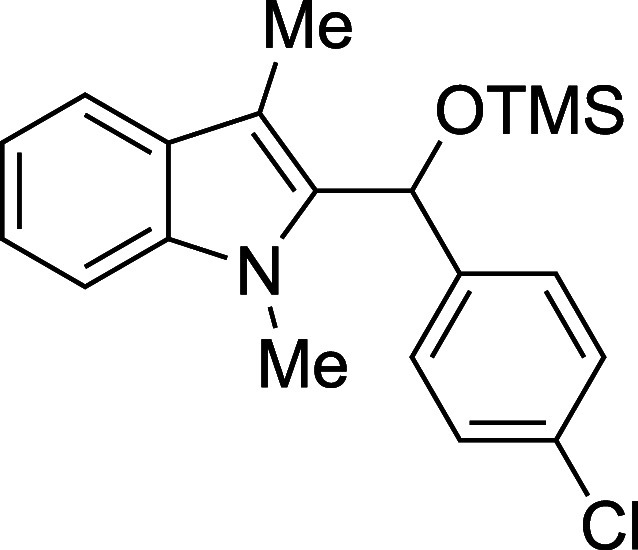


#### [(*p*-Bromophenyl)­(1-methyl-3-methyl-2-indolyl)­methoxy]­tris­(methyl)­silane (**3e**)

To an oven-dried round-bottomed 10 mL flask under a N_2_ atmosphere were added 1,3-dimethylindole (138 μL, 145 mg, 1.0 mmol), *p*-bromobenzaldehyde (185 mg, 1.0 mmol), THF (2.5 mL), and 2,6-lutidine (175 mL, 161 mg, 1.5 mmol). After the TMSOTf (253 mL, 311 mg, 1.4 mmol) was added dropwise by syringe, the reaction mixture was stirred at ambient temperature for 90 min, then quenched with pyridine (210 mL, 206 mg, 2.6 mmol) and passed through a column of silica (3 cm × 1 cm) with Et_2_O (20 mL). The solvent was removed in vacuo and the title compound^33^ was isolated as a yellow oil (383 mg, 95% yield) after purification on neutral alumina (100% hexane): IR (neat) 3055, 2954, 2855, 1484, 1471, 1366, 1250, 1184, 1068, 1046, 1009, 885, 868, 838, 808, 736 cm^–1^; ^1^H NMR (500 MHz, CDCl_3_) δ 7.58 (dd, *J* = 8.0, 1.1 Hz, 1H), 7.42–7.33 (m, 2H), 7.24–7.16 (m, 2H), 7.18–7.11 (m, 2H), 7.10 (ddd, *J* = 7.9, 5.4, 2.6 Hz, 1H), 6.18 (s, 1H), 3.40 (s, 3H), 2.43 (s, 3H), 0.08 (s, 9H); ^13^C­{^1^H} NMR (125 MHz, CDCl_3_) δ 142.0, 137.6, 135.5, 131.3, 127.8, 127.6, 122.1, 120.8, 119.1, 118.8, 108.9, 108.7, 67.2, 30.9, 9.1, −0.1; HRMS (ESI, TOF): Exact mass calcd for C_17_H_15_NBr [M–OSiMe_3_]^+^, 312.0382; found, 312.0374.
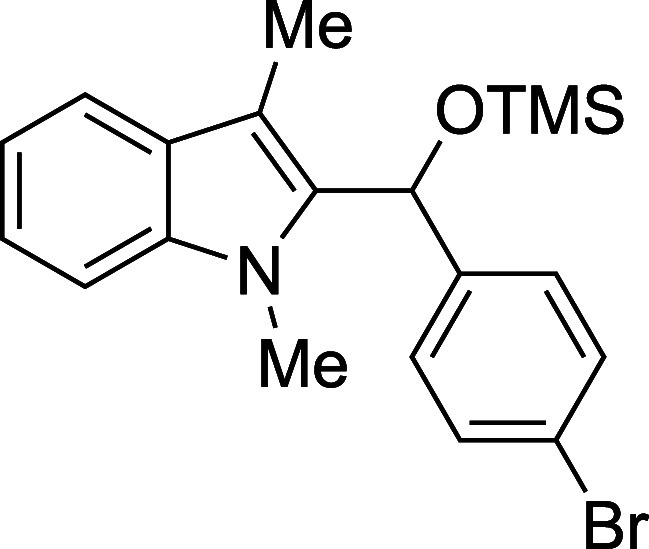


#### Triethyl­[(2-furyl)­(1-methyl-3-methyl-2-indolyl)­methoxy]­silane (**3f**)

To an oven-dried round-bottomed 10 mL flask under a N_2_ atmosphere were added 1,3-dimethylindole (138 μL, 145 mg, 1.0 mmol), THF (2.5 mL), 2-furaldehyde (99 μL, 115 mg, 1.0 mmol), and 2,6-lutidine (175 mL, 161 mg, 1.5 mmol). After the mixture was cooled to 0 °C, the TESOTf (317 mL, 370 mg, 1.4 mmol) was added dropwise by syringe and the reaction mixture was stirred at 0 °C for 60 min. The reaction was quenched with pyridine (210 mL, 206 mg, 2.6 mmol) and passed through a column of silica (3 cm × 1 cm) with Et_2_O (20 mL). The solvent was removed in vacuo and the title compound^33^ was isolated as a green oil (190 mg, 54% yield) after purification on neutral alumina (2–5% ethyl acetate/hexane): IR (neat) 2953, 2912, 2876, 1471, 1364, 1244, 1190, 1143, 1067, 1044, 1002, 850, 812, 732 cm^–1^; ^1^H NMR (500 MHz, CDCl_3_) δ 7.66 (dt, *J* = 8.0, 1.0 Hz, 1H), 7.43–7.41 (m, 1H), 7.36 (dt, *J* = 8.2, 1.0 Hz, 1H), 7.31 (ddd, *J* = 8.2, 6.8, 1.2 Hz, 1H), 7.20 (ddd, *J* = 7.9, 6.8, 1.1 Hz, 1H), 6.38 (dd, *J* = 3.3, 1.8 Hz, 1H), 6.31 (d, *J* = 1.1 Hz, 1H), 6.22 (dt, *J* = 3.3, 1.1 Hz, 1H), 3.85 (s, 3H), 2.47 (s, 3H), 1.01 (t, *J* = 8.0 Hz, 9H), 0.73 (qd, *J* = 7.9, 4.6 Hz, 6H); ^13^C­{^1^H} NMR (125 MHz, CDCl_3_) δ 155.3, 142.1, 137.6, 134.1, 127.9, 121.8, 119.0, 118.7, 110.2, 108.8, 108.3, 106.6, 63.7, 31.1, 8.8, 6.7, 4.8; HRMS (ESI, TOF): Exact mass calcd for C_21_H_30_NO_2_Si [M + H]^+^, 356.2040; found, 356.2033.
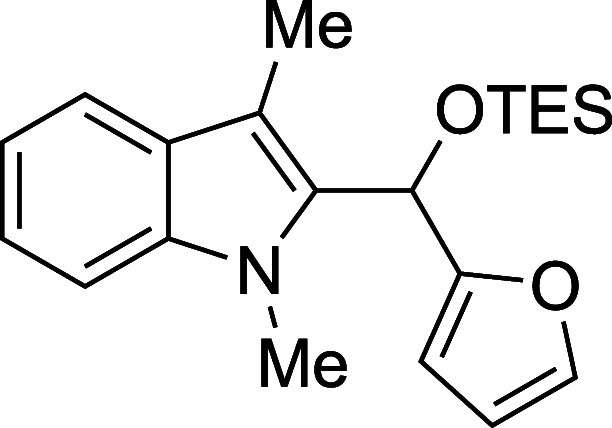


#### (3-Ethyl-1-methyl-2-indolyl)­phenylmethanol (**4a**)

The title compound^33^ was prepared according to a variation of the General Procedure with 3-ethyl-1-methylindole^35^ (159 mg, 1.0 mmol) and benzaldehyde (112 mL, 117 mg, 1.1 mmol) in Et_2_O. Amounts of 2,6-lutidine (163 μL, 150 mg, 1.4 mmol) and TMSOTf (272 μL, 334 mg, 1.5 mmol) differed from the General Procedure. After the TMSOTf was added dropwise by syringe, the mixture was stirred for 1.5 h at ambient temperature prior to workup and TBAF deprotection. The product was isolated as a yellow oil (200 mg, 76% yield) after purification on silica (2–5% ethyl acetate/hexane): IR (neat) 3390, 3056, 2961, 2928, 2869, 1471, 1449, 1366, 1333, 1182, 1013, 907, 888, 738, 712 cm^–1^; ^1^H NMR (500 MHz, CDCl_3_) δ 7.69 (dt, *J* = 7.9, 1.0 Hz, 1H), 7.42–7.33 (m, 4H), 7.34–7.25 (m, 3H), 7.17 (ddd, *J* = 8.0, 5.0, 3.0 Hz, 1H), 6.38 (d, *J* = 2.7 Hz, 1H), 3.49 (s, 3H), 3.02–2.84 (m, 2H), 2.31 (d, *J* = 2.9 Hz, 1H), 1.34 (t, *J* = 7.6 Hz, 3H); ^13^C­{^1^H} NMR (125 MHz, CDCl_3_) δ 141.4, 137.7, 134.9, 128.4, 127.1, 126.8, 125.8, 122.2, 119.2, 118.9, 116.9, 109.0, 67.1, 30.9, 17.6, 16.8; HRMS (ESI, TOF): Exact mass calcd for C_18_H_18_N [M–OH]^+^, 248.1434; found, 248.1441.
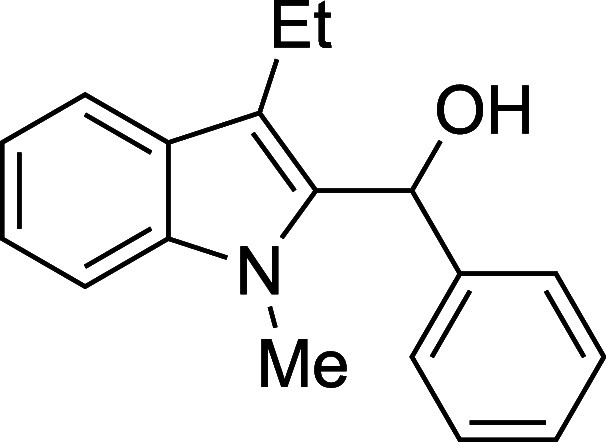


#### (3-Benzyl-1-methyl-2-indolyl)­phenylmethanol (**4b**)

The title compound^33^ was prepared according to a variation of the General Procedure with 3-benzyl-1-methylindole^36^ (221 mg, 1.0 mmol) and benzaldehyde (102 μL, 106 mg, 1.0 mmol) in Et_2_O. Amounts of 2,6-lutidine (163 μL, 150 mg, 1.4 mmol) and TMSOTf (272 μL, 333 mg, 1.5 mmol) differed from the General Procedure. After the TMSOTf was added dropwise by syringe, and the mixture was stirred overnight at ambient temperature prior to workup and TBAF deprotection. The product was isolated as an orange oil (233 mg, 71% yield, corrected for trace solvent) after purification on silica gel (1–20% ethyl acetate/hexane): IR (neat) 3518, 3062, 3028, 2912, 2851, 1492, 1470, 1446, 1369, 1324, 1173, 1015, 876, 750, 721, 710, 694 cm^–1^; ^1^H NMR (500 MHz, CDCl_3_) δ 7.62 (d, *J* = 7.9 Hz, 1H), 7.33–7.20 (m, 11H), 7.18–7.12 (m, 2H), 6.27 (s, 1H), 4.24 (d, *J* = 16.2 Hz, 1H), 4.20 (d, *J* = 16.2 Hz, 1H), 3.43 (s, 3H), 2.50 (s, 1H); ^13^C­{^1^H} NMR (125 MHz, CDCl_3_) δ 142.1, 141.4, 137.8, 136.6, 128.7, 128.49, 128.45, 127.6, 127.3, 126.1, 125.9, 122.4, 119.7, 119.5, 113.2, 109.3, 67.4, 31.1, 30.3; HRMS (ESI, TOF): Exact mass calcd for C_23_H_21_NO [M]^+^, 327.1623; found, 327.1617.
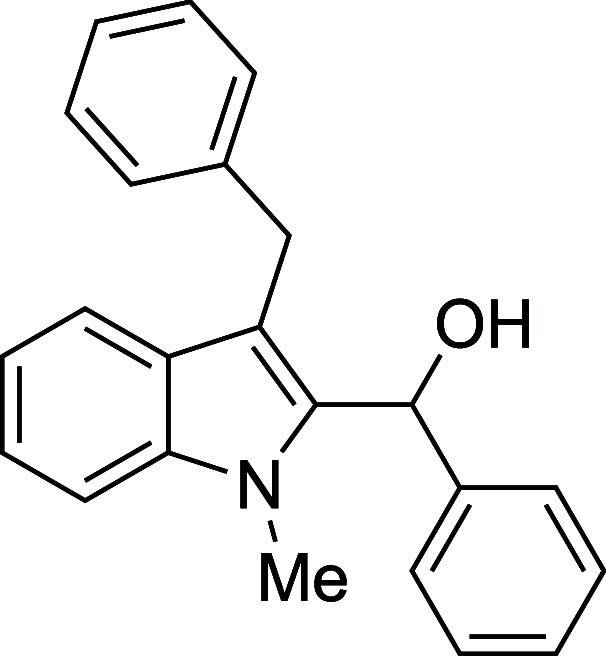


#### (1-Methyl-3-{[*p*-(trifluoromethyl)­phenyl]­methyl}-2-indolyl)­phenylmethanol (**4c**)

The title compound^33^ was prepared according to a variation of the General Procedure with 1-methyl-3-{[*p*-(trifluoromethyl)­phenyl]­methyl}­indole^36^ (289 mg, 1.0 mmol) and benzaldehyde (122 μL, 127 mg, 1.2 mmol) in Et_2_O. Amounts of 2,6-lutidine (186 μL, 171 mg, 1.6 mmol) and TMSOTf (308 μL, 378 mg, 1.7 mmol) differed from the General Procedure. After the TMSOTf was added dropwise by syringe, and the mixture was stirred overnight at ambient temperature prior to workup and TBAF deprotection. The product was isolated as a pink foam (248 mg, 63% yield) after purification on silica (2–10% ethyl acetate/hexane): IR (solid) 3412, 3058, 2936, 1615, 1473, 1367, 1321, 1161, 1106, 1065, 1017, 739, 712 cm^–1^; ^1^H NMR (500 MHz, CDCl_3_) δ 7.55 (dt, *J* = 7.9, 1.0 Hz, 1H), 7.52 (d, *J* = 8.1 Hz, 2H), 7.37 (d, *J* = 8.0 Hz, 2H), 7.36–7.25 (m, 7H), 7.16 (ddd, *J* = 8.0, 6.5, 1.5 Hz, 1H), 6.36 (s, 1H), 4.35 (d, *J* = 16.5 Hz, 2H), 4.30 (d, *J* = 16.6 Hz, 2H), 3.56 (s, 3H), 2.34 (s, 1H); ^13^C­{^1^H} NMR (125 MHz, CDCl_3_) δ 145.9, 141.0, 137.7, 136.6, 128.53, 128.45, 128.3 (q, *J* = 32.2 Hz), 127.4, 127.3, 125.7, 125.4 (q, *J* = 3.8 Hz), 124.2 (q, *J* = 272.0 Hz), 122.5, 119.5, 119.2, 111.9, 109.2, 67.5, 31.0, 30.1; HRMS (ESI, TOF): Exact mass calcd for C_24_H_19_NOF_3_ [M–H]^+^, 394.1413; found, 394.1422.
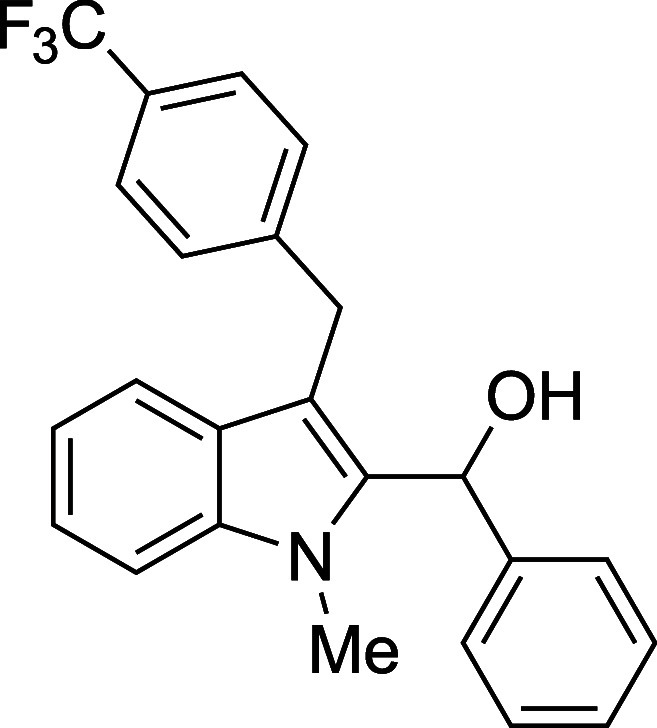


#### {3-[(*p*-Fluorophenyl)­methyl]-1-methyl-2-indolyl}­phenylmethanol (**4d**)

The title compound^33^ was prepared according to a variation of the General Procedure with 3-[(*p*-fluorophenyl)­methyl]-1-methylindole^36^ (239 mg, 1.0 mmol) and benzaldehyde (112 μL, 117 mg, 1.1 mmol) in Et_2_O. Amounts of 2,6-lutidine (163 μL, 150 mg, 1.4 mmol) and TMSOTf (272 μL, 334 mg, 1.5 mmol) differed from the General Procedure. After the TMSOTf was added dropwise by syringe, and the mixture was stirred overnight at ambient temperature prior to workup and TBAF deprotection. The product was isolated as a pink foam (230 mg, 67% yield) after purification on silica (2–5% ethyl acetate/hexane): IR (solid) 3418, 3044, 3029, 2905, 1709, 1601, 1506, 1471, 1449, 1366, 1217, 1155, 1014, 822, 737, 711, 699 cm^–1^; ^1^H NMR (500 MHz, CDCl_3_) δ 7.58 (dt, *J* = 7.9, 1.0 Hz, 1H), 7.40–7.26 (m, 7H), 7.26–7.18 (m, 2H), 7.16 (ddd, *J* = 8.0, 6.3, 1.8 Hz, 1H), 7.00–6.92 (m, 2H), 6.36 (s, 1H), 4.24 (d, *J* = 16.4 Hz, 1H), 4.20 (d, *J* = 16.4 Hz, 1H), 3.54 (s, 3H), 2.29 (s, 1H); ^13^C­{^1^H} NMR (125 MHz, CDCl_3_) δ 161.3 (d, *J* = 243.36 Hz), 141.1, 137.7, 137.4 (d, *J* = 3.1 Hz), 136.4, 129.6 (d, *J* = 7.8 Hz), 128.4, 127.33, 127.28, 125.8, 122.4, 119.39, 119.36, 115.2 (d, *J* = 21.2 Hz), 112.9, 109.2, 67.4, 31.0, 29.4; HRMS (ESI, TOF): Exact mass calcd for C_23_H_19_NF [M–OH]^+^, 328.1496; found, 328.1498.
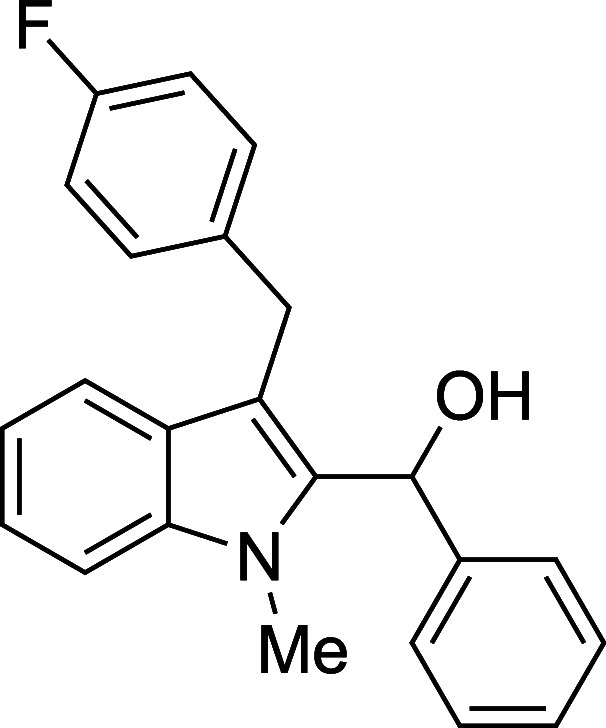


#### {3-[(*p*-Chlorophenyl)­methyl]-1-methyl-2-indolyl}­phenylmethanol (**4e**)

The title compound^33^ was prepared according to a variation of the General Procedure with 3-[(*p*-chlorophenyl)­methyl]-1-methylindole^36^ (255 mg, 1.0 mmol) and benzaldehyde (112 μL, 117 mg, 1.1 mmol) in Et_2_O. Amounts of 2,6-lutidine (163 μL, 150 mg, 1.4 mmol) and TMSOTf (272 μL, 334 mg, 1.5 mmol) differed from the General Procedure. After the TMSOTf was added dropwise by syringe, and the mixture was stirred overnight at ambient temperature prior to workup and TBAF deprotection. The product was isolated as a yellow oil (257 mg, 71% yield) after purification on silica (2–5% ethyl acetate/hexane): IR (neat) 3409, 3027, 2893, 1488, 1471, 1366, 1174, 1092, 1013, 878, 741, 711, 698 cm^–1^; ^1^H NMR (500 MHz, CDCl_3_) δ 7.56 (dt, *J* = 7.9, 1.0 Hz, 1H), 7.40–7.27 (m, 7H), 7.27–7.12 (m, 5H), 6.35 (s, 1H), 4.26 (d, *J* = 16.4 Hz, 1H), 4.22 (d, *J* = 16.5 Hz, 1H), 3.54 (s, 3H), 2.29 (s, 1H); ^13^C­{^1^H} NMR (125 MHz, CDCl_3_) δ 141.0, 140.3, 137.7, 136.5, 131.6, 129.6, 128.5, 128.4, 127.31, 127.29, 125.8, 122.4, 119.4, 119.3, 112.5, 109.2, 67.4, 31.0, 29.5; HRMS (ESI, TOF): Exact mass calcd for C_23_H_19_NOCl [M–H]^+^, 360.1150; found, 360.1163.
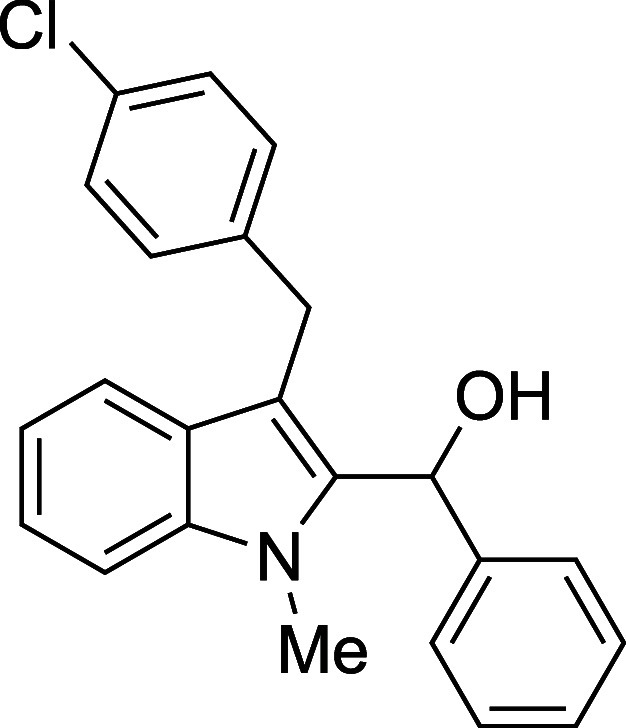


#### {3-[(*p*-Bromophenyl)­methyl]-1-methyl-2-indolyl}­phenylmethanol (**4f**)

The title compound^33^ was prepared according to a variation of the General Procedure with 3-[(*p*-bromophenyl)­methyl]-1-methylindole^36^ (300 mg, 1.0 mmol) and benzaldehyde (112 μL, 117 mg, 1.1 mmol) in Et_2_O. Amounts of 2,6-lutidine (163 μL, 150 mg, 1.4 mmol) and TMSOTf (272 μL, 334 mg, 1.5 mmol) differed from the General Procedure. After the TMSOTf was added dropwise by syringe, the mixture was stirred for 6 h at ambient temperature prior to workup and TBAF deprotection. The product was isolated as a pink foam (292 mg, 70% yield) after purification on silica (2–10% ethyl acetate/hexane): IR (solid) 3418, 3054, 3027, 2894, 1485, 1470, 1448, 1365, 1174, 1070, 1009, 877, 741, 711, 698 cm^–1^; ^1^H NMR (500 MHz, CDCl_3_) δ 7.53 (dt, *J* = 7.9, 1.0 Hz, 1H), 7.38–7.34 (m, 2H), 7.34–7.24 (m, 7H), 7.15–7.08 (m, 3H), 6.31 (s, 1H), 4.21 (d, *J* = 16.4 Hz, 1H), 4.17 (d, *J* = 16.5 Hz, 1H), 3.51 (s, 3H), 2.31 (d, *J* = 2.8 Hz, 1H); ^13^C­{^1^H} NMR (125 MHz, CDCl_3_) δ 141.0, 140.8, 137.7, 136.5, 131.5, 130.0, 128.5, 127.33, 127.29, 125.8, 122.4, 119.7, 119.5, 119.3, 112.3, 109.2, 67.4, 31.0, 29.6; HRMS (ESI, TOF): Exact mass calcd for C_23_H_19_NBr [M–OH]^+^, 388.0695; found, 388.0703.
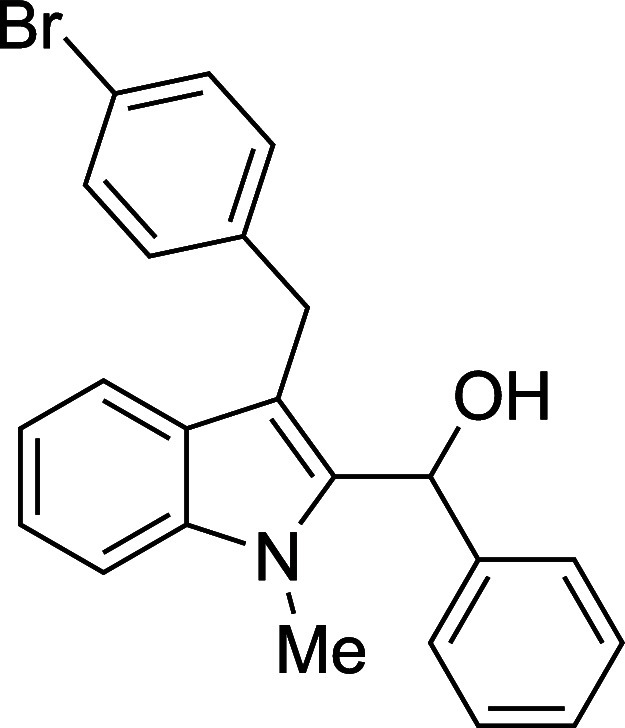


#### {3-[(*p*-Methoxyphenyl)­methyl]-1-methyl-2-indolyl}­phenylmethanol (**4g**)

The title compound^33^ was prepared according to a variation of the General Procedure with 3-[(*p*-methoxyphenyl)­methyl]-1-methylindole^36^ (251 mg, 1.0 mmol) and benzaldehyde (112 μL, 117 mg, 1.1 mmol) in Et_2_O. Amounts of 2,6-lutidine (163 μL, 150 mg, 1.4 mmol) and TMSOTf (272 μL, 334 mg, 1.5 mmol) differed from the General Procedure. After the TMSOTf was added dropwise by syringe, and the mixture was stirred overnight at ambient temperature prior to workup and TBAF deprotection. The product was isolated as a pink foam (275 mg, 77% yield) after purification on silica (0–10% ethyl acetate/hexane): IR (solid) 3419, 3348, 3028, 2901, 2834, 1609, 1508, 1493, 1471, 1366, 1240, 1172, 1107, 1027, 907, 731, 712, 700 cm^–1^; ^1^H NMR (500 MHz, CDCl_3_) δ 7.56 (dt, *J* = 8.0, 1.1 Hz, 1H), 7.34–7.19 (m, 7H), 7.14 (d, *J* = 6.6 Hz, 2H), 7.12–7.06 (m, 1H), 6.81–6.72 (m, 2H), 6.34–6.26 (m, 1H), 4.21–4.12 (m, 2H), 3.73 (s, 3H), 3.46 (s, 3H), 2.27 (d, *J* = 3.1 Hz, 1H); ^13^C­{^1^H} NMR (125 MHz, CDCl_3_) δ 157.8, 141.2, 137.7, 136.3, 134.0, 129.1, 128.3, 127.4, 127.1, 125.8, 122.2, 119.5, 119.2, 113.9, 113.5, 109.1, 67.3, 55.3, 31.0, 29.2; HRMS (ESI, TOF): Exact mass calcd for C_24_H_22_NO_2_ [M–H]^+^, 356.1645; found, 356.1659.
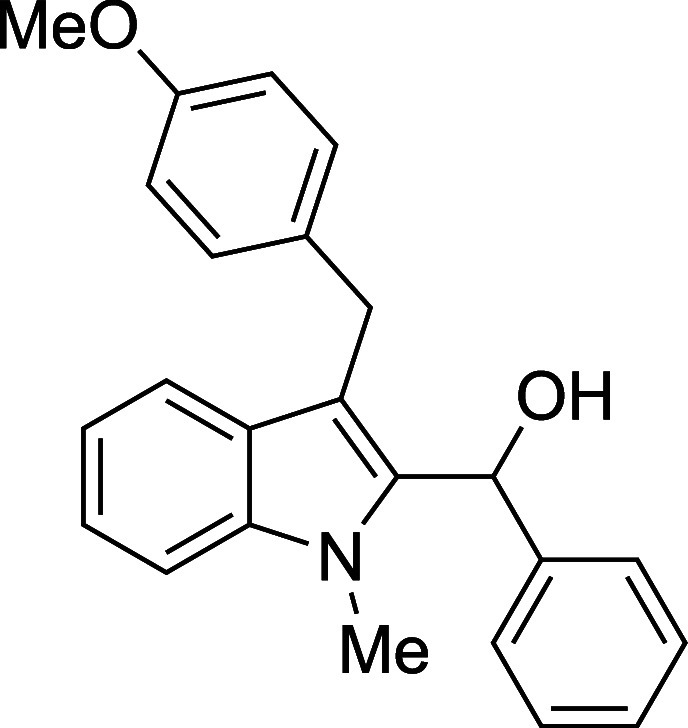


#### {1-Methyl-3-[(2-naphthyl)­methyl]-2-indolyl}­phenylmethanol (**4h**)

The title compound^33^ was prepared according to a variation of the General Procedure with 1-methyl-3-[(2-naphthyl)­methyl]­indole^36^ (257 mg, 1.0 mmol) and benzaldehyde (112 mL, 117 mg, 1.1 mmol) in Et_2_O. Amounts of 2,6-lutidine (163 μL, 150 mg, 1.4 mmol) and TMSOTf (272 μL, 334 mg, 1.5 mmol) differed from the General Procedure. After the TMSOTf was added dropwise by syringe, and the mixture was stirred for 5 h at 0 °C prior to workup and TBAF deprotection. The product was isolated as a white foam (281 mg, 77% yield) after purification on silica (2–5% ethyl acetate/hexane): IR (solid) 3418, 3052, 2892, 1471, 1364, 1014, 815, 740, 711, 699 cm^–1^; ^1^H NMR (500 MHz, CDCl_3_) δ 7.94–7.81 (m, 1H), 7.80–7.74 (m, 2H), 7.74–7.70 (m, 1H), 7.69 (dt, *J* = 7.9, 1.1 Hz, 1H), 7.51–7.44 (m, 3H), 7.39–7.27 (m, 7H), 7.19 (ddd, *J* = 8.0, 6.0, 2.0 Hz, 1H), 6.40 (d, *J* = 2.8 Hz, 1H), 4.45 (d, *J* = 1.0 Hz, 2H), 3.55 (s, 3H), 2.36 (d, *J* = 3.4 Hz, 1H); ^13^C­{^1^H} NMR (125 MHz, CDCl_3_) δ 141.2, 139.4, 137.8, 136.6, 133.7, 132.1, 128.4, 128.2, 127.7, 127.63, 127.61, 127.24, 127.21, 126.2, 126.0, 125.9, 125.3, 122.4, 119.6, 119.4, 112.9, 109.2, 67.4, 31.1, 30.4; HRMS (ESI, TOF): Exact mass calcd for C_27_H_23_NONa [M + Na]^+^, 400.1672; found, 400.1690.
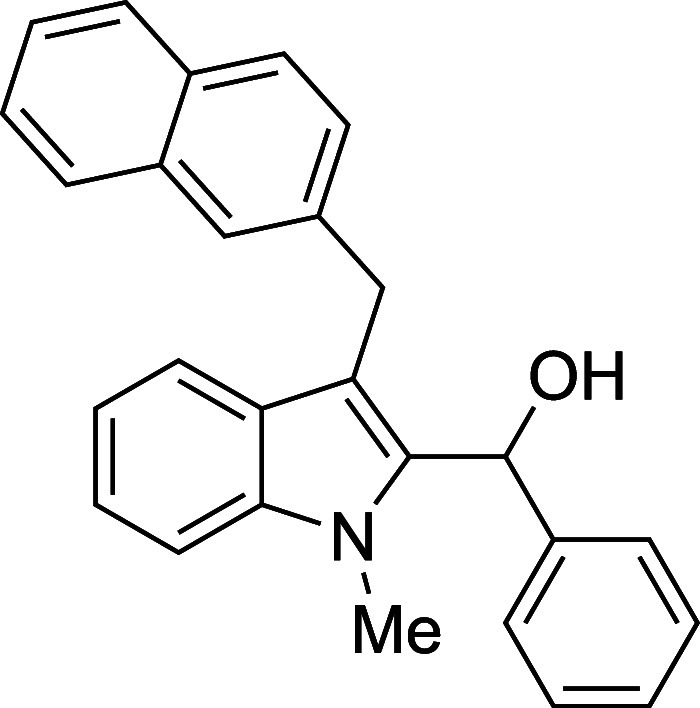


#### (3-Cyclohexyl-1-methyl-2-indolyl)­phenylmethanol (**4i**)

The title compound^33^ was prepared according to a variation of the General Procedure with 3-cyclohexyl-1-methylindole^37^ (213 mg, 1.0 mmol) and benzaldehyde (112 mL, 117 mg, 1.1 mmol) in Et_2_O. Amounts of 2,6-lutidine (163 μL, 150 mg, 1.4 mmol) and TMSOTf (272 μL, 334 mg, 1.5 mmol) differed from the General Procedure. After the TMSOTf was added dropwise by syringe, and the mixture was stirred for 6 h at ambient temperature prior to workup and TBAF deprotection. The product was isolated as a white solid (155 mg, 48% yield) after purification on silica (2–5% ethyl acetate/hexane). Product contaminated with <5% of unidentified impurity: mp: 136–140 °C; IR (solid) 3307, 3055, 3028, 2925, 2849, 1485, 1470, 1447, 1369, 1333, 1217, 1012, 887, 731, 691 cm^–1^; ^1^H NMR (500 MHz, CDCl_3_) δ 7.88 (dt, *J* = 8.0, 1.0 Hz, 1H), 7.40–7.34 (m, 4H), 7.31–7.21 (m, 3H), 7.12 (ddd, *J* = 8.0, 6.6, 1.5 Hz, 1H), 6.47 (d, *J* = 2.9 Hz, 1H), 3.46 (s, 3H), 2.95 (tt, *J* = 12.3, 3.4 Hz, 1H), 2.26 (d, *J* = 3.0 Hz, 1H), 2.15–2.01 (m, 2H), 1.96–1.86 (m, 4H), 1.84–1.79 (m, 1H), 1.47–1.36 (m, 3H); ^13^C­{^1^H} NMR (125 MHz, CDCl_3_) δ 141.5, 137.9, 134.4, 128.3, 127.0, 125.8, 125.7, 121.8, 120.9, 120.3, 118.5, 109.2, 67.0, 36.8, 34.1, 33.9, 30.9, 27.37, 27.36, 26.3; HRMS (ESI, TOF): Exact mass calcd for C_22_H_25_NONa [M + Na]^+^, 342.1828; found, 342.1835.
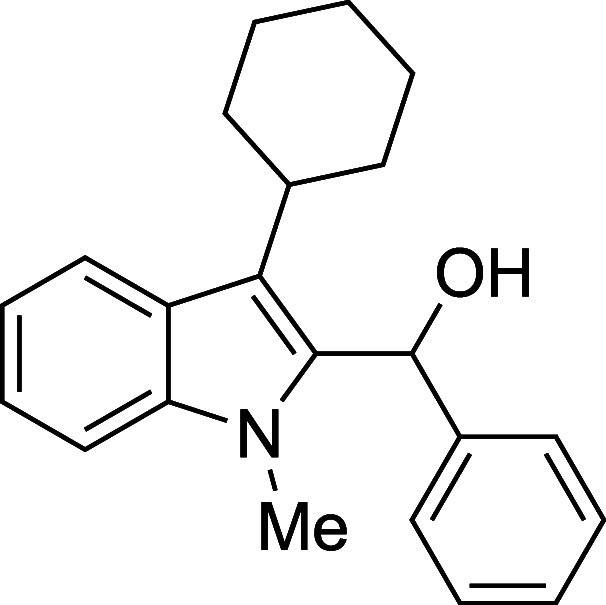


#### (1-Allyl-3-methyl-2-indolyl)­phenylmethanol (**5a**)

The title compound^33^ was prepared according to a variation of the General Procedure with 1-allyl-3-methylindole^28^ (171 mg, 1.0 mmol) and benzaldehyde (112 mL, 117 mg, 1.1 mmol) in THF. Amounts of 2,6-lutidine (163 μL, 150 mg, 1.4 mmol) and TMSOTf (272 μL, 334 mg, 1.5 mmol) differed from the General Procedure. After the TMSOTf was added dropwise by syringe, the mixture was stirred for 1 h at ambient temperature prior to workup and TBAF deprotection. The product was isolated as a yellow oil (246 mg, 89% yield) after purification on silica (2–10% ethyl acetate/hexane): IR (neat) 3374, 3059, 3028, 2916, 2859, 1448, 1358, 1326, 1179, 1019, 998, 910, 737, 697 cm^–1^; ^1^H NMR (500 MHz, CDCl_3_) δ ^1^H NMR (500 MHz, CDCl_3_) δ 7.66 (dt, *J* = 7.8, 1.1 Hz, 1H), 7.44–7.33 (m, 4H), 7.34–7.27 (m, 1H), 7.29–7.23 (m, 2H), 7.19 (dt, *J* = 8.0, 4.0 Hz, 1H), 6.40–6.33 (m, 1H), 5.66 (ddt, *J* = 17.1, 10.2, 5.1 Hz, 1H), 5.02 (dq, *J* = 10.3, 1.6 Hz, 1H), 4.87 (dq, *J* = 17.2, 1.7 Hz, 1H), 4.72 (ddt, *J* = 17.2, 4.9, 1.8 Hz, 1H), 4.61 (ddt, *J* = 17.2, 5.3, 1.7 Hz, 1H), 2.42 (m, 4H); ^13^C­{^1^H} NMR (125 MHz, CDCl_3_) δ 141.5, 137.0, 135.4, 134.0, 128.4, 128.2, 127.2, 125.8, 122.3, 119.15, 119.13, 116.0, 109.9, 109.8, 67.4, 46.7, 9.0; HRMS (ESI, TOF): Exact mass calcd for C_19_H_18_N [M–OH]^+^, 260.1434; found, 260.1431.
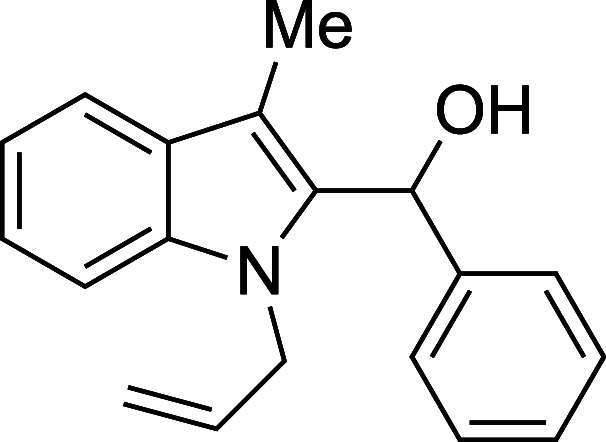


#### (1-Benzyl-3-methyl-2-indolyl)­phenylmethanol (**5b**)

The title compound^33^ was prepared according to a variation of the General Procedure with 1-benzyl-3-methylindole^29^ (221 mg, 1.0 mmol) and benzaldehyde (112 mL, 117 mg, 1.1 mmol) in THF. Amounts of 2,6-lutidine (163 μL, 150 mg, 1.4 mmol) and TMSOTf (272 μL, 334 mg, 1.5 mmol) differed from the General Procedure. After the TMSOTf was added dropwise by syringe, the mixture was stirred for 4 h at 0 °C prior to workup and TBAF deprotection. The product was isolated as a gray foam (301 mg, 92% yield) after purification on silica (2–5% ethyl acetate/hexane): IR (solid) 3544, 3427, 3067, 3029, 2918, 2865, 1467, 13451, 1353, 1331, 1264, 1176, 1028, 731, 696 cm^–1^; ^1^H NMR (500 MHz, CDCl_3_) δ 7.73–7.63 (m, 1H), 7.41–7.31 (m, 2H), 7.34–7.26 (m, 2H), 7.28–7.11 (m, 7H), 6.91–6.83 (m, 2H), 6.35 (d, *J* = 3.6 Hz, 1H), 5.32 (d, *J* = 17.0 Hz, 1H), 5.26 (d, *J* = 17.1 Hz, 1H), 2.42 (s, 3H), 2.14 (d, *J* = 4.0 Hz, 1H); ^13^C­{^1^H} NMR (125 MHz, CDCl_3_) δ 141.4, 138.4, 137.3, 135.8, 128.5, 128.4, 128.3, 127.2, 126.9, 125.9, 125.7, 122.5, 119.3, 119.1, 110.2, 109.8, 67.6, 47.6, 9.1; HRMS (ESI, TOF): Exact mass calcd for C_23_H_21_NONa [M + Na]^+^, 350.1515; found, 350.1518.
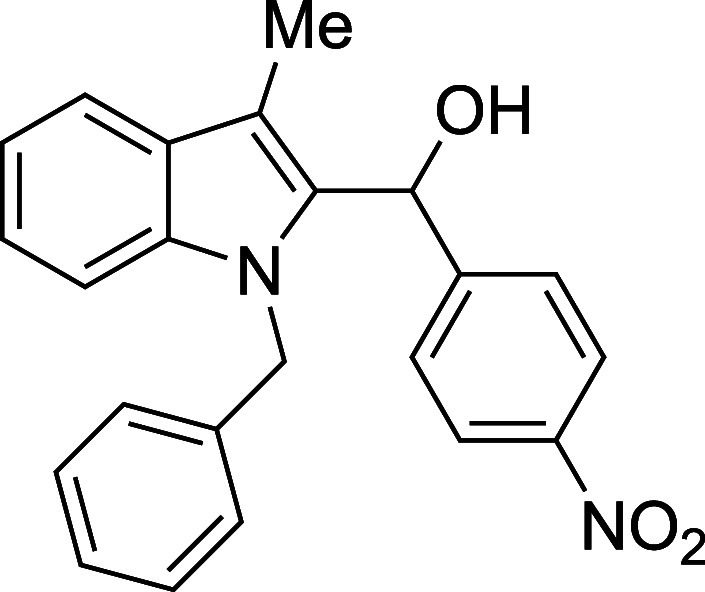


#### (1-Benzyl-3-methyl-2-indolyl)­(*p*-nitrophenyl)­methanol (**5c**)

The title compound^33^ was prepared according to a variation of the General Procedure with 1-benzyl-3-methylindole^29^ (221 mg, 1.0 mmol) and *p*-nitrobenzaldehyde (151 mg, 1.0 mmol) in THF. Amounts of 2,6-lutidine (163 μL, 150 mg, 1.4 mmol) and TMSOTf (272 μL, 334 mg, 1.5 mmol) differed from the General Procedure. After the TMSOTf was added dropwise by syringe, and the mixture was stirred for 1 h at ambient temperature prior to workup and TBAF deprotection. The product was isolated as a yellow foam (311 mg, 84% yield) after purification on silica (2–10% ethyl acetate/hexane): IR (solid) 3528, 3064, 3029, 2915, 2859, 1599, 1412, 1494, 1452, 1340, 1176, 1014, 848, 737, 694 cm^–1^; ^1^H NMR (500 MHz, CDCl_3_) δ 8.02–7.95 (m, 2H), 7.69 (dt, *J* = 7.5, 0.9 Hz, 1H), 7.45–7.39 (m, 2H), 7.27–7.16 (m, 3H), 7.15–7.08 (m, 3H), 6.75–6.66 (m, 2H), 6.38 (s, *J* = 1.1 Hz, 1H), 5.36 (d, *J* = 17.2 Hz, 1H), 5.23 (d, *J* = 17.2 Hz, 1H), 2.65 (s, 1H), 2.44 (s, 3H); ^13^C­{^1^H} NMR (125 MHz, CDCl_3_) δ 148.6, 146.9, 137.8, 137.6, 134.6, 128.4, 127.9, 126.9, 126.6, 125.6, 123.4, 123.2, 119.6, 119.4, 110.9, 109.9, 66.8, 47.4, 9.1; HRMS (ESI, TOF): Exact mass calcd for C_23_H_19_N_2_O_2_ [M–OH]^+^, 355.1441; found, 355.1447.
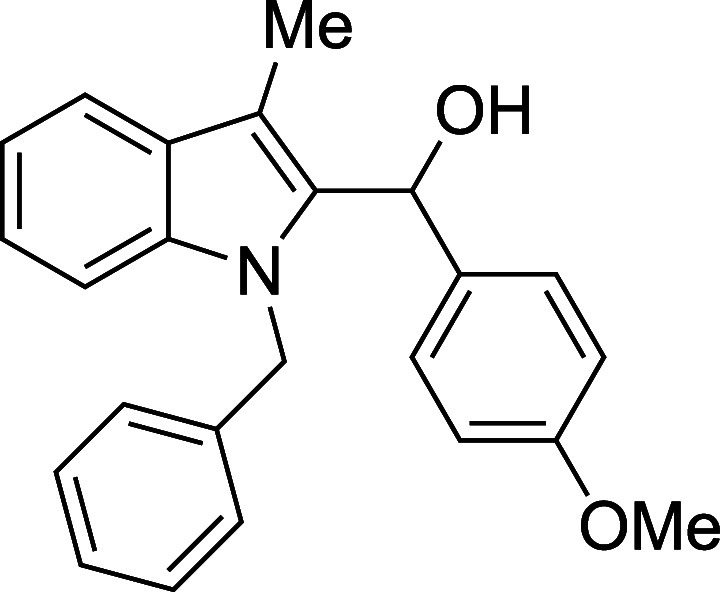


#### (1-Benzyl-3-methyl-2-indolyl)­(*p*-methoxyphenyl)­methanol (**5d**)

The title compound^16c^ was prepared according to a variation of the General Procedure with 1-benzyl-3-methylindole^29^ (221 mg, 1.0 mmol) and *p*-anisaldehyde (122 μL, 136 mg, 1.0 mmol) in THF. Amounts of 2,6-lutidine (163 μL, 150 mg, 1.4 mmol) and TMSOTf (272 μL, 334 mg, 1.5 mmol) differed from the General Procedure. The mixture was cooled to 0 °C, and TMSOTf was added dropwise by syringe. The mixture was stirred for 8 h at 0 °C prior to workup and TBAF deprotection. The product was isolated as a white solid (246 mg, 69% yield) after purification on silica gel (2–10% ethyl acetate/hexane): mp: 103–107 °C; IR (solid): 3543, 3025, 2998, 2915, 2837, 1604, 1505, 1465, 1450, 1265, 1240, 1164, 1028, 826, 767, 752 cm^–1^; ^1^H NMR (500 MHz, CDCl_3_) δ ^1^H NMR (500 MHz, CDCl_3_) δ 7.68–7.64 (m, 1H), 7.25–7.20 (m, 2H), 7.20–7.13 (m, 6H), 6.87–6.83 (m, 2H), 6.83–6.78 (m, 2H), 6.29 (dt, *J* = 4.0, 1.0 Hz, 1H), 5.28 (s, 2H), 3.79 (s, 3H), 2.42 (s, 3H), 2.08 (d, *J* = 4.0 Hz, 1H); ^13^C­{^1^H} NMR (125 MHz, CDCl_3_) δ 158.8, 138.4, 137.3, 135.9, 133.5, 128.5, 128.3, 127.0, 126.9, 125.8, 122.4, 119.2, 119.0, 113.7, 110.0, 109.7, 67.5, 55.3, 47.5, 9.1; HRMS (ESI, TOF): Exact mass calcd for C_24_H_23_NO_2_Na [M + Na]^+^, 380.1621; found, 380.1630.
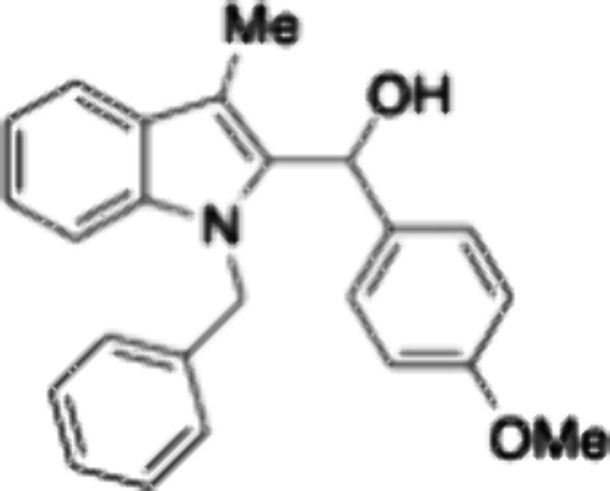


## Supplementary Material







## Data Availability

The data underlying this study are available in the published article and its .

## Notes

^1^H and ^13^C NMR spectra for compounds **1a**–**p** and **3a**–**5d** and additional experimental methods (PDF)
FAIR data, including the primary NMR FID files, for compounds **1a**–**p** (ZIP)
FAIR data, including the primary NMR FID files, for compounds **3a**–**5d** (ZIP)
