# Supplementary material for: Friedel–Crafts Addition of 3‑Alkylated Indoles to Aldehydes: 2‑Hydroxyalkylation Promoted by Trimethylsilyl Trifluoromethanesulfonate
Source: J Org Chem. 2026 Apr 29;91(18):6255–67. doi: 10.1021/acs.joc.5c03195 (PMC13162324; doi:10.1021/acs.joc.5c03195)

Friedel–Crafts Addition of 3-Alkylated Indoles to Aldehydes: 2-Hydroxyalkylation Promoted by Trimethylsilyl Trifluoromethanesulfonate

*Eric Zhou, Helen L. Xia, and C. Wade Downey**

Department of Chemistry, University of Richmond, Richmond, VA, USA 23173

Supporting Information

I. General information S1

II. Synthesis of starting materials S2

III. Optimization of reaction conditions S5

IV. Promoter screen S6

V. ^{1}H and ^{13}C NMR spectra for all products S7

General. Reactions were carried out under an atmosphere of nitrogen with a septum cap in oven-dried glassware with magnetic stirring. Tetrahydrofuran (THF), methylene chloride (CH_2Cl_2), and diethyl ether (Et_2O) were purified by passage through a bed of activated alumina. Trimethylsilyl trifluoromethanesulfonate (TMSOTf) was stored in a Schlenk flask under inert atmosphere. Certain aldehydes were distilled prior to use and stored in a refrigerator (*o*-anisaldehyde, *p*-anisaldehyde, *o*-tolualdehyde, *p*-tolualdehyde, 2-thiophenecarboxaldehyde, 2-furanaldehyde, *p*-(trifluoromethyl)benzaldehyde, *p*-fluorobenzaldehyde). All 3-methylskatole was used as received from Ambeed. All other chemicals were used as received or synthesized by literature procedures as noted. Reactions at elevated temperatures were heated in an oil bath. Purification of reaction products was carried out by flash chromatography using silica gel (230-400 mesh). Analytical thin layer chromatography was performed on silica gel plates. Visualization was accomplished with UV light and phosphomolybdic acid stain, followed by heating. Infrared spectra were recorded on an FT-IR spectrometer. ^{1}H NMR spectra were recorded on a 500 MHz spectrometer or 400 MHz spectrometer, and are reported in ppm using solvent as an internal standard ($CDCl_3$ at 7.28 ppm). Data are reported as (ap = apparent, s = singlet, d = doublet, t = triplet, q = quartet, sx = sextet, sp=septet, m = multiplet, b = broad; coupling constant(s) in Hz; integration). Proton-decoupled ^{13}C NMR spectra were recorded on a 125 MHz spectrometer or 100 MHz spectrometer and are reported in ppm using solvent as an internal standard ($CDCl_3$ at 77.0 ppm). High-resolution mass spectra were obtained by electrospray ionization (TOF, ion trap) unless otherwise noted. Melting points were determined using a capillary melting point apparatus.

General Procedure for Synthesis of 3-Alkylated Indoles

This procedure is adopted from the method reported by Jeffrey.[1] To an oven-dried, round-bottomed 100-mL flask under N_2 atmosphere were added methylene chloride (30 mL), triethylsilane (4.8 mL, 3.5 g, 30 mmol), and trifluoroacetic acid (1.1 mL, 1.7 g, 15 mmol). To a separate oven-dried, round-bottomed 50-mL flask were added methylene chloride (10 mL), 1-methylindole (1.25 mL, 1.31 g, 10.0 mmol), and aldehyde (11 mmol). After the contents were stirred for 5 min, the solution in the 50-mL flask was transferred via syringe to the 100-mL flask. The resulting reaction mixture was warmed in an oil bath to 50 °C and stirred overnight, then allowed to cool to room temperature. The mixture was diluted with 100 mL Et_2O and washed sequentially with saturated $NaHCO_3$ (100 mL) and water (100 mL). The organic layer was dried over Na_2SO_4, then filtered and concentrated in vacuo. The residue was purified by chromatography on silica gel (0-2% EtOAc/hexane).

^{1}H NMR data for previously unreported 3-alkylated indoles

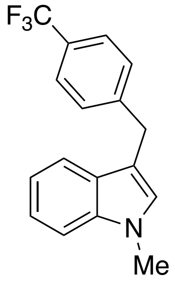

1-Methyl-3-{[*p*-(trifluoromethyl)phenyl]methyl}indole

^{1}H NMR (400 MHz, $CDCl_3$) δ 7.59 (d, *J* = 7.6 Hz, 2H), 7.56 – 7.51 (m, 1H), 7.44 (d, *J* = 7.9 Hz, 2H), 7.40 – 7.35 (m, 1H), 7.30 (tdd, *J* = 8.3, 2.9, 1.4 Hz, 1H), 7.15 (dq, *J* = 6.7, 2.7 Hz, 1H), 6.84 (s, 1H), 4.22 (s, 2H), 3.80 (s, 3H).

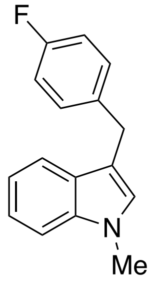

3-[(*p*-Fluorophenyl)methyl]-1-methylindole

^{1}H NMR (500 MHz, $CDCl_3$) δ 7.51 (dq, *J* = 8.0, 1.0 Hz, 1H), 7.33 (dd, *J* = 8.2, 1.0 Hz, 1H), 7.28 – 7.23 (m, 3H), 7.10 (ddt, *J* = 7.9, 6.9, 1.0 Hz, 1H), 7.02 – 6.95 (m, 2H), 6.78 (s, 1H), 4.10 (s, 2H), 3.77 (s, 3H).

---

[1] Acharya, A.; Anumandla, D.; Jeffrey, C. S. Dearomative Indole Cycloaddition Reactions of Aza-Oxyallyl Cationic Intermediates: Modular Access to Pyrroloindolines. *J. Am. Chem. Soc.* **2015**, *137*, 14858-14860. doi: 10.1021/jacs.5b10184.

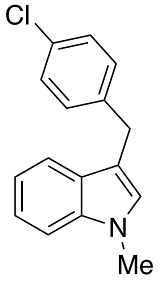

3-[(*p*-Chlorophenyl)methyl]-1-methylindole

^{1}H NMR (500 MHz, $CDCl_3$) δ 7.38 (dt, *J* = 8.0, 1.0 Hz, 1H), 7.20 (dt, *J* = 8.3, 1.0 Hz, 1H), 7.17 – 7.10 (m, 5H), 6.99 (ddd, *J* = 7.9, 6.9, 1.0 Hz, 1H), 6.66 (s, 1H), 3.98 (s, 2H), 3.64 (s, 3H).

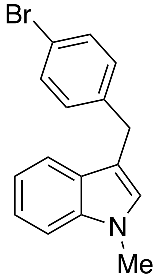

3-[(*p*-Bromophenyl)methyl]-1-methylindole

^{1}H NMR (400 MHz, $CDCl_3$) δ 7.50 (dt, *J* = 7.8, 1.0 Hz, 1H), 7.46 – 7.38 (m, 2H), 7.33 (d, *J* = 8.2 Hz, 1H), 7.30 – 7.23 (m, 1H), 7.22 – 7.15 (m, 2H), 7.11 (dd, *J* = 7.9, 6.9 Hz, 1H), 6.79 (s, 1H), 4.09 (s, 2H), 3.77 (s, 3H).

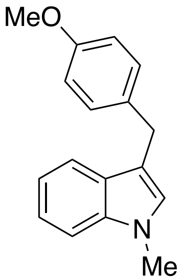

3-[(*p*-Methoxyphenyl)methyl]-1-methylindole

^{1}H NMR (500 MHz, $CDCl_3$) δ 7.55 (d, *J* = 7.9 Hz, 1H), 7.35 – 7.29 (m, 1H), 7.27 – 7.20 (m, 3H), 7.10 (dt, *J* = 8.0, 6.9 Hz, 1H), 6.89 – 6.83 (m, 2H), 6.77 (s, 1H), 4.07 (s, 2H), 3.82 (s, 3H), 3.76 (s, 3H).

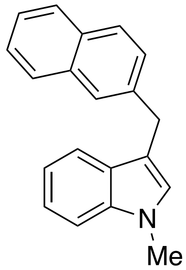

1-Methyl-3-[(2-naphthyl)methyl]indole

^{1}H NMR (500 MHz, $CDCl_3$) δ 8.08 – 8.02 (m, 1H), 8.02 – 7.95 (m, 3H), 7.83 (dt, *J* = 7.9, 1.0 Hz, 1H), 7.72 – 7.62 (m, 3H), 7.53 – 7.44 (m, 2H), 7.35 (ddd, *J* = 8.0, 5.7, 2.2 Hz, 1H), 6.92 (sz, 1H), 4.51 (s, 2H), 3.81 (s, 3H).

General Procedure for Optimization Studies

To an oven-dried round-bottomed 5-mL flask under N_2 atmosphere were added the indole (0.1 mmol), solvent (0.5 mL), benzaldehyde (0.1 mmol), and 2,6-lutidine (17 µL, 16 mg, 0.15 mmol). To this solution TMSOTf (25 µL, 26 mg, 0.14 mmol) was added dropwise by syringe. The reaction was stirred at the indicated temperature for the indicated time, then quenched with pyridine (21 µL, 21 mg, 0.26 mmol) and passed through a plug of silica (3 cm x 0.3 cm) with Et_2O (5 mL) or a 1:1 mixture of EtOAc:hexane (5 mL). The solvent was removed in vacuo, and the unpurified reaction mixture was analyzed by ^1H NMR spectroscopy.

Table S1. Optimization of Reaction Conditions

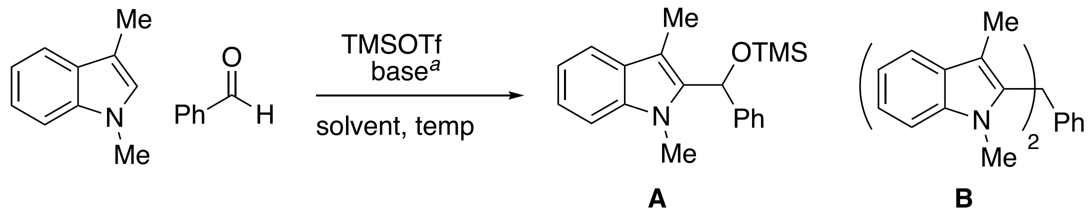

base (equiv)	TMSOTf (equiv)	solvent	T (°C)	conv (%)[b]	**A**:**B**[b]
Et_3N (1.4)	1.5	Et_2O	23	99	8:1
i-Pr_2NEt (1.4)	1.5	Et_2O	23	99	8:1
2,6-lutidine (1.4)	1.5	Et_2O	23	100	16:1
2,6-lutidine (1.5)	1.4	Et_2O	23	100	30:1
2,6-lutidine (1.5)	1.4	toluene	23	100	12:1
2,6-lutidine (1.5)	1.4	CH_2Cl_2	23	100	17:1
2,6-lutidine (1.5)	1.4	CyH	23	98	20:1
2,6-lutidine (1.5)	1.4	THF	23	100	32:1
2,6-lutidine (1.5)	1.4	THF	0	93	40:1
2,6-lutidine (1.5)	1.4	THF	–78	0	-

a Typical reaction conditions: 0.1 mmol *N*-methylskatole, 0.1 mmol benzaldehyde, 0.5 mL solvent, 1h.

b Determined by ^1H NMR spectroscopy of unpurified reaction mixture

General Procedure for Promoter Screen

To an oven-dried round-bottomed 5-mL flask under N_2 atmosphere were added the indole (0.1 mmol), THF (0.5 mL), benzaldehyde (0.1 mmol), and, if used, 2,6-lutidine (17 μL, 16 mg, 0.15 mmol). To this solution the promoter was added. The reaction was stirred for 1 h at ambient temperature, then quenched with pyridine (21 μL, 21 mg, 0.26 mmol) and passed through a plug of silica (3 cm x 0.3 cm) with Et_2O (5 mL) or a 1:1 mixture of EtOAc:hexane (5 mL). The solvent was removed in vacuo, and the unpurified reaction mixture was analyzed by ^1H NMR spectroscopy.

Table S2. Promoter Survey

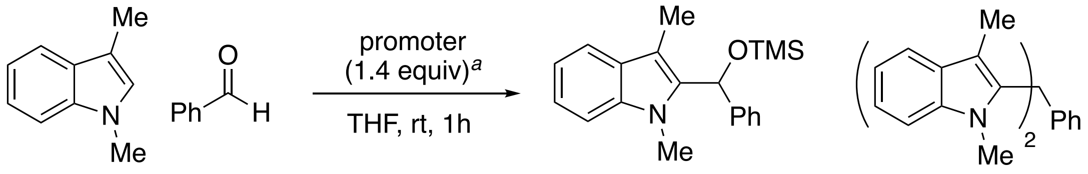

A B

promoter	2,6-lutidine (equiv)	conv (%)^b	**A**:**B**^{b}
$LiClO_4$	1.5	0	–
$LiClO_4$	0	0	–
$MgBr_2$•OEt_2	1.5	0	–
$MgBr_2$•OEt_2	0	0	–
$Fe(OTf)_3$	1.5	5	0:100
$Fe(OTf)_3$	0	90	0:100
$Zn(OTf)_2$	1.5	0	–
$Zn(OTf)_2$	0	0	–
BF_3•OEt_2	1.5	0	–
BF_3•OEt_2	0	0	–
TMSCl	1.5	0	–
TMSCl	0	0	–
TMSOTf	1.5	100	32:1
TMSOTf	0	100	0:100

a Typical reaction conditions: 0.1 mmol *N*-methylskatole, 0.1 mmol benzaldehyde, 0.5 mL solvent, 1h.

b Determined by ^1H NMR spectroscopy of unpurified reaction mixture

^{1}H NMR spectrum for product **1a** ($CDCl_3$, 500 MHz)

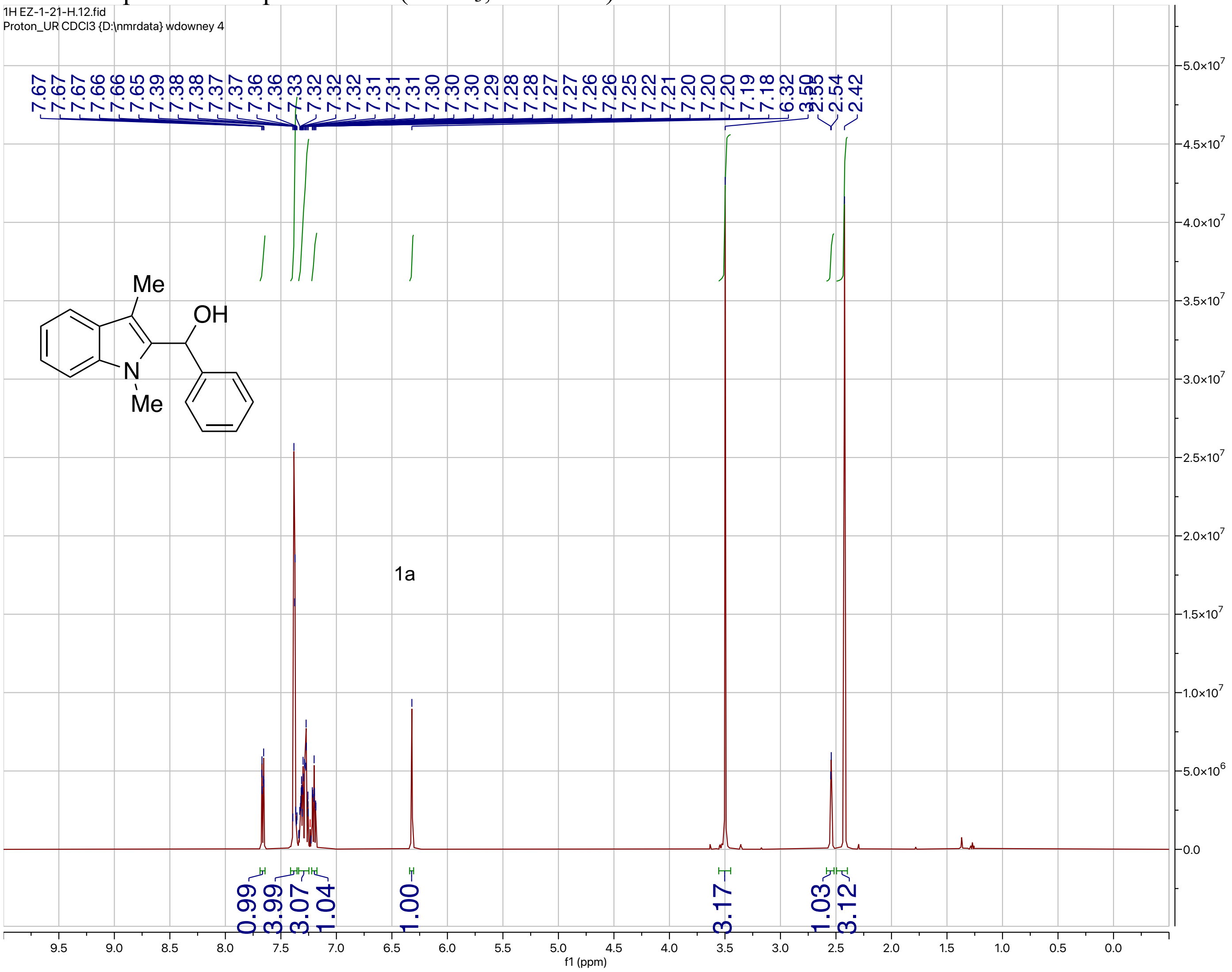

$^{13}C\{^{1}H\}$ NMR spectrum for product **1a** ($CDCl_3$, 126 MHz)

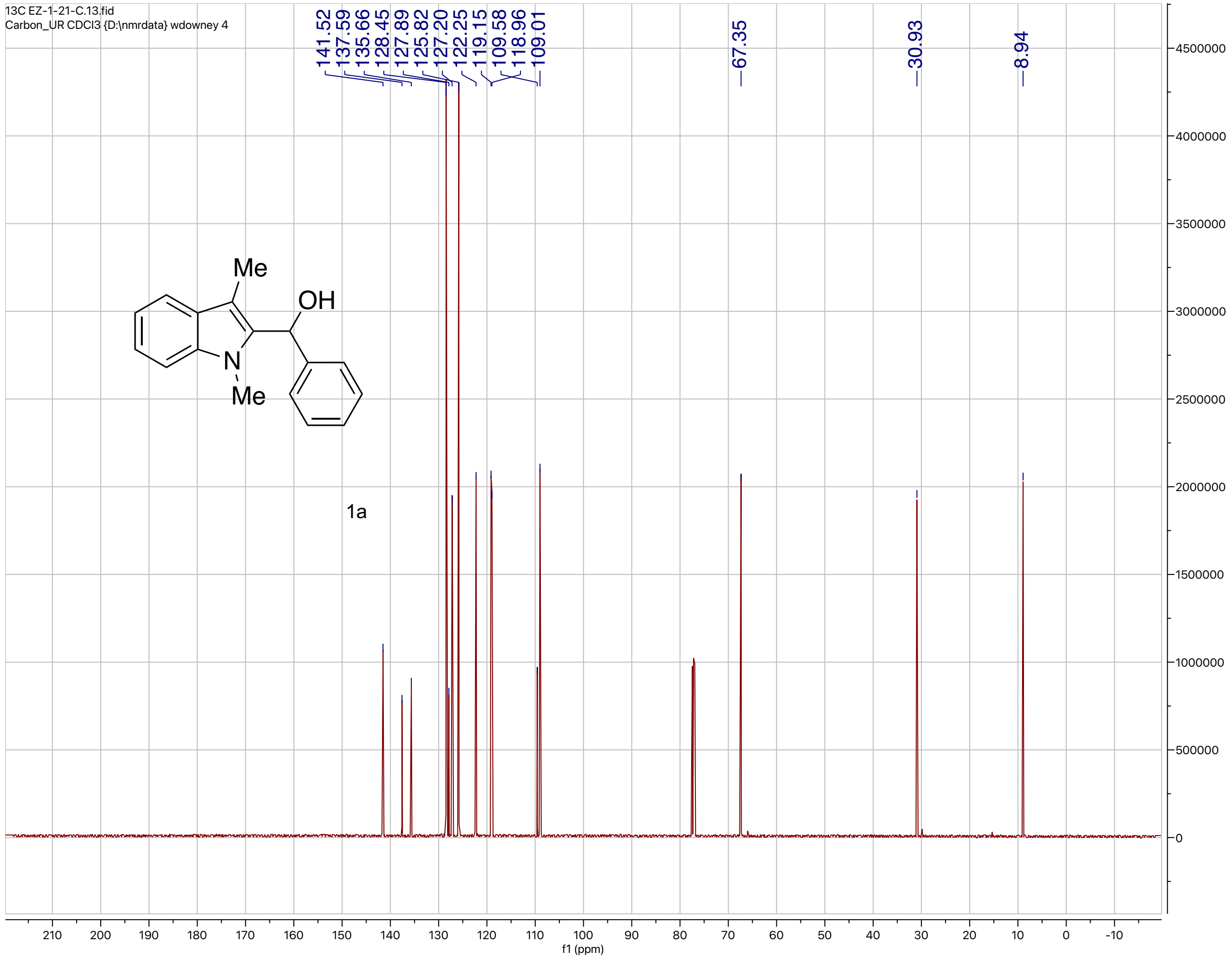

^{1}H NMR spectrum for product **1b** ($CDCl_3$, 500 MHz)

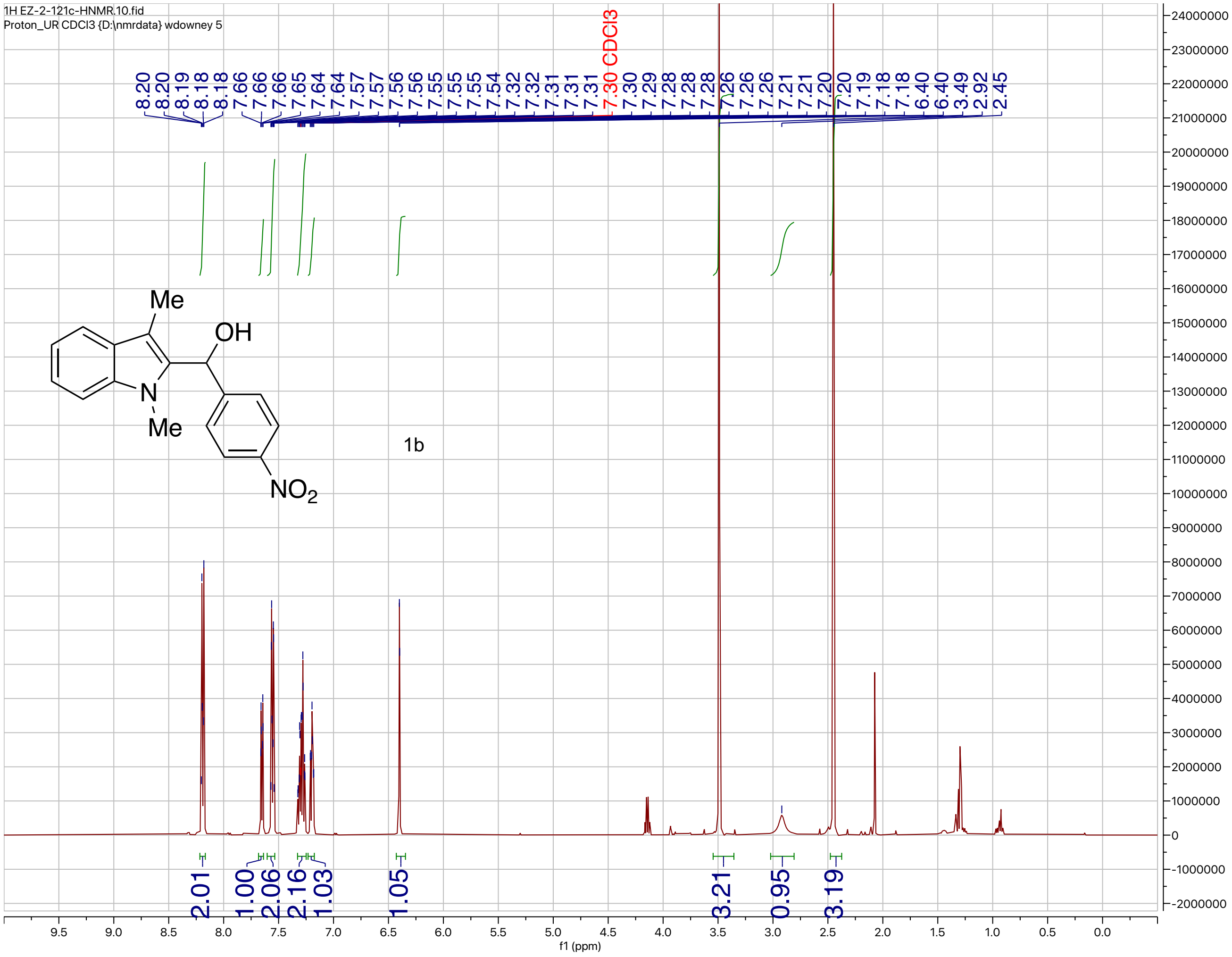

$^{13}C\{^{1}H\}$ NMR spectrum for product **1b** ($CDCl_3$, 126 MHz)

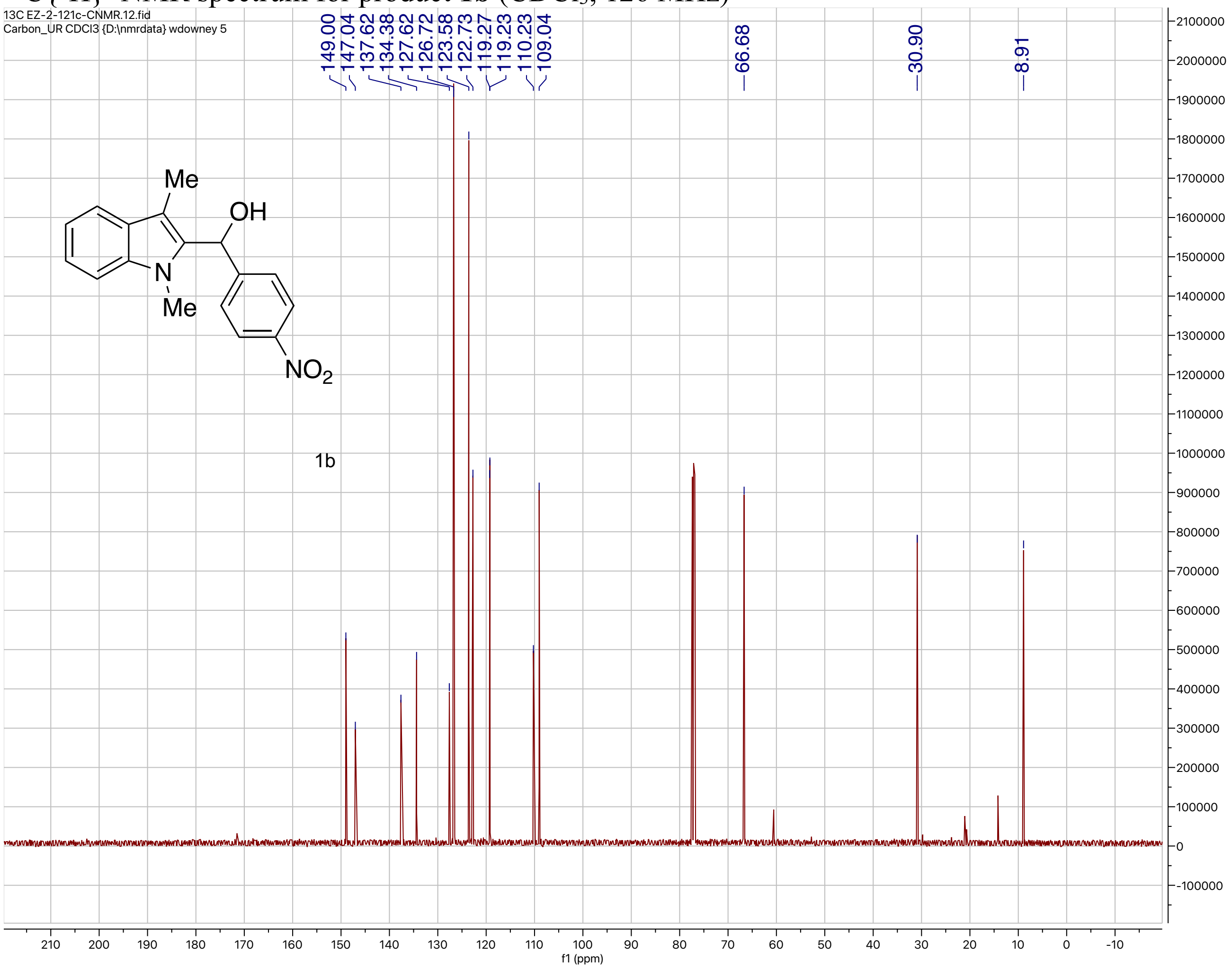

^{1}H NMR spectrum for product **1c** ($CDCl_3$, 500 MHz)

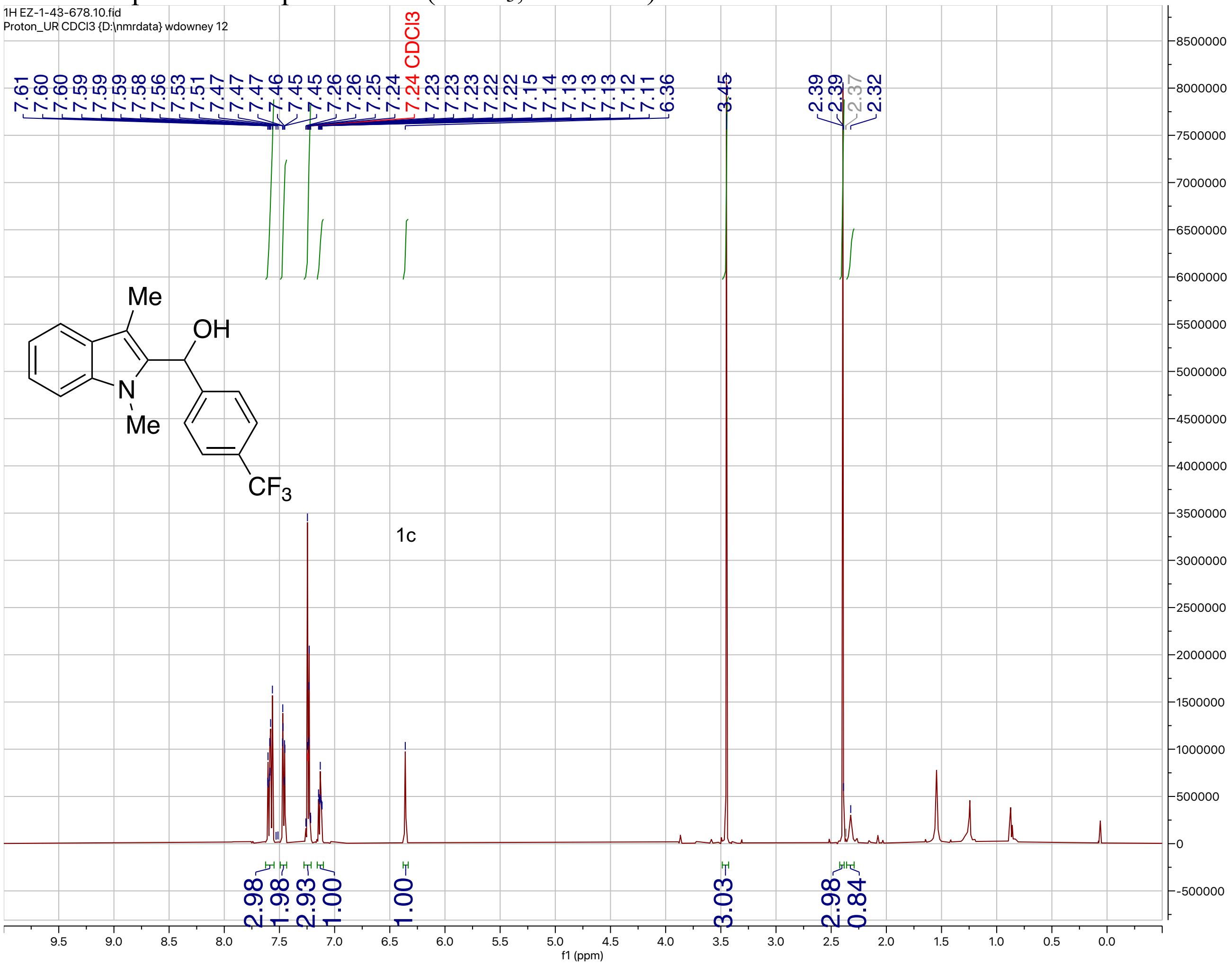

$^{13}C\{^{1}H\}$ NMR spectrum for product **1c** ($CDCl_3$, 126 MHz)

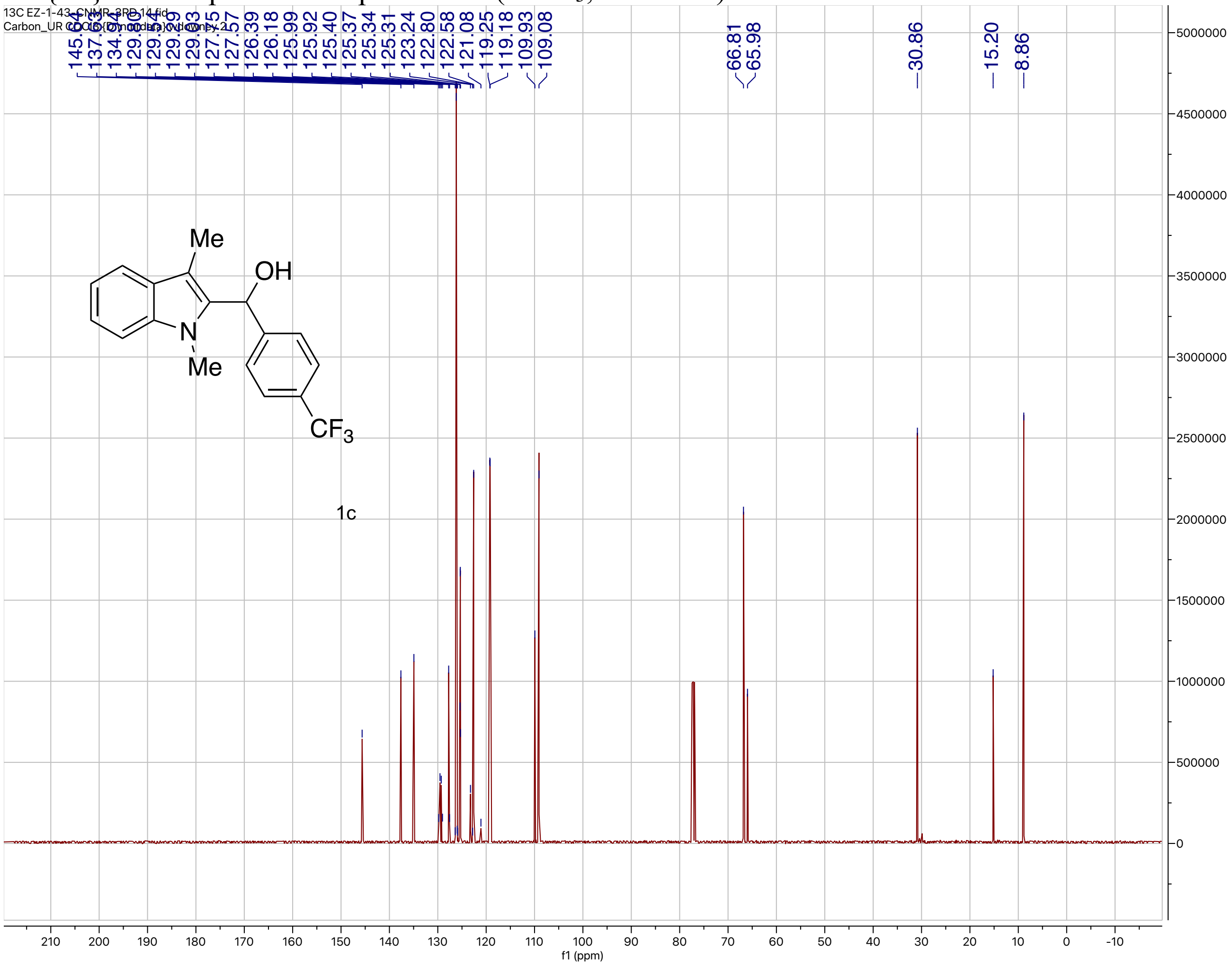

^{1}H NMR spectrum for product **1d** (CDCl$_3$, 500 MHz)

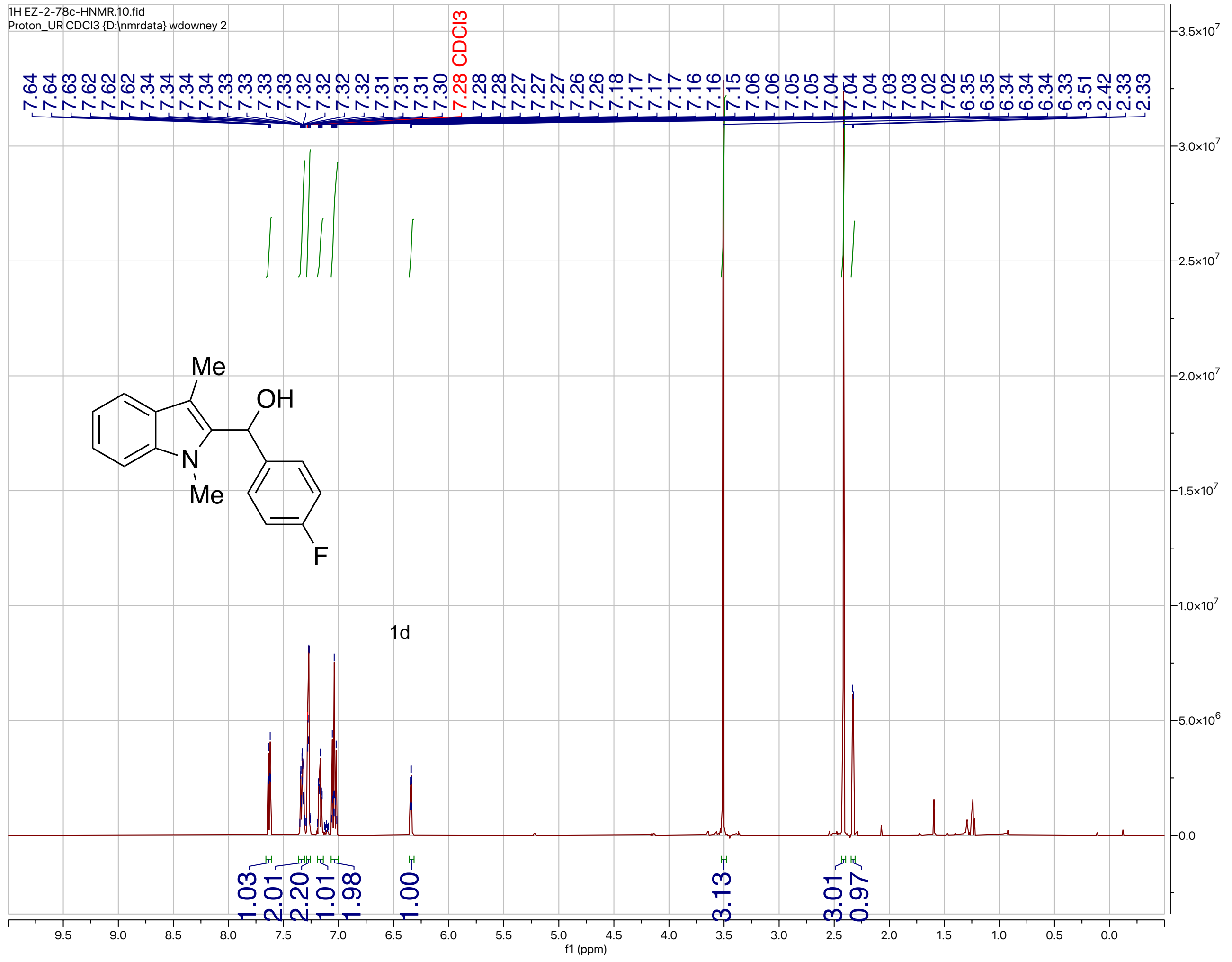

$^{13}C\{^{1}H\}$ NMR spectrum for product **1d** ($CDCl_3$, 126 MHz)

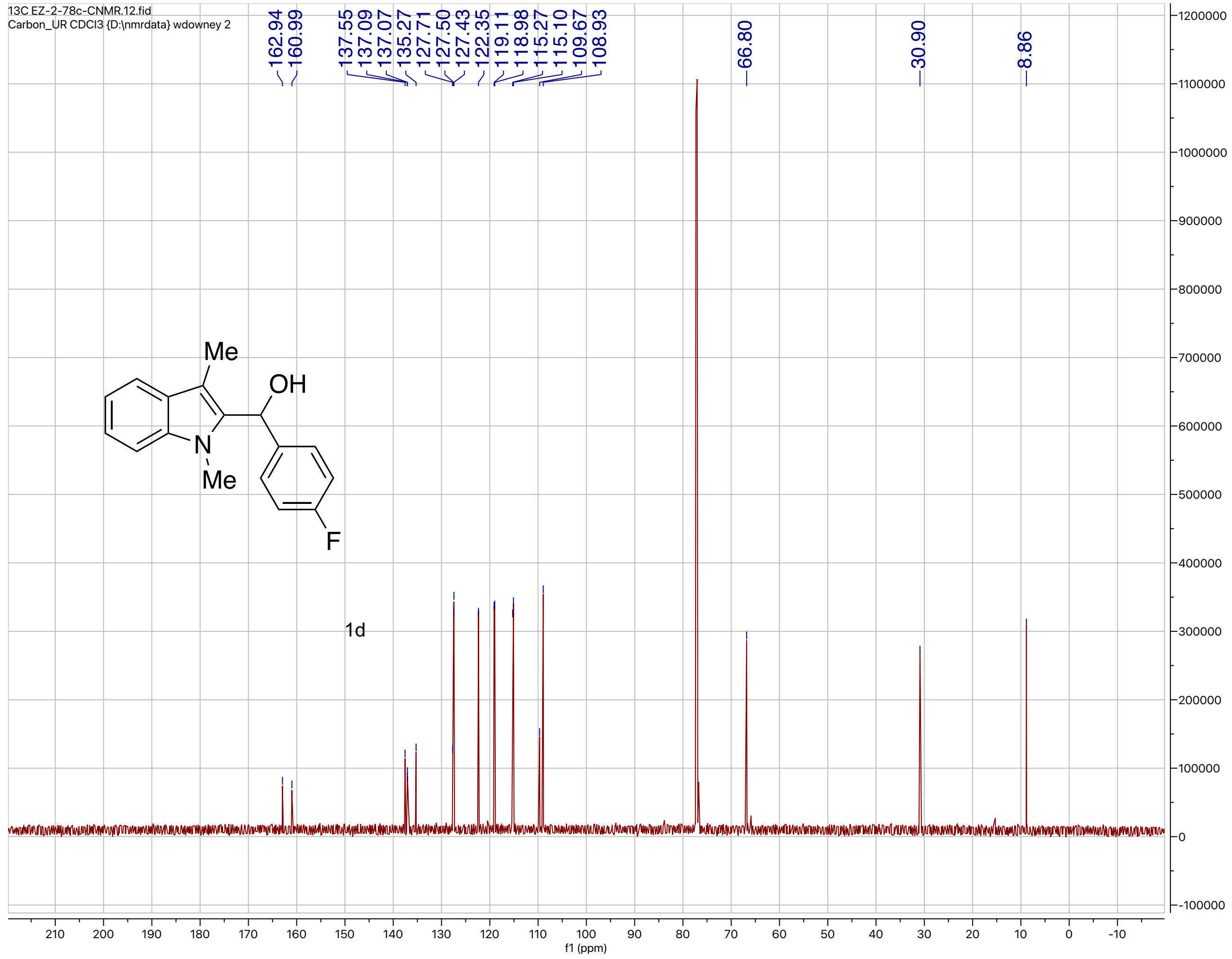

^{1}H NMR spectrum for product **1e** (CDCl$_3$, 500 MHz)

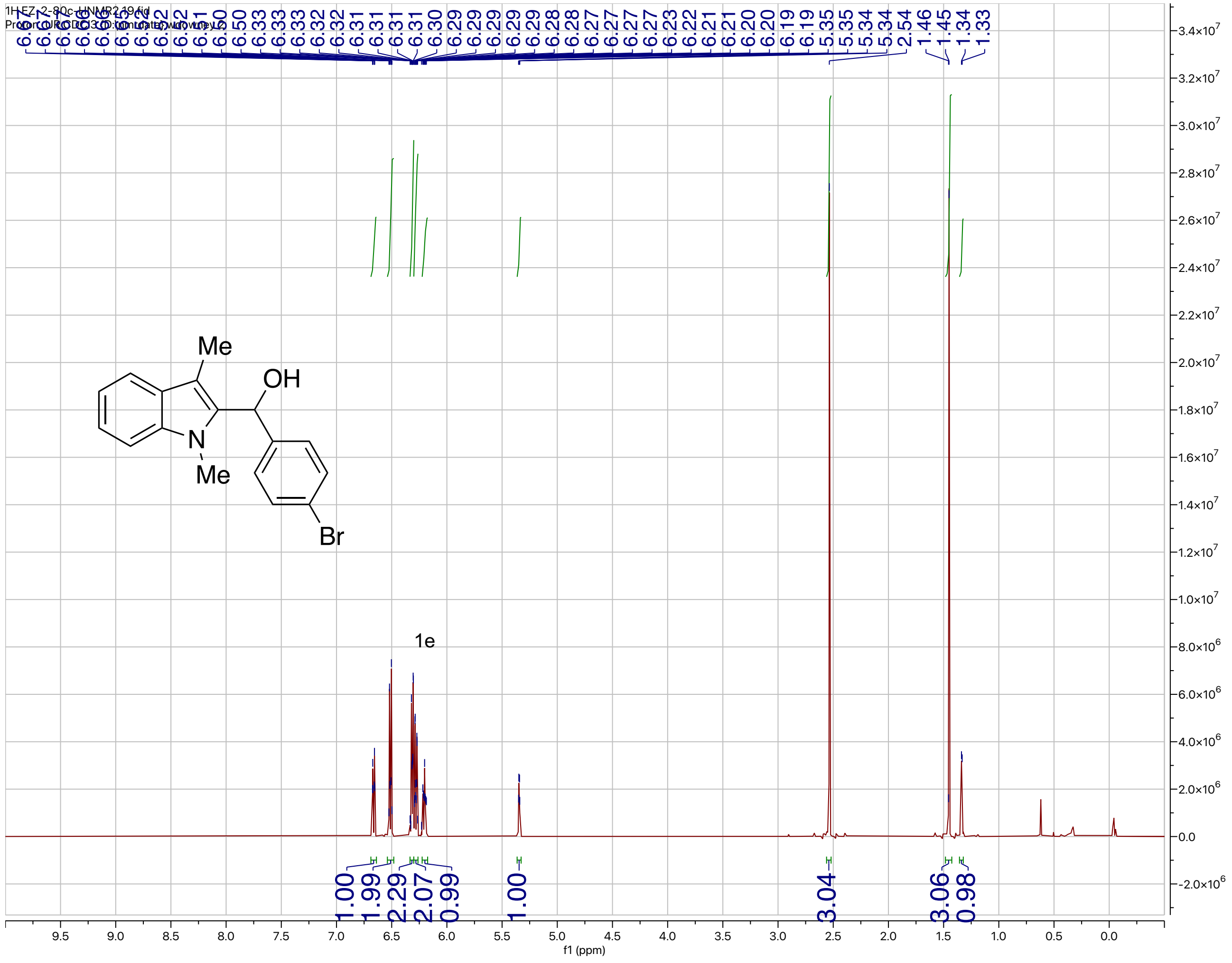

$^{13}C\{^1H\}$ NMR spectrum for product **1e** ($CDCl_3$, 126 MHz)

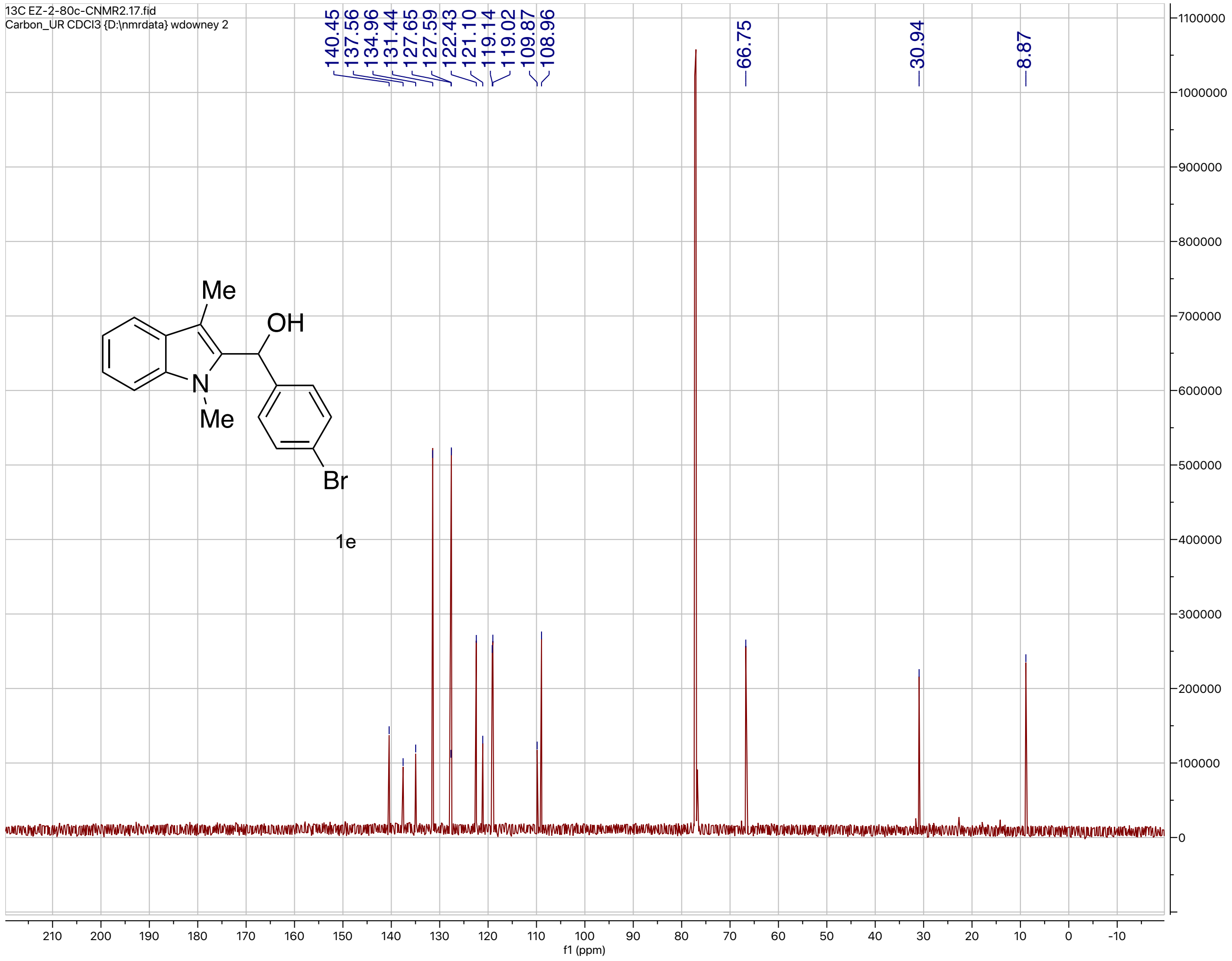

^{1}H NMR spectrum for product **1f** ($CDCl_3$, 500 MHz)

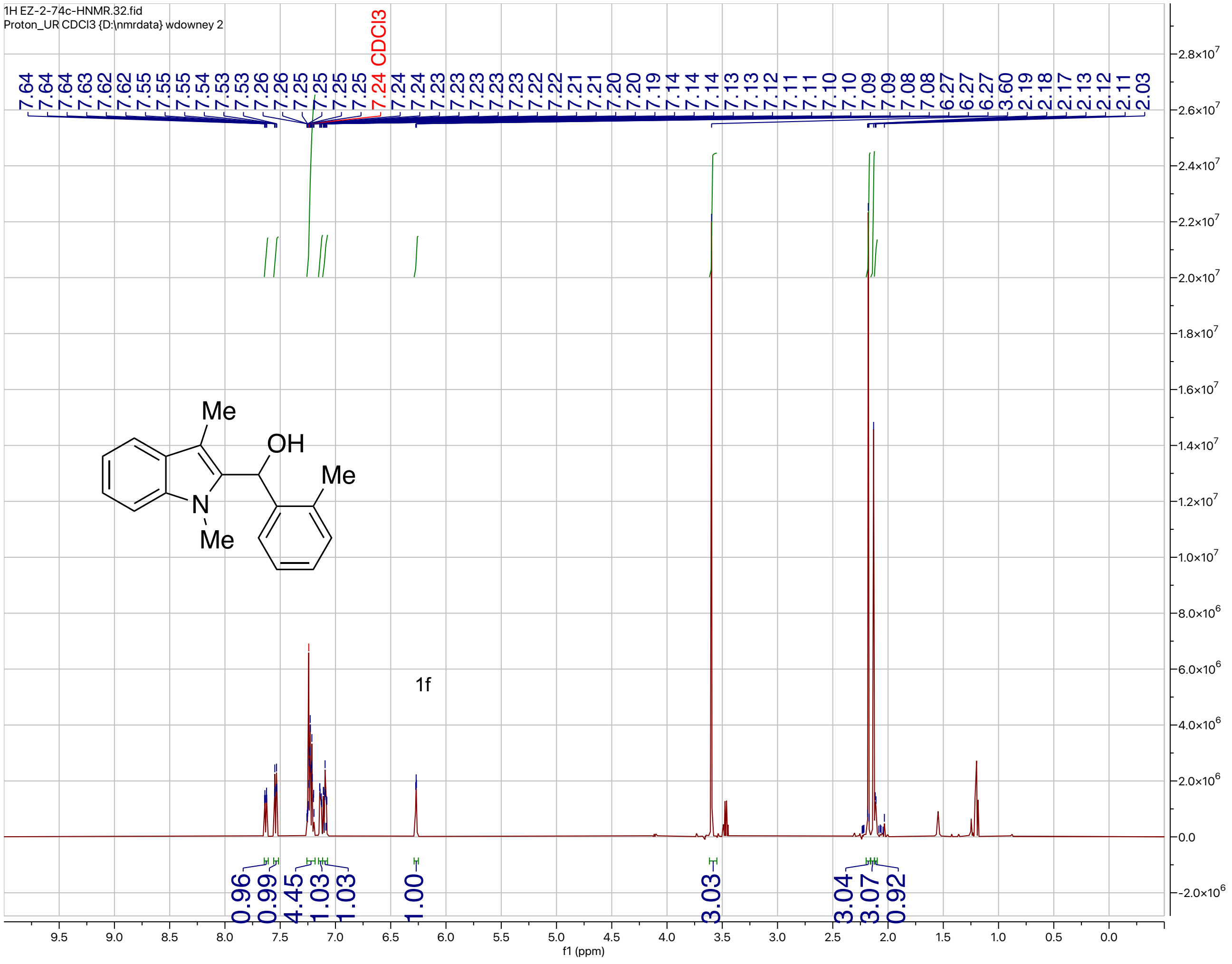

$^{13}C\{^{1}H\}$ NMR spectrum for product **1f** ($CDCl_3$, 126 MHz)

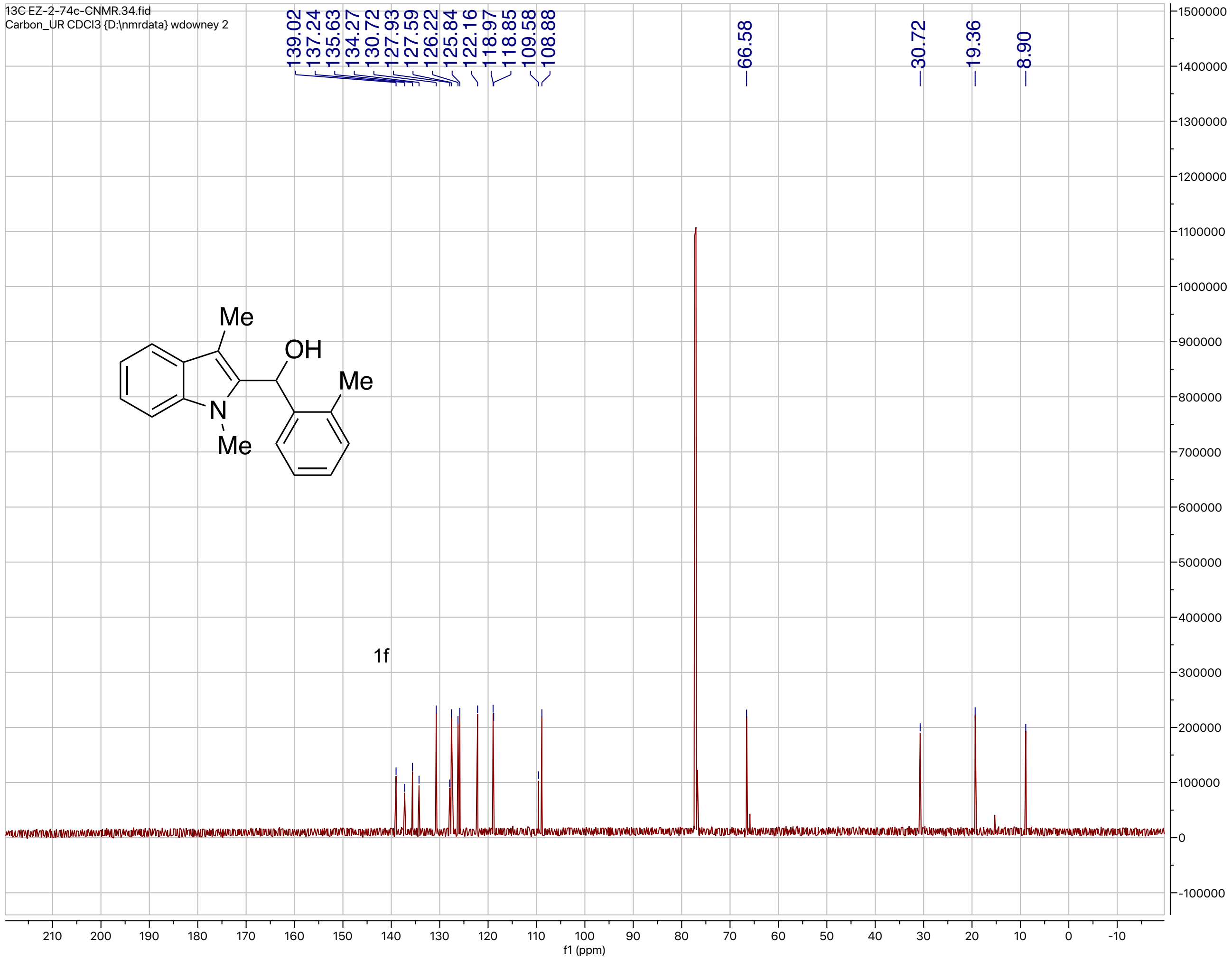

^{1}H NMR spectrum for product **1g** ($CDCl_3$, 500 MHz)

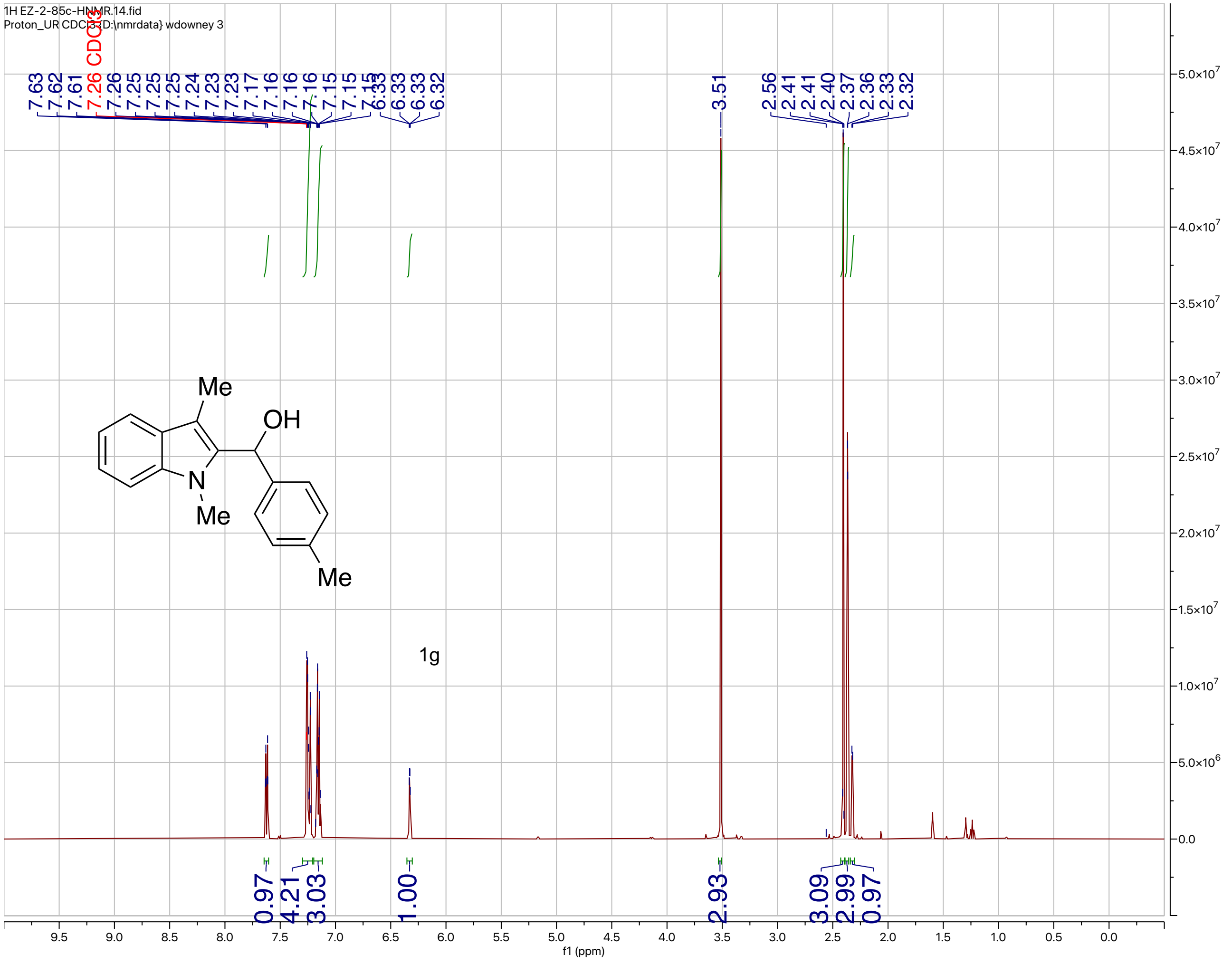

$^{13}C\{^{1}H\}$ NMR spectrum for product **1g** ($CDCl_3$, 126 MHz)

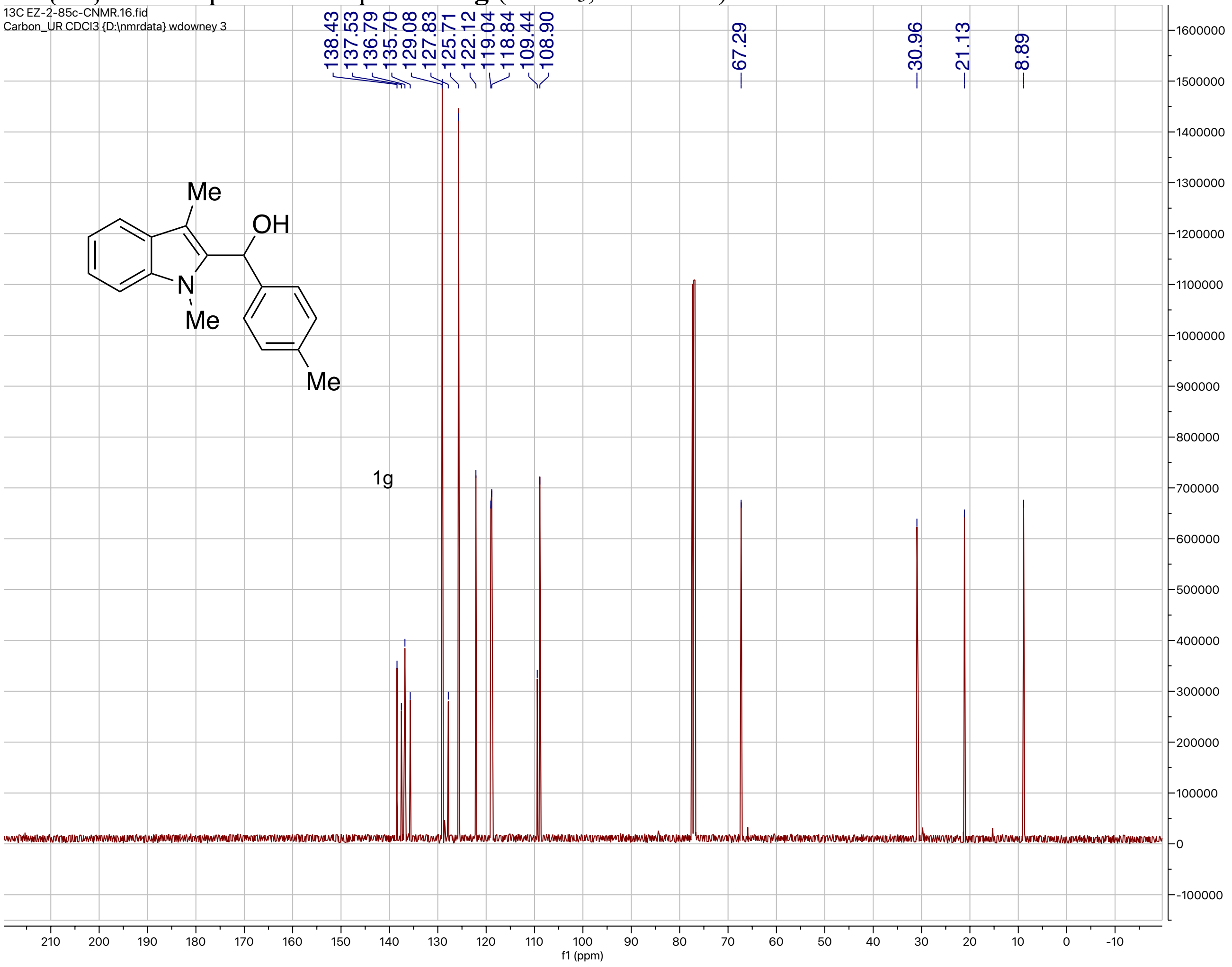

^{1}H NMR spectrum for product **1h** ($CDCl_3$, 500 MHz)

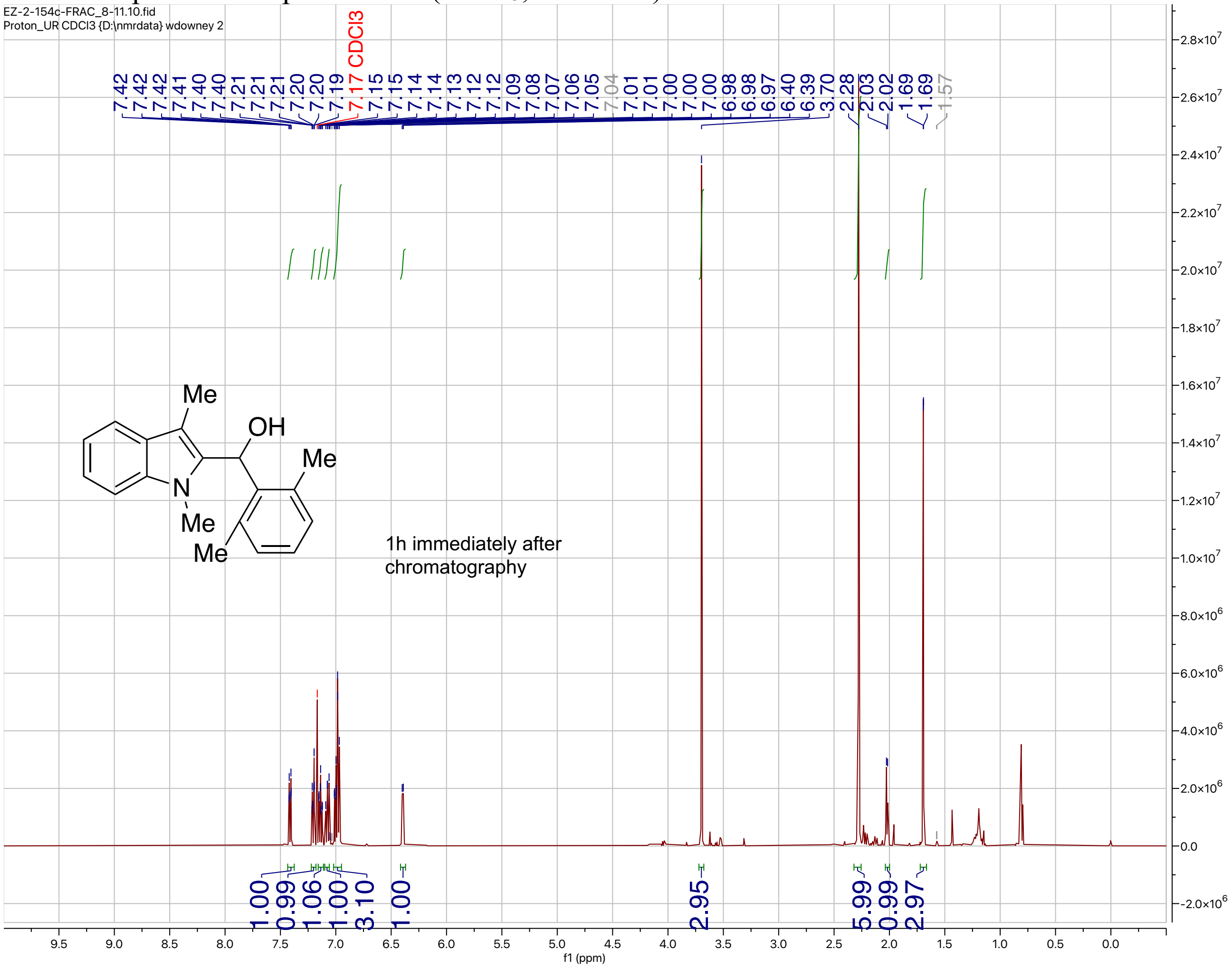

^{1}H NMR spectrum for product **1h** ($CDCl_3$, 500 MHz)

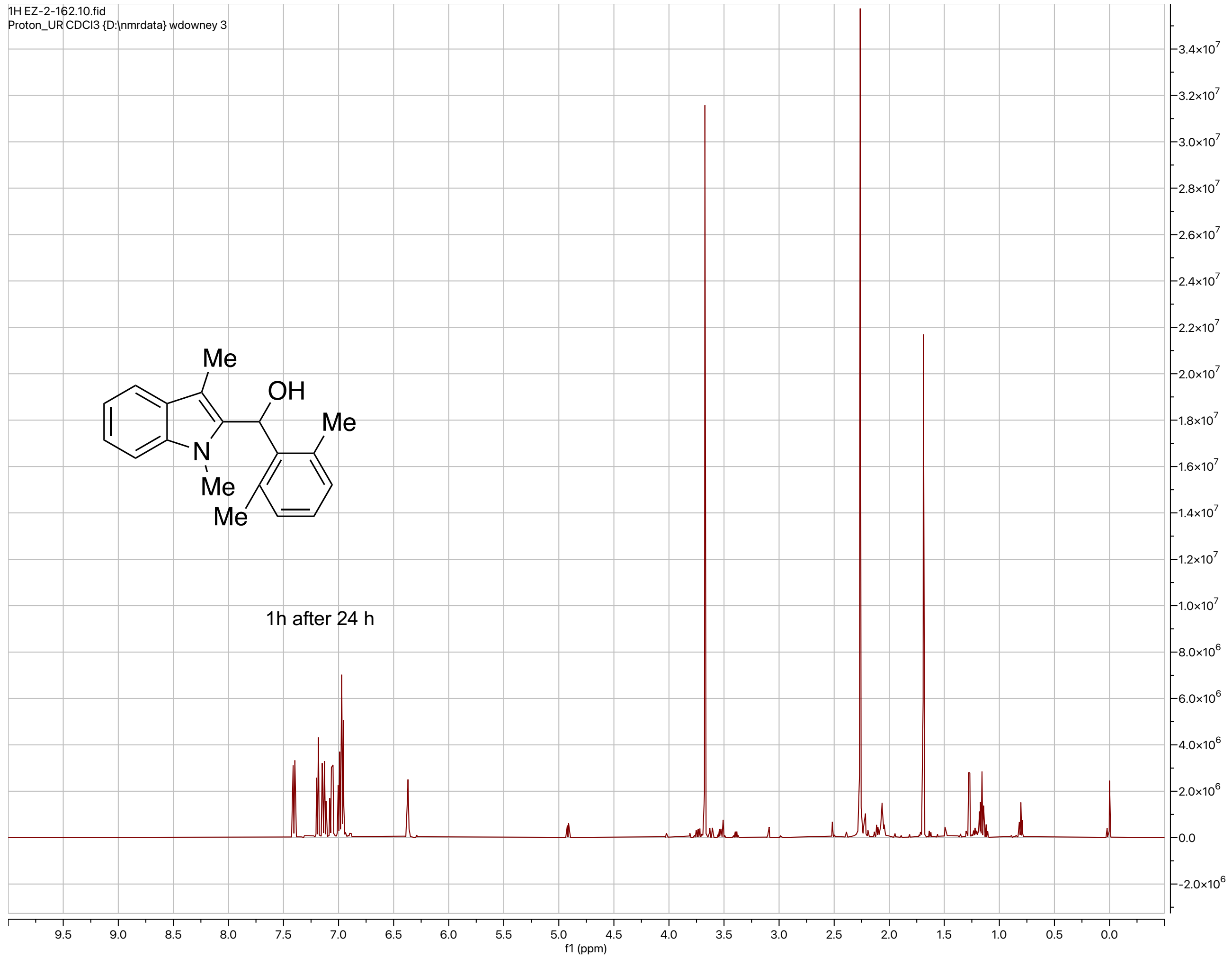

$^{13}C\{^{1}H\}$ NMR spectrum for product **1h** ($CDCl_3$, 126 MHz)

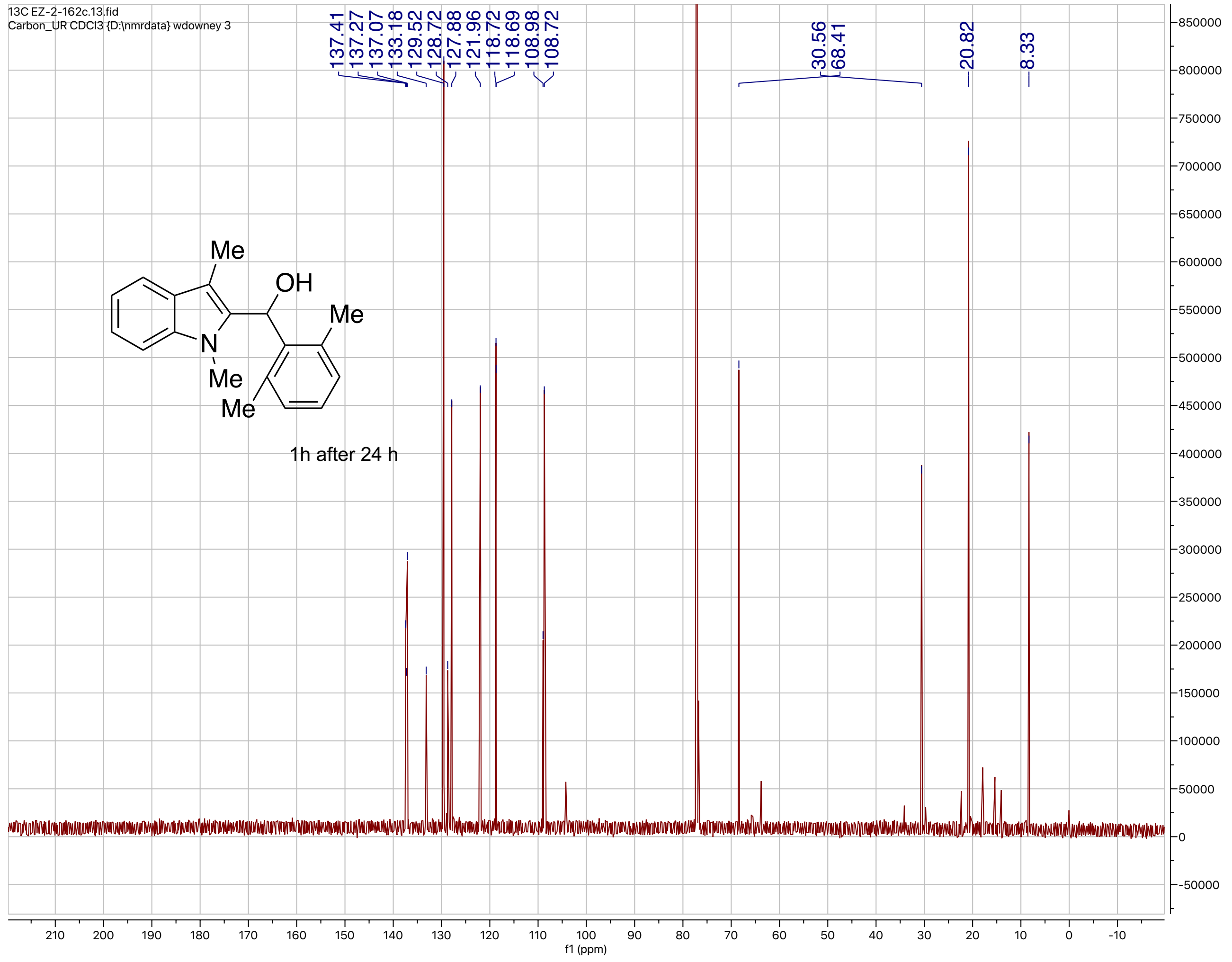

^{1}H NMR spectrum for product **1i** ($CDCl_3$, 400 MHz)

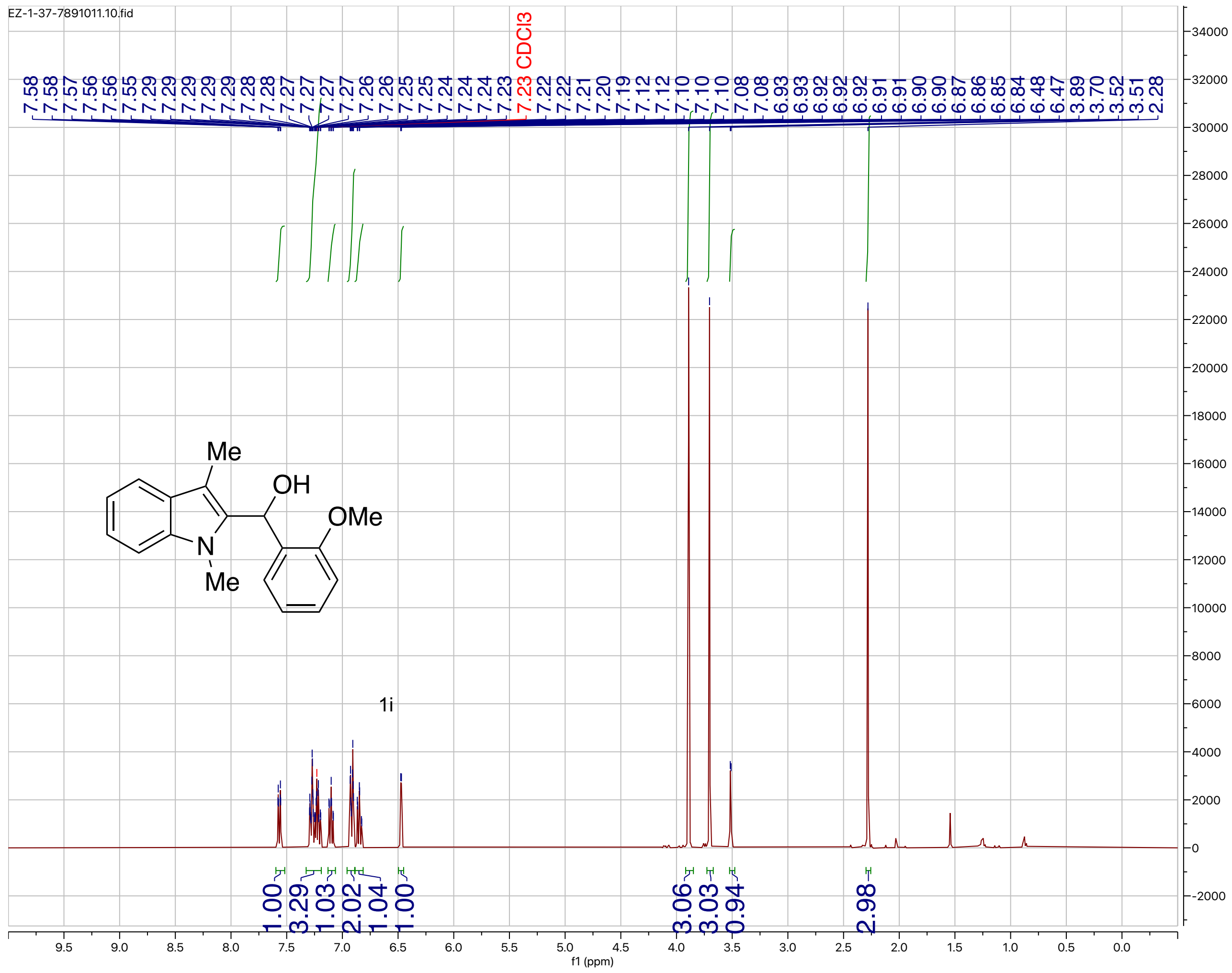

$^{13}C\{^{1}H\}$ NMR spectrum for product **1i** ($CDCl_3$, 100 MHz)

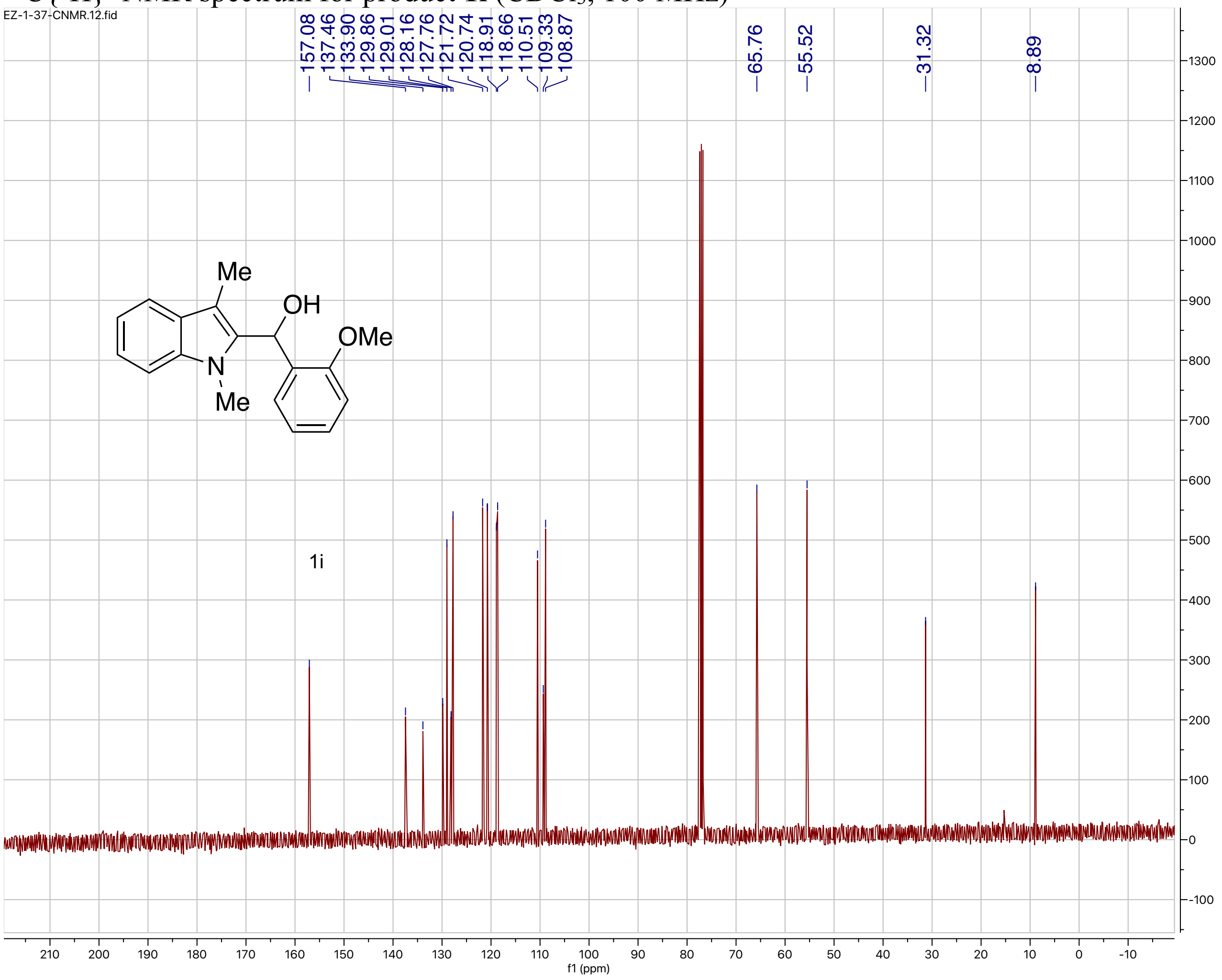

^{1}H NMR spectrum for product **1j** ($CDCl_3$, 500 MHz)

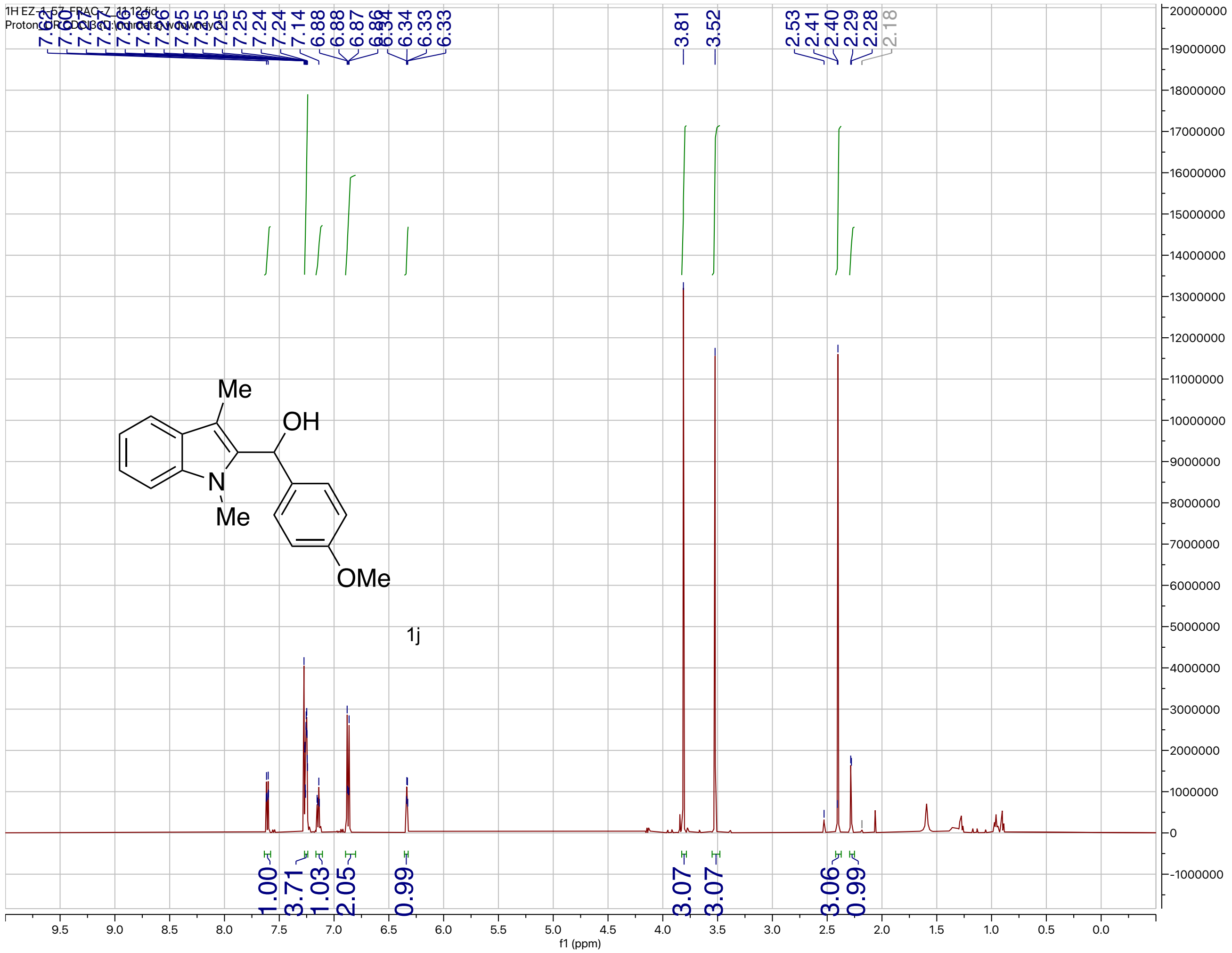

$^{13}C\{^{1}H\}$ NMR spectrum for product **1j** ($CDCl_3$, 126 MHz)

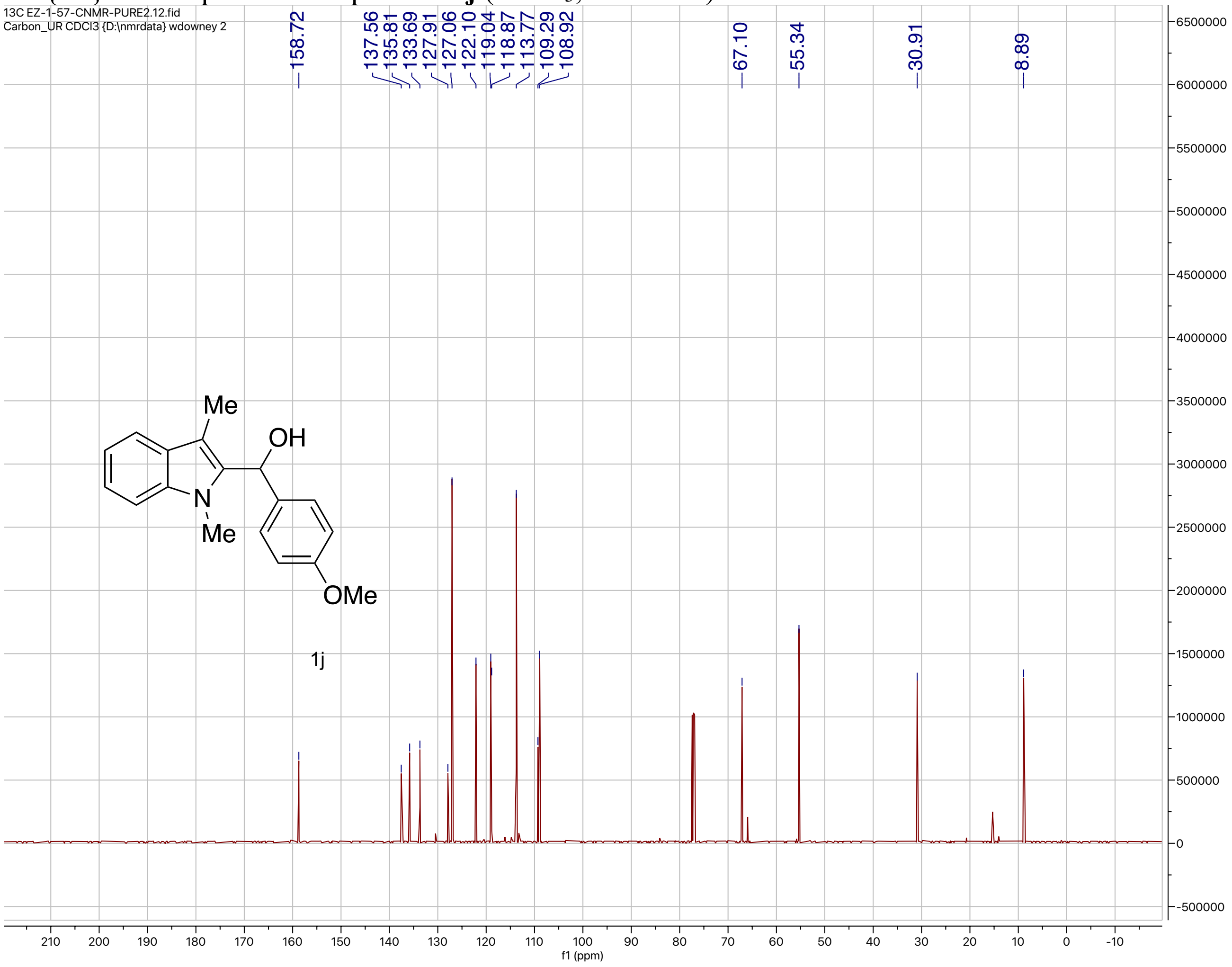

^{1}H NMR spectrum for product **1l** ($CDCl_3$, 400 MHz)

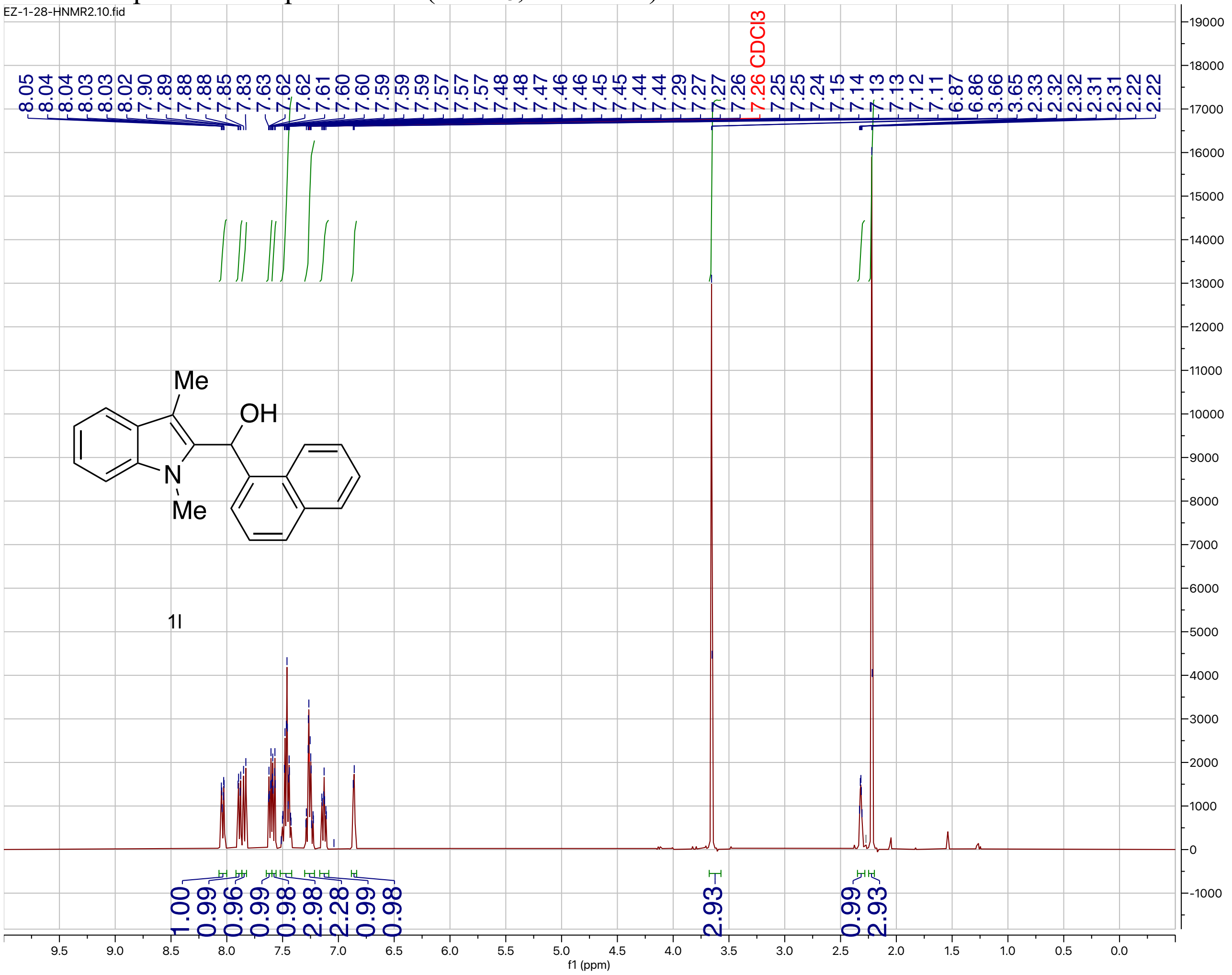

$^{13}C\{^{1}H\}$ NMR spectrum for product **1l** ($CDCl_3$, 100 MHz)

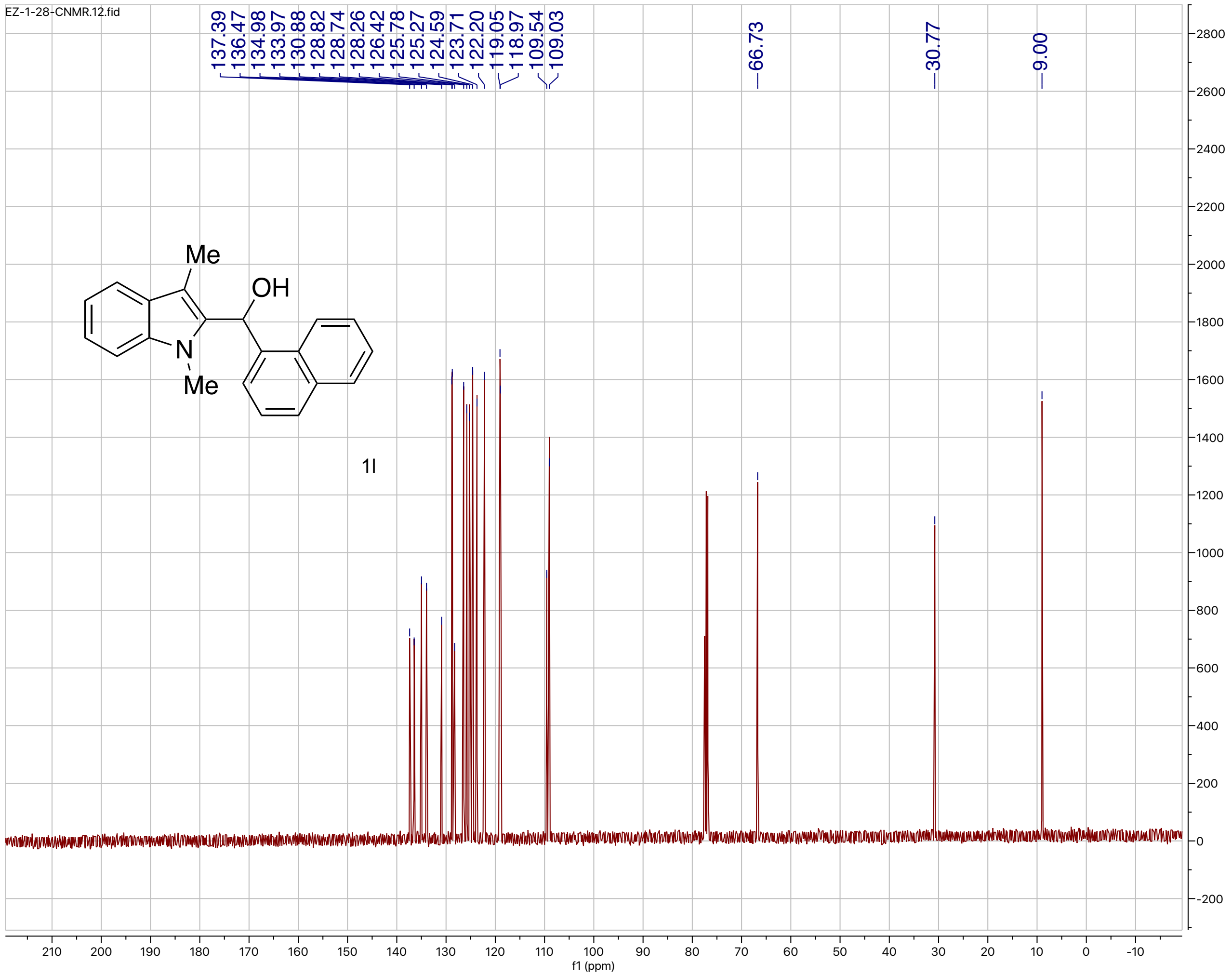

^{1}H NMR spectrum for product **1m** ($CDCl_3$, 500 MHz)

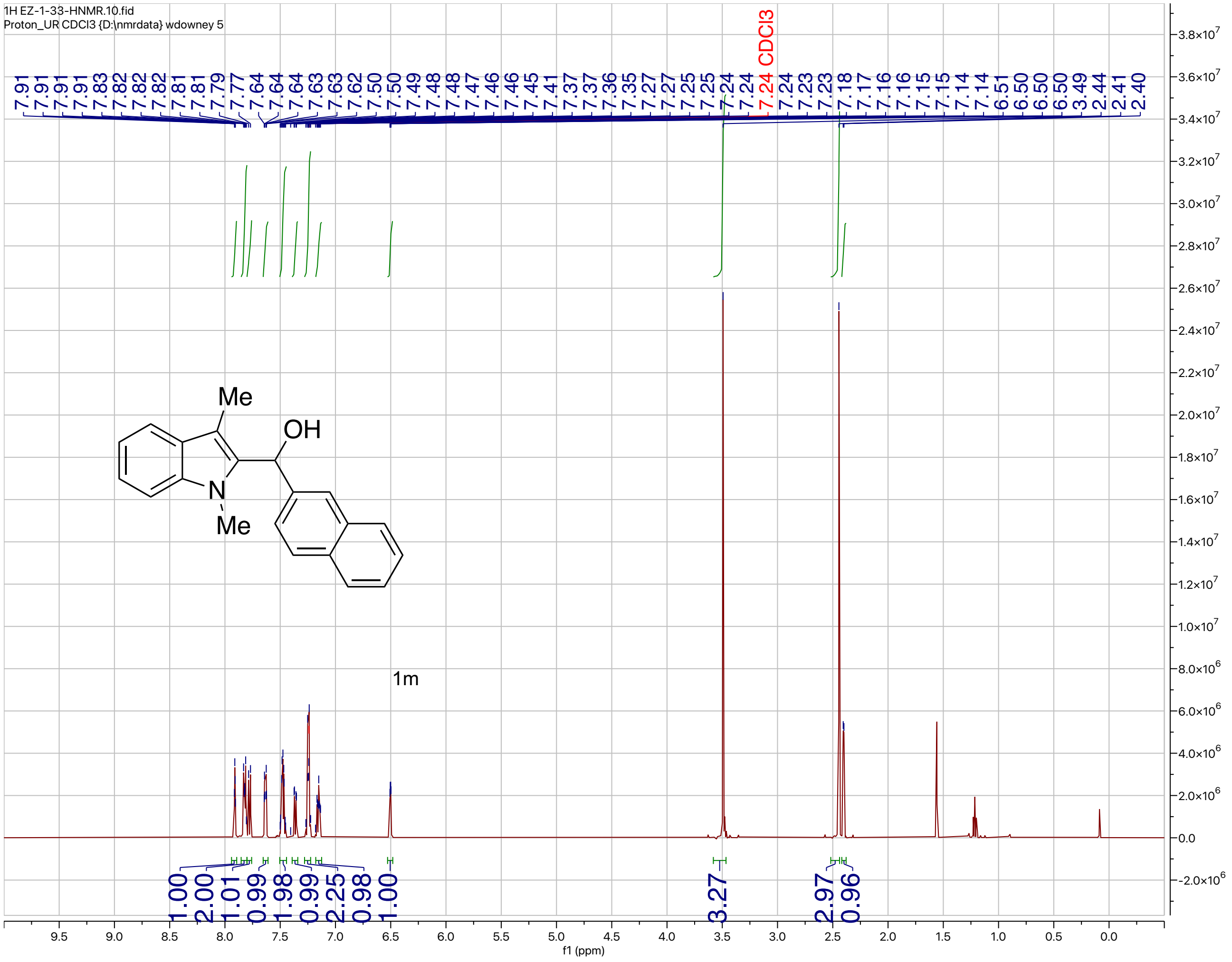

$^{13}C\{^{1}H\}$ NMR spectrum for product **1m** ($CDCl_3$, 126 MHz)

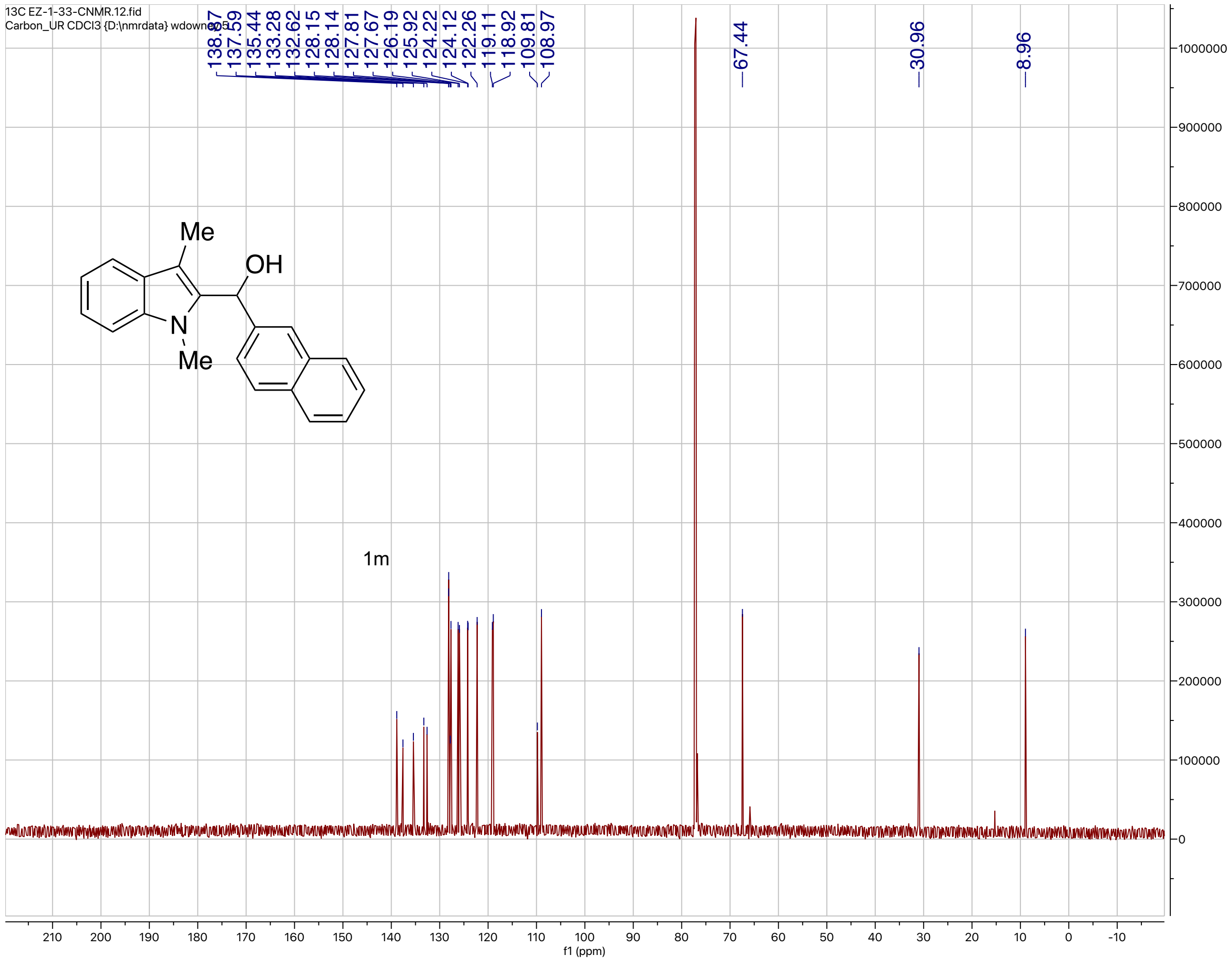

^{1}H NMR spectrum for product **1n** ($CDCl_3$, 500 MHz)

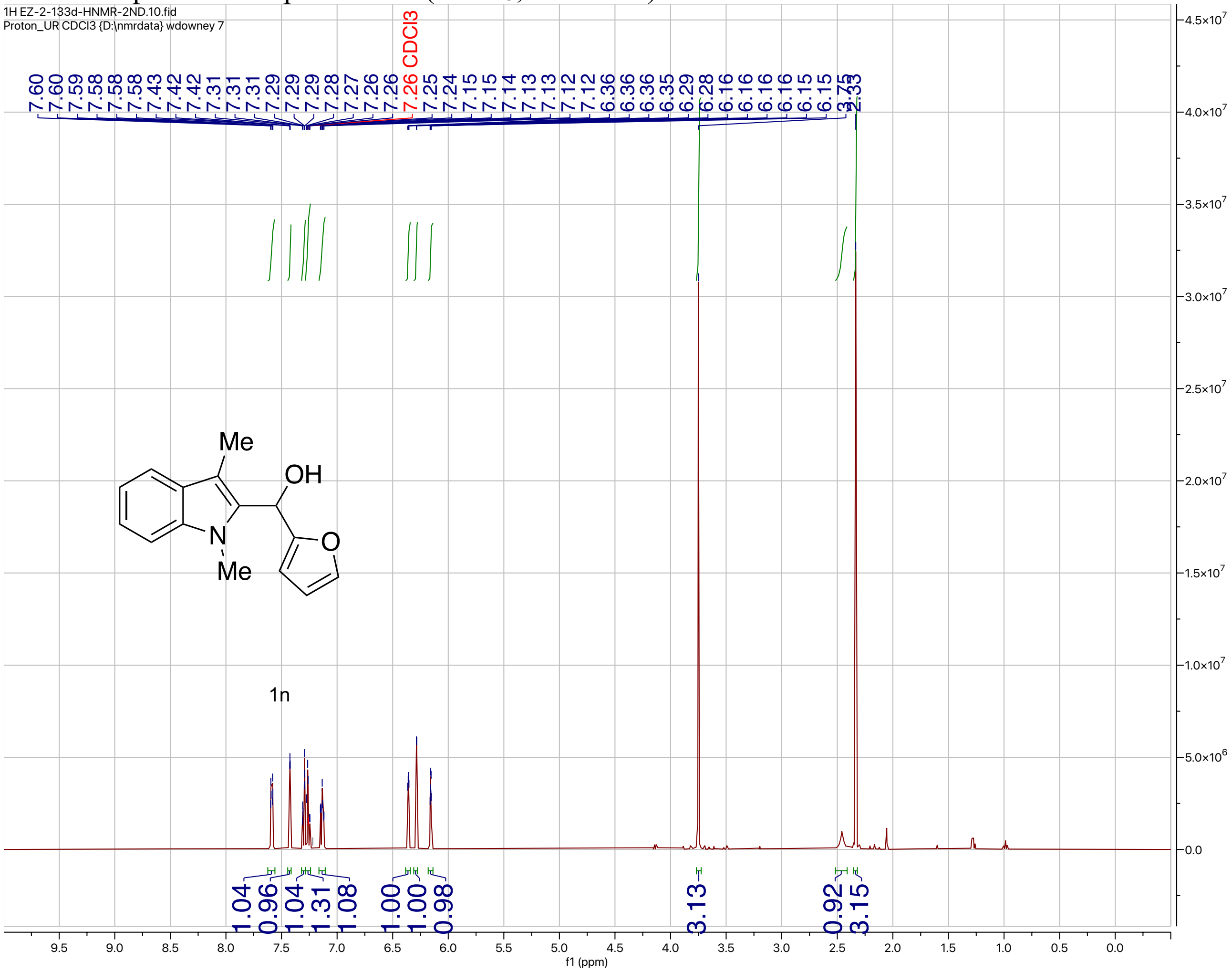

$^{13}C\{^1H\}$ NMR spectrum for product **1n** ($CDCl_3$, 126 MHz)

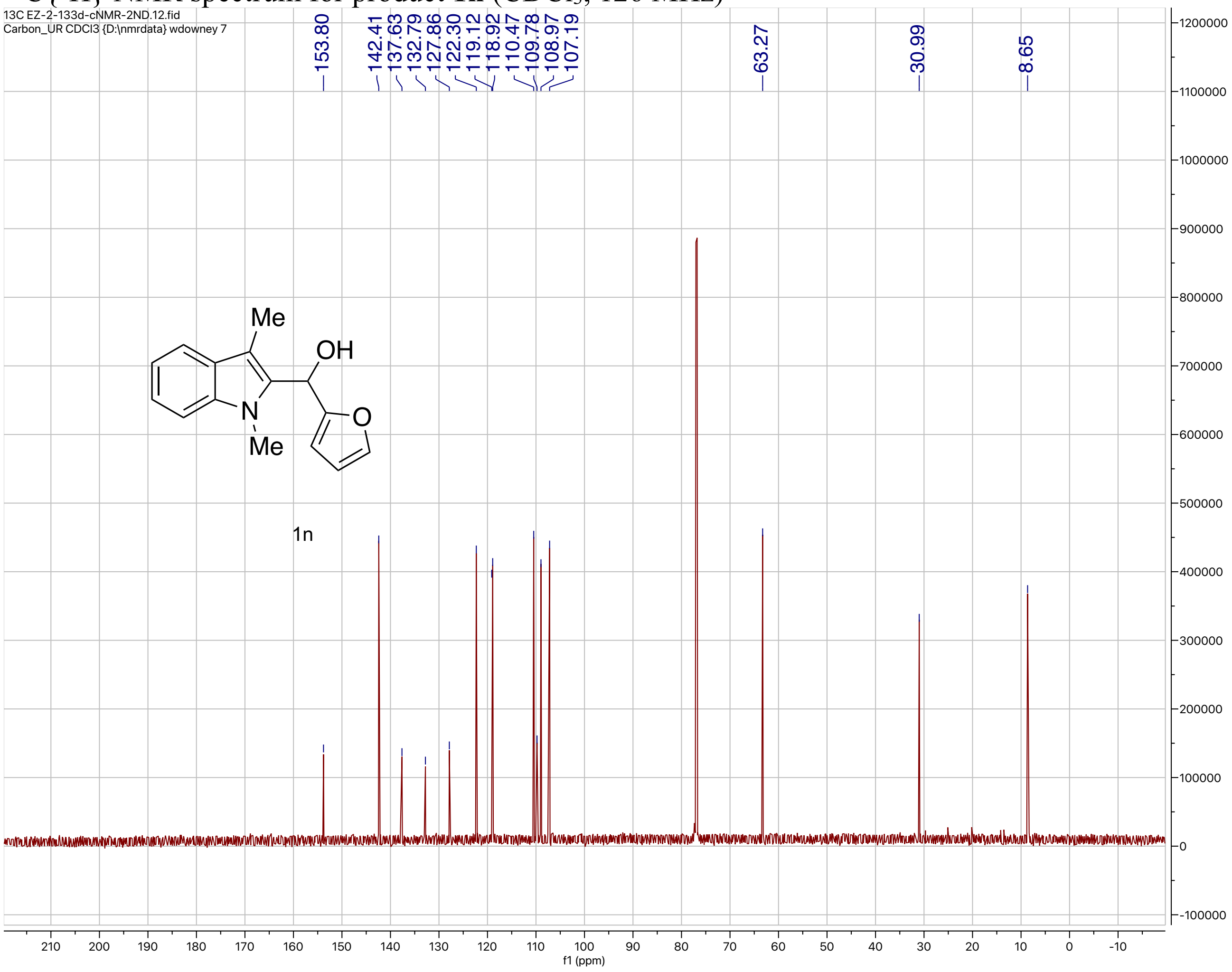

^{1}H NMR spectrum for product **1o** ($CDCl_3$, 500 MHz)

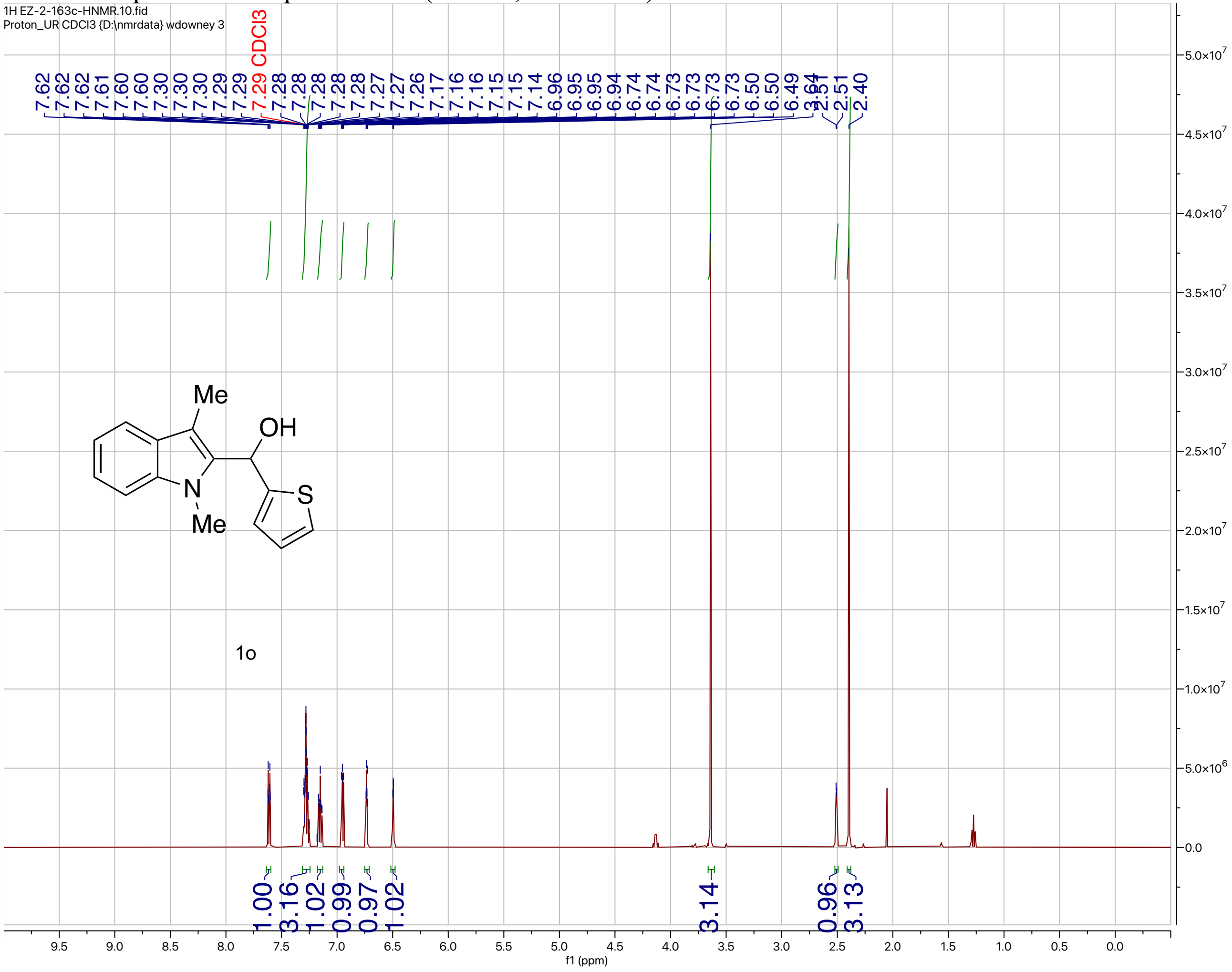

$^{13}C\{^{1}H\}$ NMR spectrum for product **1o** ($CDCl_3$, 126 MHz)

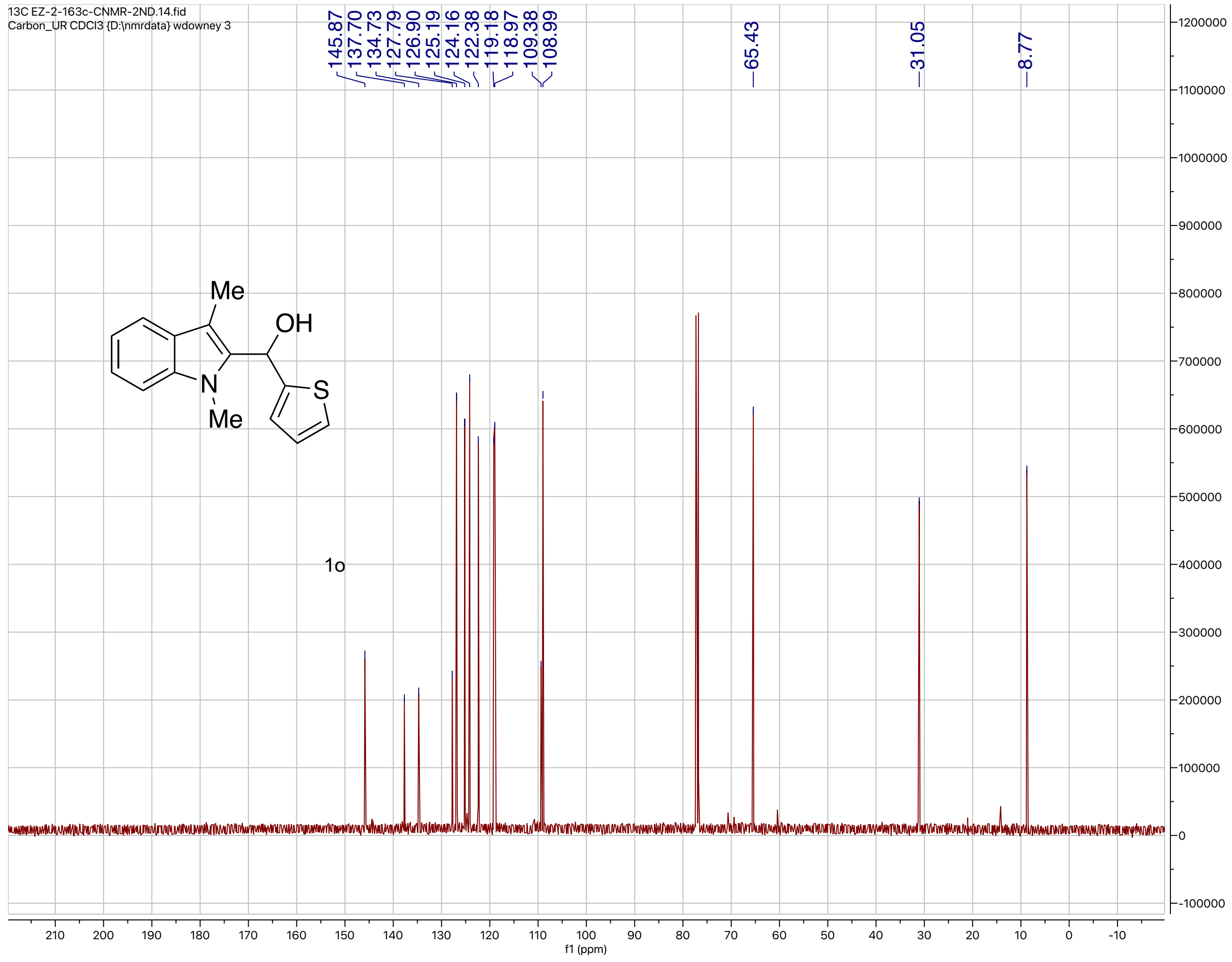

^{1}H NMR spectrum for product **1p** ($CDCl_3$, 500 MHz)

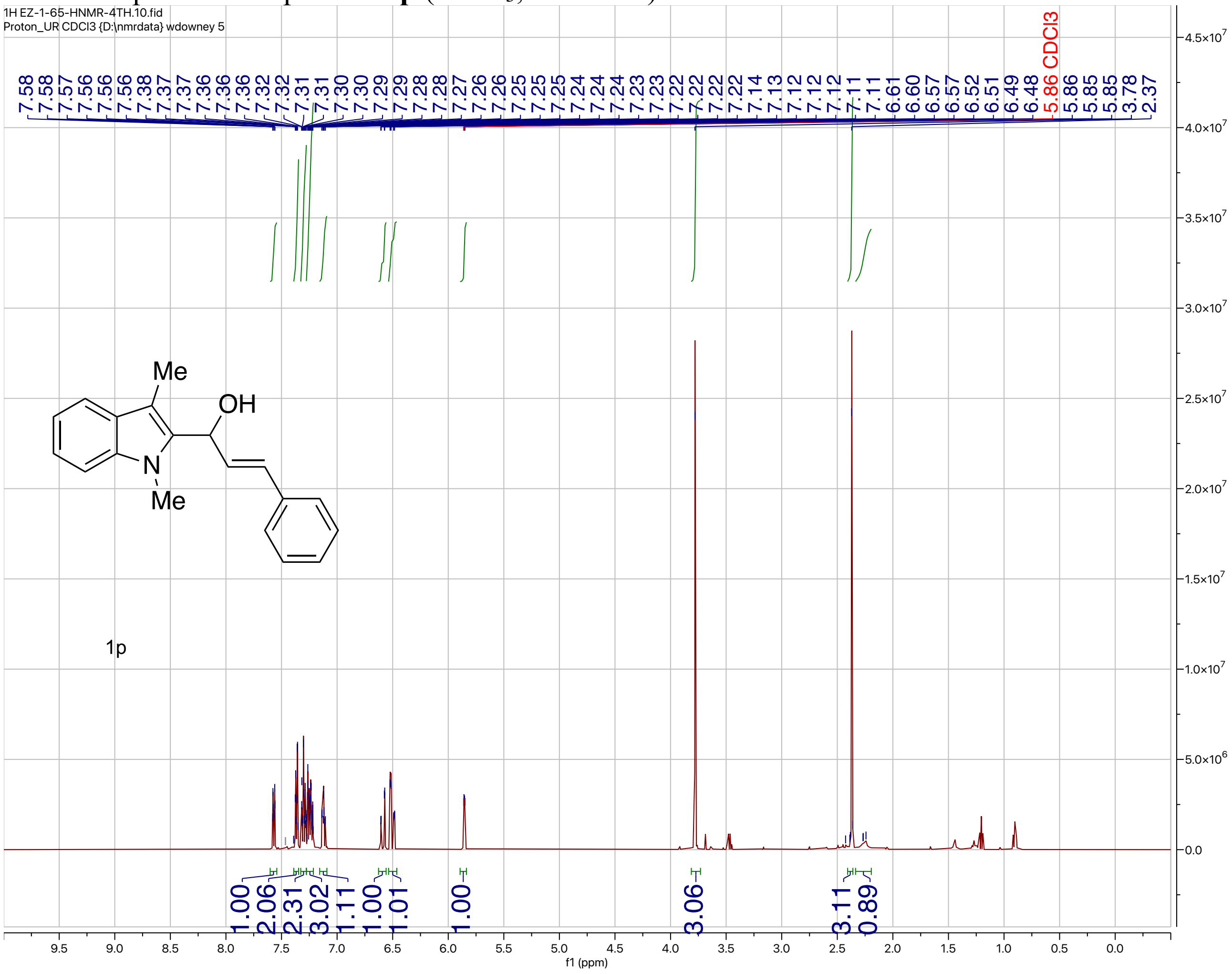

$^{13}C\{^{1}H\}$ NMR spectrum for product **1p** ($CDCl_3$, 126 MHz)

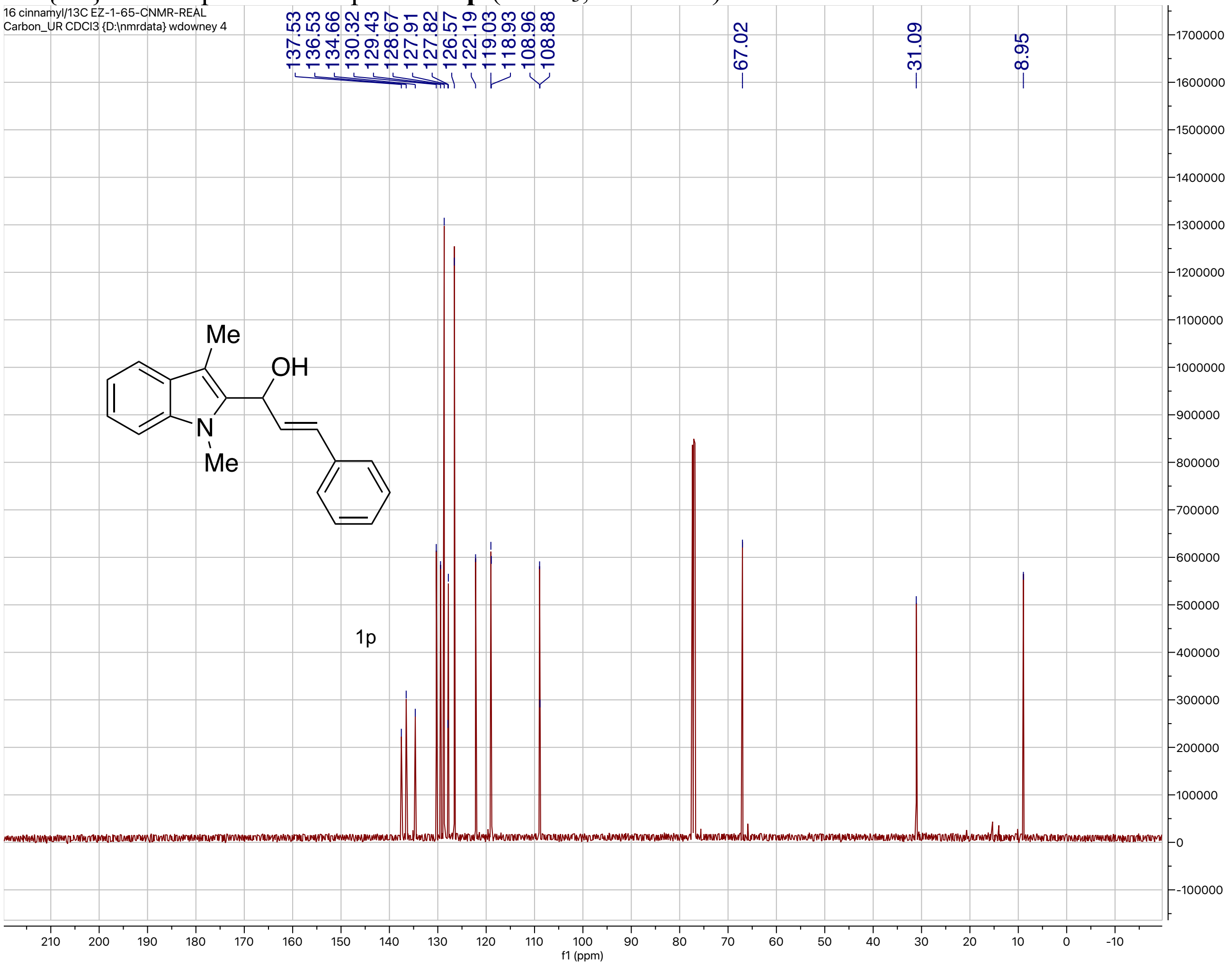

^{1}H NMR spectrum for product **3a** ($CDCl_3$, 500 MHz)

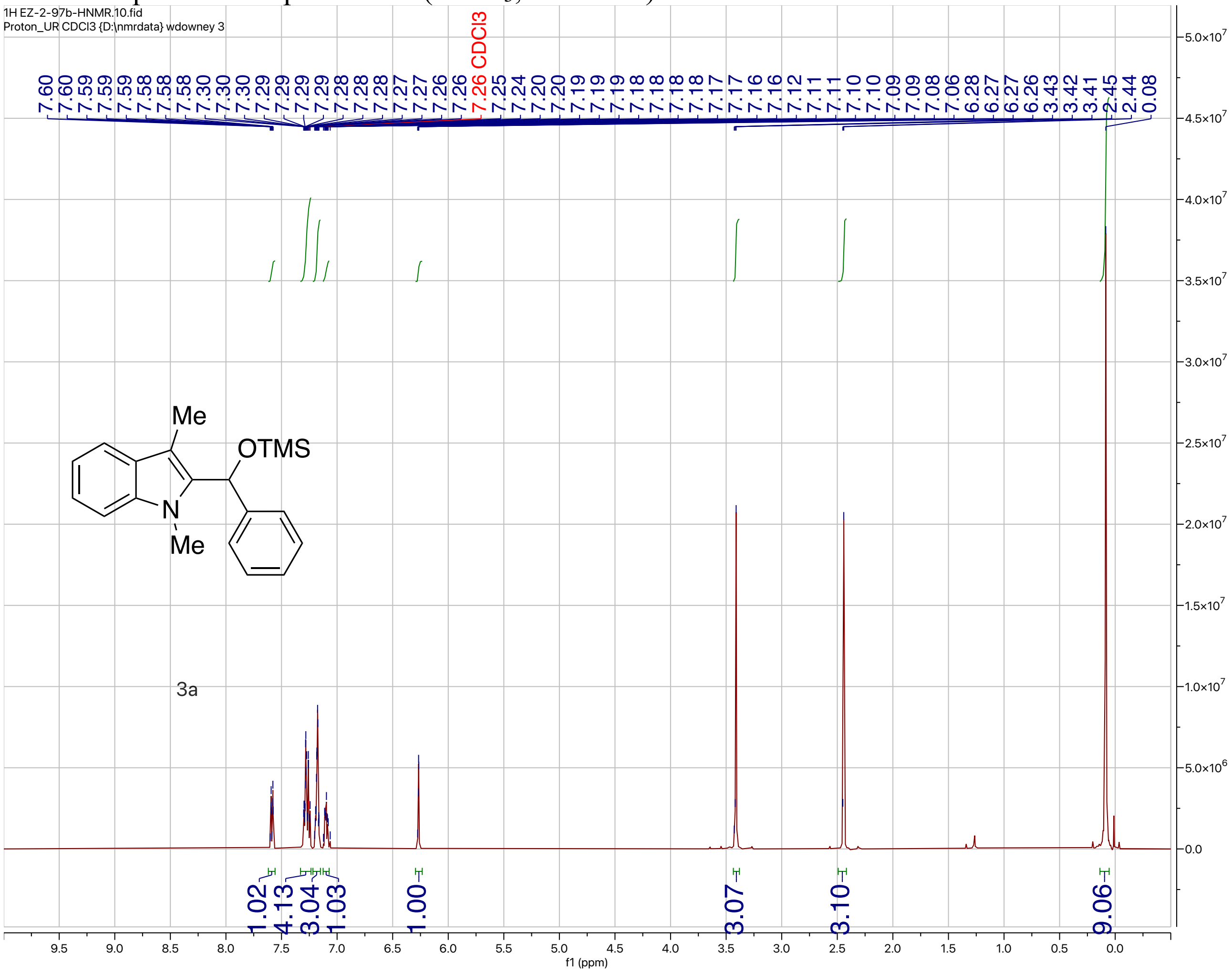

$^{13}C\{^{1}H\}$ NMR spectrum for product **3a** ($CDCl_3$, 126 MHz)

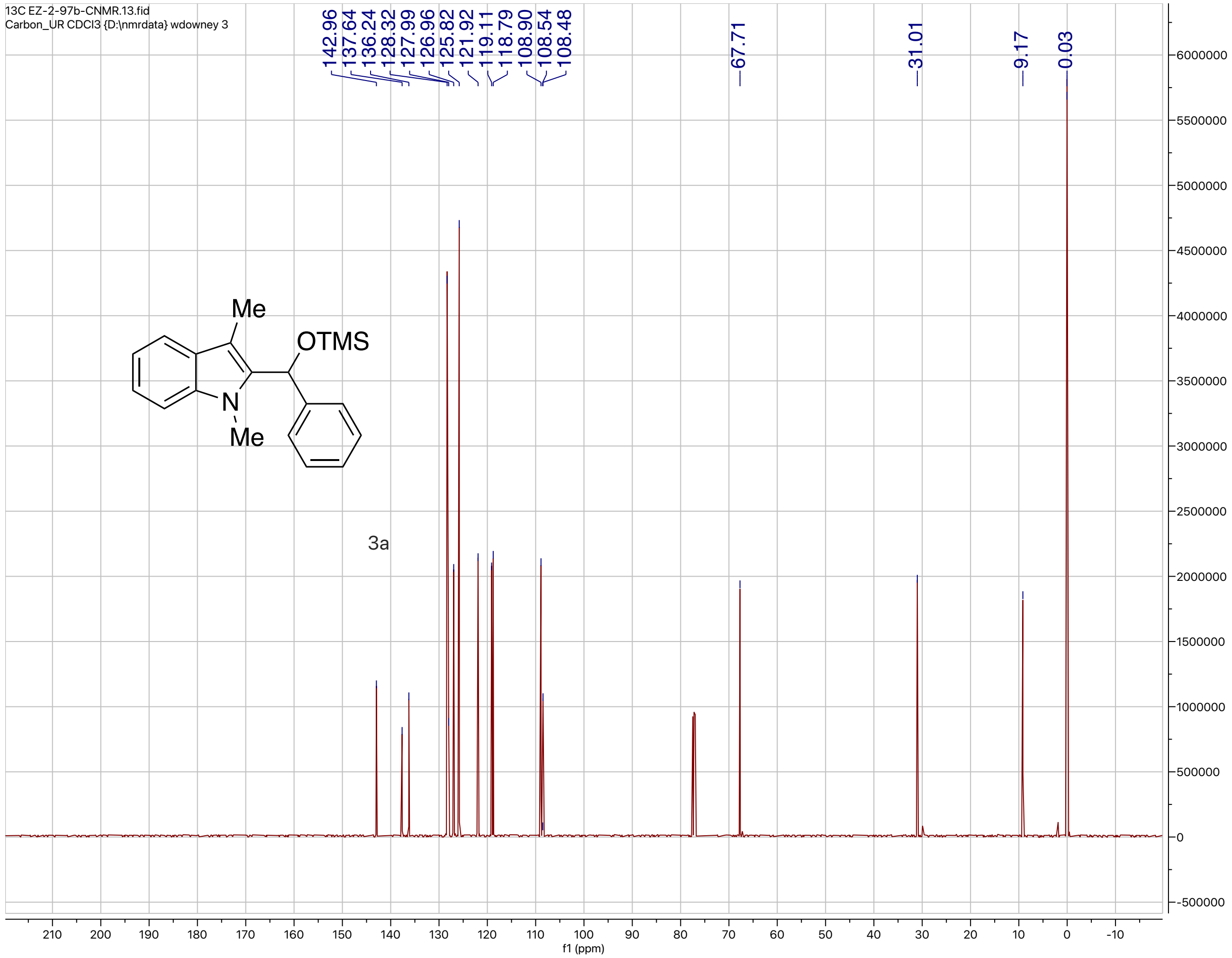

^{1}H NMR spectrum for product **3b** ($CDCl_3$, 500 MHz)

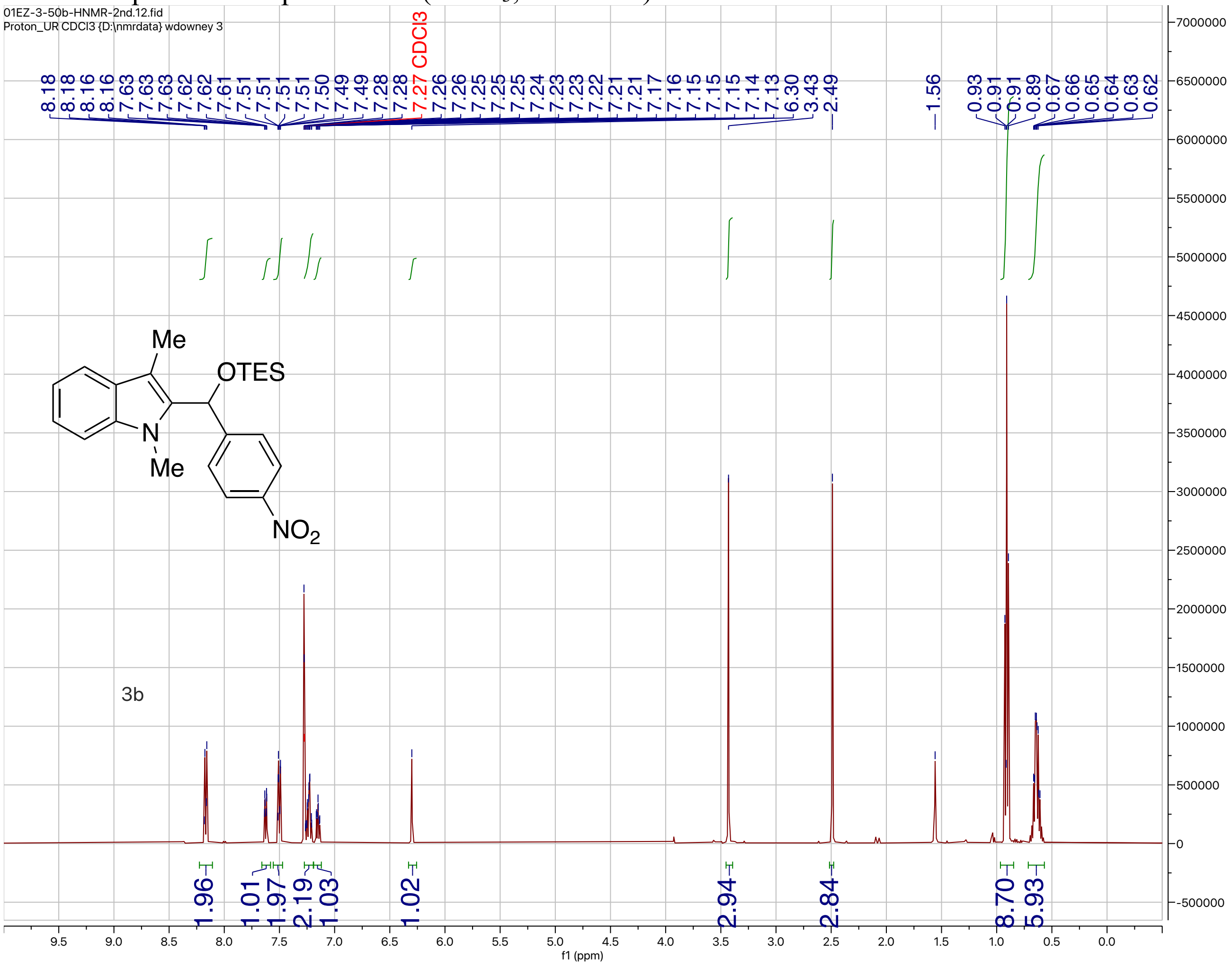

$^{13}C\{^{1}H\}$ NMR spectrum for product **3b** ($CDCl_3$, 126 MHz)

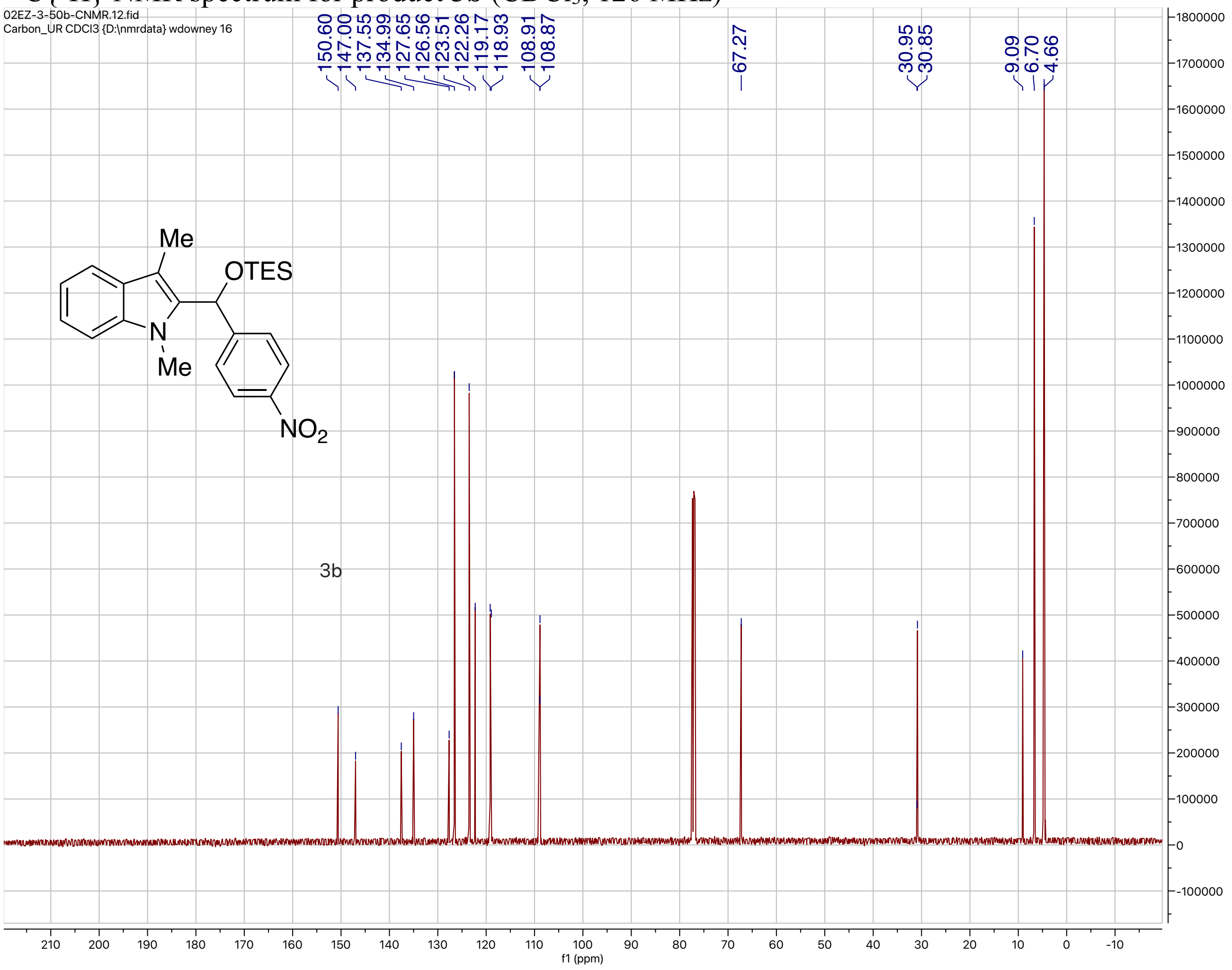

^{1}H NMR spectrum for product **3d** ($CDCl_3$, 500 MHz)

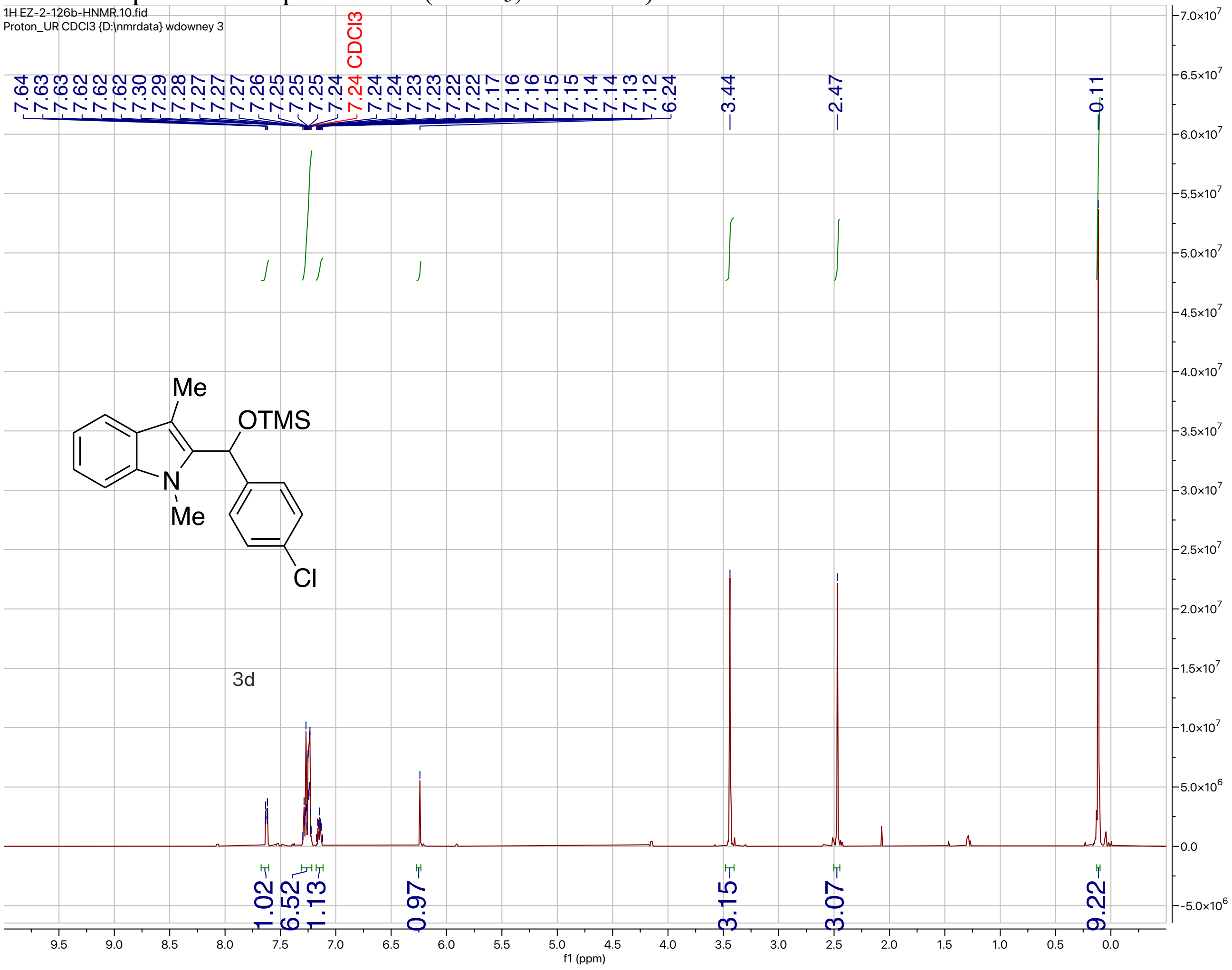

$^{13}C\{^1H\}$ NMR spectrum for product **3d** ($CDCl_3$, 126 MHz)

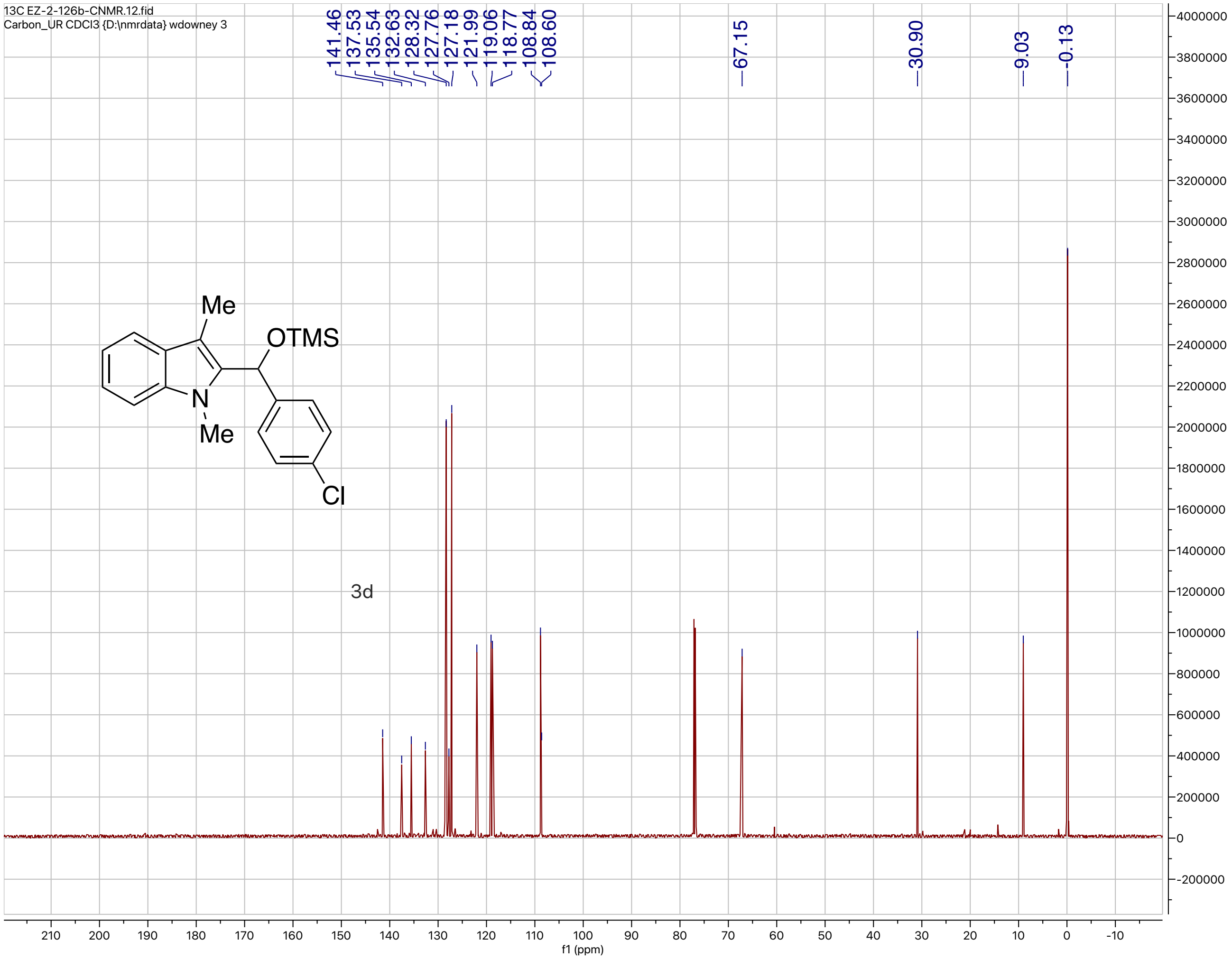

^{1}H NMR spectrum for product **3e** ($CDCl_3$, 500 MHz)

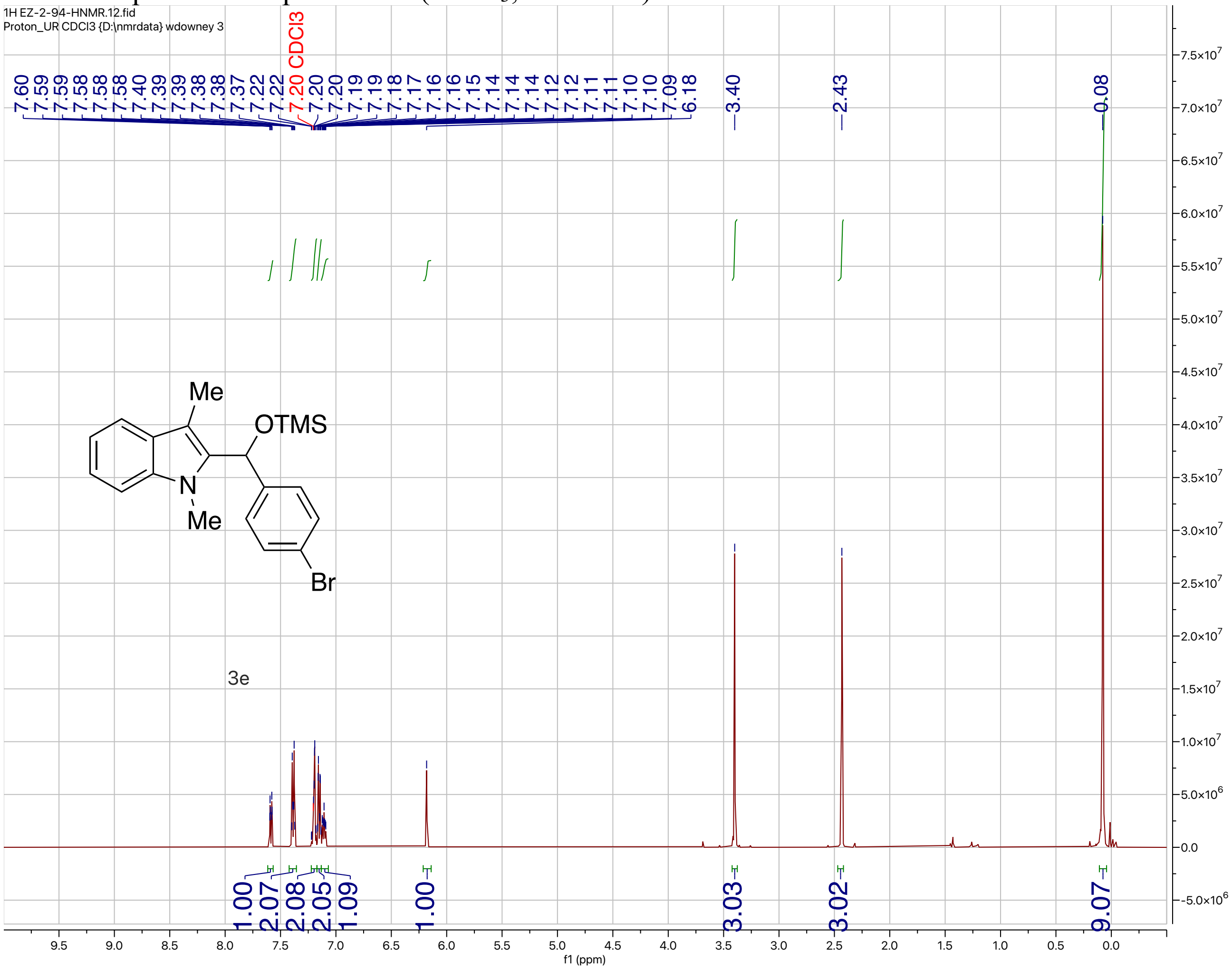

$^{13}C\{^{1}H\}$ NMR spectrum for product **3e** ($CDCl_3$, 126 MHz)

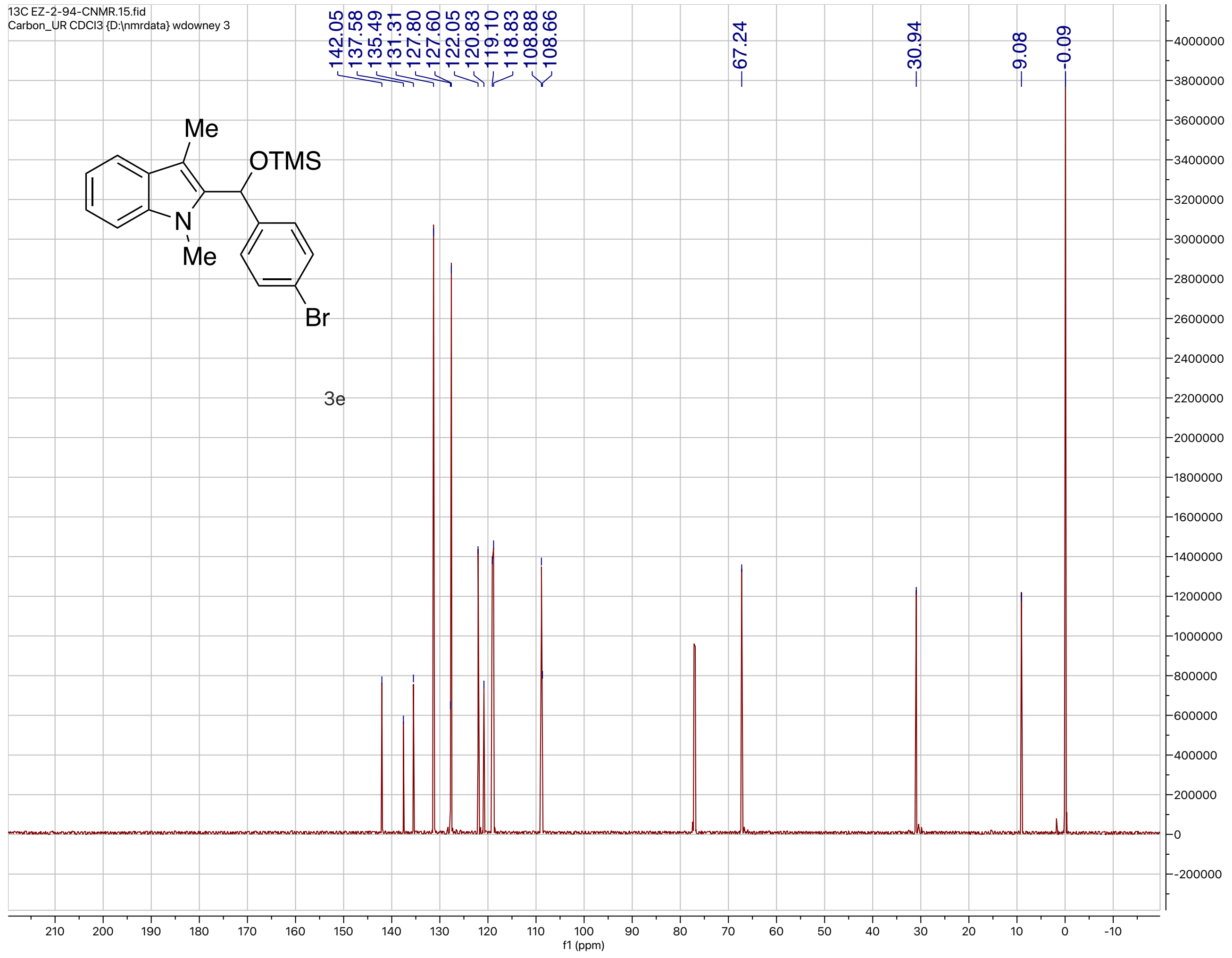

^{1}H NMR spectrum for product **3f** ($CDCl_3$, 500 MHz)

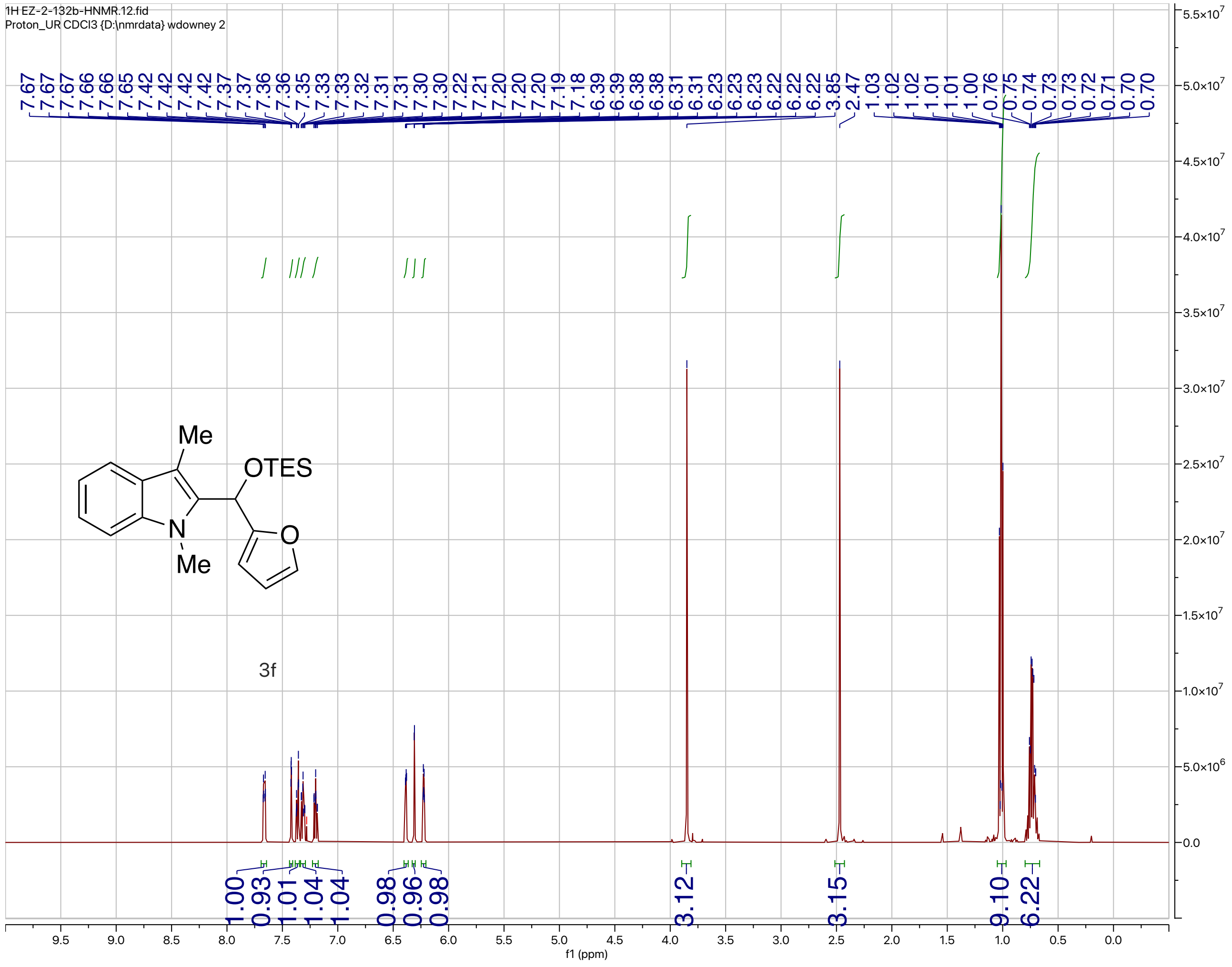

$^{13}C\{^{1}H\}$ NMR spectrum for product **3f** ($CDCl_3$, 126 MHz)

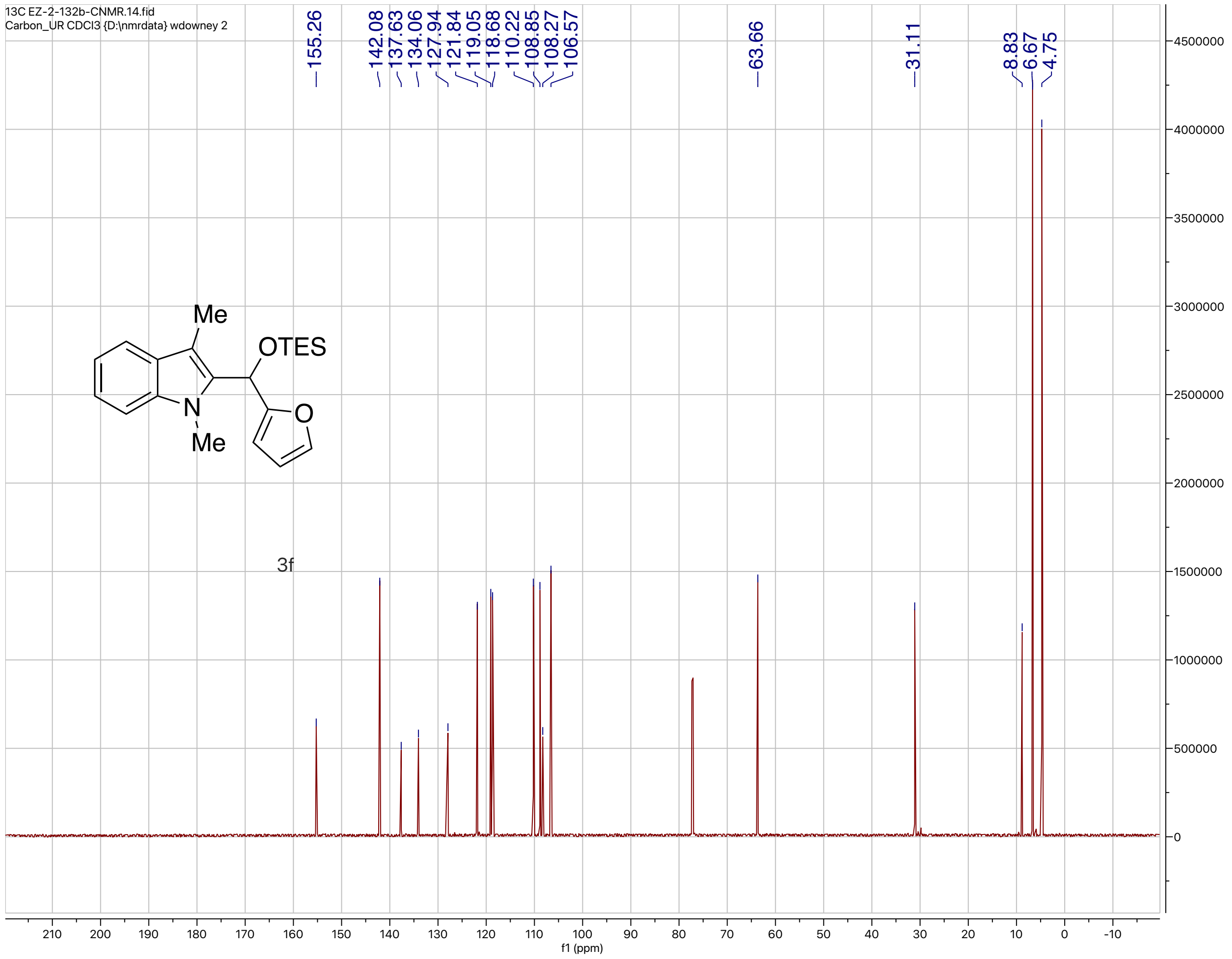

^{1}H NMR spectrum for product **4a** ($CDCl_3$, 500 MHz)

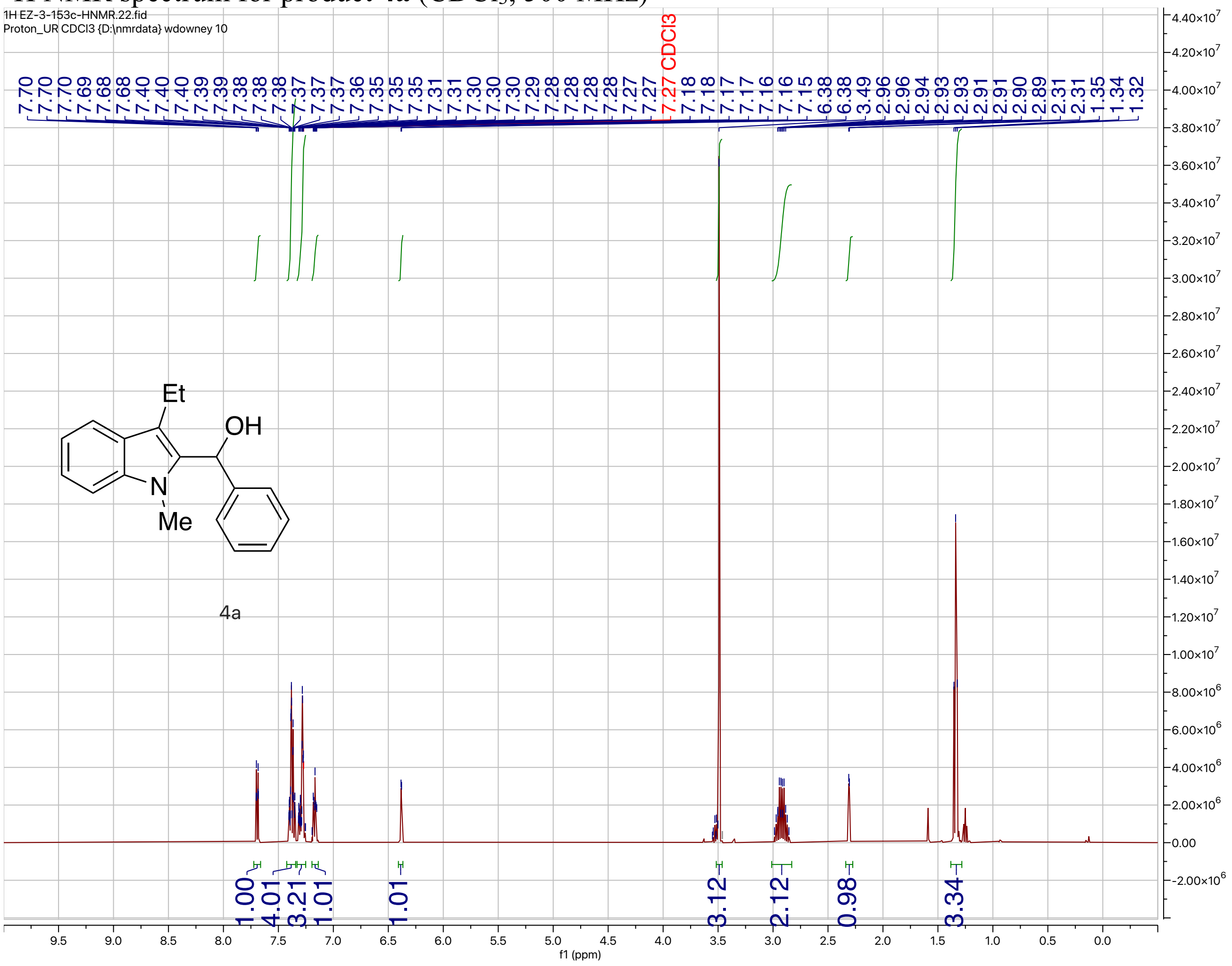

$^{13}C\{^{1}H\}$ NMR spectrum for product **4a** ($CDCl_3$, 126 MHz)

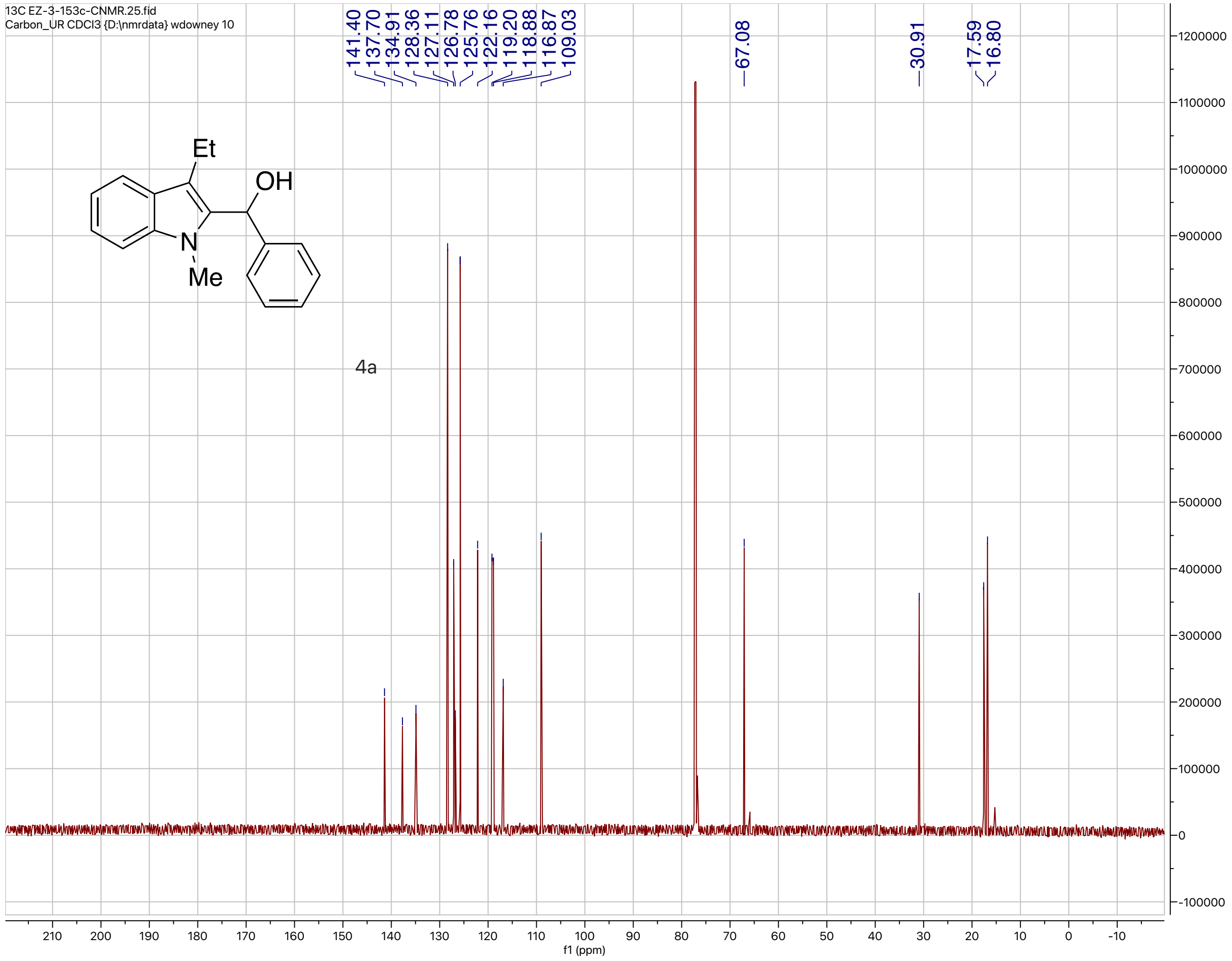

^{1}H NMR spectrum for product **4b** ($CDCl_3$, 500 MHz)

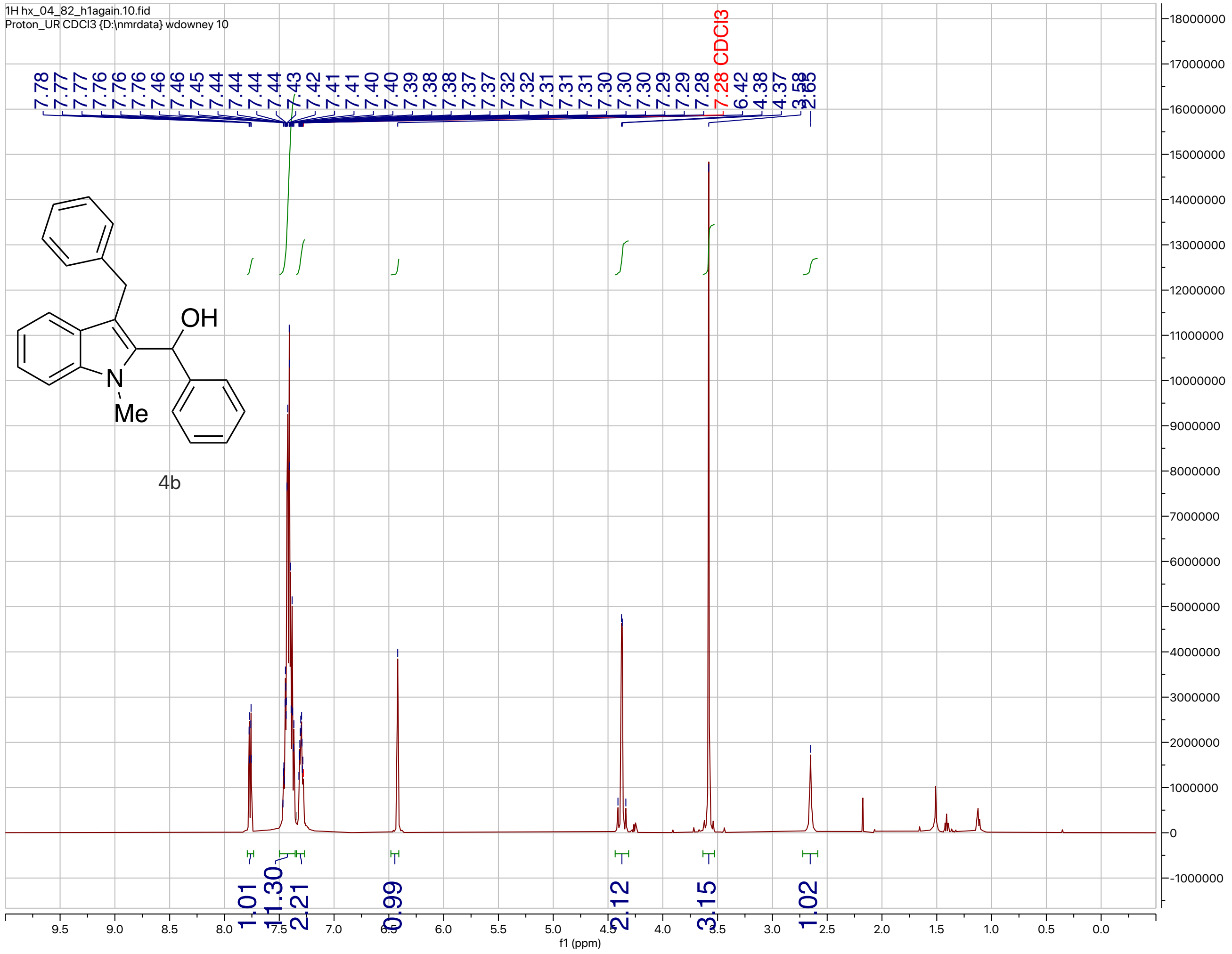

$^{13}C\{^{1}H\}$ NMR spectrum for product **4b** ($CDCl_3$, 126 MHz)

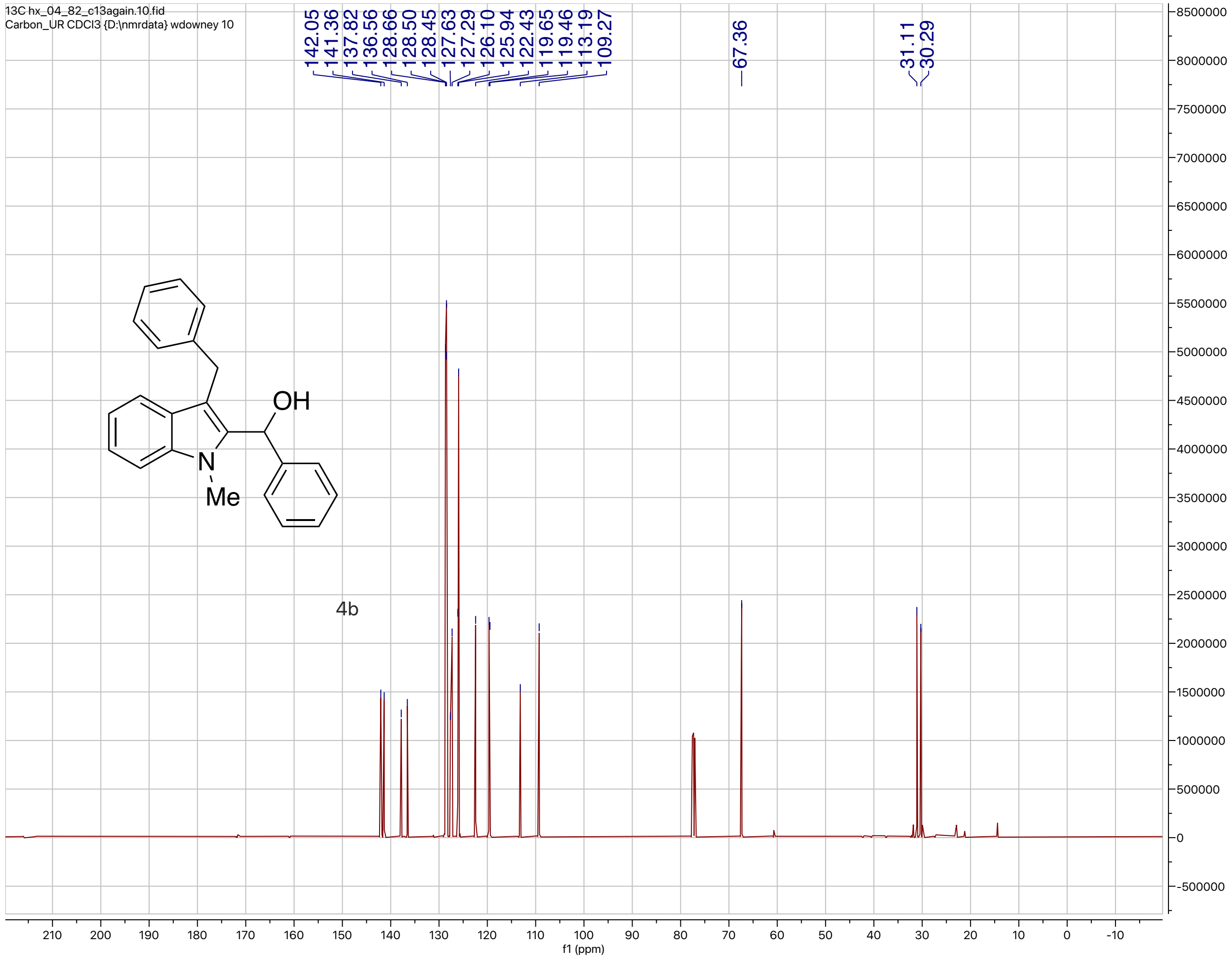

^{1}H NMR spectrum for product **4c** ($CDCl_3$, 500 MHz)

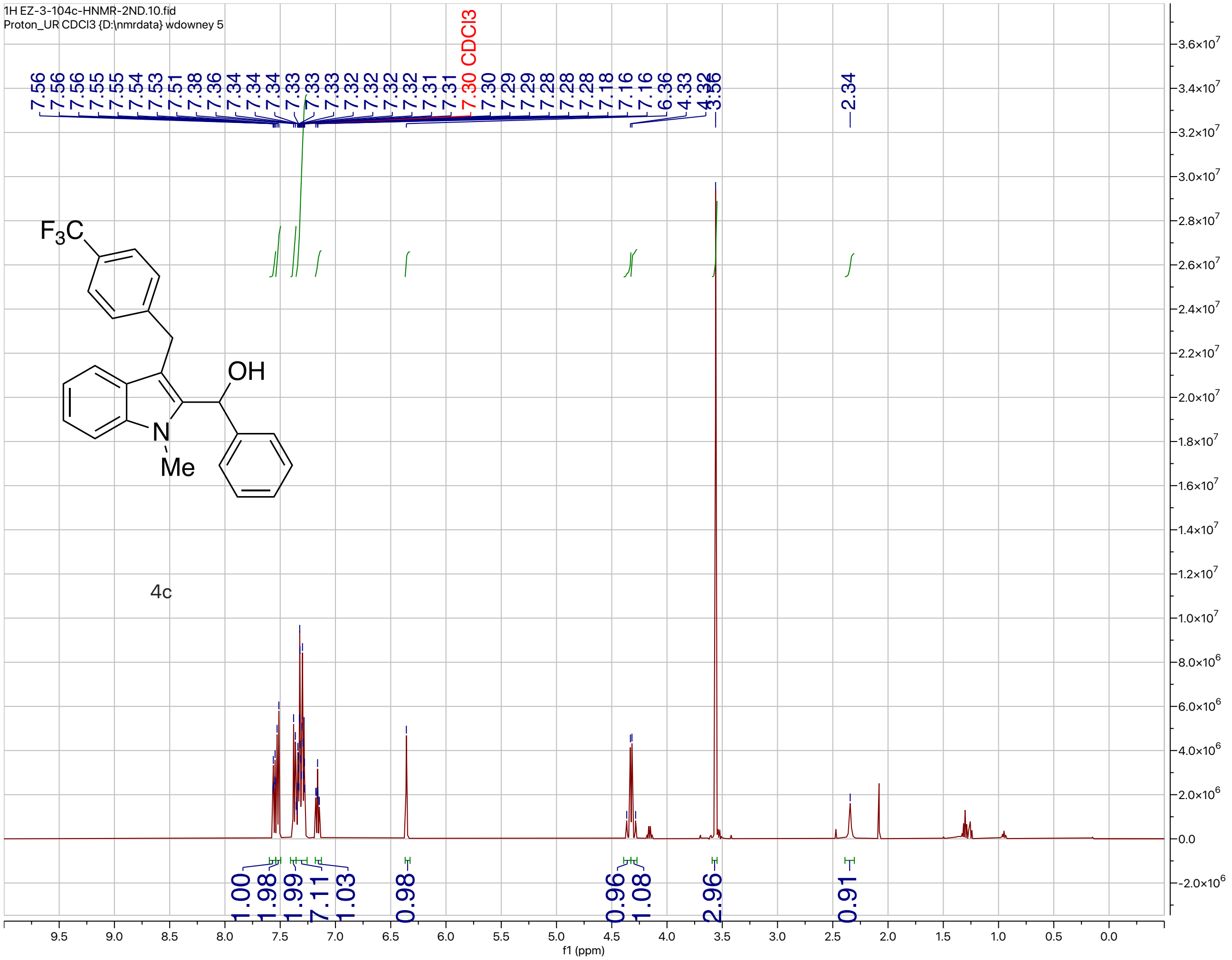

$^{13}C\{^{1}H\}$ NMR spectrum for product **4c** ($CDCl_3$, 126 MHz)

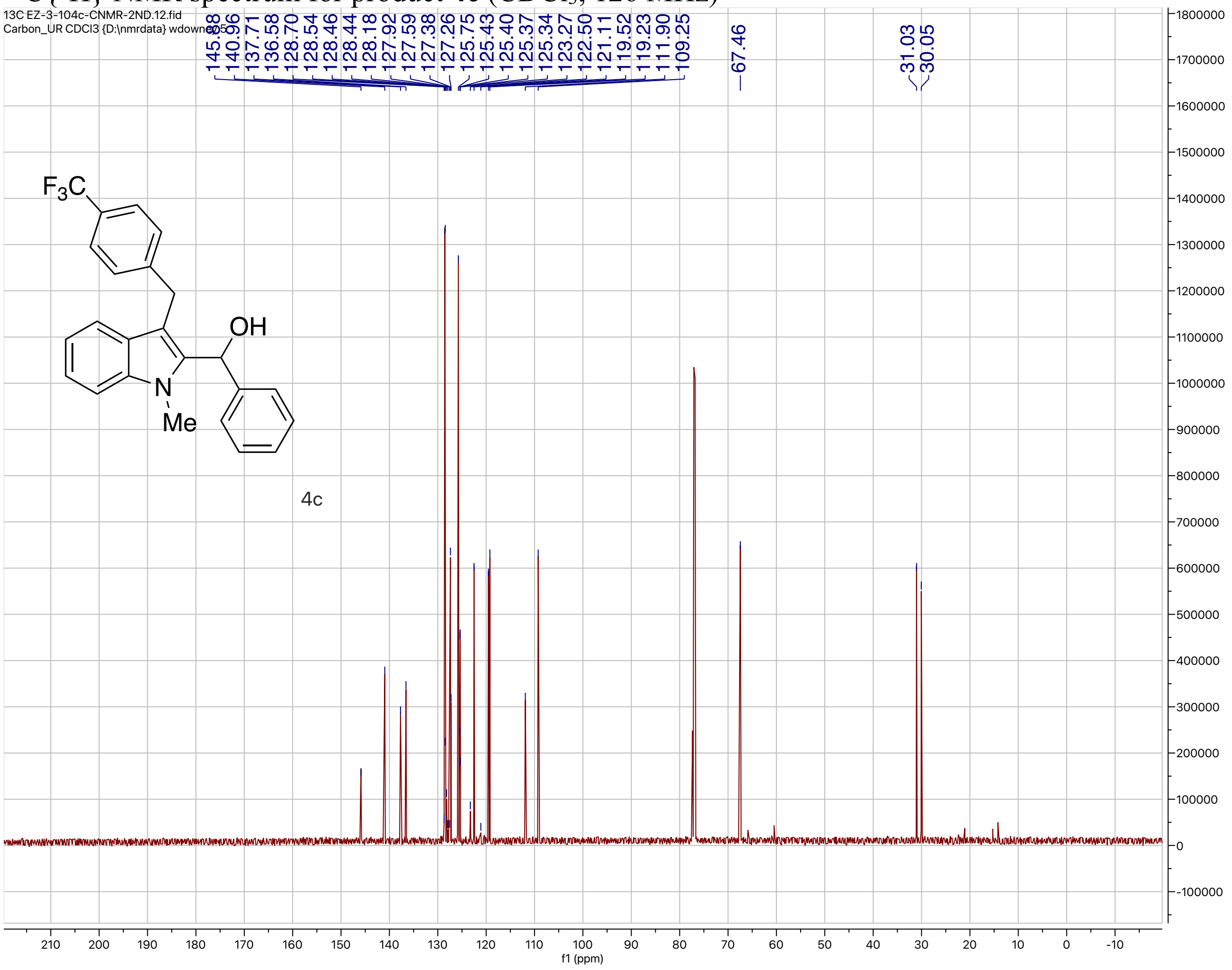

^{1}H NMR spectrum for product **4d** ($CDCl_3$, 500 MHz)

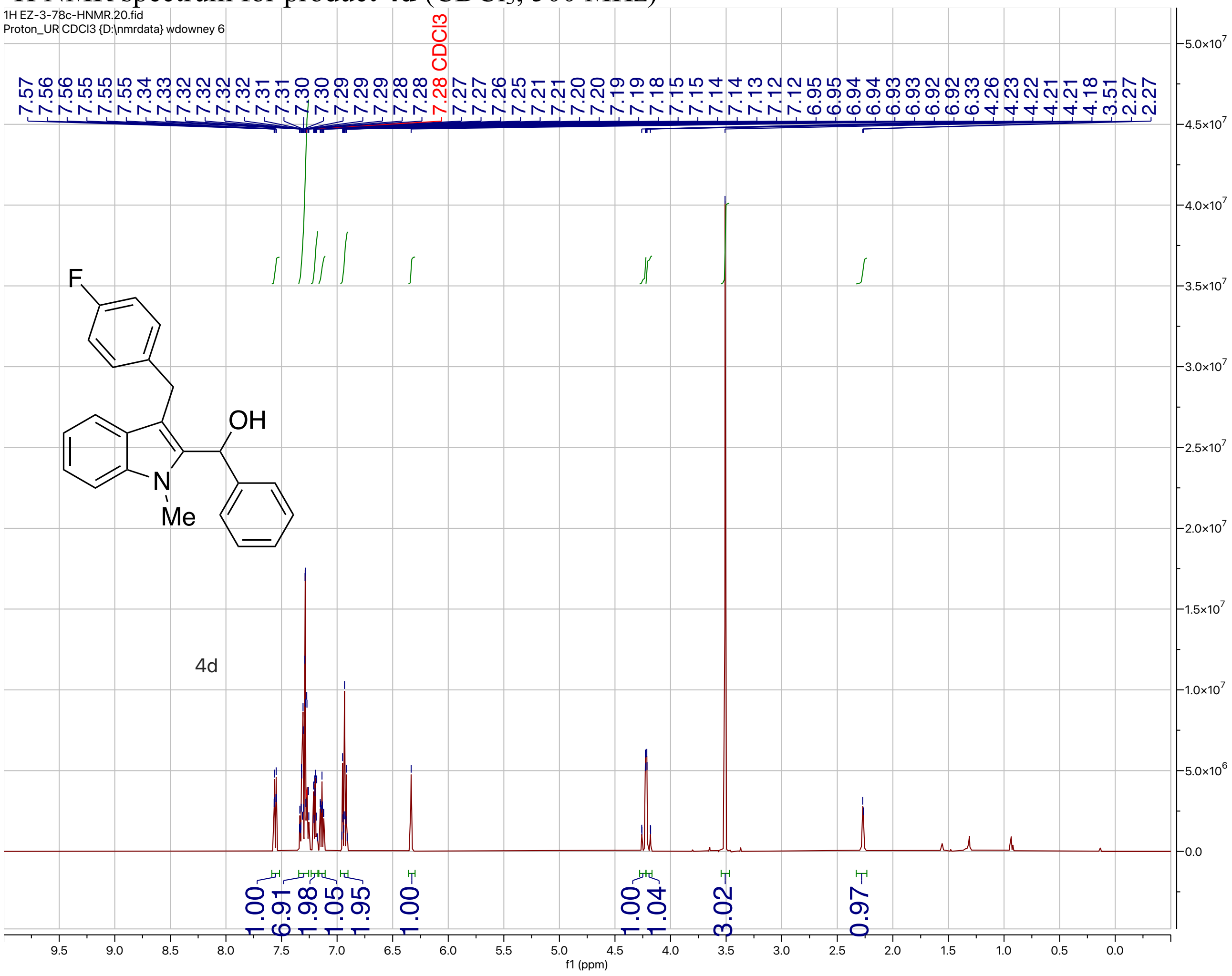

$^{13}C\{^{1}H\}$ NMR spectrum for product **4d** ($CDCl_3$, 126 MHz)

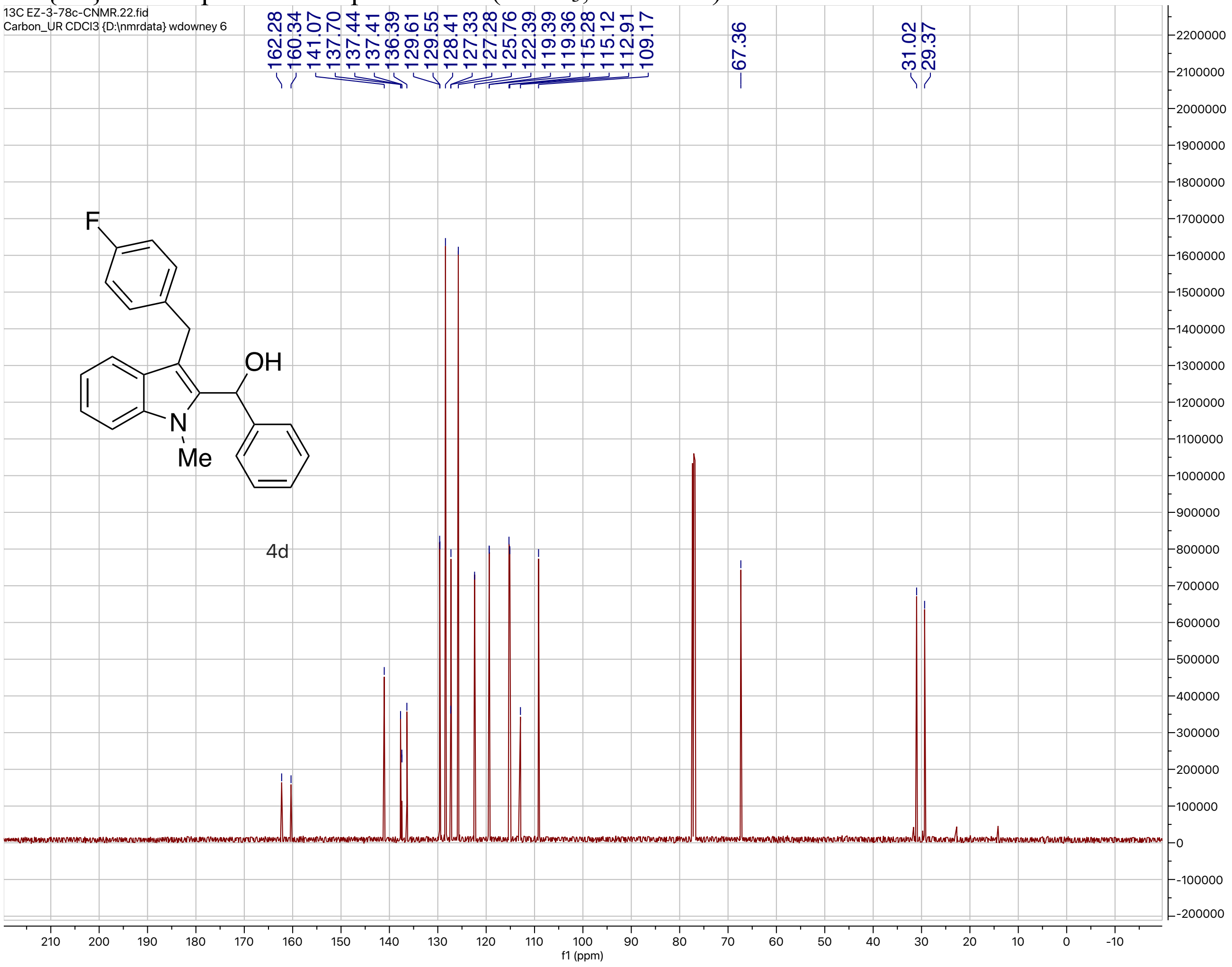

^{1}H NMR spectrum for product **4e** ($CDCl_3$, 500 MHz)

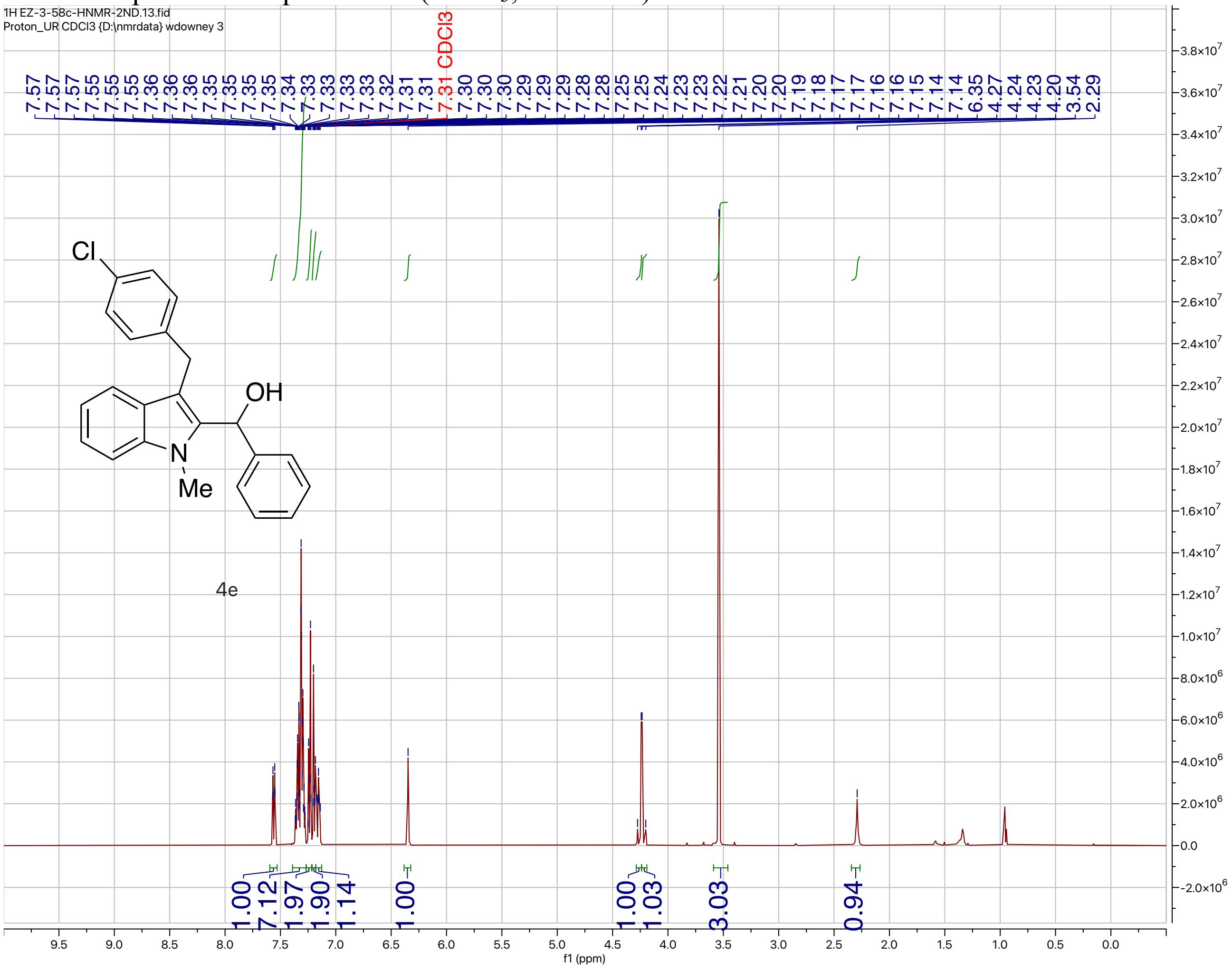

$^{13}C\{^{1}H\}$ NMR spectrum for product **4e** ($CDCl_3$, 126 MHz)

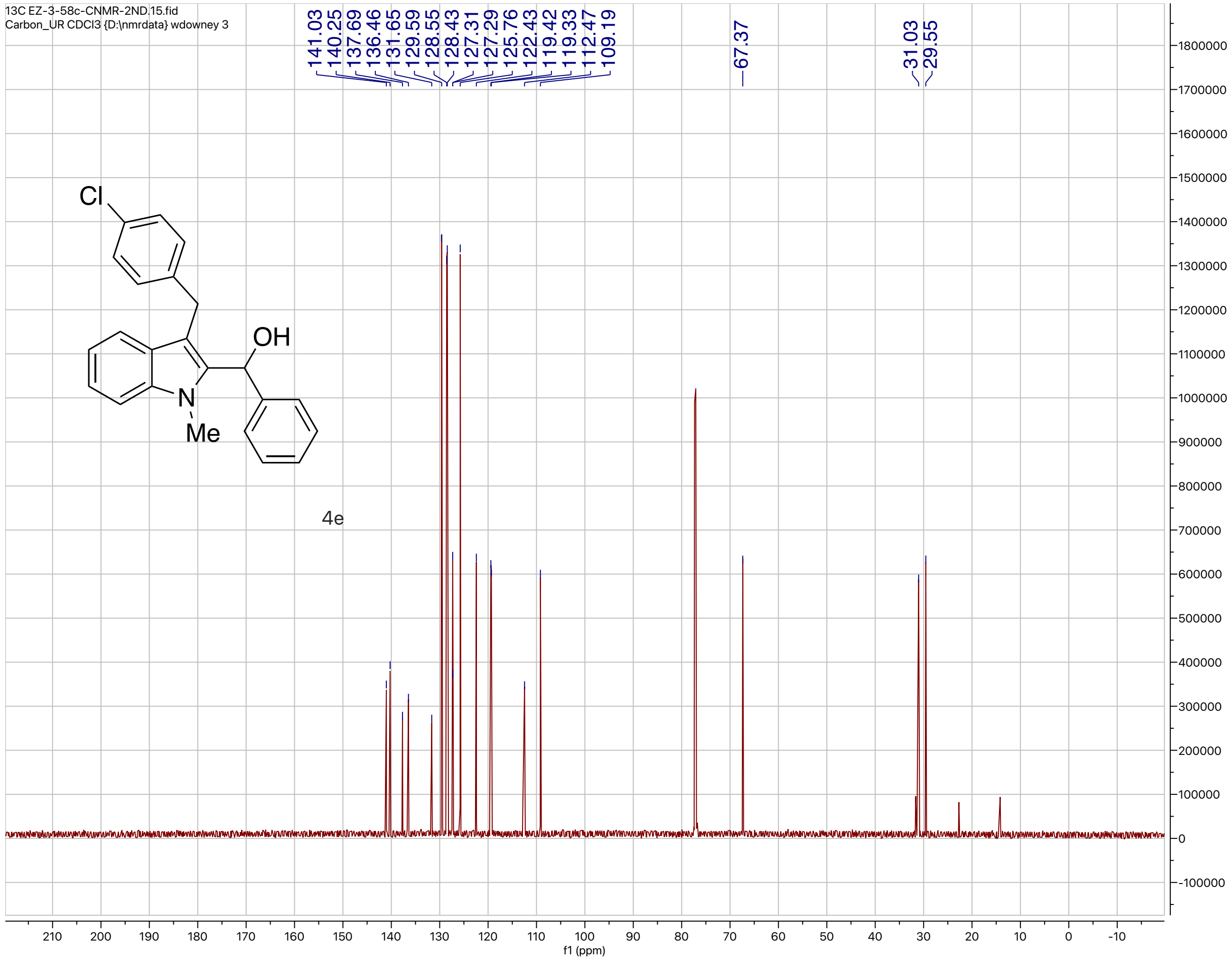

^{1}H NMR spectrum for product **4f** ($CDCl_3$, 500 MHz)

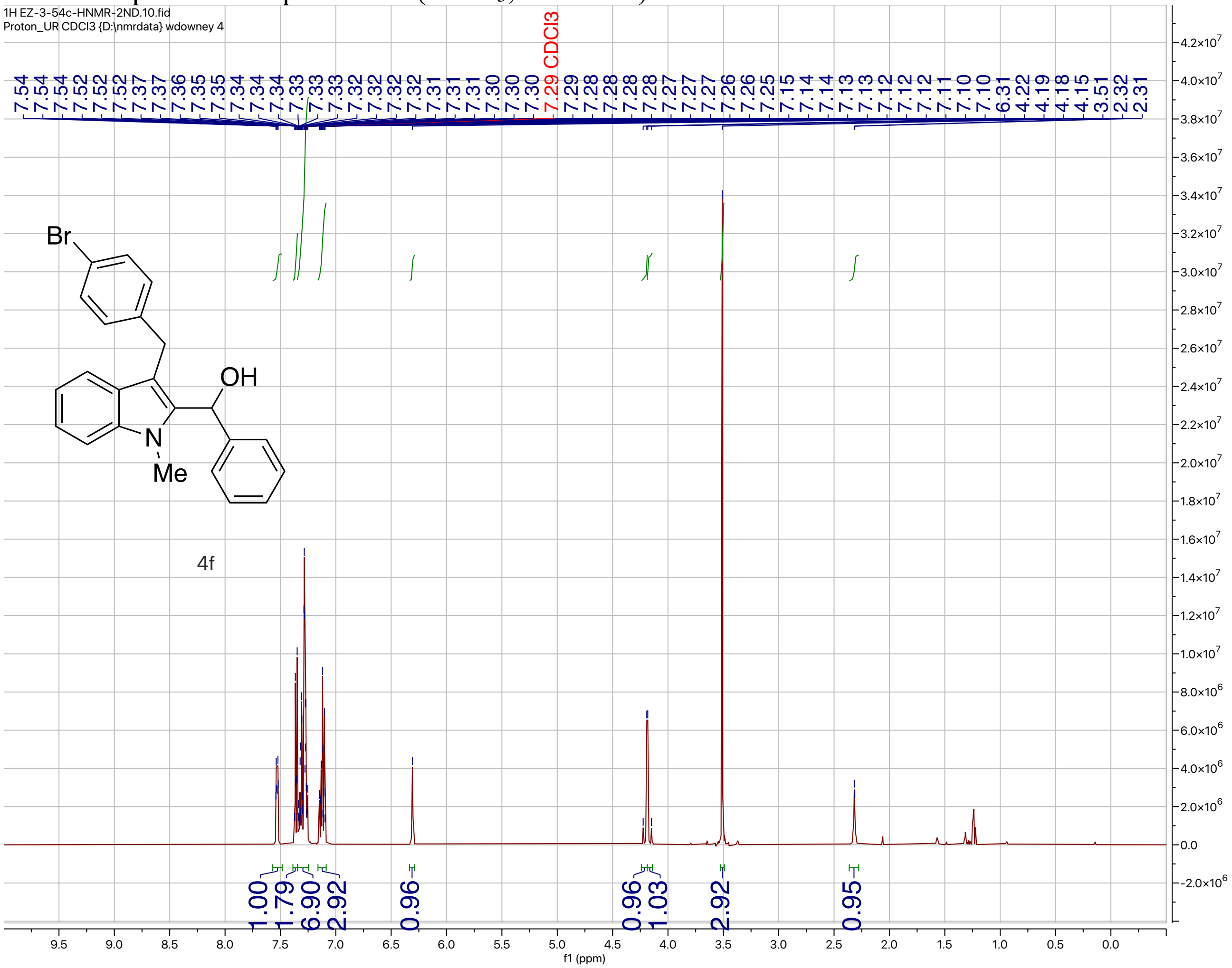

$^{13}C\{^{1}H\}$ NMR spectrum for product **4f** ($CDCl_3$, 126 MHz)

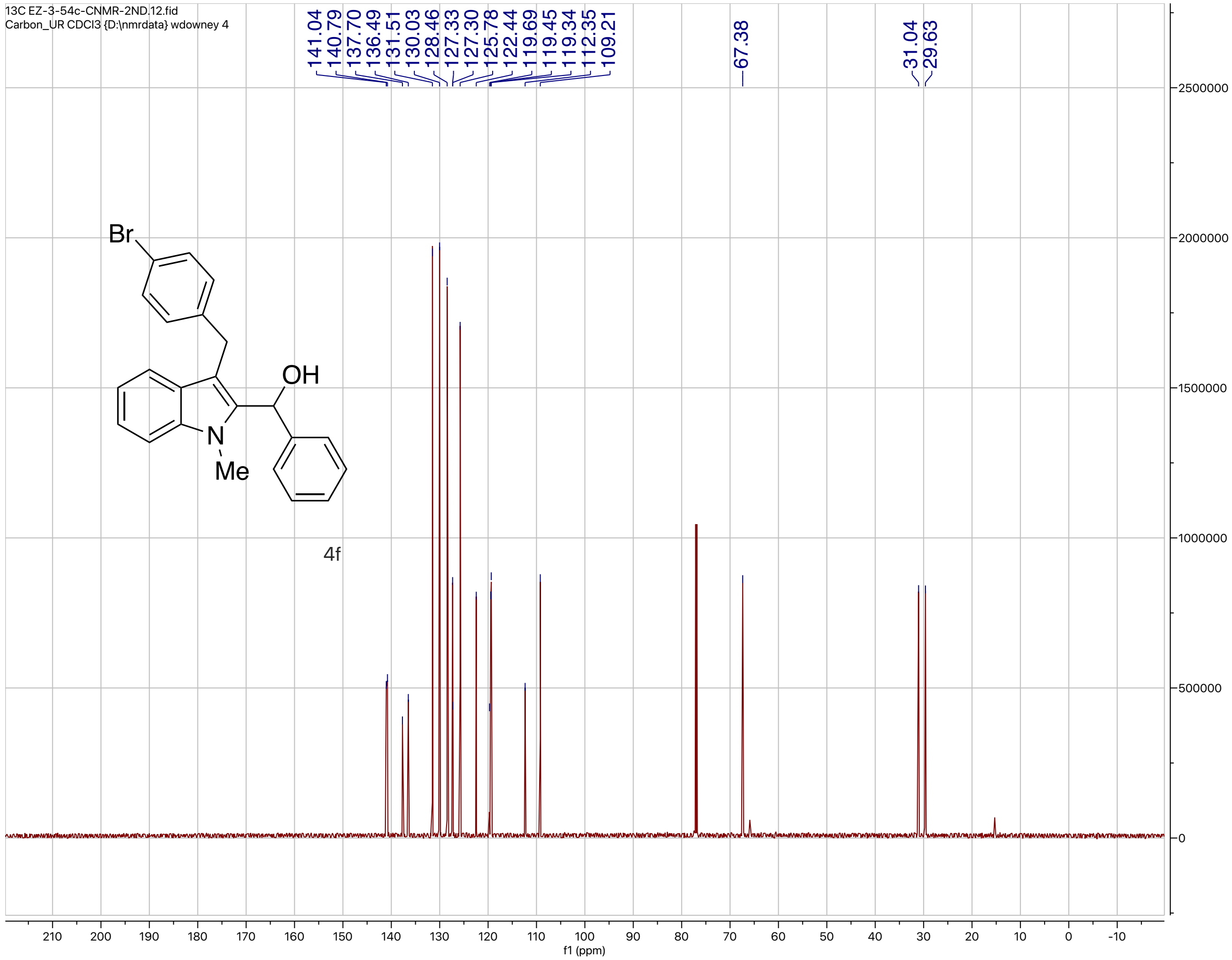

^{1}H NMR spectrum for product **4g** ($CDCl_3$, 500 MHz)

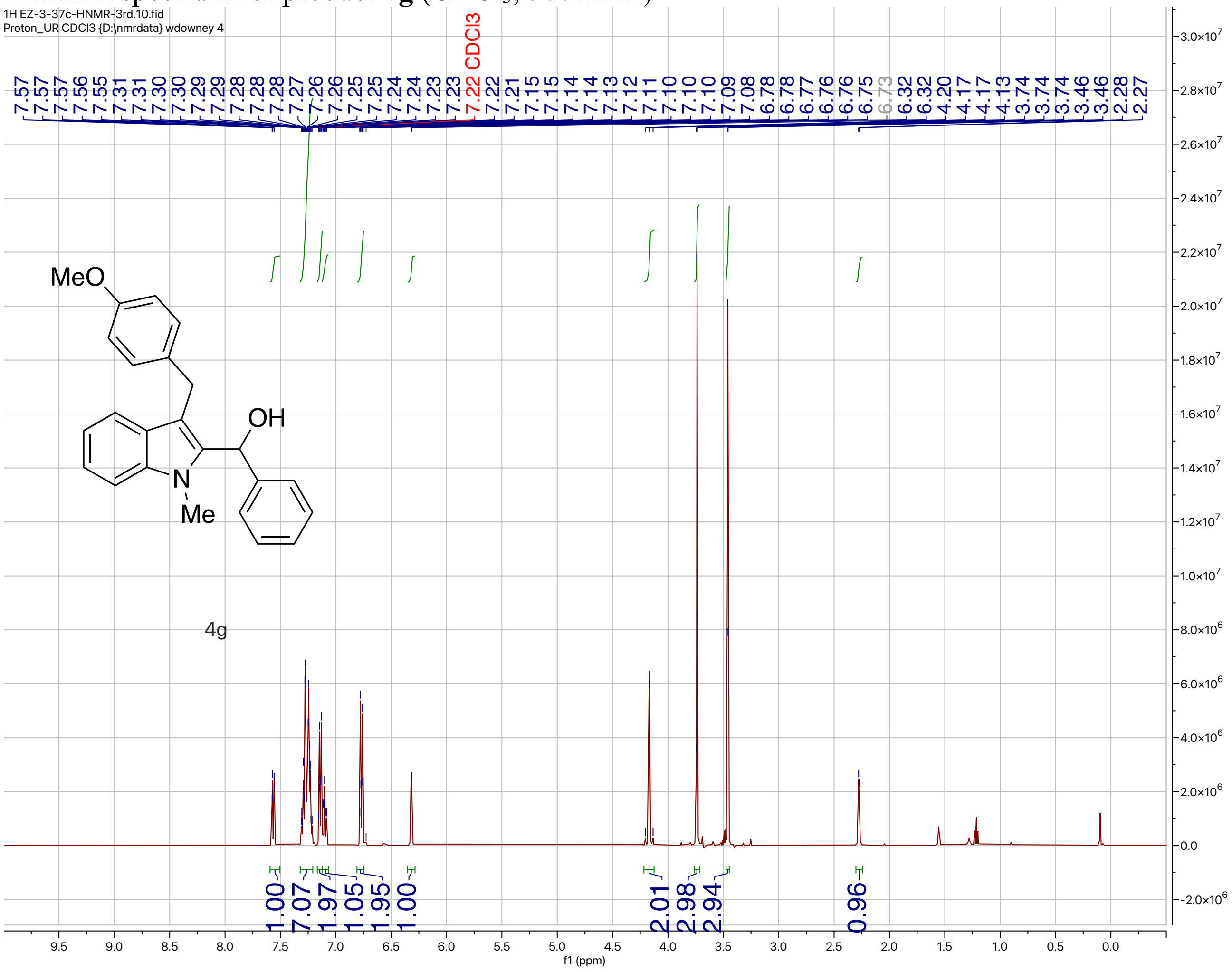

$^{13}C\{^{1}H\}$ NMR spectrum for product **4g** ($CDCl_3$, 126 MHz)

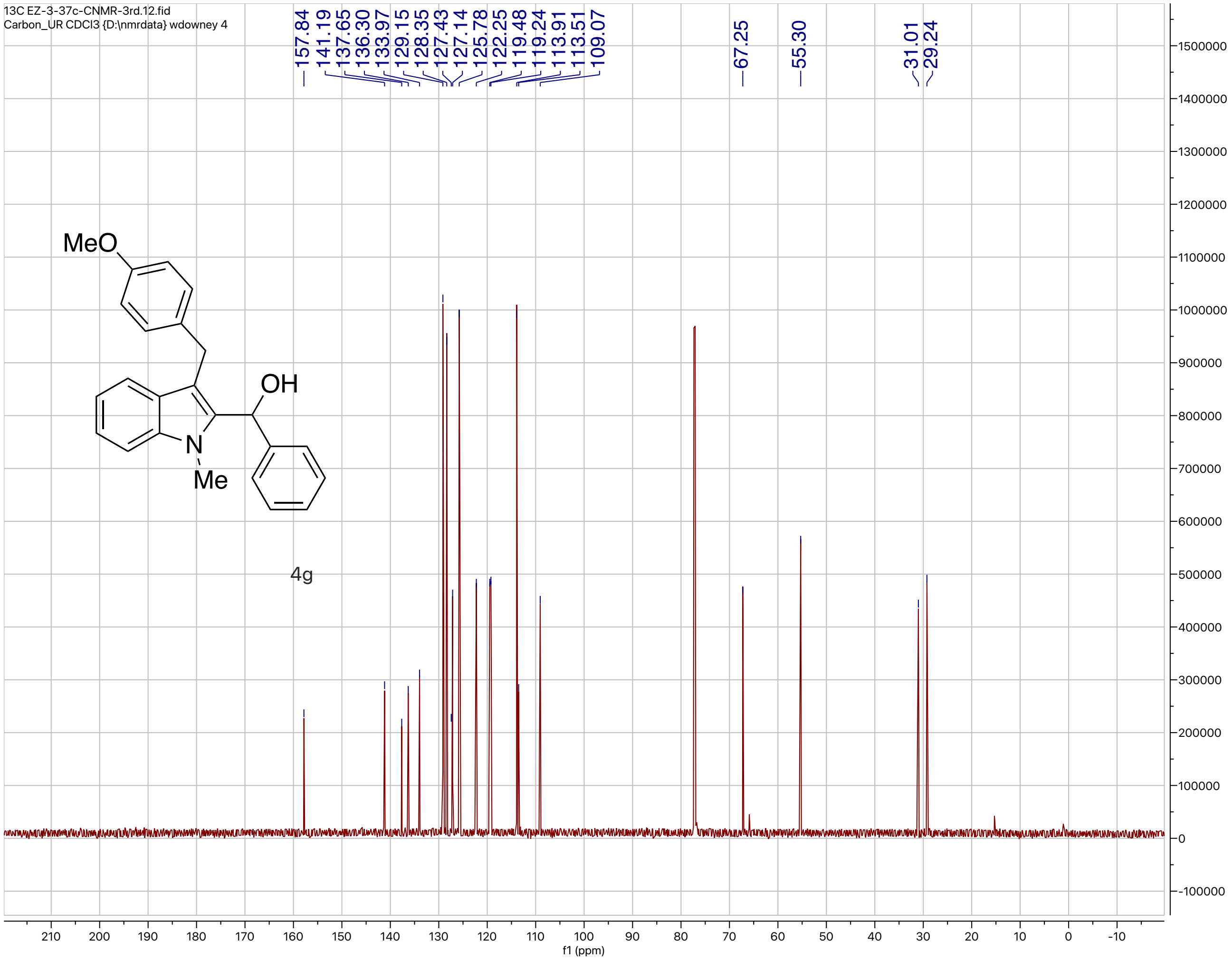

^{1}H NMR spectrum for product **4h** ($CDCl_3$, 500 MHz)

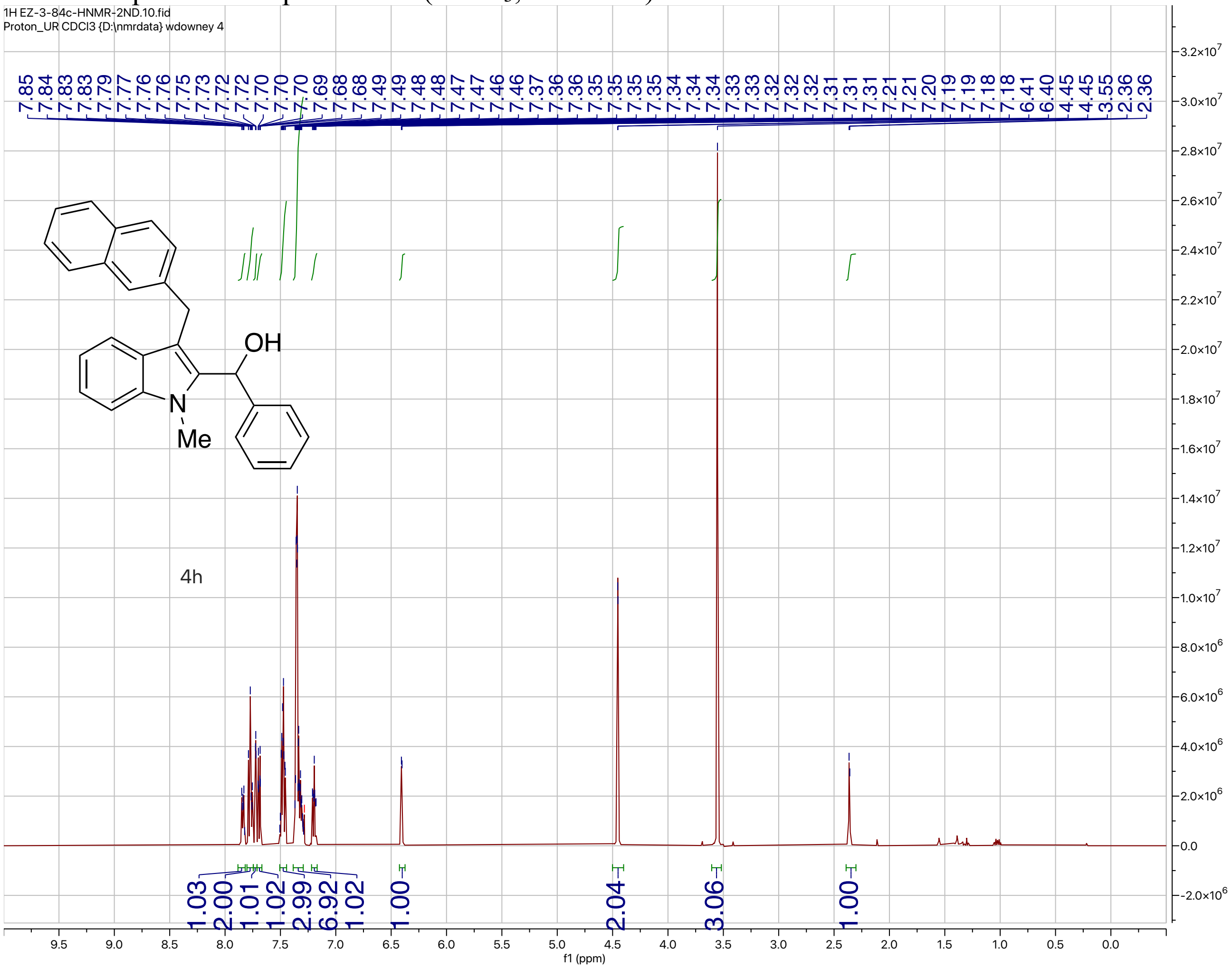

$^{13}C\{^{1}H\}$ NMR spectrum for product **4h** ($CDCl_3$, 126 MHz)

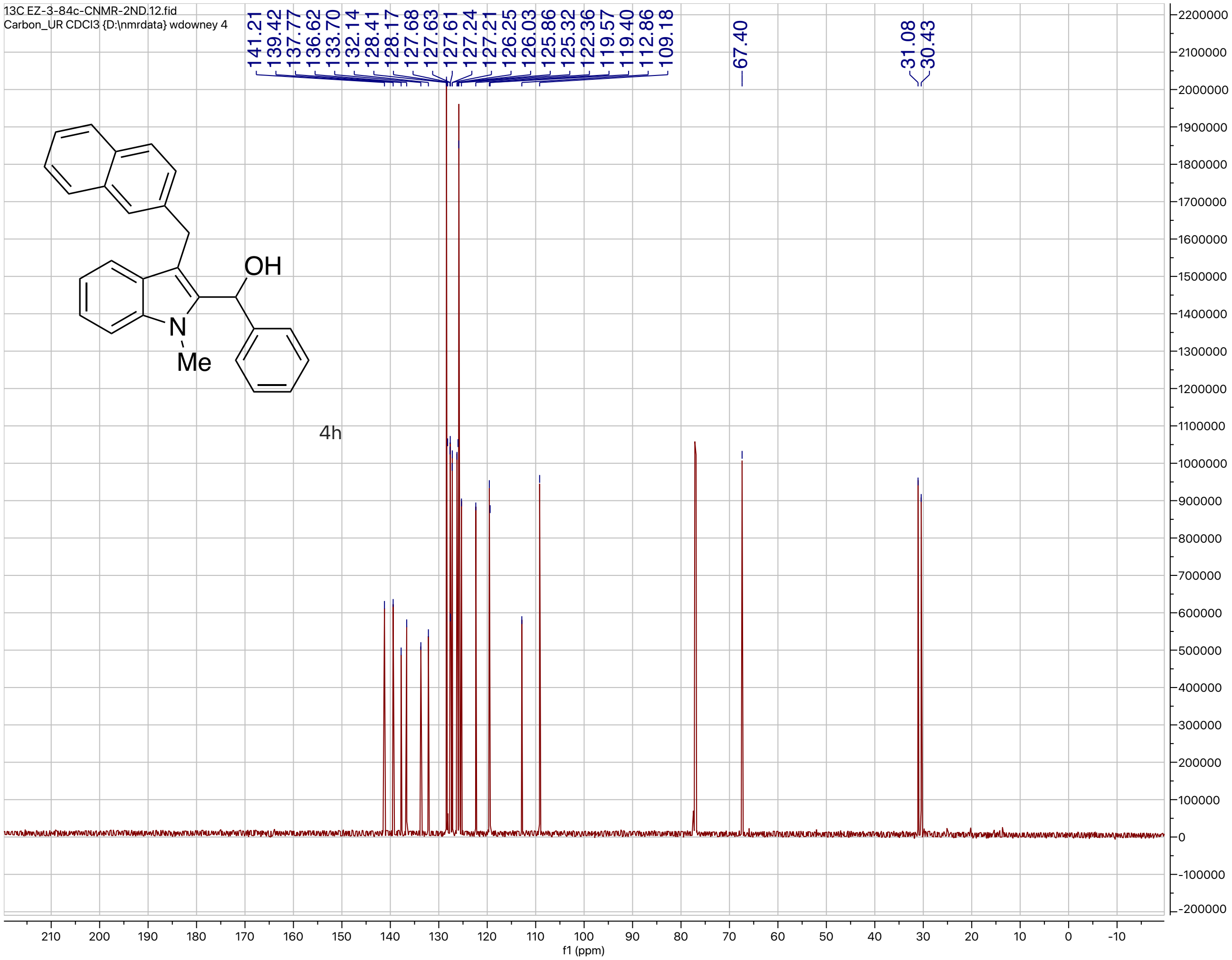

^{1}H NMR spectrum for product **4i** ($CDCl_3$, 500 MHz)

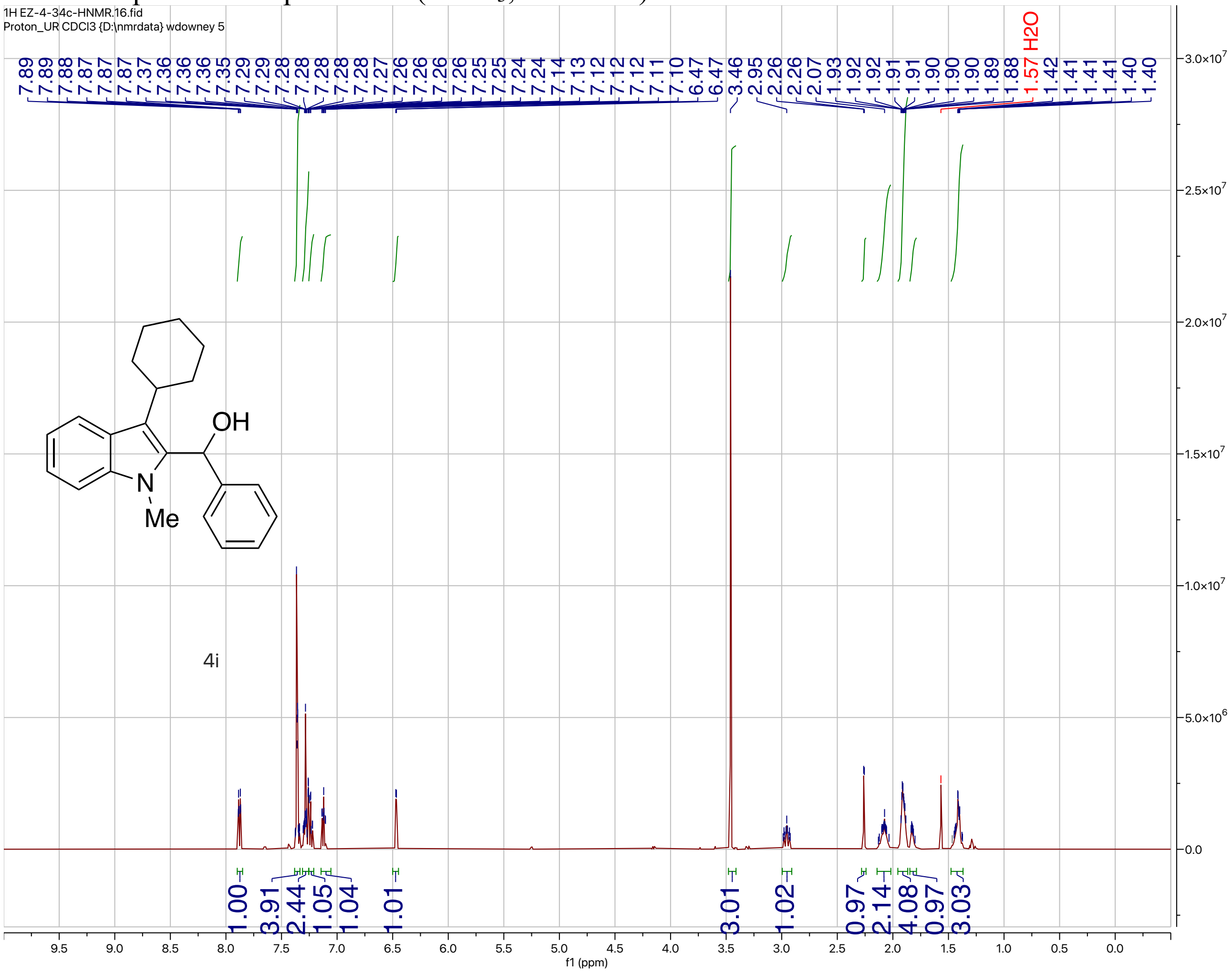

$^{13}C\{^{1}H\}$ NMR spectrum for product **4i** ($CDCl_3$, 126 MHz)

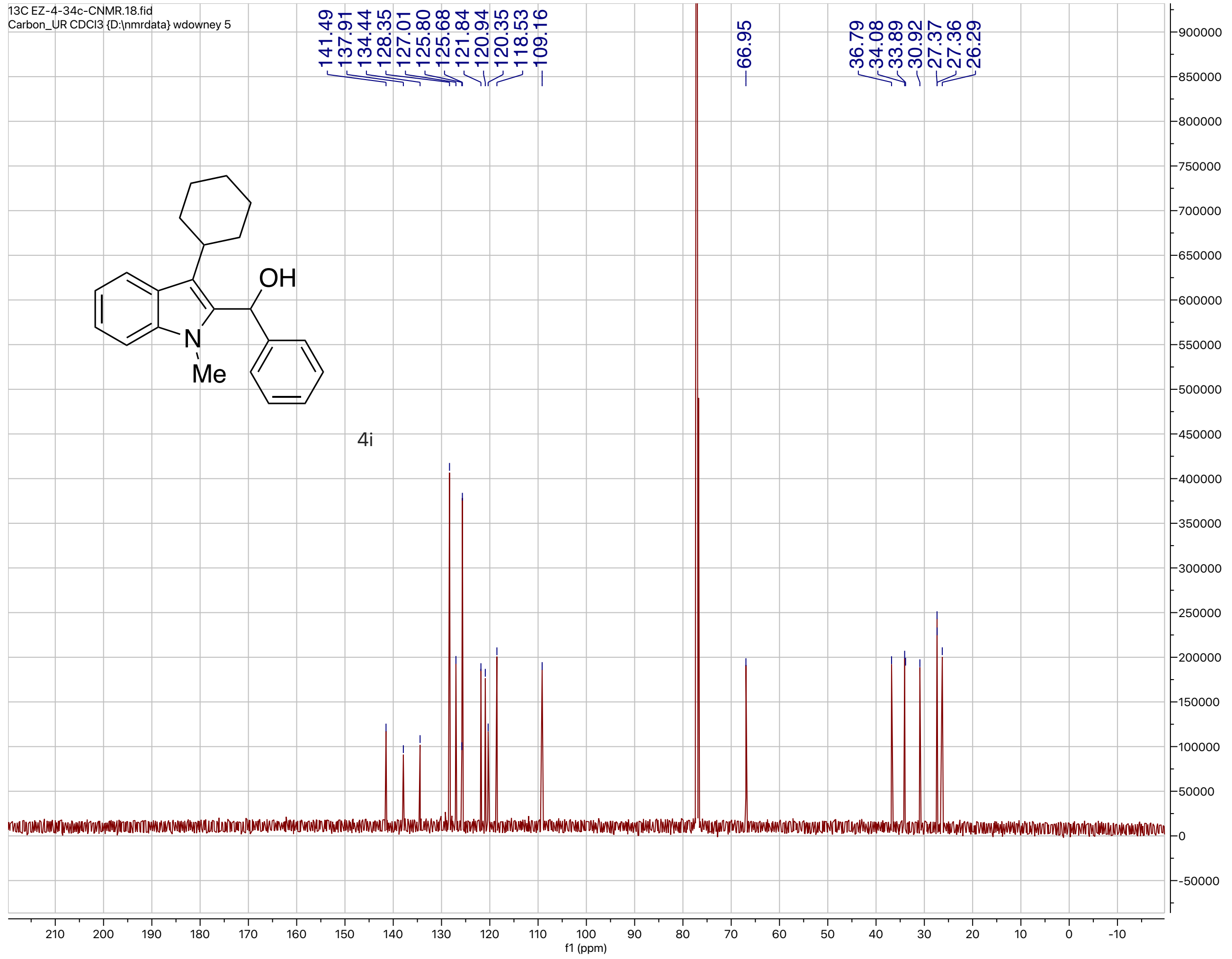

^{1}H NMR spectrum for product **5a** ($CDCl_3$, 500 MHz)

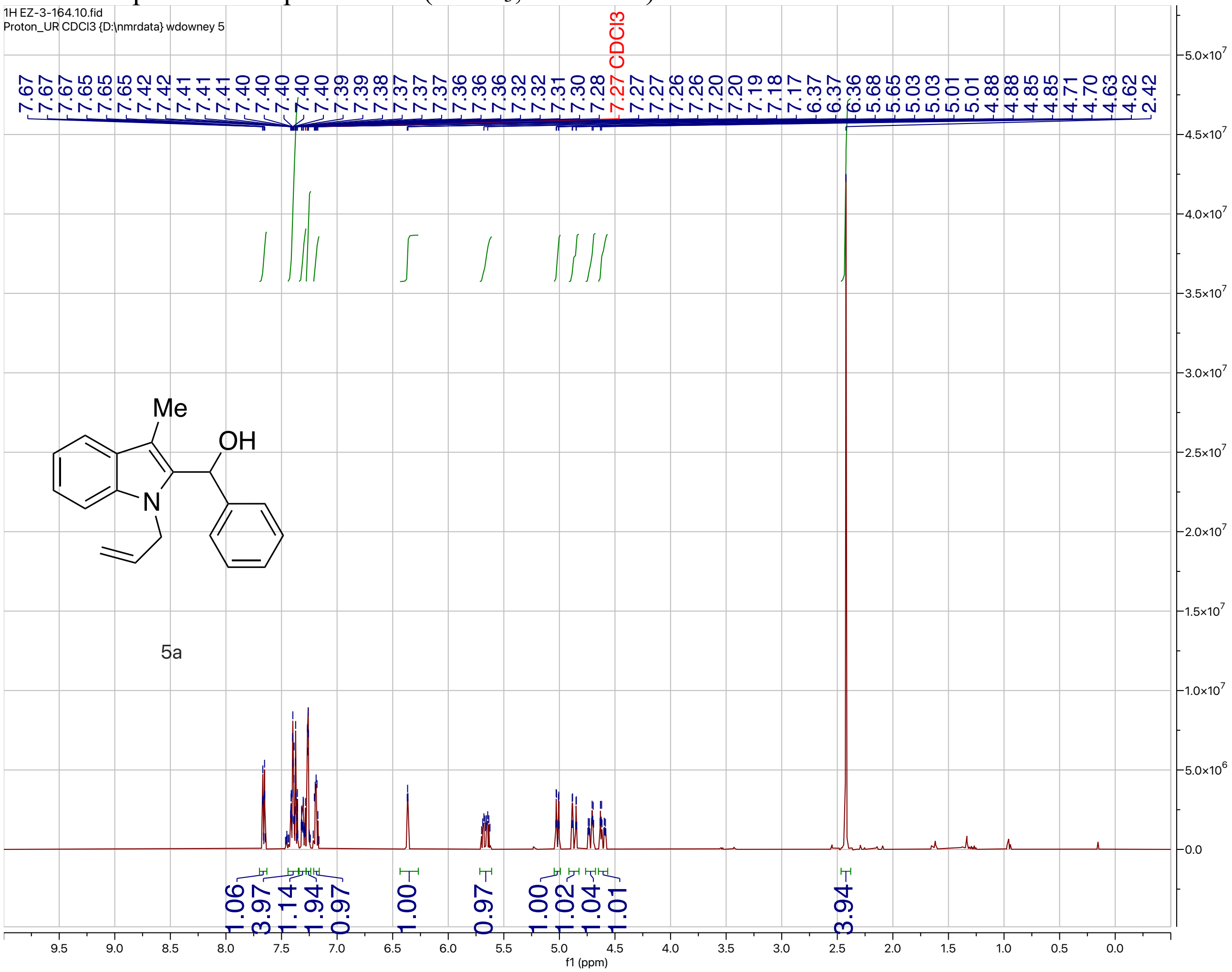

$^{13}C\{^{1}H\}$ NMR spectrum for product **5a** ($CDCl_3$, 126 MHz)

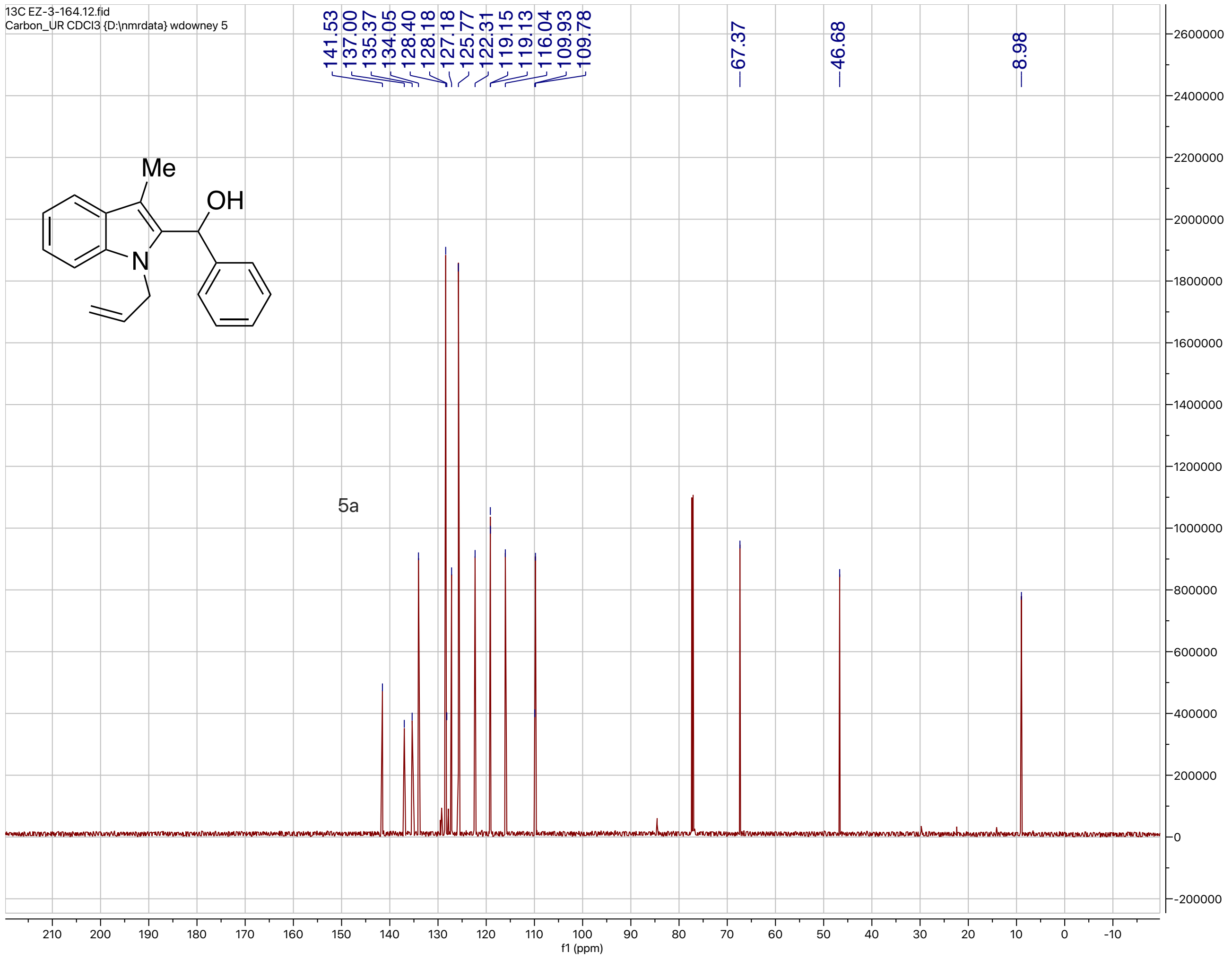

^{1}H NMR spectrum for product **5b** ($CDCl_3$, 500 MHz)

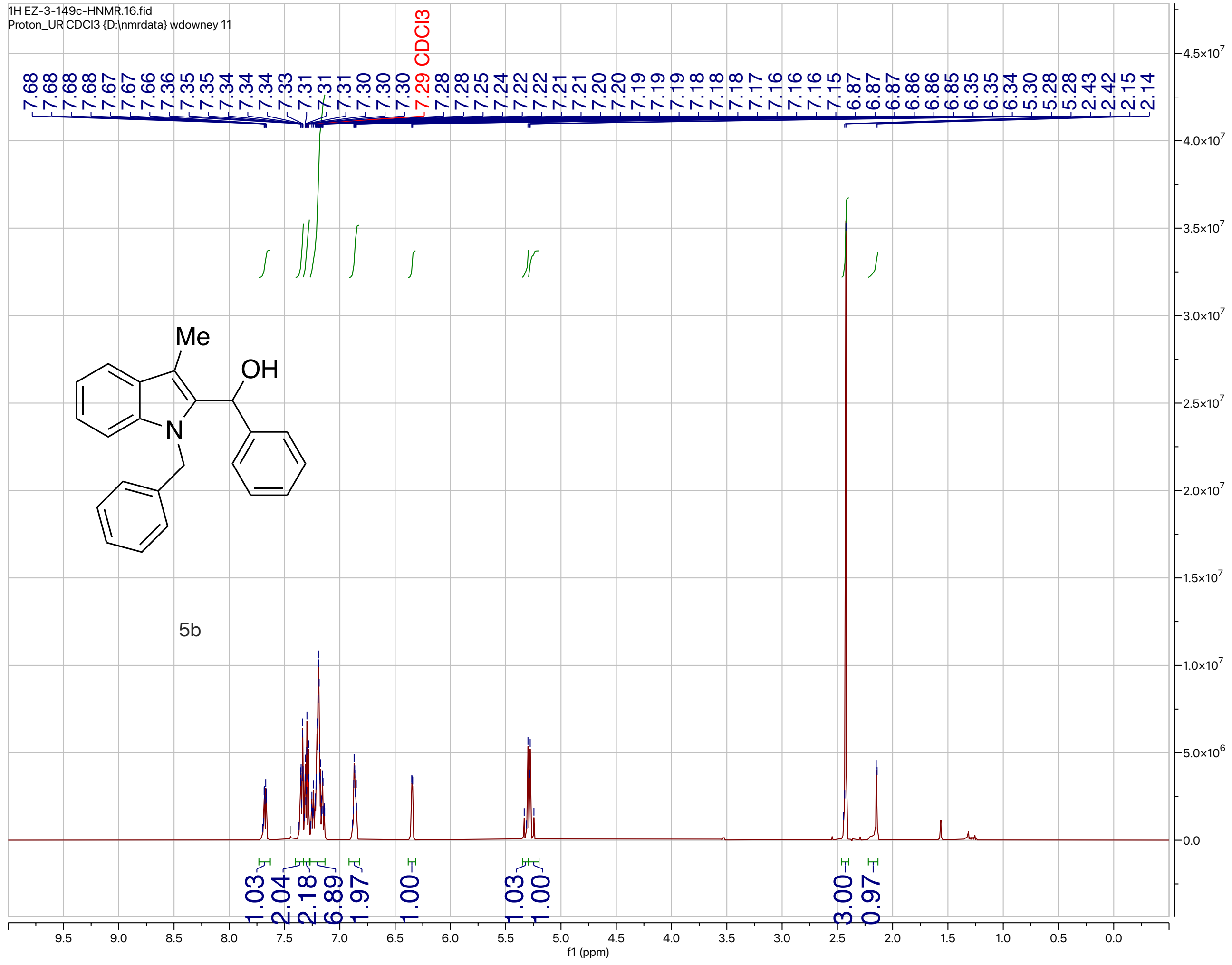

$^{13}C\{^{1}H\}$ NMR spectrum for product **5b** ($CDCl_3$, 126 MHz)

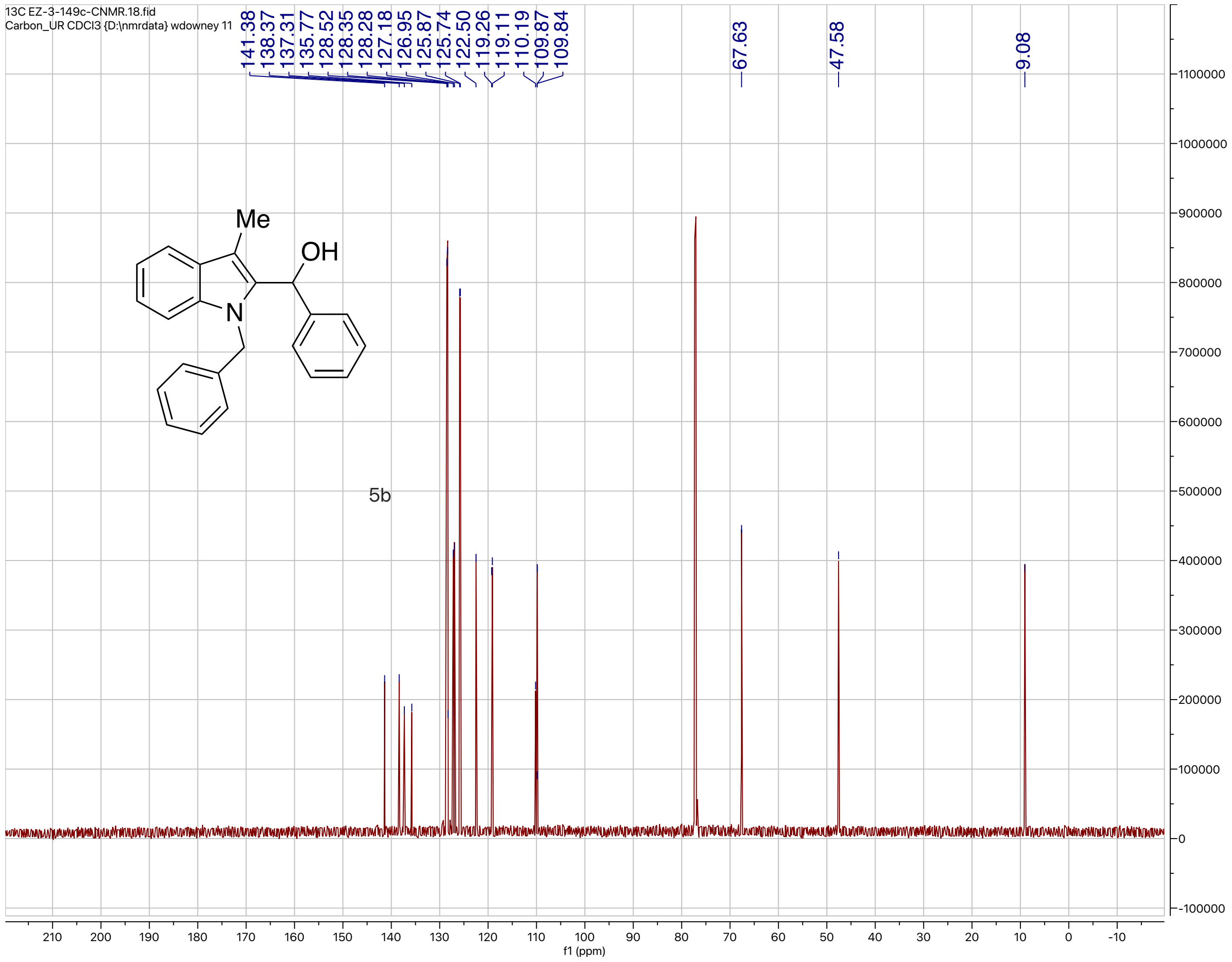

^{1}H NMR spectrum for product **5c** ($CDCl_3$, 500 MHz)

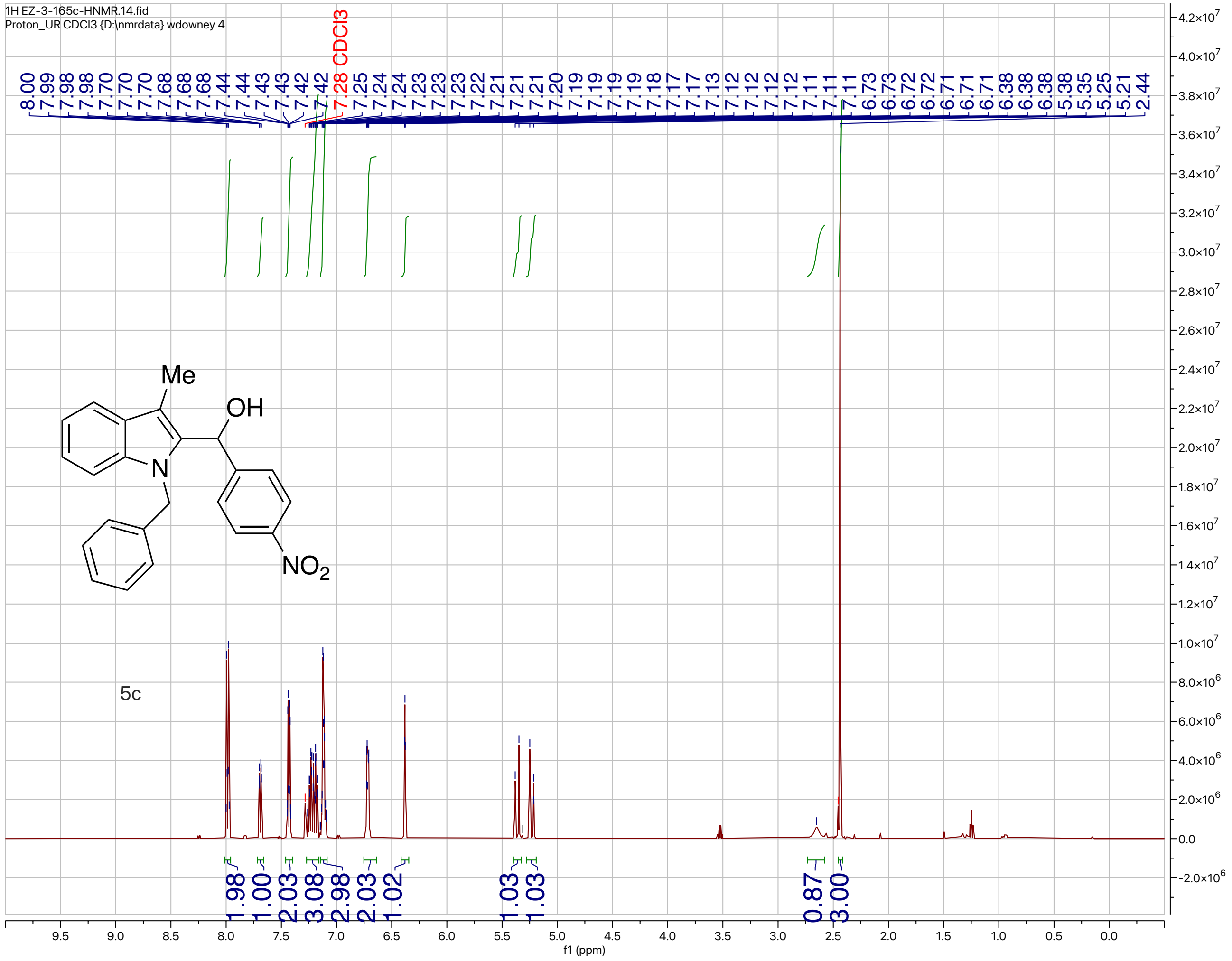

$^{13}C\{^{1}H\}$ NMR spectrum for product **5c** ($CDCl_3$, 126 MHz)

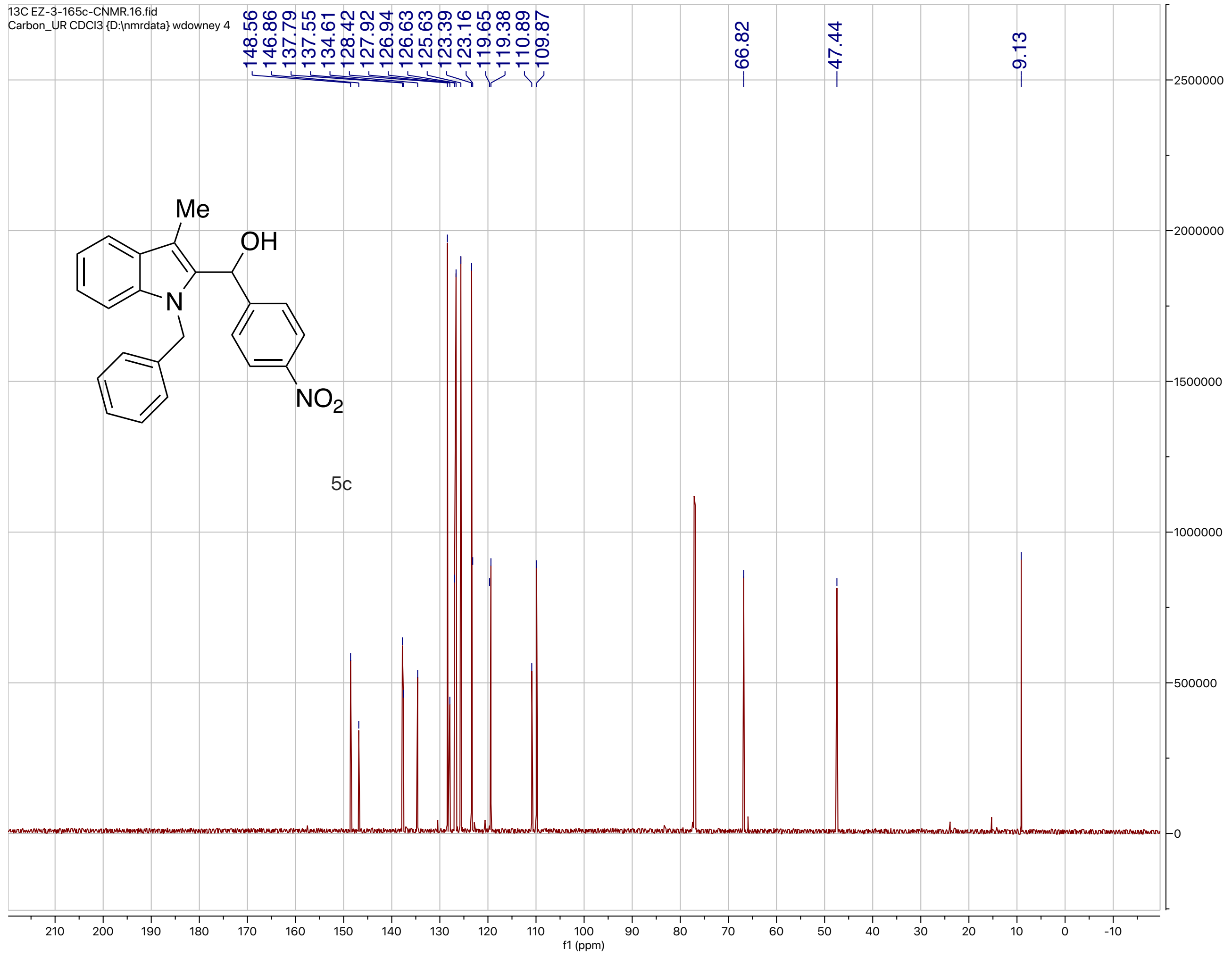

^{1}H NMR spectrum for product **5d** ($CDCl_3$, 500 MHz)

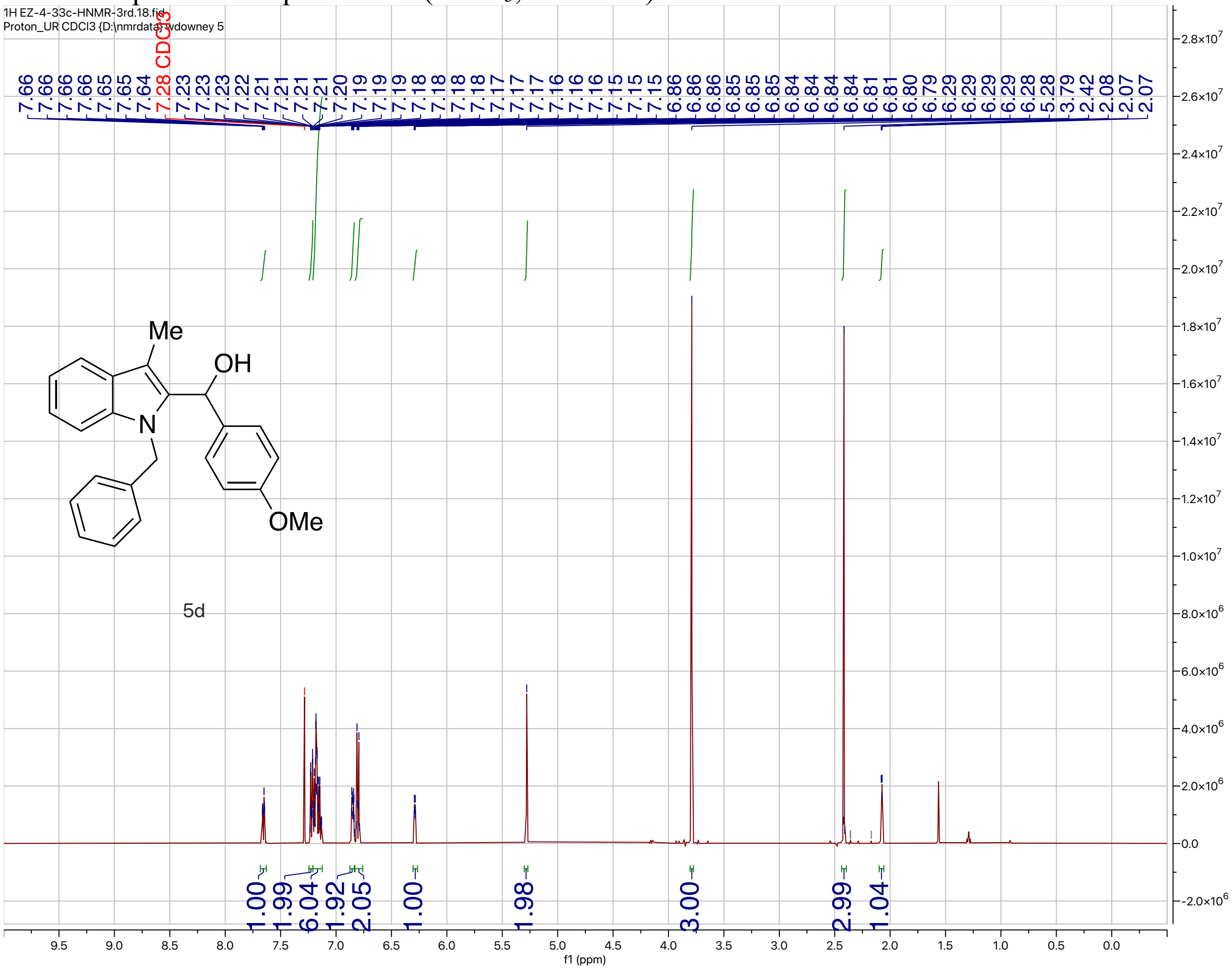

$^{13}C\{^{1}H\}$ NMR spectrum for product **5d** ($CDCl_3$, 126 MHz)

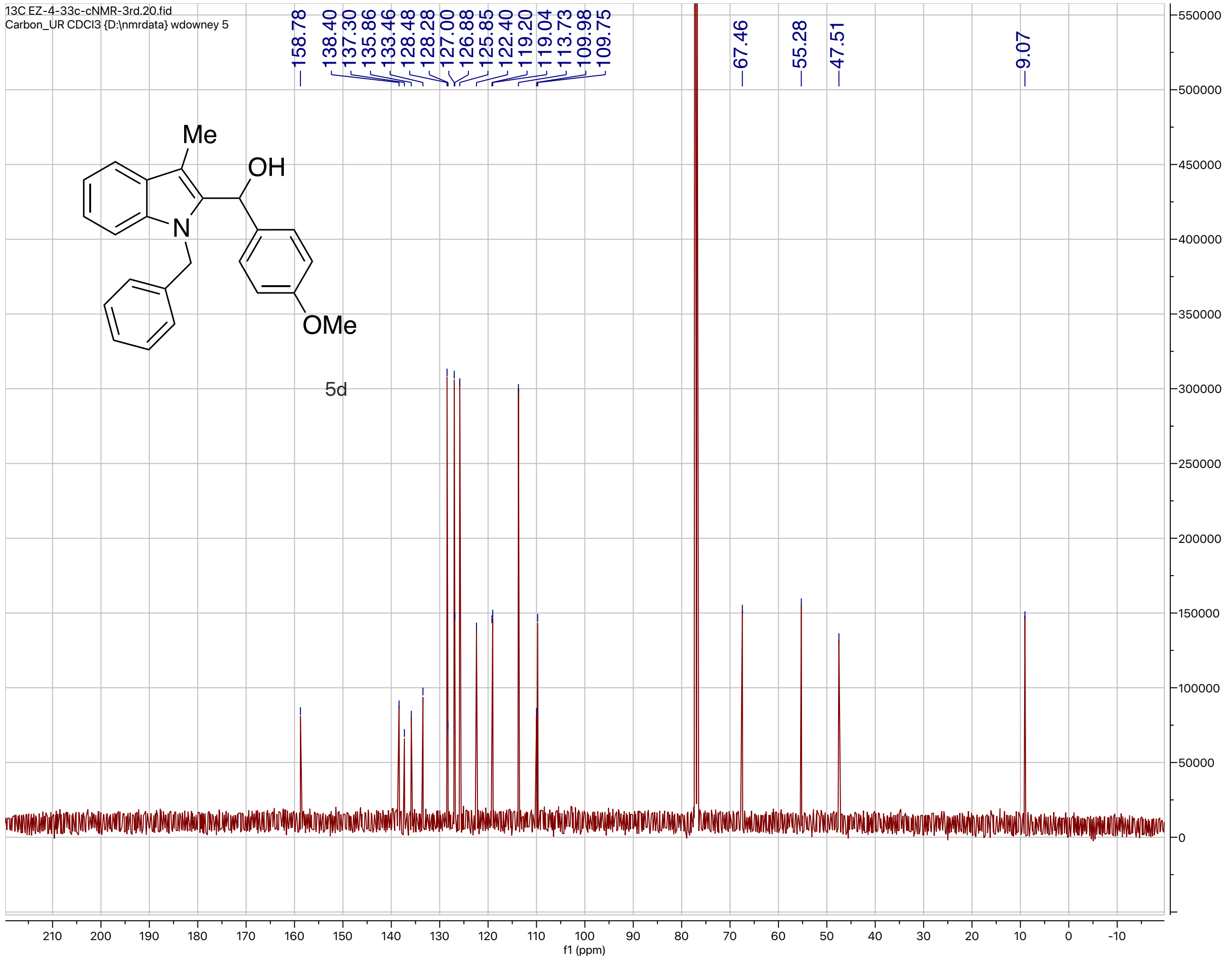

Supplement: Supplementary file 1 [file jo5c03195_si_001.pdf]
