# Supplementary material for: Friedel–Crafts Addition of 3‑Alkylated Indoles to Aldehydes: 2‑Hydroxyalkylation Promoted by Trimethylsilyl Trifluoromethanesulfonate
Source: J Org Chem. 2026 Apr 29;91(18):6255–67. doi: 10.1021/acs.joc.5c03195 (PMC13162324; doi:10.1021/acs.joc.5c03195)
Supplement: Supplementary file 2 [file jo5c03195_si_002.zip › FID for publication1/NMR info.docx]

**Spectrometers used for collection of data**

Bruker Advance lll 400 MHz, with BACS-60 Autosampler

Bruker Advance Neo 500 MHz with Sample Case-24

**Data processing software**

Bruker Topspin 4.50

Mestrenova version 15.0.1-35756
