# Supplementary material for: Friedel–Crafts Addition of 3‑Alkylated Indoles to Aldehydes: 2‑Hydroxyalkylation Promoted by Trimethylsilyl Trifluoromethanesulfonate
Source: J Org Chem. 2026 Apr 29;91(18):6255–67. doi: 10.1021/acs.joc.5c03195 (PMC13162324; doi:10.1021/acs.joc.5c03195)
Supplement: Supplementary file 2 [file jo5c03195_si_002.zip › FID for publication1/1f/1H EZ-2-74c-HNMR/32/pdata/1/email_EZ-2-74c-HNMR_32_1.pdf]

Proton_UR CDCl3 {D:\nmrdata} wdowney 2

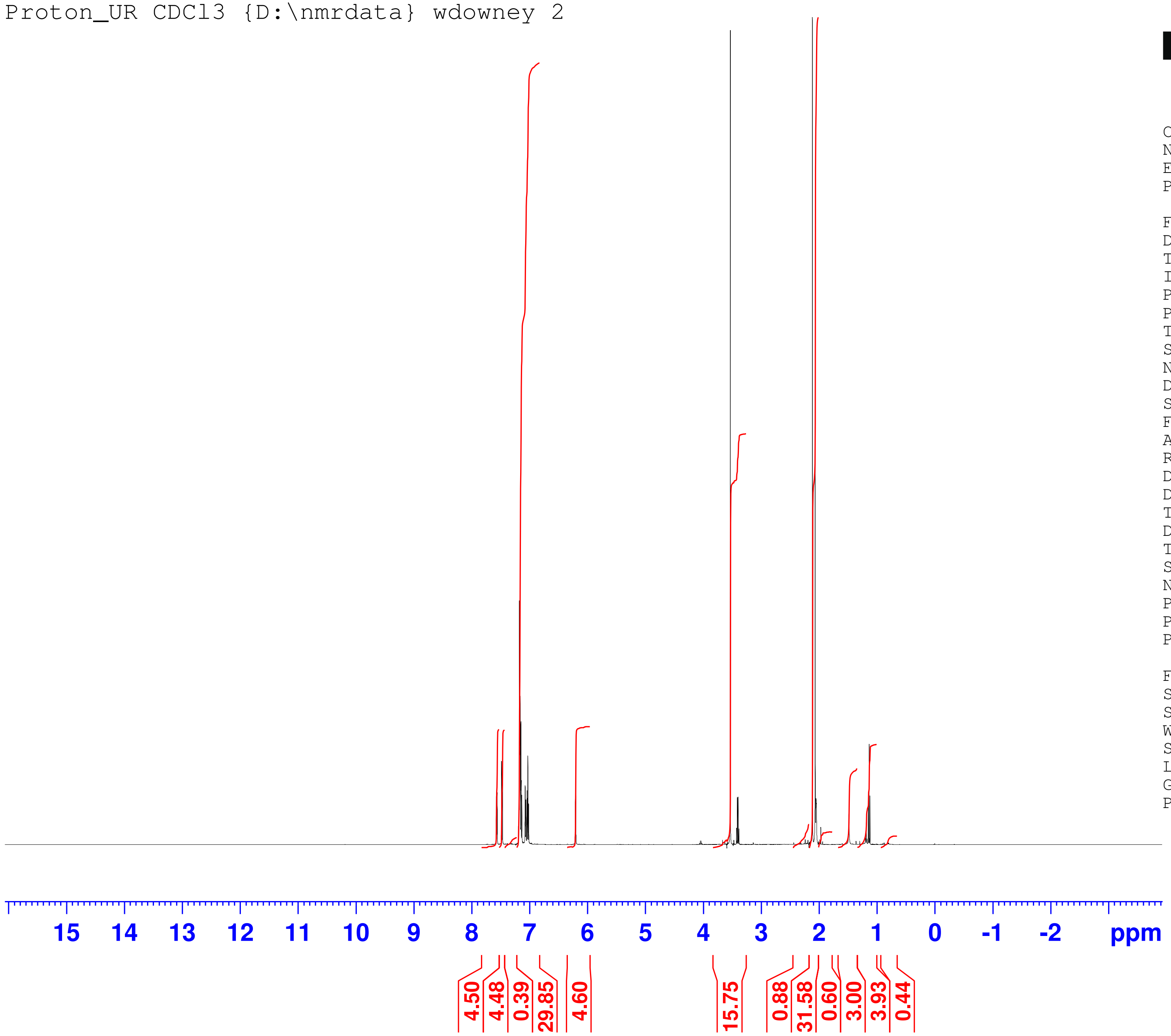

BRUKER

Current Data Parameters
NAME EZ-2-74c-HNMR
EXPNO 32
PROCNO 1

F2 - Acquisition Parameters
Date_ 20240717
Time 15.35 h
INSTRUM Avance
PROBHD Z169712_0016 (
PULPROG zg30
TD 65536
SOLVENT CDCl3
NS 16
DS 2
SWH 10000.000 Hz
FIDRES 0.305176 Hz
AQ 3.2767999 sec
RG 101
DW 50.000 usec
DE 11.14 usec
TE 295.0 K
D1 1.00000000 sec
TD0 1
SFO1 500.1330883 MHz
NUC1 1H
P0 2.65 usec
P1 7.96 usec
PLW1 25.72999954 W

F2 - Processing parameters
SI 65536
SF 500.1300537 MHz
WDW EM
SSB 0
LB 0.30 Hz
GB 0
PC 1.00
