# Supplementary material for: Friedel–Crafts Addition of 3‑Alkylated Indoles to Aldehydes: 2‑Hydroxyalkylation Promoted by Trimethylsilyl Trifluoromethanesulfonate
Source: J Org Chem. 2026 Apr 29;91(18):6255–67. doi: 10.1021/acs.joc.5c03195 (PMC13162324; doi:10.1021/acs.joc.5c03195)
Supplement: Supplementary file 2 [file jo5c03195_si_002.zip › FID for publication1/1a/1H EZ-1-21-H/12/pdata/1/email_EZ-1-21-H_12_1.pdf]

Proton_UR CDCl3 {D:\nmrdata} wdowney 4

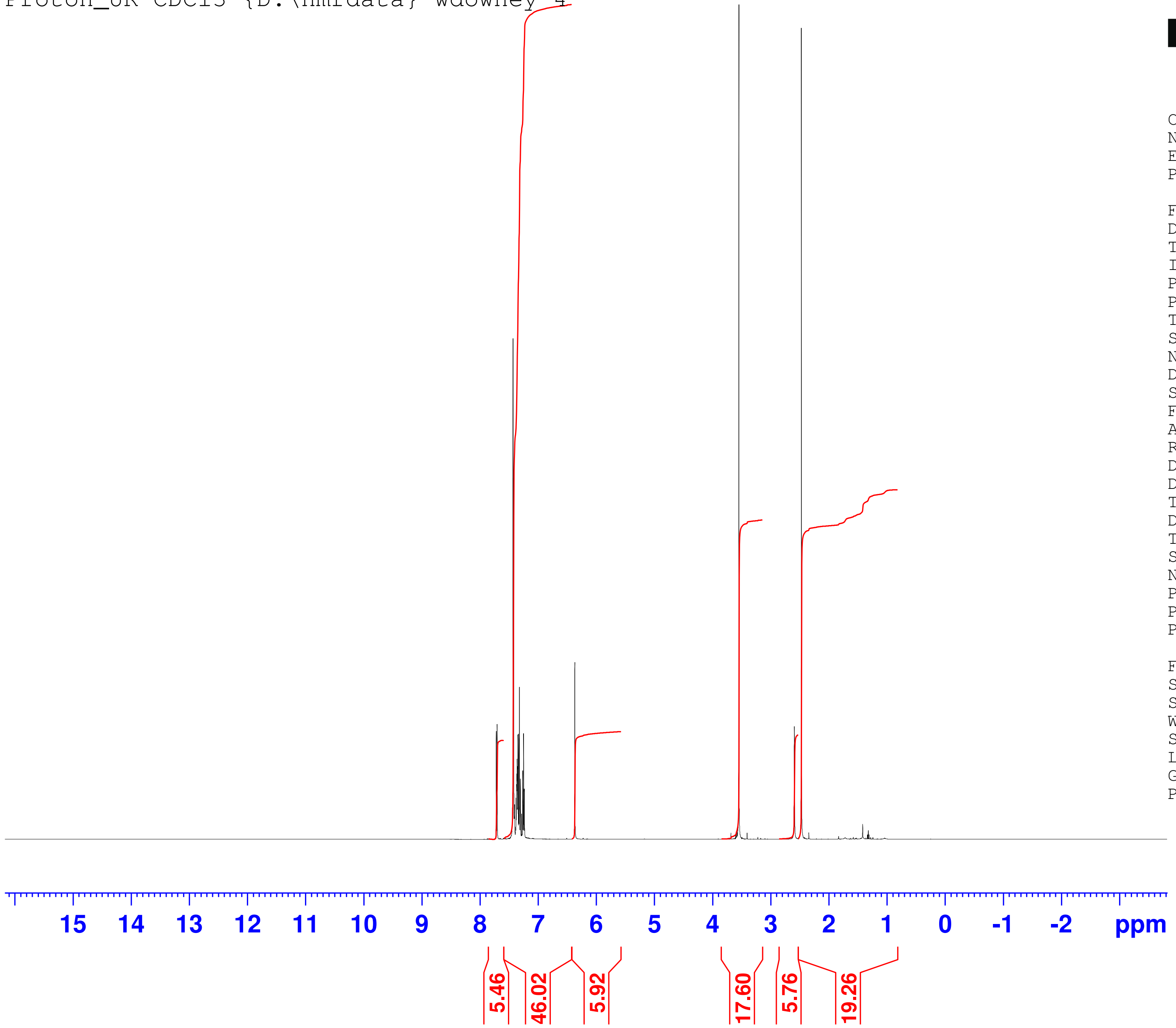

Current Data Parameters
NAME EZ-1-21-H
EXPNO 12
PROCNO 1

F2 - Acquisition Parameters

Date_	20240131
Time	10.09 h
INSTRUM	Avance
PROBHD	Z169712_0016 (
PULPROG	zg30
TD	65536
SOLVENT	CDCl3
NS	16
DS	2
SWH	10000.000 Hz
FIDRES	0.305176 Hz
AQ	3.2767999 sec
RG	32
DW	50.000 usec
DE	11.14 usec
TE	298.0 K
D1	1.00000000 sec
TD0	1
SFO1	500.1330883 MHz
NUC1	1H
P0	2.65 usec
P1	7.96 usec
PLW1	25.72999954 W

F2 - Processing parameters

SI	65536
SF	500.1300000 MHz
WDW	EM
SSB	0
LB	0.30 Hz
GB	0
PC	1.00
