# Supplementary material for: Friedel–Crafts Addition of 3‑Alkylated Indoles to Aldehydes: 2‑Hydroxyalkylation Promoted by Trimethylsilyl Trifluoromethanesulfonate
Source: J Org Chem. 2026 Apr 29;91(18):6255–67. doi: 10.1021/acs.joc.5c03195 (PMC13162324; doi:10.1021/acs.joc.5c03195)
Supplement: Supplementary file 2 [file jo5c03195_si_002.zip › FID for publication1/1n/1H EZ-2-133d-HNMR-2ND/10/pdata/1/email_EZ-2-133d-HNMR-2ND_10_1.pdf]

Proton_UR CDCl3 {D:\nmrdata} wdowney 7

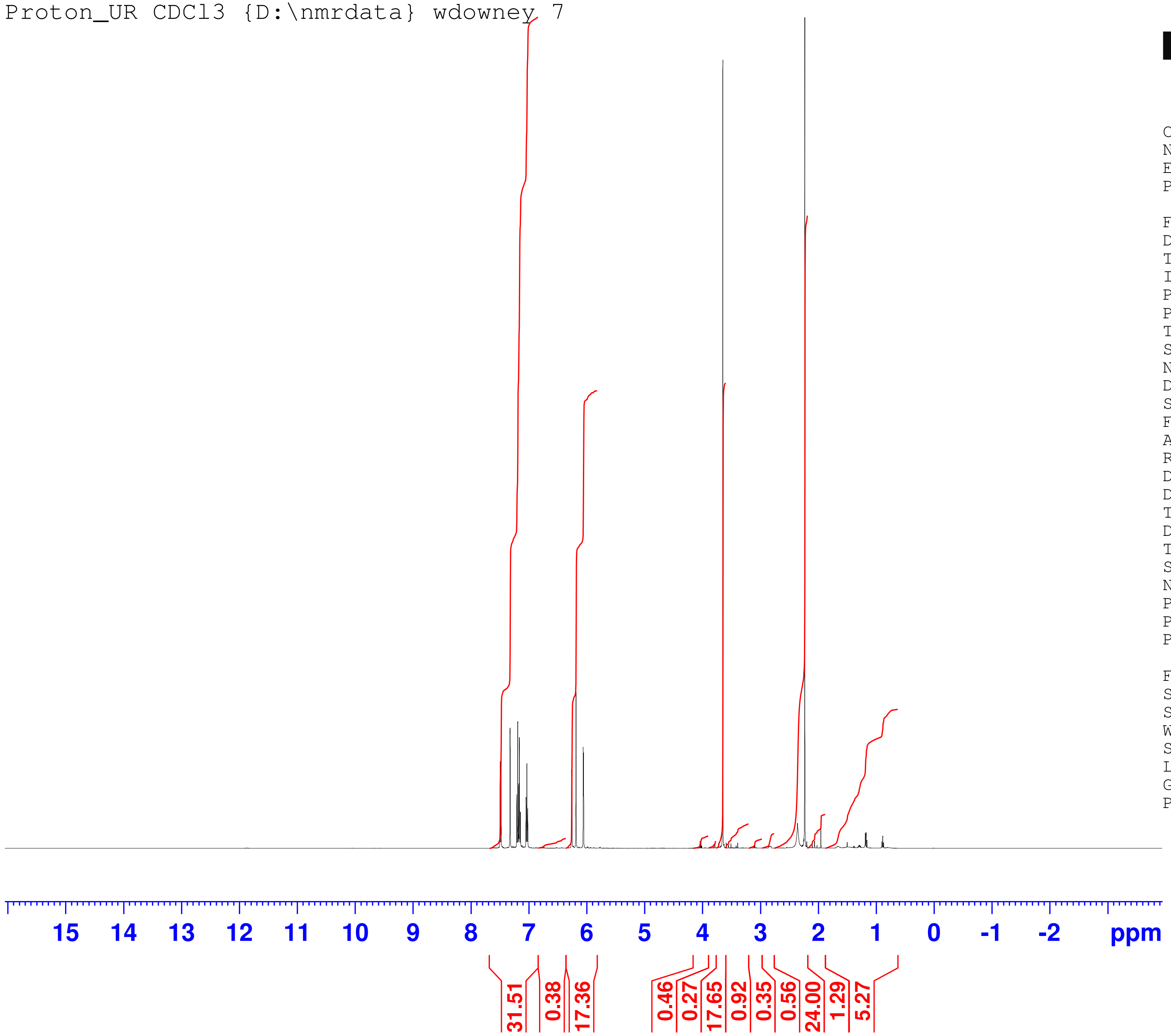


```
Current Data Parameters
NAME      EZ-2-133d-HNMR-2ND
EXPNO                     10
PROCNO                     1

F2 - Acquisition Parameters
Date_               20250206
Time                   19.07 h
INSTRUM               Avance
PROBHD      Z169712_0016 (
PULPROG                 zg30
TD                     65536
SOLVENT                CDCl3
NS                        16
DS                         2
SWH                10000.000 Hz
FIDRES              0.305176 Hz
AQ                 3.2767999 sec
RG                       101
DW                    50.000 usec
DE                     11.14 usec
TE                     305.0 K
D1                1.00000000 sec
TD0                        1
SFO1             500.1330883 MHz
NUC1                      1H
P0                      2.65 usec
P1                      7.96 usec
PLW1             25.72999954 W

F2 - Processing parameters
SI                     65536
SF               500.1300607 MHz
WDW                       EM
SSB                        0
LB                      0.30 Hz
GB                         0
PC                      1.00
```
