# Supplementary material for: Friedel–Crafts Addition of 3‑Alkylated Indoles to Aldehydes: 2‑Hydroxyalkylation Promoted by Trimethylsilyl Trifluoromethanesulfonate
Source: J Org Chem. 2026 Apr 29;91(18):6255–67. doi: 10.1021/acs.joc.5c03195 (PMC13162324; doi:10.1021/acs.joc.5c03195)
Supplement: Supplementary file 2 [file jo5c03195_si_002.zip › FID for publication1/1b/1H EZ-2-121c-HNMR/10/pdata/1/email_EZ-2-121c-HNMR_10_1.pdf]

Proton_UR CDCl3 {D:\nmrdata} wdowney 5

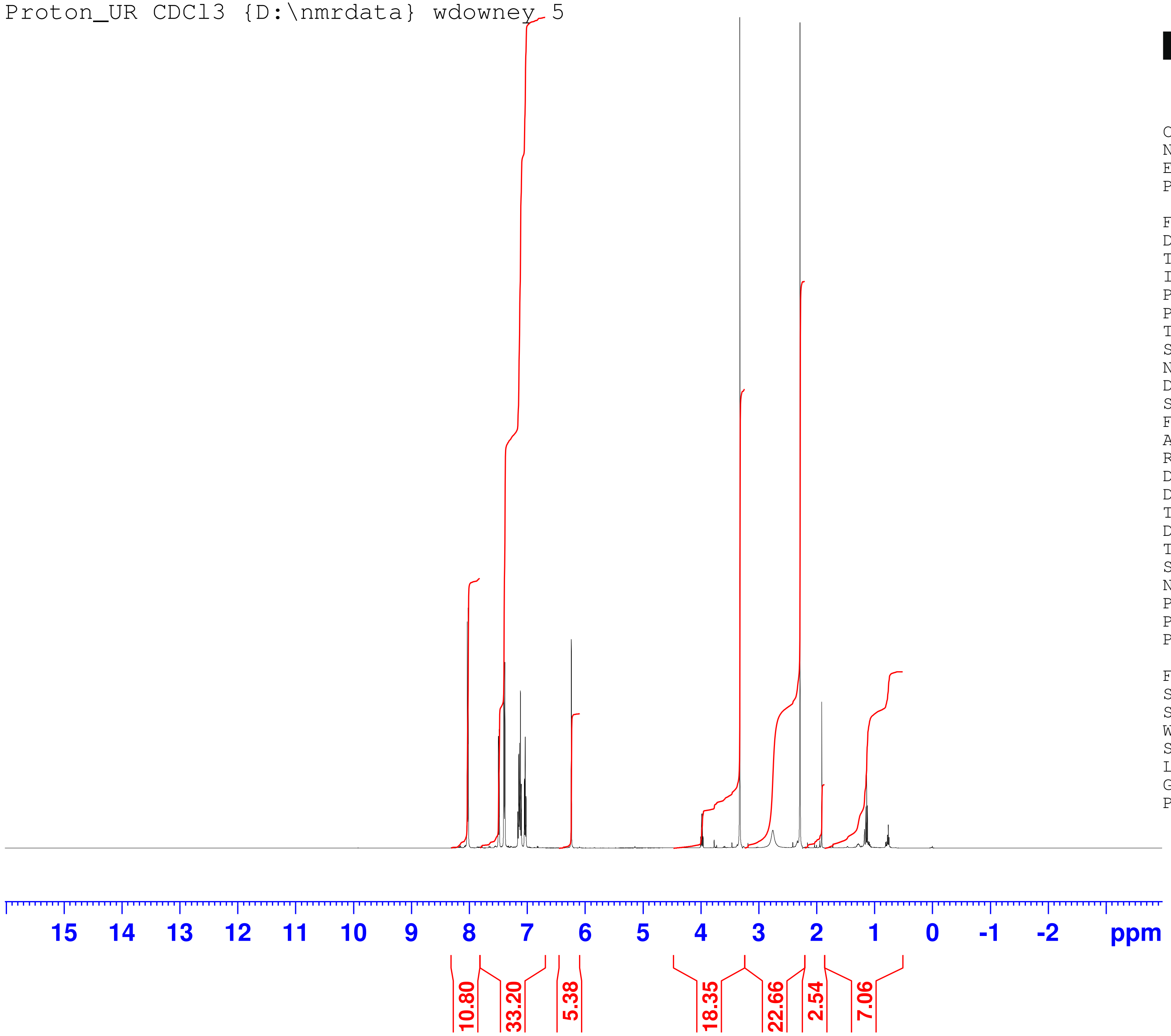

BRUKER

```
Current Data Parameters
NAME      EZ-2-121c-HNMR
EXPNO                 10
PROCNO                 1

F2 - Acquisition Parameters
Date_           20241203
Time                9.38 h
INSTRUM           Avance
PROBHD   Z169712_0016 (
PULPROG             zg30
TD                 65536
SOLVENT            CDCl3
NS                    16
DS                     2
SWH            10000.000 Hz
FIDRES          0.305176 Hz
AQ             3.2767999 sec
RG                  45.2
DW                50.000 usec
DE                 11.14 usec
TE                 295.2 K
D1            1.00000000 sec
TD0                    1
SFO1         500.1330883 MHz
NUC1                  1H
P0                  2.65 usec
P1                  7.96 usec
PLW1         25.72999954 W

F2 - Processing parameters
SI                 65536
SF           500.1300749 MHz
WDW                   EM
SSB                    0
LB                  0.30 Hz
GB                     0
PC                  1.00
```
