# Supplementary material for: Friedel–Crafts Addition of 3‑Alkylated Indoles to Aldehydes: 2‑Hydroxyalkylation Promoted by Trimethylsilyl Trifluoromethanesulfonate
Source: J Org Chem. 2026 Apr 29;91(18):6255–67. doi: 10.1021/acs.joc.5c03195 (PMC13162324; doi:10.1021/acs.joc.5c03195)
Supplement: Supplementary file 2 [file jo5c03195_si_002.zip › FID for publication1/1b/13C EZ-2-121c-CNMR/12/pdata/1/email_EZ-2-121c-CNMR_12_1.pdf]

Carbon_UR CDCl3 {D:\nmrdata} wdowney 5

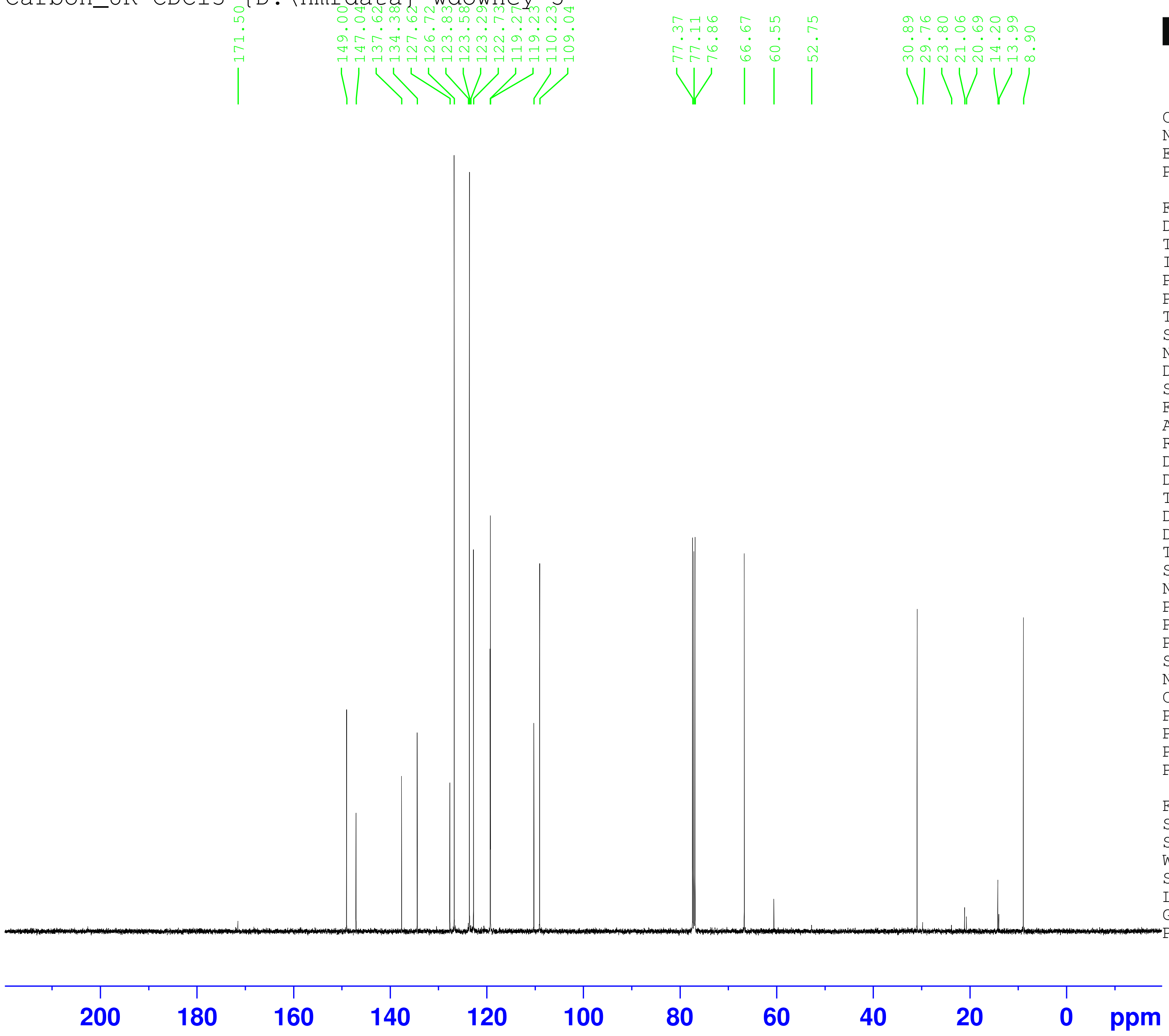

Current Data Parameters

NAME	EZ-2-121c-CNMR	
EXPNO	12	
PROCNO	1	

F2 - Acquisition Parameters

Date_	20241203	
Time	9.53	h
INSTRUM	Avance	
PROBHD	Z169712_0016 (	
PULPROG	zgpg30	
TD	65536	
SOLVENT	CDCl3	
NS	256	
DS	1	
SWH	30120.482	Hz
FIDRES	0.919204	Hz
AQ	1.0878977	sec
RG	101	
DW	16.600	usec
DE	6.50	usec
TE	295.3	K
D1	2.00000000	sec
D11	0.03000000	sec
TD0	1	
SFO1	125.7703643	MHz
NUC1	13C	
P0	3.00	usec
P1	9.00	usec
PLW1	104.23000336	W
SFO2	500.1320005	MHz
NUC2	1H	
CPDPRG[2	waltz65	
PCPD2	80.00	usec
PLW2	25.72999954	W
PLW12	0.25472999	W
PLW13	0.12813000	W

F2 - Processing parameters

SI	32768	
SF	125.7577885	MHz
WDW	EM	
SSB	0	
LB	1.00	Hz
GB	0	
PC	1.40	
