# Supplementary material for: Friedel–Crafts Addition of 3‑Alkylated Indoles to Aldehydes: 2‑Hydroxyalkylation Promoted by Trimethylsilyl Trifluoromethanesulfonate
Source: J Org Chem. 2026 Apr 29;91(18):6255–67. doi: 10.1021/acs.joc.5c03195 (PMC13162324; doi:10.1021/acs.joc.5c03195)
Supplement: Supplementary file 2 [file jo5c03195_si_002.zip › FID for publication1/1p/1H EZ-1-65-HNMR-4TH/10/pdata/1/email_EZ-1-65-HNMR-4TH_10_1.pdf]

Proton_UR CDCl3 {D:\nmrdata} wdowney 5

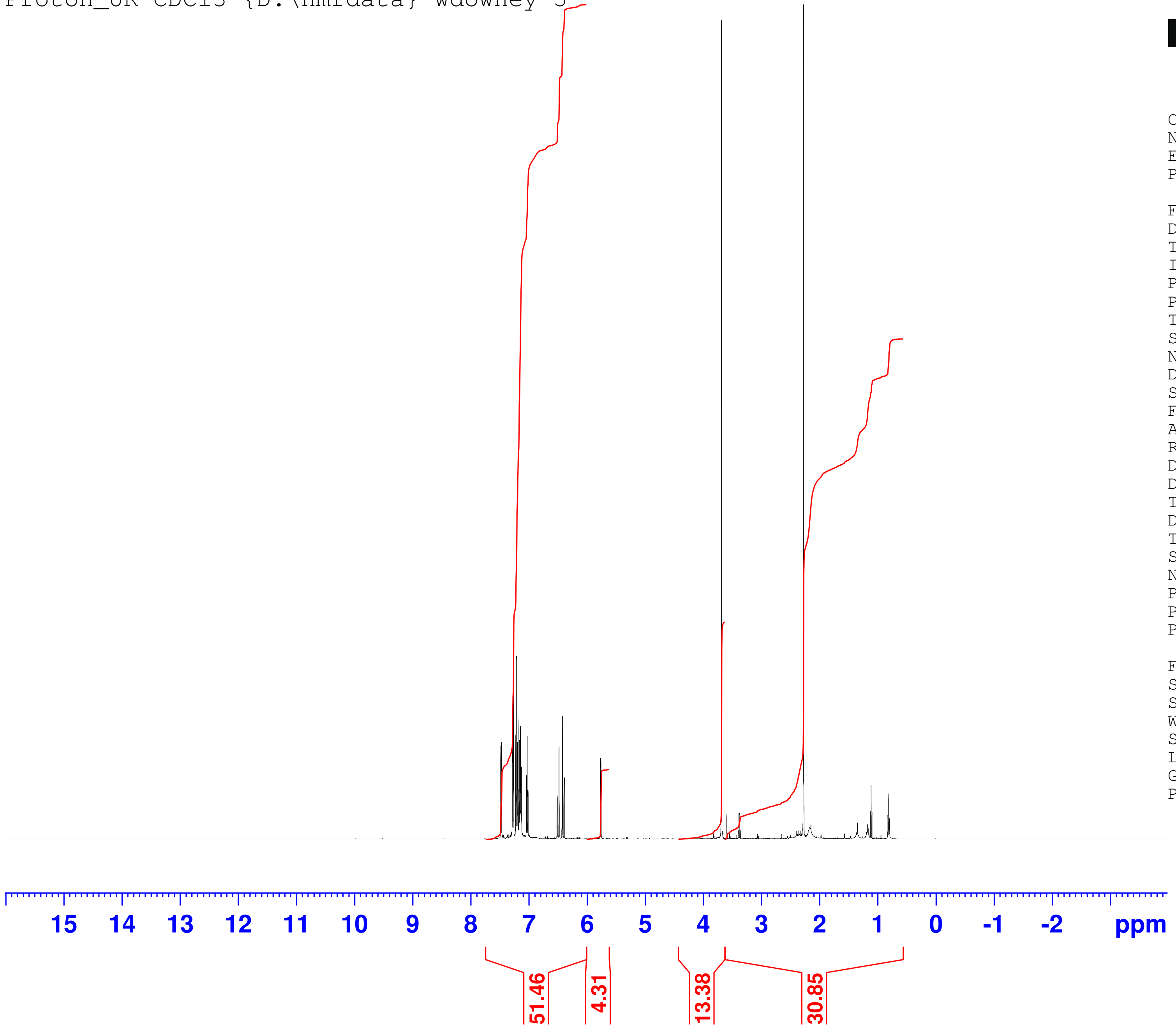


```
Current Data Parameters
NAME       EZ-1-65-HNMR-4TH
EXPNO                    10
PROCNO                    1

F2 - Acquisition Parameters
Date_              20240611
Time                   9.11 h
INSTRUM              Avance
PROBHD      Z169712_0016 (
PULPROG               zg30
TD                    65536
SOLVENT               CDCl3
NS                       16
DS                        2
SWH            10000.000 Hz
FIDRES          0.305176 Hz
AQ             3.2767999 sec
RG                     45.2
DW                   50.000 usec
DE                    11.14 usec
TE                    295.0 K
D1               1.00000000 sec
TD0                       1
SFO1         500.1330883 MHz
NUC1                     1H
P0                     2.65 usec
P1                     7.96 usec
PLW1         25.72999954 W

F2 - Processing parameters
SI                    65536
SF           500.1300788 MHz
WDW                      EM
SSB                       0
LB                     0.30 Hz
GB                        0
PC                     1.00
```
