# Supplementary material for: Friedel–Crafts Addition of 3‑Alkylated Indoles to Aldehydes: 2‑Hydroxyalkylation Promoted by Trimethylsilyl Trifluoromethanesulfonate
Source: J Org Chem. 2026 Apr 29;91(18):6255–67. doi: 10.1021/acs.joc.5c03195 (PMC13162324; doi:10.1021/acs.joc.5c03195)
Supplement: Supplementary file 2 [file jo5c03195_si_002.zip › FID for publication1/1c/1H EZ-1-43-HNMR/10/pdata/1/email_EZ-1-43-HNMR_10_1.pdf]

Proton_UR CDCl3 {D:\nmrdata} wdowney 2

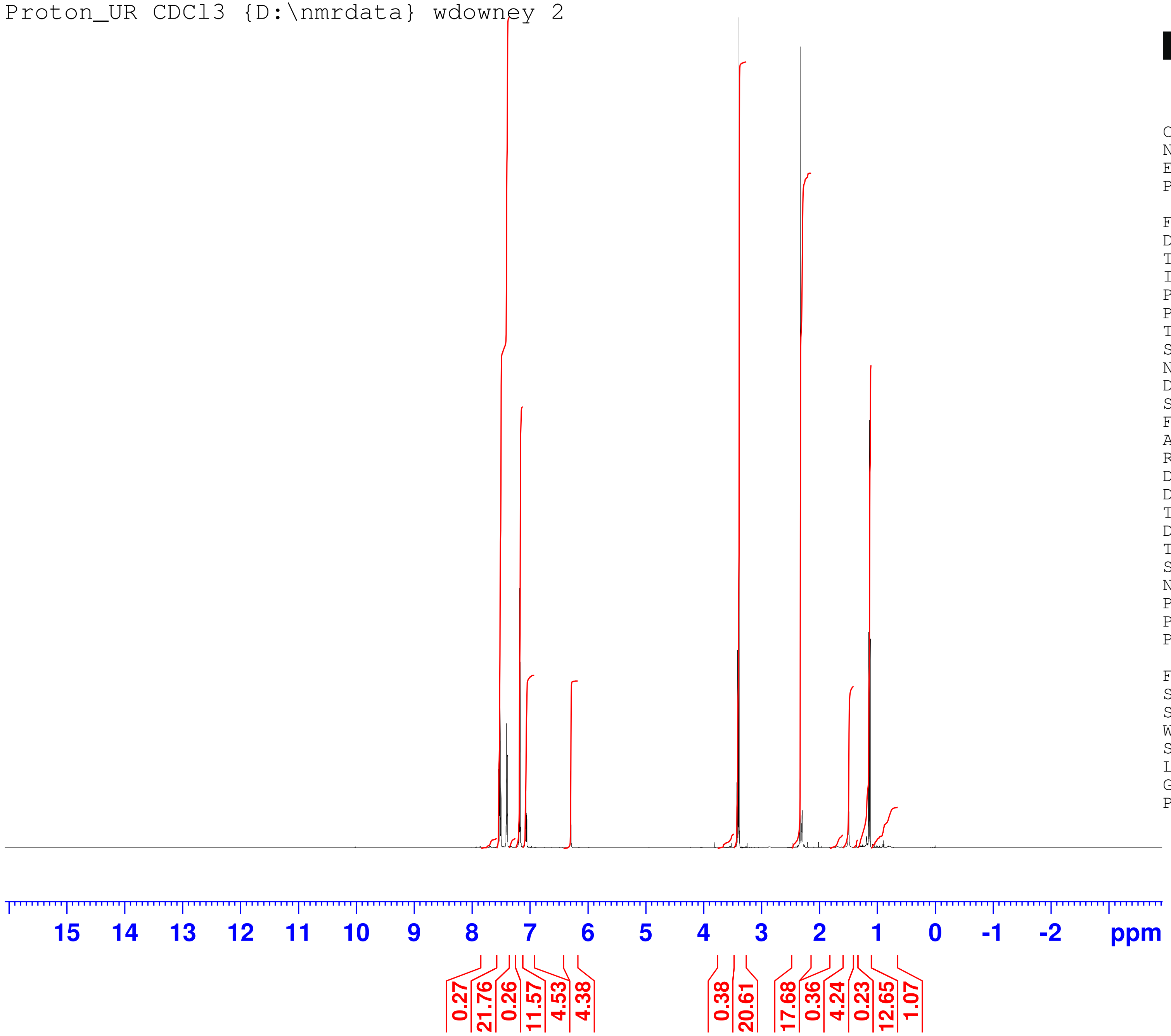

BRUKER

Current Data Parameters
NAME EZ-1-43-HNMR
EXPNO 10
PROCNO 1

F2 - Acquisition Parameters

Date_	20240523	
Time	15.35	h
INSTRUM	Avance	
PROBHD	Z169712_0016 (	
PULPROG	zg30	
TD	65536	
SOLVENT	CDCl3	
NS	16	
DS	2	
SWH	10000.000	Hz
FIDRES	0.305176	Hz
AQ	3.2767999	sec
RG	101	
DW	50.000	usec
DE	11.14	usec
TE	295.0	K
D1	1.00000000	sec
TD0	1	
SFO1	500.1330883	MHz
NUC1	1H	
P0	2.65	usec
P1	7.96	usec
PLW1	25.72999954	W

F2 - Processing parameters

SI	65536	
SF	500.1300508	MHz
WDW	EM	
SSB	0	
LB	0.30	Hz
GB	0	
PC	1.00	
