# Supplementary material for: Friedel–Crafts Addition of 3‑Alkylated Indoles to Aldehydes: 2‑Hydroxyalkylation Promoted by Trimethylsilyl Trifluoromethanesulfonate
Source: J Org Chem. 2026 Apr 29;91(18):6255–67. doi: 10.1021/acs.joc.5c03195 (PMC13162324; doi:10.1021/acs.joc.5c03195)
Supplement: Supplementary file 2 [file jo5c03195_si_002.zip › FID for publication1/1m/13C EZ-1-33-CNMR/12/pdata/1/email_EZ-1-33-CNMR_12_1.pdf]

Carbon_UR CDCl3 {D:\nmrdata} wdowney 5

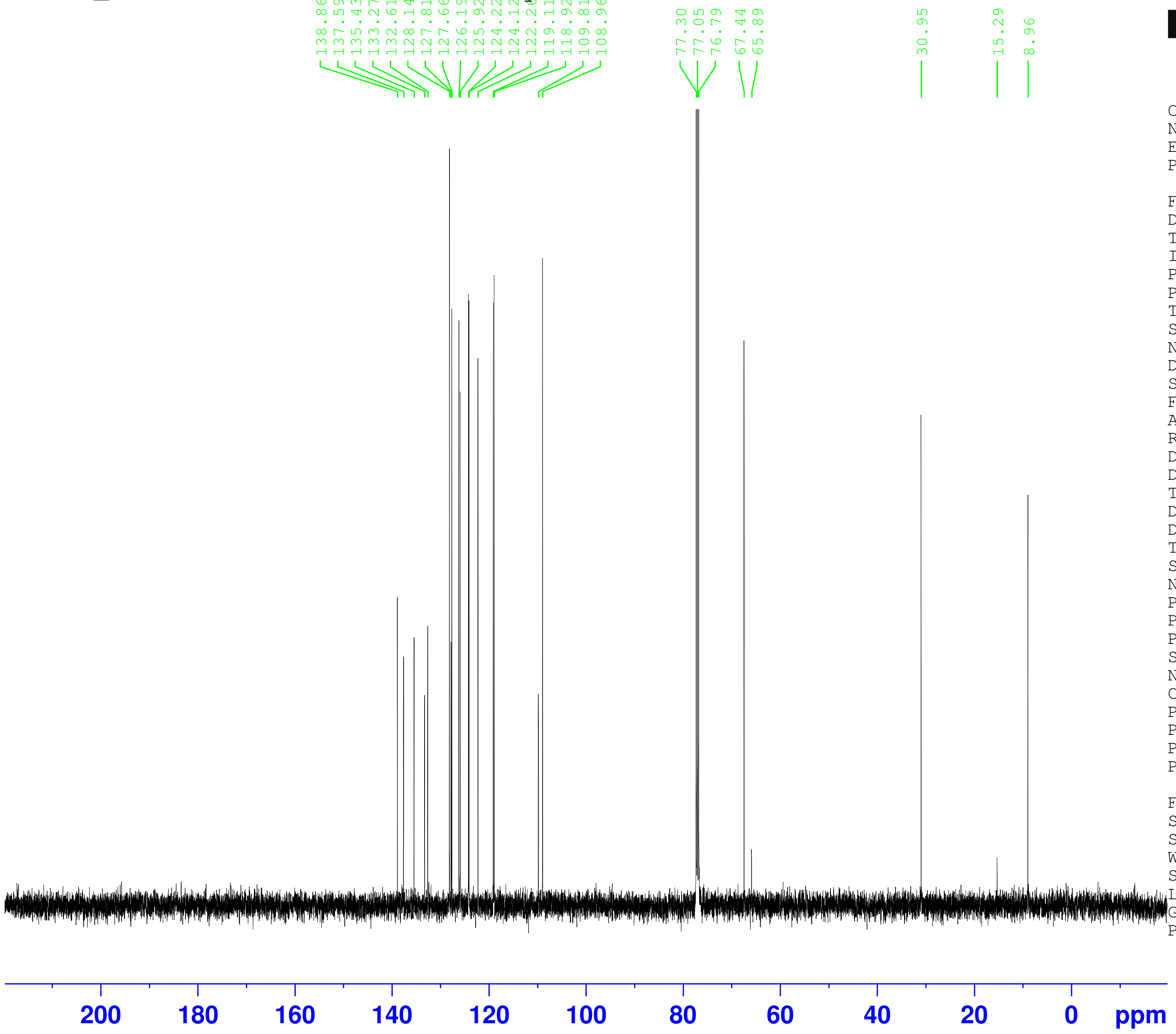


```
Current Data Parameters
NAME          EZ-1-33-CNMR
EXPNO                   12
PROCNO                   1

F2 - Acquisition Parameters
Date_             20240410
Time                 17.05 h
INSTRUM             Avance
PROBHD   Z169712_0016 (
PULPROG            zgpg30
TD                   65536
SOLVENT              CDCl3
NS                     256
DS                       1
SWH              30120.482 Hz
FIDRES            0.919204 Hz
AQ               1.0878977 sec
RG                     101
DW                  16.600 usec
DE                    6.50 usec
TE                   298.0 K
D1              2.00000000 sec
D11             0.03000000 sec
TD0                      1
SFO1           125.7703643 MHz
NUC1                   13C
P0                    3.00 usec
P1                    9.00 usec
PLW1          104.23000336 W
SFO2           500.1320005 MHz
NUC2                    1H
CPDPRG[2           waltz65
PCPD2                80.00 usec
PLW2           25.72999954 W
PLW12           0.25472999 W
PLW13           0.12813000 W

F2 - Processing parameters
SI                   32768
SF             125.7577885 MHz
WDW                     EM
SSB                      0
LB                    1.00 Hz
GB                       0
PC                    1.40
```
