# Supplementary material for: Friedel–Crafts Addition of 3‑Alkylated Indoles to Aldehydes: 2‑Hydroxyalkylation Promoted by Trimethylsilyl Trifluoromethanesulfonate
Source: J Org Chem. 2026 Apr 29;91(18):6255–67. doi: 10.1021/acs.joc.5c03195 (PMC13162324; doi:10.1021/acs.joc.5c03195)
Supplement: Supplementary file 2 [file jo5c03195_si_002.zip › FID for publication1/1h/column fractions/EZ-2-154c-FRAC_8-11/10/pdata/1/email_EZ-2-154c-FRAC_8-11_10_1.pdf]

Proton_UR CDCl3 {D:\nmrdata} wdowney 2

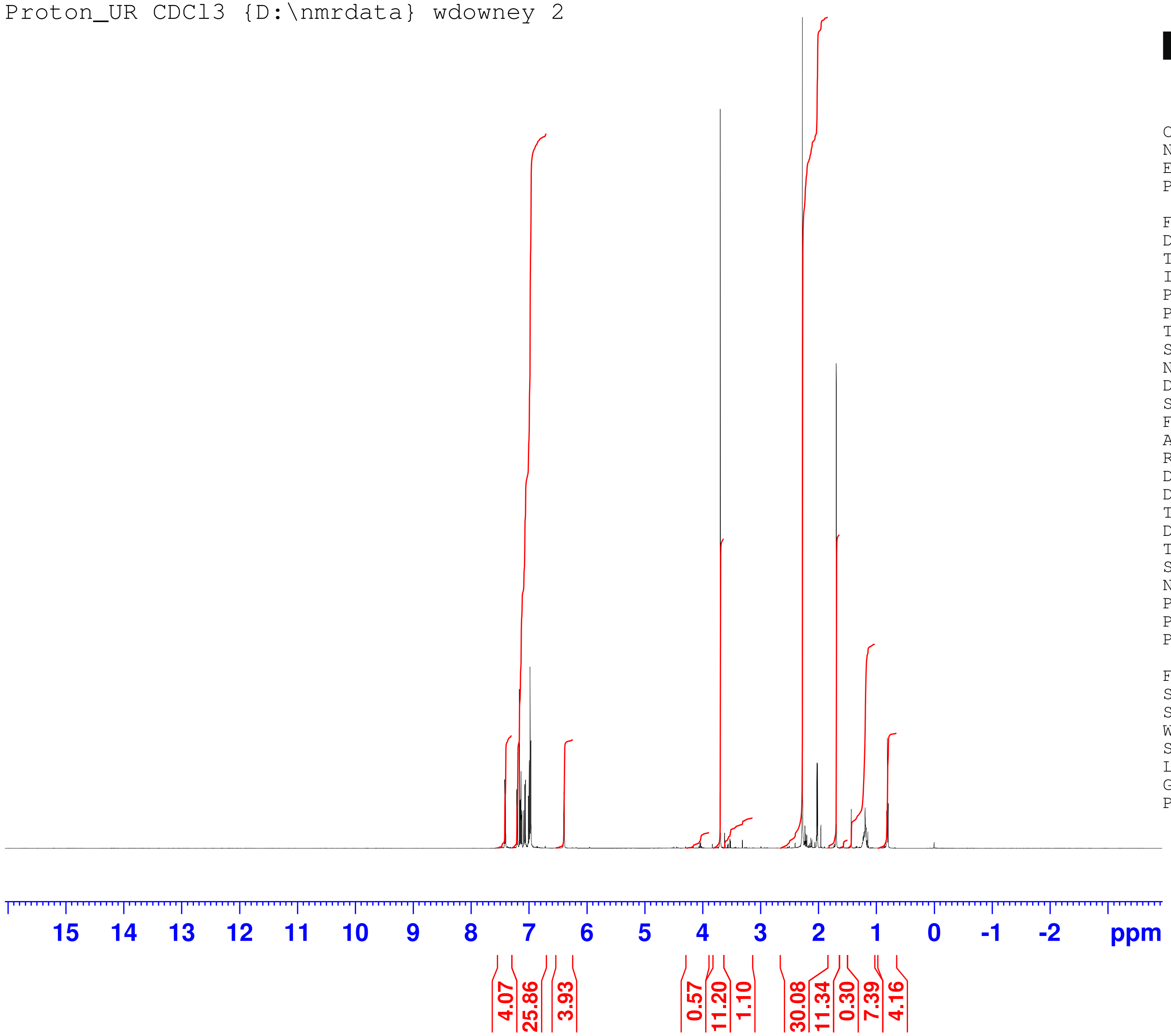


```
Current Data Parameters
NAME       EZ-2-154c-FRAC_8-11
EXPNO                       10
PROCNO                       1

F2 - Acquisition Parameters
Date_                 20250218
Time                     11.57 h
INSTRUM                 Avance
PROBHD       Z169712_0016 (
PULPROG                   zg30
TD                       65536
SOLVENT                  CDCl3
NS                          16
DS                           2
SWH                  10000.000 Hz
FIDRES                0.305176 Hz
AQ                   3.2767999 sec
RG                         101
DW                      50.000 usec
DE                       11.14 usec
TE                       305.0 K
D1                  1.00000000 sec
TD0                          1
SFO1               500.1330883 MHz
NUC1                        1H
P0                        2.65 usec
P1                        7.96 usec
PLW1               25.72999954 W

F2 - Processing parameters
SI                       65536
SF                 500.1300594 MHz
WDW                         EM
SSB                          0
LB                        0.30 Hz
GB                           0
PC                        1.00
```
