# Supplementary material for: Friedel–Crafts Addition of 3‑Alkylated Indoles to Aldehydes: 2‑Hydroxyalkylation Promoted by Trimethylsilyl Trifluoromethanesulfonate
Source: J Org Chem. 2026 Apr 29;91(18):6255–67. doi: 10.1021/acs.joc.5c03195 (PMC13162324; doi:10.1021/acs.joc.5c03195)
Supplement: Supplementary file 3 [file jo5c03195_si_003.zip › FID for publication3-5/3b/02EZ-3-50b-CNMR/12/pdata/1/email_EZ-3-50b-CNMR_12_1.pdf]

Carbon_UR CDCl3 {D:\nmrdata} wdowney 16

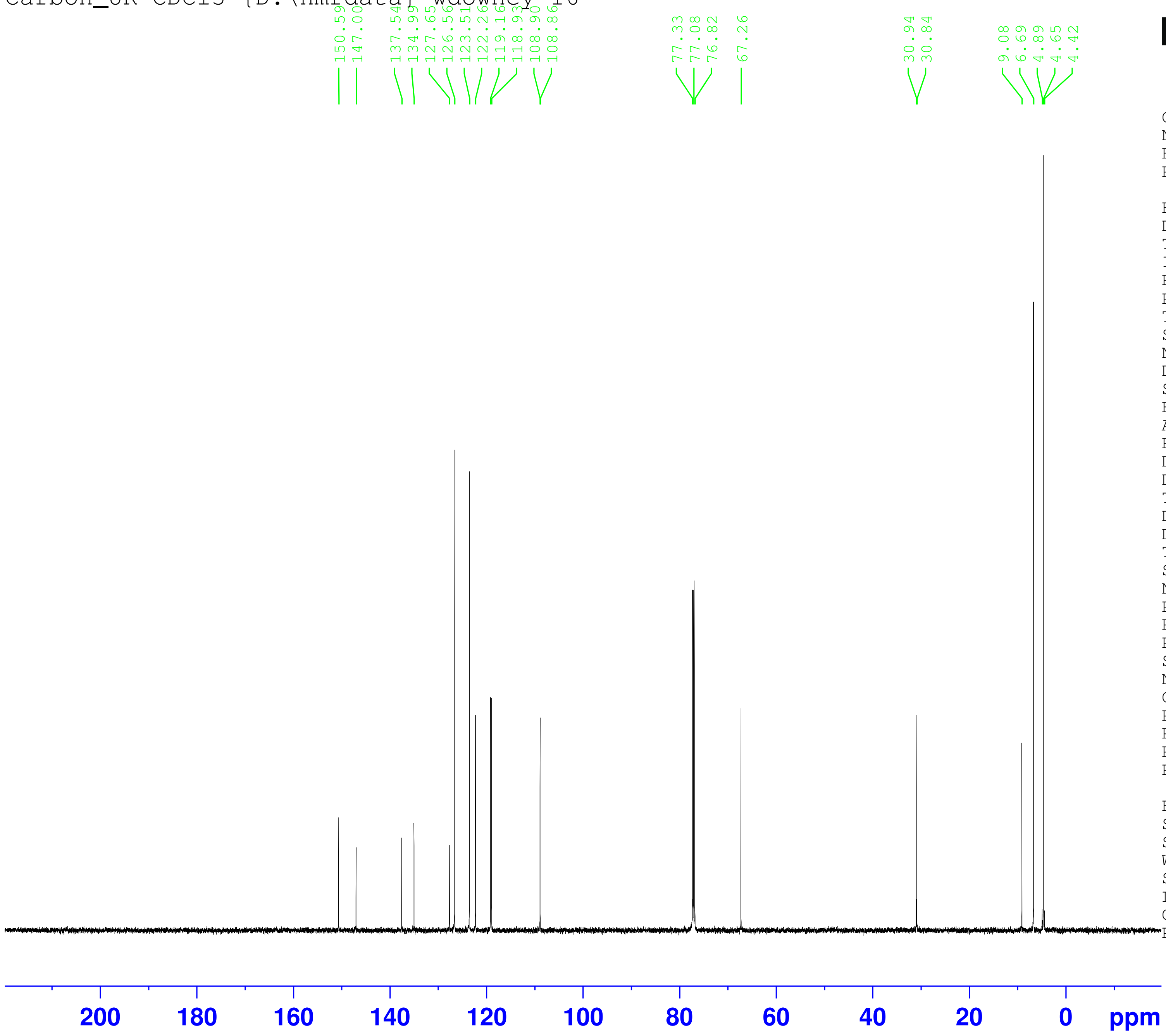

BRUKER

Current Data Parameters

NAME	EZ-3-50b-CNMR
EXPNO	12
PROCNO	1

F2 - Acquisition Parameters

Date_	20250522
Time	14.06 h
INSTRUM	Avance
PROBHD	Z169712_0016 (
PULPROG	zgpg30
TD	65536
SOLVENT	CDCl3
NS	256
DS	1
SWH	30120.482 Hz
FIDRES	0.919204 Hz
AQ	1.0878977 sec
RG	101
DW	16.600 usec
DE	6.50 usec
TE	295.7 K
D1	2.00000000 sec
D11	0.03000000 sec
TD0	1
SFO1	125.7703643 MHz
NUC1	13C
P0	3.00 usec
P1	9.00 usec
PLW1	104.23000336 W
SFO2	500.1320005 MHz
NUC2	1H
CPDPRG[2	waltz65
PCPD2	80.00 usec
PLW2	25.72999954 W
PLW12	0.25472999 W
PLW13	0.12813000 W

F2 - Processing parameters

SI	32768
SF	125.7577885 MHz
WDW	EM
SSB	0
LB	1.00 Hz
GB	0
PC	1.40
