# Supplementary material for: Friedel–Crafts Addition of 3‑Alkylated Indoles to Aldehydes: 2‑Hydroxyalkylation Promoted by Trimethylsilyl Trifluoromethanesulfonate
Source: J Org Chem. 2026 Apr 29;91(18):6255–67. doi: 10.1021/acs.joc.5c03195 (PMC13162324; doi:10.1021/acs.joc.5c03195)
Supplement: Supplementary file 3 [file jo5c03195_si_003.zip › FID for publication3-5/3d/13C EZ-2-126b-CNMR/12/pdata/1/email_EZ-2-126b-CNMR_12_1.pdf]

Carbon_UR CDCl3 {D:\nmrdata} wdowney 3

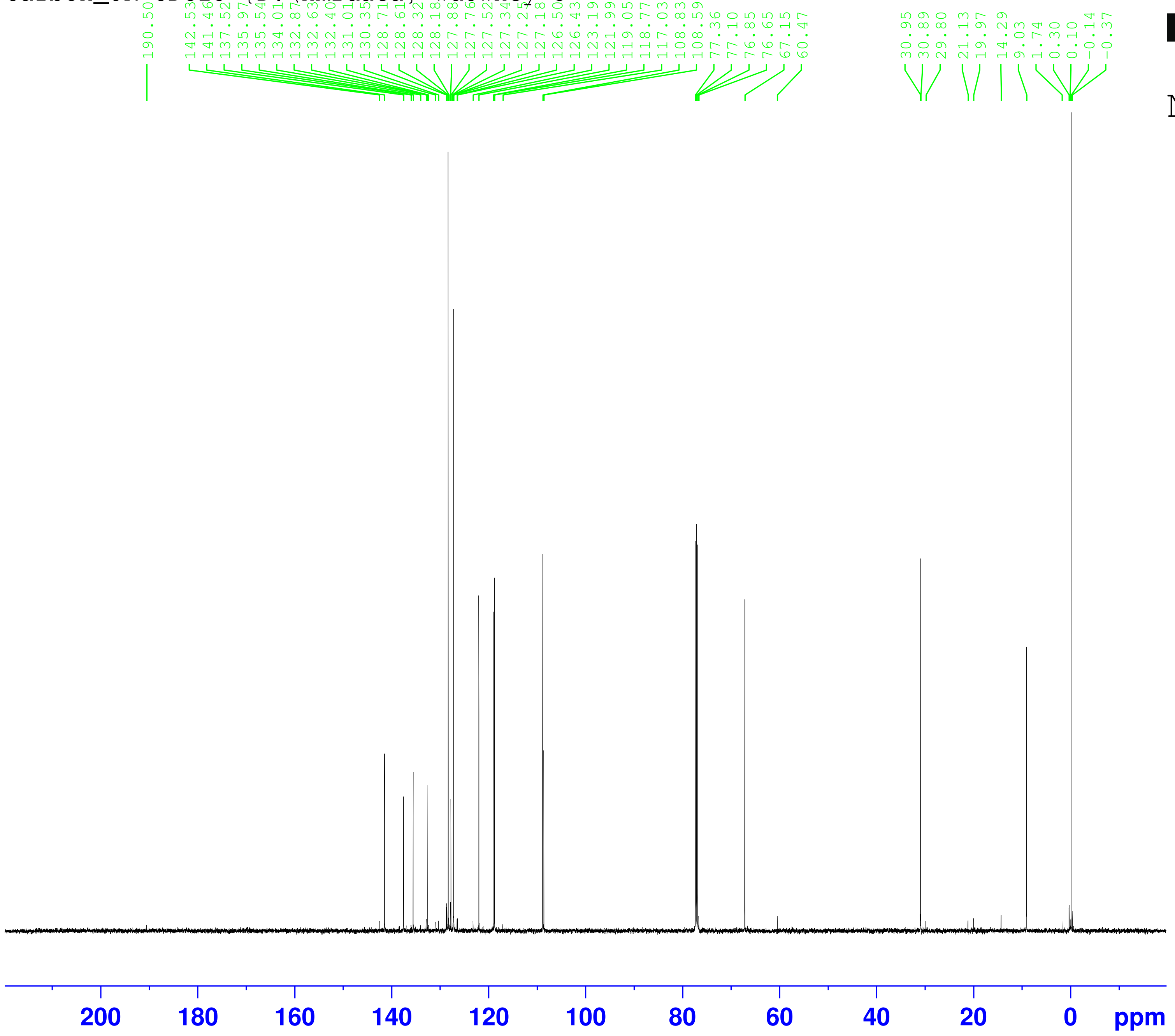

No parameters
