# Supplementary material for: Friedel–Crafts Addition of 3‑Alkylated Indoles to Aldehydes: 2‑Hydroxyalkylation Promoted by Trimethylsilyl Trifluoromethanesulfonate
Source: J Org Chem. 2026 Apr 29;91(18):6255–67. doi: 10.1021/acs.joc.5c03195 (PMC13162324; doi:10.1021/acs.joc.5c03195)
Supplement: Supplementary file 3 [file jo5c03195_si_003.zip › FID for publication3-5/4e/1H EZ-3-58c-HNMR-2ND/13/pdata/1/email_EZ-3-58c-HNMR-2ND_13_1.pdf]

Proton_UR CDCl3 {D:\nmrdata} wdowney 3

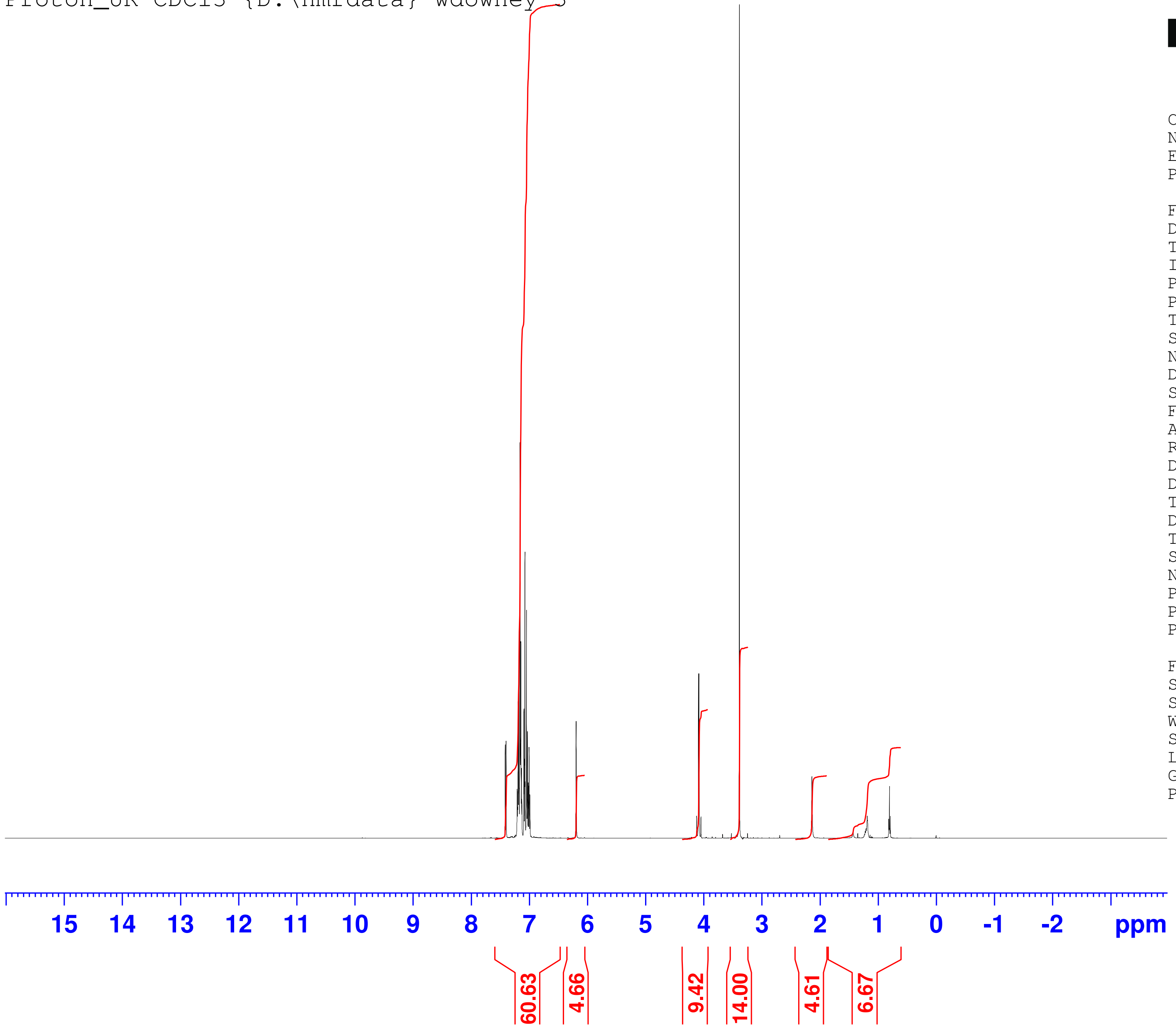

BRUKER

Current Data Parameters

NAME	EZ-3-58c-HNMR-2ND
EXPNO	13
PROCNO	1

F2 - Acquisition Parameters

Date_	20250603
Time	13.13 h
INSTRUM	Avance
PROBHD	Z169712_0016 (
PULPROG	zg30
TD	65536
SOLVENT	CDCl3
NS	16
DS	2
SWH	10000.000 Hz
FIDRES	0.305176 Hz
AQ	3.2767999 sec
RG	57
DW	50.000 usec
DE	11.14 usec
TE	300.0 K
D1	1.00000000 sec
TD0	1
SFO1	500.1330883 MHz
NUC1	1H
P0	2.65 usec
P1	7.96 usec
PLW1	25.72999954 W

F2 - Processing parameters

SI	65536
SF	500.1300758 MHz
WDW	EM
SSB	0
LB	0.30 Hz
GB	0
PC	1.00
