# Supplementary material for: Friedel–Crafts Addition of 3‑Alkylated Indoles to Aldehydes: 2‑Hydroxyalkylation Promoted by Trimethylsilyl Trifluoromethanesulfonate
Source: J Org Chem. 2026 Apr 29;91(18):6255–67. doi: 10.1021/acs.joc.5c03195 (PMC13162324; doi:10.1021/acs.joc.5c03195)
Supplement: Supplementary file 3 [file jo5c03195_si_003.zip › FID for publication3-5/3a/13C EZ-2-97b-CNMR/13/pdata/1/email_EZ-2-97b-CNMR_13_1.pdf]

Carbon_UR CDCl3 {D:\nmrdata} wdowney 3

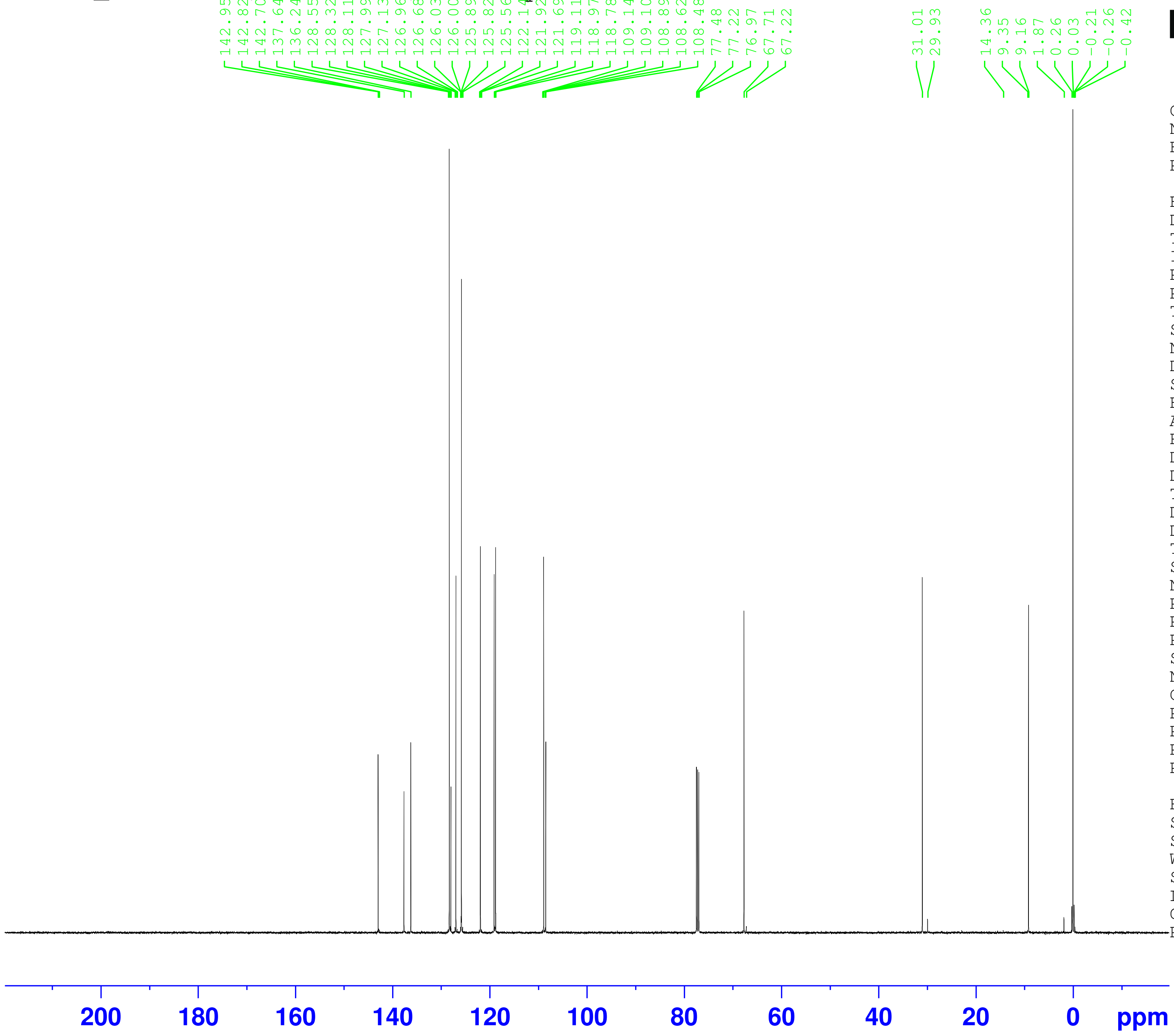

Current Data Parameters
NAME EZ-2-97b-CNMR
EXPNO 13
PROCNO 1

F2 - Acquisition Parameters
Date_ 20240923
Time 10.23 h
INSTRUM Avance
PROBHD Z169712_0016 (
PULPROG zgpg30
TD 65536
SOLVENT CDCl3
NS 256
DS 1
SWH 30120.482 Hz
FIDRES 0.919204 Hz
AQ 1.0878977 sec
RG 101
DW 16.600 usec
DE 6.50 usec
TE 295.4 K
D1 2.00000000 sec
D11 0.03000000 sec
TD0 1
SFO1 125.7703643 MHz
NUC1 13C
P0 3.00 usec
P1 9.00 usec
PLW1 104.23000336 W
SFO2 500.1320005 MHz
NUC2 1H
CPDPRG[2 waltz65
PCPD2 80.00 usec
PLW2 25.72999954 W
PLW12 0.25472999 W
PLW13 0.12813000 W

F2 - Processing parameters
SI 32768
SF 125.7577885 MHz
WDW EM
SSB 0
LB 1.00 Hz
GB 0
PC 1.40
